# Insights into LiAlH_4_ Catalyzed Imine Hydrogenation

**DOI:** 10.1002/chem.202003862

**Published:** 2020-11-26

**Authors:** Holger Elsen, Jens Langer, Gerd Ballmann, Michael Wiesinger, Sjoerd Harder

**Affiliations:** ^1^ Inorganic and Organometallic Chemistry University Erlangen-Nürnberg Egerlandstrasse 1 91058 Erlangen Germany

**Keywords:** DFT, heterobimetallic, hydride, hydrogenation, imine

## Abstract

Commercial LiAlH_4_ can be used in catalytic quantities in the hydrogenation of imines to amines with H_2_. Combined experimental and theoretical investigations give deeper insight in the mechanism and identifies the most likely catalytic cycle. Activity is lost when Li in LiAlH_4_ is exchanged for Na or K. Exchanging Al for B or Ga also led to dramatically reduced activities. This indicates a heterobimetallic mechanism in which cooperation between Li and Al is crucial. Potential intermediates on the catalytic pathway have been isolated from reactions of MAlH_4_ (M=Li, Na, K) and different imines. Depending on the imine, double, triple or quadruple imine insertion has been observed. Prolonged reaction of LiAlH_4_ with PhC(H)=N*t*Bu led to a side‐reaction and gave the double insertion product LiAlH_2_[N]_2_ ([N]=N(*t*Bu)CH_2_Ph) which at higher temperature reacts further by *ortho*‐metallation of the Ph ring. A DFT study led to a number of conclusions. The most likely catalyst for hydrogenation of PhC(H)=N*t*Bu with LiAlH_4_ is LiAlH_2_[N]_2_. Insertion of a third imine via a heterobimetallic transition state has a barrier of +23.2 kcal mol^−1^ (Δ*H*). The rate‐determining step is hydrogenolysis of LiAlH[N]_3_ with H_2_ with a barrier of +29.2 kcal mol^−1^. In agreement with experiment, replacing Li for Na (or K) and Al for B (or Ga) led to higher calculated barriers. Also, the AlH_4_^−^ anion showed very high barriers. Calculations support the experimentally observed effects of the imine substituents at C and N: the lowest barriers are calculated for imines with aryl‐substituents at C and alkyl‐substituents at N.

## Graphical abstract

**LiAlH_4_ catalysis**: A combined experimental and theoretical investigation on LiAlH_4_ catalyzed imine hydrogenation reveals mechanistic details as well as metal and substrate effects.

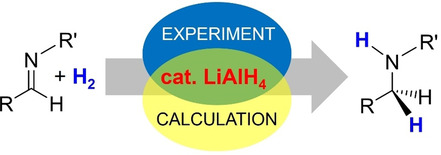

## Introduction

Since its first synthesis LiAlH_4_ has become one of the most commonly used reducing agents. Saline lithium hydride (LiH)_∞_ is essentially unreactive towards double bonds of any kind due to its high lattice energy and low solubility in organic solvents.^[1]^ Aluminium hydride (AlH_3_) is in contrast highly reactive but even as it ether complex it decomposes easily in its elements.^[2]^ Its combination LiAlH_4_, however, is stable and highly reactive and has since its discovery in 1947 been developed into a very useful reducing agent.^[3]^ This commercially available metal hydride source is well soluble in ethereal solvents and reacts readily with polar C=O bond in aldehydes, ketones and carboxylic acids.^[4]^ Nitriles react violently with LiAlH_4_ and, under more forcing conditions, even reduction of the C=N bond in imines can be achieved. Despite the requirement of elevated temperatures, main group metal‐mediated imine transformations are of prime industrial importance.^[5]^

Although these applications are based on stoichiometric use of LiAlH_4_, the last decades have seen some interesting examples of LiAlH_4_ (or related compounds) in catalysis.[^6^, ^7^, ^8^, ^9^, ^10^, ^11^, ^12^, ^13^] During the development of early main group metal catalyzed imine hydrogenation,^[14]^ we found that commercially available LiAlH_4_ can be used under relatively mild conditions in catalytic instead of stoichiometric quantities (2.5 mol % catalyst loading, 1 bar H_2_ and 85 °C).[^13^, ^15^] Such a non‐stoichiometric route prevents the generally hazardous aqueous work‐up and avoids considerable amounts of Li/Al salts as side‐products. Noteworthy is the fact that reactions could be carried out in neat imine, without additional solvents. This eliminates the need for rigorously solvent drying and makes the procedure highly atom economical and environmentally benign.

While LiAlH_4_ performed well in imine hydrogenation catalysis, homologues such as NaAlH_4_ and NaBH_4_ were shown to be much less active.^[13]^ This clearly shows that not only the nature of the hydride source (Al−H or B−H) but also the alkali metal (Li or Na) influence catalytic activity. Most recently, we introduced group 2 metal alanates, Ae(AlH_4_)_2_ (Ae=alkaline earth), in imine hydrogenation catalysis.^[16]^ Although Mg(AlH_4_)_2_ is less active than LiAlH_4_, the heavier alanates Ca(AlH_4_)_2_, and especially Sr(AlH_4_)_2_, showed high activities, considerably broadening the substrate scope. As the salt [*n*Bu_4_N^+^][AlH_4_^−^] was found to be essentially inactive,^[16]^ the presence of the *s*‐block metal is crucial. This strong indication for a heterobimetallic mechanism is supported by a comprehensive study by the Mulvey group.^[17]^ Comparison of the activities of neutral and anionic aluminium hydride compounds in hydroboration catalysis, clearly suggest a heterobimetallic mechanism.

**Scheme 1:**
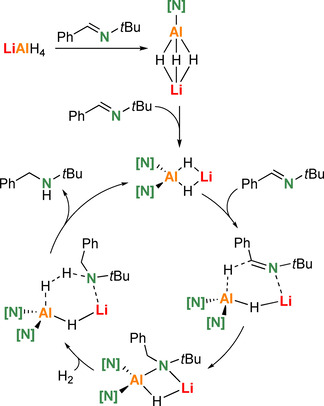
Proposed catalytic cycle for the hydrogenation of Ph(H)C=N*t*Bu by precatalyst LiAlH_4_; [N]=N(*t*Bu)CH_2_Ph.

We proposed a mechanism in which LiAlH_4_ first reacts with two equivalents of imine to give a mixed hydride/amide complex LiAlH_2_[N]_2_ which is the actual catalyst (Scheme 1, [N]=N(*t*Bu)CH_2_Ph). This assumption is based on the fact that the second imine insertion is generally faster than the first while the third insertion is more difficult.^[13]^ This does not only hold for LiAlH_4_/imine reactivity but also for LiAlH_4_/R_2_NH deprotonations and explains nicely why there are many isolated examples of complexes like LiAlH_2_[N]_2_.[^11^, ^18^, ^19^, ^20^, ^21^, ^22^] Starting with LiAlH_2_[N]_2_ as a catalyst, the first step is insertion of a third imine. We proposed this to be a heterobimetallic process in which imine‐Li coordination activated the C=N bond for nucleophilic attack by the Al−H unit. This is followed by amine formation in the reaction of LiAlH[N]_3_ with H_2_. Since a slight increase in H_2_ pressure has an accelerating effect,^[13]^ this hydrogenation step was proposed to be the rate‐determining most difficult step.

In this contribution, we report additional experimental proof for such a heterobimetallic mechanism and support our observations with a comprehensive computational study.

## Results and Discussion

### Metal modifications

We recently presented that group 2 metal alanates, Ae(AlH_4_)_2_, are considerably better catalysts with activities increasing in the order Ae=Mg>Ca>Sr.^[16]^ Herein, we explore further metal modifications of both, the *s*‐ and *p*‐block metals.

While NaAlH_4_ was found to be less active than LiAlH_4_,^[13]^ we became interested in exploring KAlH_4_ in imine hydrogenation. Analogue to Schlesinger's first synthesis of LiAlH_4_ ^[3]^ and NaAlH_4_,^[23]^ it was attempted to prepare KAlH_4_ by reaction of four equivalents of KH with one equivalent of AlCl_3_. This gave due to solubility problems incomplete H/Cl exchange. More effective was the reaction of KH with AlH_3_⋅(THF)_2_. The product was only sparingly soluble in THF but dissolved sufficiently in imine PhC(H)=N*t*Bu to obtain ^1^H and ^27^Al NMR data (see Figure S1). These data confirm a THF‐free product containing the AlH_4_^−^ ion; the ^27^Al NMR spectrum shows a quintet with ^1^ *J*_Al‐H_=168 Hz. While neither pure KH nor AlH_3_(THF)_2_ was able to catalyze imine reduction, the catalyst KAlH_4_ led to product formation but only in trace quantities. Compared to the activities of LiAlH_4_ and NaAlH_4_ (Table 1, entries 1–3), it is clear that catalyst performance decreases with the size of the alkali metal: Li>Na>K. Since KAlH_4_ is notably insoluble, this may also be related to solubility. The significantly better soluble complex KAlH_4_⋅[(18‐crown‐6)/THF] was prepared according to literature.^[24]^ While its performance is far better than that of neat KAlH_4_ (cf. entries 3–4), it is less active than LiAlH_4_. This implies that the *s*‐block metal requires some Lewis acidity.

**Table 1:** Selected bond lengths (Å) and angles (°) for complexes **1**–**6**; (*μ*‐H)=bridging hydride and H_t_=terminal hydride.

Complex	**1**	**2**	**3**	**4**	**5**	**6**
M−(*μ*‐H)	–	2.37(2)	2.30(3)	2.68(3) 3.04(3)	1.88(2) 1.89(2)	–
Al−(*μ*‐H)	–	1.57(2)	1.66(3)	1.54(3) 1.57(3)	1.60(2) 1.61(2)	–
Al−H_t_	1.57(2)	1.53(2)	1.56(3)	–	–	–
M−Al	–	3.6082(9)	3.7112(8)	3.498(3)	2.598(2)	–
Al−N	1.879(2) 1.885(1) 1.889(1)	1.861(2) 1.876(2)	1.588(1) 1.860(1)	1.857(2) 1.876(2)	1.852(2) 1.864(1)	1.879(6)–1.893(3)
N‐Al‐N	109.10(6) 110.70(6) 111.79(6)	117.54(7)	118.62(6)	119.0(1)	122.63(7)	107.6(2)–112.0(2)
M‐(*μ*‐H)‐Al	–	131(1)	138(1)	94(1) 108(1)	95.8(8) 95.9(9)	–

As we previously found that the borate NaBH_4_ is essentially inactive in imine hydrogenation,^[13]^ we became interested in exchanging the *p*‐block metal Al for Ga. Although the synthesis of LiGaH_4_ and NaGaH_4_ has been described by Wiberg as early as 1951,^[25]^ attempts to use these salts in catalysis gave already at room temperature decomposition in Ga and LiH (or NaH). The more stable TMEDA adducts [Li^+^(TMEDA)_2_][GaH_4_^−^] [Na^+^(TMEDA)_2_][GaH_4_^−^] could easily be prepared and isolated according to Bakum et al.,^[26]^ however, these were found to be insoluble in THF and gave after 24 hours at 85 °C only sub‐stoichiometric imine‐to‐amine conversion (entries 5–6).

### Isolation of intermediates

**Figure 1:**
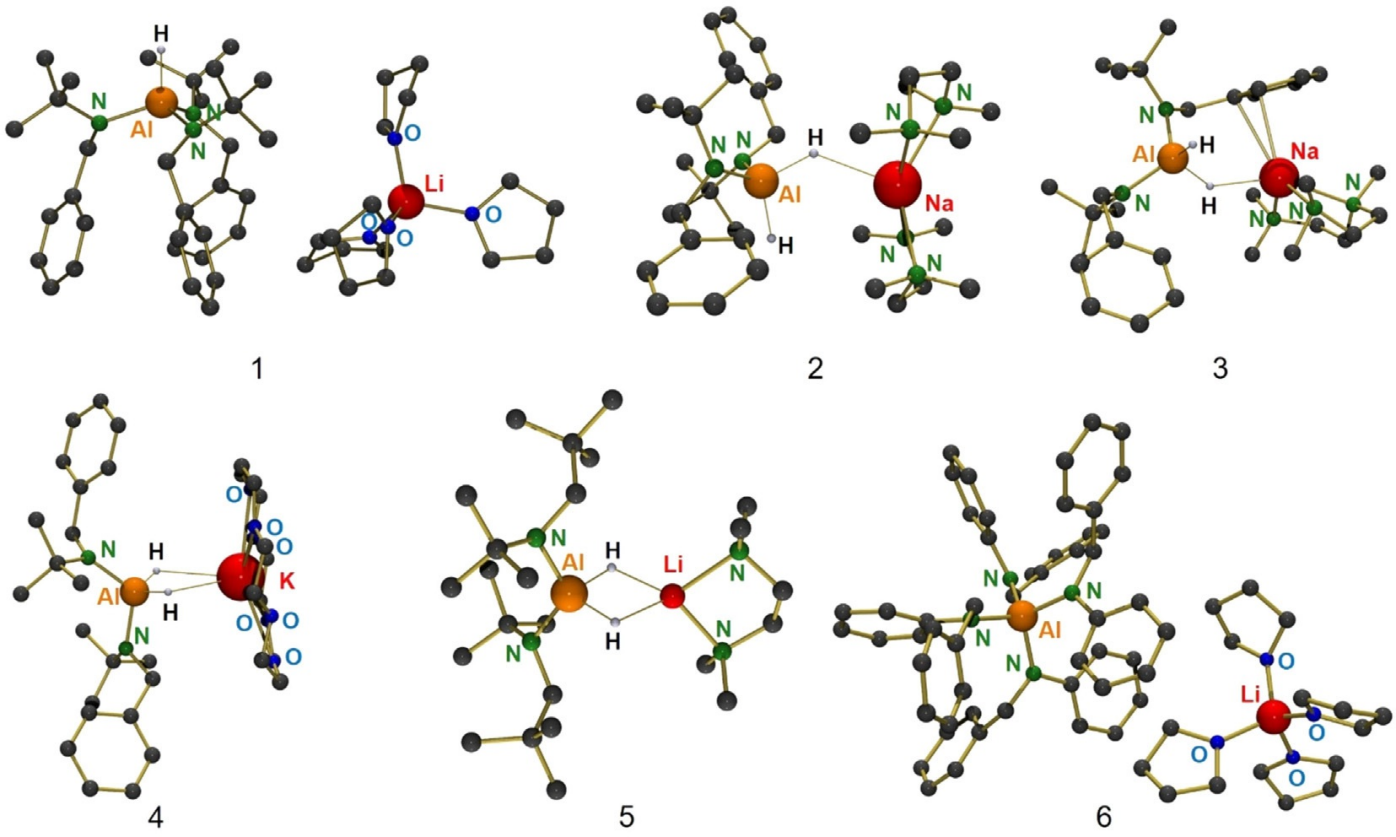
Crystal structures of complexes **1**–**6**; H atoms only partially shown. Selected bond distances and angles are summarized in Table 1.

According to the proposed catalytic cycle in Scheme 1, the first step is a double addition of imine to LiAlH_4_ to give LiAlH_2_[N]_2_; [N]=N(*t*Bu)CH_2_Ph. Indeed, reaction of LiAlH_4_ with one equivalent of imine gave under catalytic conditions a mixture of unreacted LiAlH_4_, LiAlH_3_[N] and LiAlH_2_[N]_2_. This implies that the second addition is faster than the first. While clean isolation of the single addition product is difficult to achieve, reaction of LiAlH_4_ with two equivalents of imine cleanly gave LiAlH_2_[N]_2_, which we previously isolated in the form of the TMEDA and PMDTA complexes LiAlH_2_[N]_2_⋅[TMEDA]_2_ (**I**) and LiAlH_2_[N]_2_⋅[PMDTA] (**II**).^[13]^ This high preference for formation of the double addition product is generally observed.[^11^, ^18^, ^19^, ^20^, ^21^, ^22^] Addition of a third imine needs much longer reaction times and an excess of imine. We now introduce the isolation and characterization of LiAlH[N]_3_⋅[THF]_4_ (**1**) obtained after long term reaction of LiAlH_4_ with an eightfold excess of imine at room temperature. Its crystal structure (Figure 1, Table 2) shows a solvent‐separated‐ion‐pair.

**Table 2:** Catalytic hydrogenation of imines.

Entry	Substrate	Catalyst (mol %)	*T* [°C]	H_2_ [bar]	*t* [h]	Conversion [%]^[a]^
1	PhC(H)=N*t*Bu	LiAlH_4_ (5)^[13]^	85	6	6	>99
2	PhC(H)=N*t*Bu	NaAlH_4_ (5)^[13]^	85	6	6	77
3	PhC(H)=N*t*Bu	KAlH_4_ (5)	85	20	24	traces
4	PhC(H)=N*t*Bu	KAlH_4_⋅[18‐crown‐6/THF] (5)	85	20	24	46
5	PhC(H)=N*t*Bu	LiGaH_4_⋅[TMEDA]_2_ (10)	85	20	24	traces
6	PhC(H)=N*t*Bu	NaGaH_4_⋅[TMEDA]_2_ (10)	85	20	24	3
7	*t*BuC(H)=N*t*Bu	LiAlH_4_ (5)^[13]^	85	6	96	66
8	PhC(H)=NPh	LiAlH_4_ (5)	85	6	96	13

[a] Conversion determined by ^1^H NMR.

**Figure 2:**
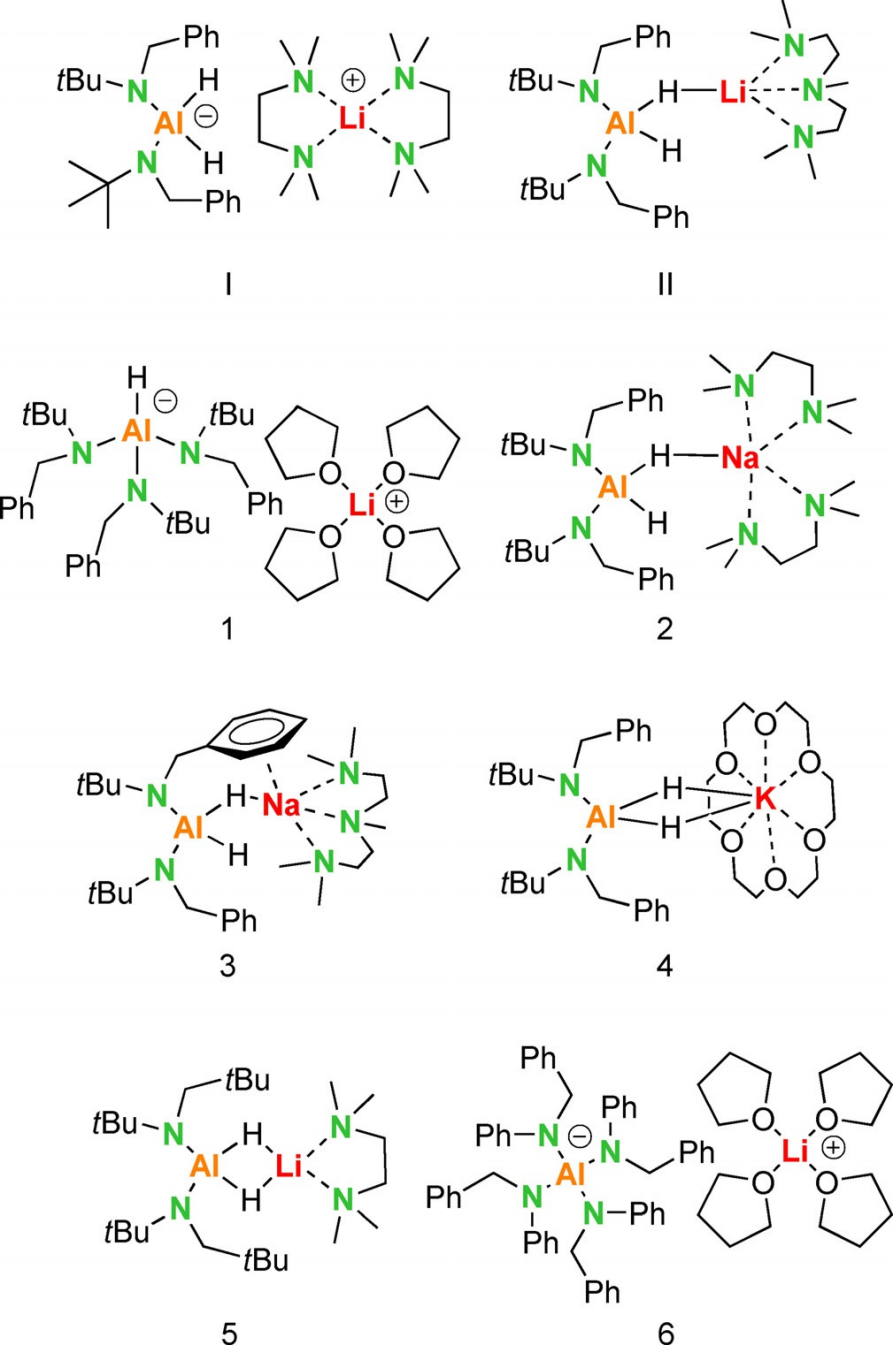
Experimentally confirmed group 1 metal aluminate complexes with amide ligands.

We also isolated intermediates along the catalytic pathway for alanate catalysts with the heavier *s*‐block metals Na and K. Reaction of NaAlH_4_ with two equivalents of PhC(H)=N*t*Bu gave double addition and products were isolated in the form of the TMEDA and PMDTA complexes NaAlH_2_[N]_2_⋅[TMEDA]_2_ (**2**) and NaAlH_2_[N]_2_⋅[PMDTA] (**3**). Their crystal structures (Figure 1, Table 2) show that these Na alanates have the same composition as the comparable Li alanates **I** and **II**. Due to the larger size of Na^+^ vs. Li^+^, there are additional contacts between Na^+^ and the AlH_2_[N]_2_^−^ ion. Reaction of KAlH_4_ with two equivalents of PhC(H)=N*t*Bu gave in the presence of 18‐crown‐6 the intermediate KAlH_2_[N]_2_⋅[18‐crown‐6] (**4**) which despite extensive solvation of K^+^ by the crown ether shows an intimate Al(μ‐H)_2_  K contact (Figure 1, Table 2); a similar [(Me_3_Si)_2_ n]_2_Al(*μ*‐H)_2_Li⋅(12‐crown‐4) complex was isolated by the Mulvey and Hevia groups (Figure 2).^[11]^

Apart from intermediates with the standard imine PhC(H)=N*t*Bu, which has been the benchmark in catalyst screening, also intermediates with other imines have been isolated. As previously reported, catalytic reduction of *t*BuC(H)=N*t*Bu is much more sluggish (Table 1, entry 7)^[13]^ because the C=N bond in this bis‐alkylated imine is not activated by conjugation with Ph. Under forced conditions, however, the double addition product was formed and could be crystallized in the form of LiAlH_2_[N(*t*Bu)CH_2_ *t*Bu]_2_⋅(TMEDA) (**5**). Despite two equivalents of TMEDA have been used, a contact‐ion‐pair with only one TMEDA ligand and an intimate Al(*μ*‐H)_2_Li contact crystallized (Figure 1, Table 2). This could be understood by the strongly electron‐releasing character of the *t*BuCH_2_(*t*Bu)N^−^ ions that makes the AlH_2_ unit much more hydridic. Since the C=N bond in PhC(H)=NPh is activated by conjugation over an extended π‐system, addition to LiAlH_4_ was found to be extremely fast. In this case we have been able to isolate the fourfold addition product LiAl[N(Ph)CH_2_Ph]_4_⋅(THF)_4_ (**6**) which crystallized as a solvent‐separated‐ion‐pair (Figure 1, Table 2).

### Possible pathways for catalyst deactivation

The thermal decomposition of LiAlH_4_ is well investigated, especially in the context of its potential in hydrogen storage.^[27]^ Deactivation and thermal decomposition of intermediates in the catalytic cycle for LiAlH_4_ catalyzed imine hydrogenation, however, has so far not been investigated.

**Figure 3:**
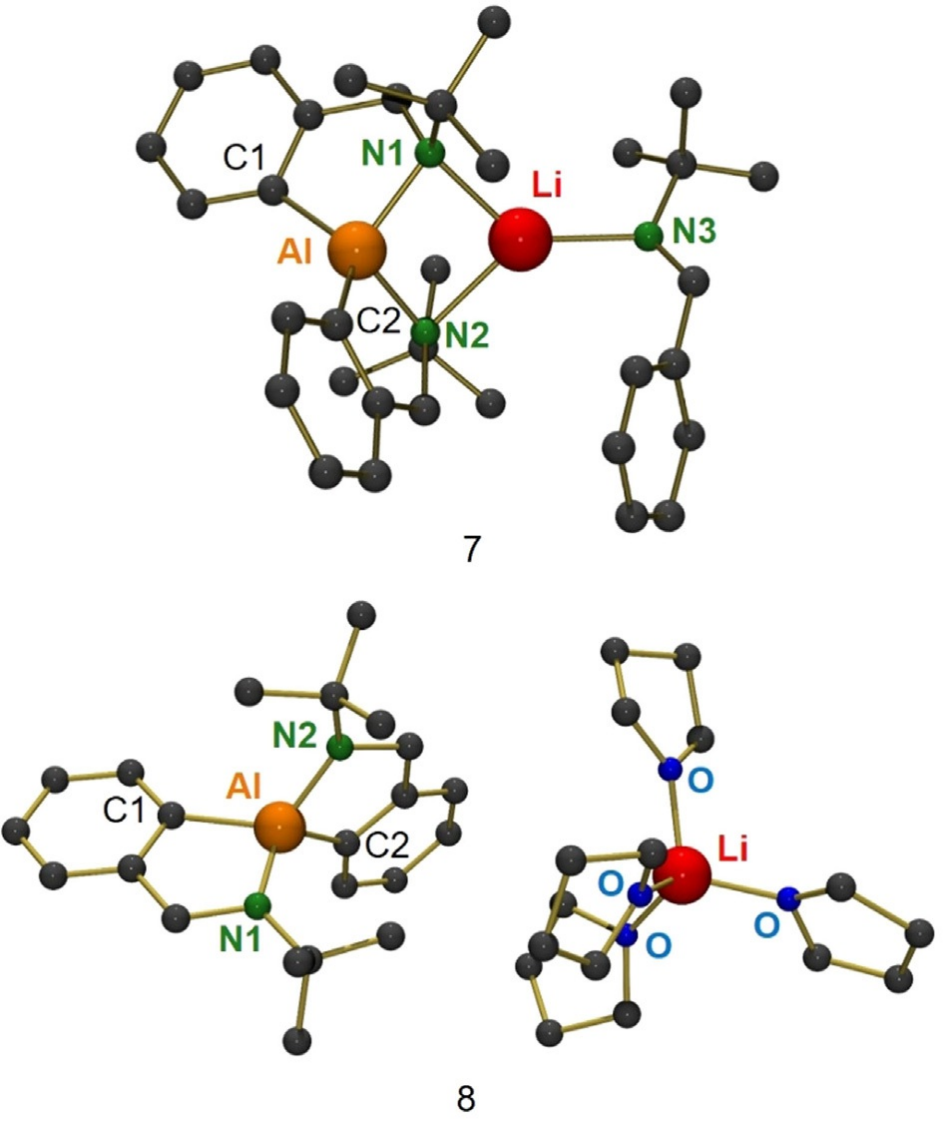
Crystal structures of complexes **7** and **8**; H atoms not shown. In **7**, the imine ligand bound to Li is disordered with the amine (here the imine is shown). Selected bond lengths (Å): **7**: Al−Li: 2.667(7), Al−C1: 1.969(4), Al−C2: 1.970(5) Al−N1: 1.895(3), Li−N1: 2.173(8), Al−N2: 1.935(3), Li−N2: 2.092(8), Li−N3: 2.125(9). **8**: Al−C1: 1.997(3), Al−N1: 1.861(2), Al−N2: 1.868(3), Al−C2: 1.999(3).

To this purpose, LiAlH_4_ was reacted with an excess of PhC(H)=N*t*Bu imine at 85 °C for multiple days. X‐ray analysis of crystals obtained from this reaction mixture showed that reduction of the imine to amide has been followed by *ortho*‐metallation. Complex **7** (Figure 3) is comprised of an alanate anion with two C,N‐chelating ligands and a Li^+^ cation that bridges both N atoms and is additionally solvated by an imine. The latter neutral ligand is heavily disordered with reduced imine: PhCH_2_N(H)*t*Bu. This illustrates that deprotonation in the *ortho*‐Ph position proceeds by an amide base. Alternatively, *ortho*‐metallation takes place by the Al‐H functionality and H_2_ gas produced is responsible for amine formation by hydrogenolysis. The selective *ortho*‐alumination with mixed Li/Al‐amide bases has been reported previously by the Mulvey and Hevia groups.[^28^, ^29^]

Recrystallization of **7** from a THF/hexane mixture gave complex **8**, a solvent‐separated‐ion‐pair (Figure 3). Complexes **7** and **8** do not react with H_2_ (1 bar, 80 °C, 5d) to give AlH_4_^−^ or other hydride species. This implies that the here observed *ortho*‐metallation could be a catalyst deactivation pathway. Complex **8** was isolated in crystalline form in yields up to 48 %. This combined imine insertion and subsequent *ortho*‐metallation protocol may be exploited in synthesis.

### DFT calculations

Given the complexity of the system with two different metals (Li and Al), two different anions (amide and hydride), the generally high dynamics of polar complexes and the importance of solvation, it is clear that any calculational study on LiAlH_4_ catalyzed imine hydrogenation is challenging. Aim of this comprehensive study is to gain detailed information on possible reaction mechanisms and to answer following questions. What is the catalytically active species: LiAlH_4_, LiAlH_3_[N], LiAlH_2_[N]_2_ or LiAlH[N]_3_ (or a mixture of these)? Is this true heterobimetallic catalysis and what is the role of the two metals? What is the influence of varying the *s*‐block metal (Li, Na, K) or the *p*‐block metal (Al, Ga, B)? Can we understand why PhC(H)=N*t*Bu is a preferred substrate while *t*BuC(H)=N*t*Bu and especially PhC(H)=NPh are much harder to reduce?

Energy profiles for the catalytic pathways have been calculated by DFT theory at the B3PW91/6‐311++G**//6‐31+G** level. Since solvent effects in these polar reactions can be important, corrections have been applied using the polarizable continuum model (PCM) simulating THF (*ϵ*=7.4257). All energy profiles show Δ*H* values in kcal mol^−1^. Computational methods overestimate the entropic factors and therefore Δ*G* values for energy barriers of reactions with entropy loss are calculated too high.^[30]^ For completeness, energy profiles with Δ*G* values can be found in Supporting Information (Schemes S3–S7). Minima and transition states (marked by *) have been identified by frequency calculation.

**Scheme 2:**
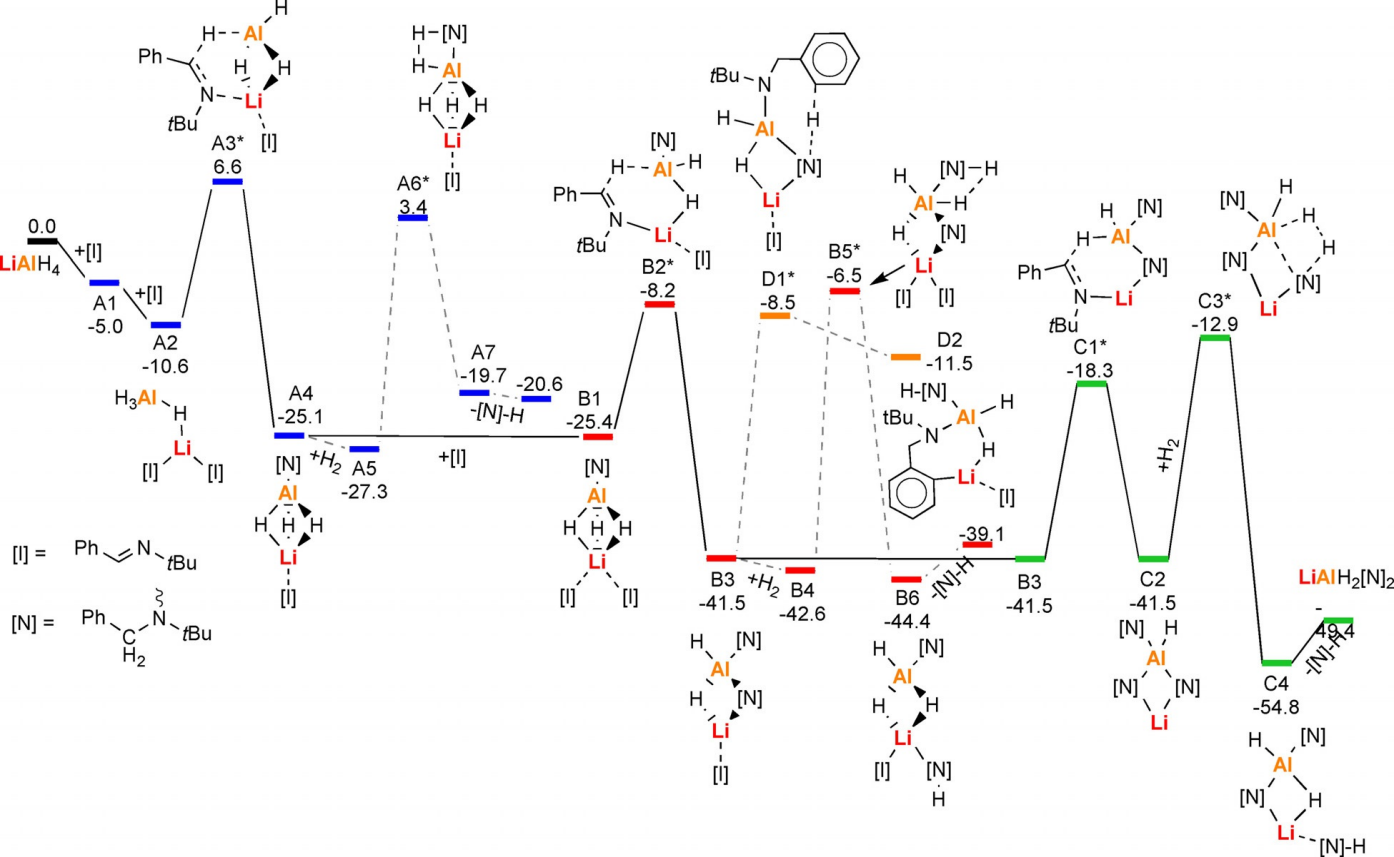
Full energy profile for the hydrogenation of PhC(H)=N*t*Bu with LiAlH_4_ using the imine as explicit solvent (B3PW91/6–311++G**(PCM=THF)//6–31+G**); ΔH in kcal mol^−1^. Pathways A (blue) and B (red) show the formation of the active catalyst: LiAlH_2_[N]_2_ (B_3_). Pathway C (green) represents the catalytic cycle for B3. is the catalytic cycle. Path D (grey) shows a pathway for catalyst decomposition by ortho‐metalation. The most likely intermediates are connected by solid lines.

In an explorative study on a very simple model system, that is, the hydrogenation of MeC(H)=NMe with LiAlH_4_, we found that solvation of the Li^+^ cation is highly important (see Scheme S1). This preliminary study demonstrated that modelling a polar reaction medium only with PCM was not sufficient. Energy barriers dropped significantly when solvation effects were explicitly modelled with solvent molecules as well. Therefore, in a second step we calculated the full energy profile for LiAlH_4_ catalyzed hydrogenation of our benchmark substrate PhC(H)=N*t*Bu, using this imine also for solvation (Scheme 2). Starting with LiAlH_4_, in which Li^+^ is bound by HAl(*μ*‐H_3_)Li bridging, solvation of Li^+^ with imine [I] is exothermic by 5.0 kcal mol^−1^ (**A1**). While coordination with the imine ligand keeps the Al(*μ*‐H_3_)Li bridge intact, solvation with a second imine cleaves two Li‐H contacts resulting in Al(*μ*‐H)Li bridging (**A2**). Imine reduction proceeds through a transition state (**A3**) in which the imine is activated by *N*‐Li coordination and attacked by an Al‐H unit. The amide [N], formed after reduction, is bound to Al in a terminal position and Al(*μ*‐H_3_)Li bridging is restored. With +17.2 kcal mol^−1^, the activation energy needed for this heterobimetallic process is only moderate. Subsequent amide→amine transformation by reaction with H_2_ has a much higher barrier (**A5→A6**: +30.7 kcal mol^−1^) and is endothermic (**A5→A7**: +7.6 kcal mol^−1^). Instead of following this high energy path, it is clearly easier to insert a second imine (B1→B2*: +17.2 kcal mol^−1^). In product **B3**, the Al and Li cations are bridged by an amide and hydride. Hydrogenolysis of the terminal amide by H_2_ is again a high energy process (B4→B5*: +36.1 kcal mol^−1^). The alternative reaction of the bridging amide with H_2_ has an even higher barrier (38.3 kcal mol^−1^) and insertion of a third imine is clearly preferred (B3→C1*: +23.2 kcal mol^−1^). The product **C2** is rather crowded and attempts to find a transition state for insertion of a fourth imine failed due to space limitation. Attempts to optimize the geometry of LiAl[N]_4_ led to dissociation into Li[N] and Al[N]_3_, a process that also has been observed experimentally.^[18]^ Two of the three amide ligands in LiAlH[N]_3_ bridge Li and Al and the third is bound only to Al in a terminal position. Interestingly, in this case the lowest barrier for reaction with H_2_ was found for hydrogenolysis of a bridging amide (C2→C3*: +28.6 kcal mol^−1^; that for hydrogenolysis of the terminal amide is slightly higher: +29.2 kcal mol^−1^). This last step is clearly the bottle‐neck in the catalytic reaction.

The high barrier for this last rate‐limiting step explains the high temperature of 85 °C needed for catalysis. The high temperature could also induce side‐reactions, like the experimentally observed *ortho*‐metallation (vide supra). Starting from **B3** we located two transition states for *ortho*‐alumination. The lowest barrier is found for deprotonation of the terminal amide by the bridging amide (B3→D1*: +33.0 kcal mol^−1^). A much higher barrier was found for *ortho*‐alumination by Al−H (+48.4 kcal mol^−1^). Although *ortho*‐deprotonation could be feasible at high temperatures, the insertion of an imine is still the preferred reaction (B3→C3*: 23.2 kcal mol^−1^).

**Scheme 3:**
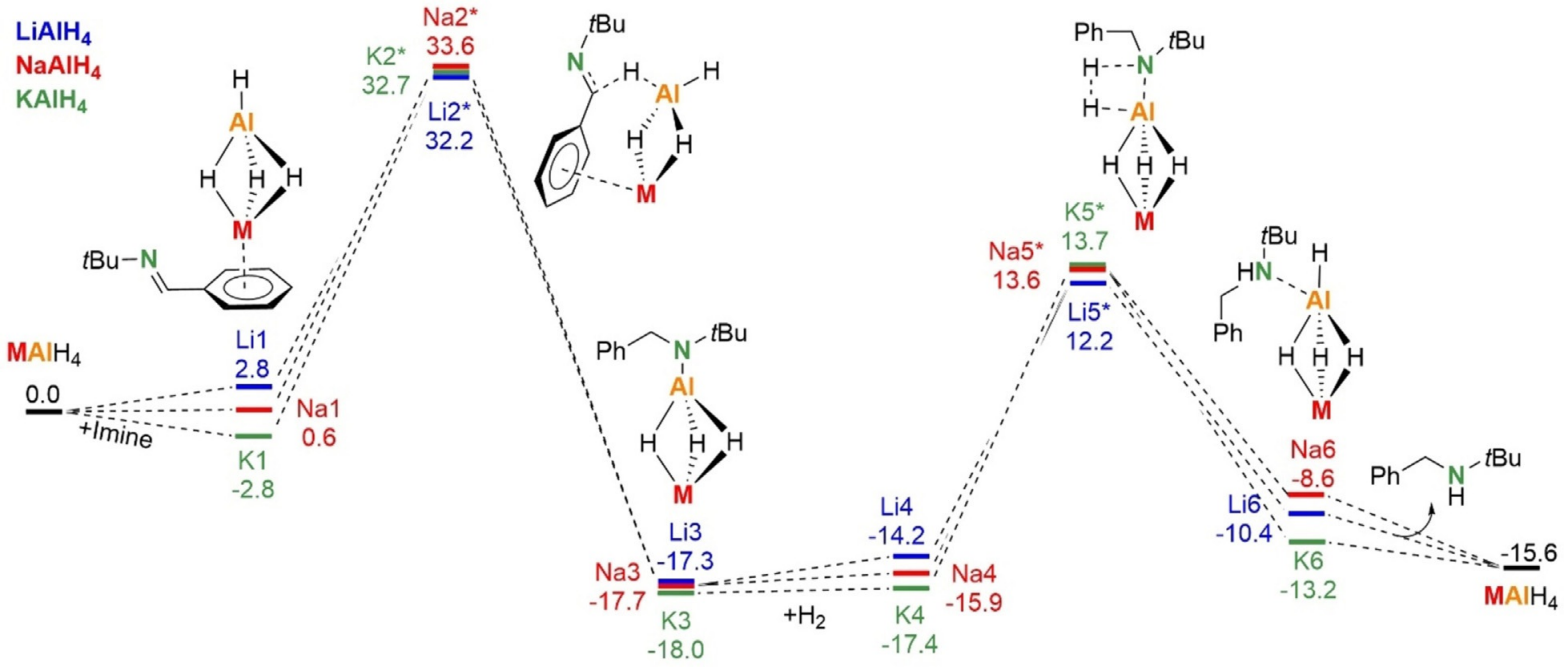
Comparison of the metal influence on hydrogenation of imines with MAlH_4_ catalysts (M=Li, Na, K); B3PW91/6–311++G**(PCM=THF)//6–31+G**; Δ*H* in kcal mol^−1^.

This comprehensive calculation study shows that LiAlH2[N]2 (B3) is the most likely catalyst. The role of Li+ in this heterobimetallic catalyst is coordination and activation of the imine substrate. The influence of alkali metal size was studied by calculating energy profiles for the series of catalysts MAlH_4_ (M=Li, Na, K). Since we only aim to compare the different metal catalysts among each other, we simplified the model system to the “naked” catalysts and omitted solvation by additional imine ligands and only included corrections for solvation by the PCM model for THF. As solvation effects can be large for *s*‐block metal complexes, absolute energy values should be treated with caution. Lack of solvation leads to increased energy barriers, but the energy profiles clearly show trends and the effect of metal exchange. In all cases a characteristic Ph⋅⋅⋅M interaction was found in the transition states (Li2*, Na2* and K2*; Scheme 3). The relative energies for these transition states differ only by 1–2 kcal mol^−1^. Since the preliminary MAlH_4_⋅(imine) complexes **Li1**, **Na1** and **K1** become more stable with alkali metal size, the energy barriers increase in the order: Li (+29.4 kcal mol^−1^)<Na (+33.0 kcal mol^−1^)<K (+35.5 kcal mol^−1^). The barrier for the subsequent hydrogenolysis step also increase with metal size: Li (+26.4 kcal mol^−1^)<Na (+29.5 kcal mol^−1^)<K (+31.1 kcal mol^−1^). The calculated order for these energy barriers (Li<Na<K) is in line with the experimental observation that LiAlH_4_ is the most active catalyst.

**Scheme 4:**
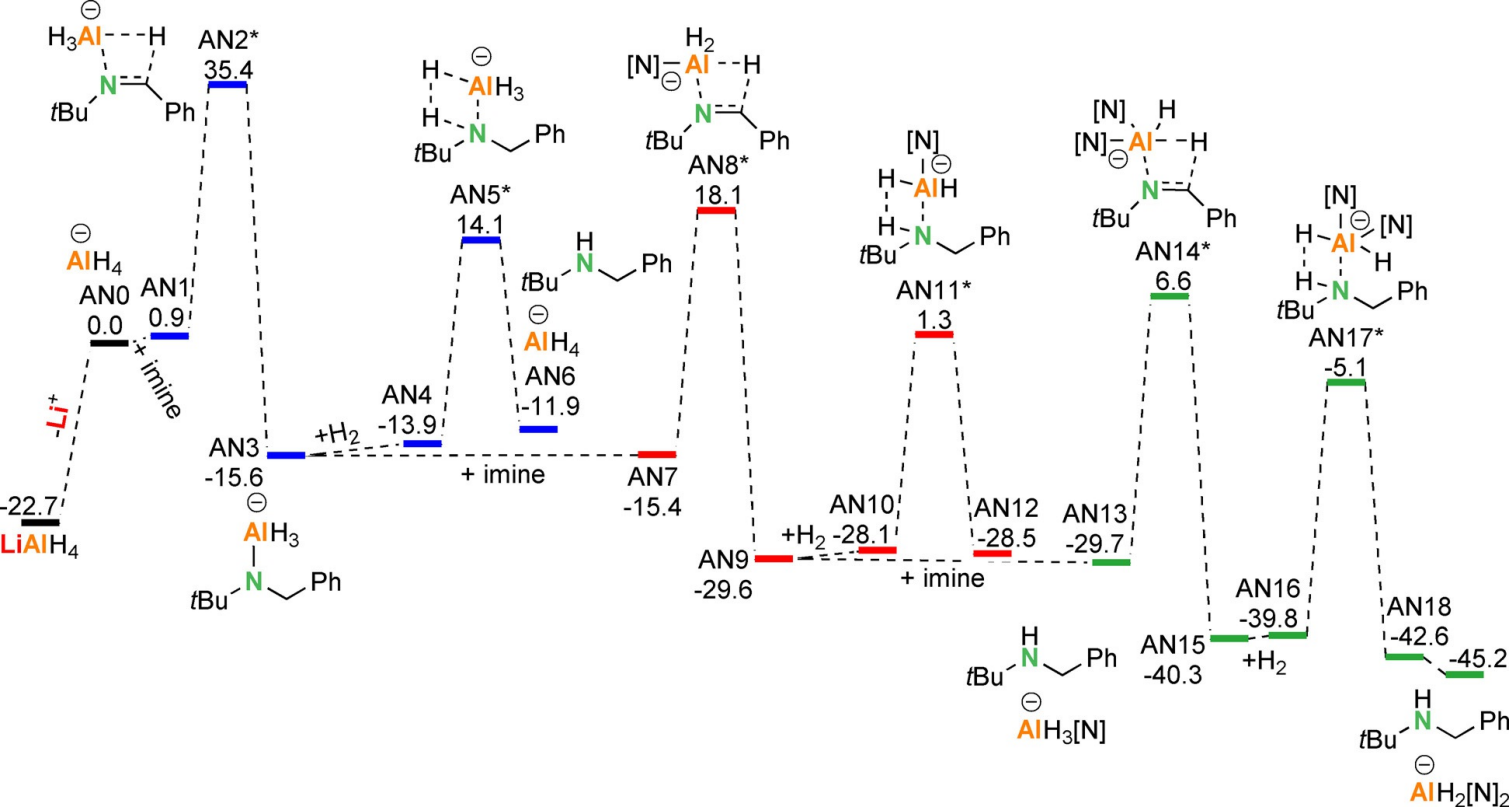
Full energy profile for the hydrogenation of PhC(H)=N*t*Bu with the anion AlH_4_^−^ (B3PW91/6–311++G**(PCM=THF)//6–31+G**); Δ*H* in kcal mol^−1^.

These calculations clearly demonstrate the crucial influence of the alkali metal. It can, however, not be excluded that LiAlH_4_ dissolved in neat imine or in a polar solvent like THF forms a solvent‐separated‐ion‐pair, [Li^+^(solvent)_*n*_][AlH_4_^−^], in which AlH_4_^−^ is the true catalyst. The electrostatic energy needed to separate LiAlH_4_ in Li^+^ and AlH_4_^−^ ions was calculated to be only +22.7 kcal mol^−1^, a value that could be partially compensated for by solvation. Indeed, solvation of Li^+^ with four equivalents of THF is exothermic by Δ*H*=−23.7 kcal mol^−1^, indicating that an equilibrium between contact‐ion‐pair and solvent‐separated‐ion‐pair is feasible. However, the energy profile for imine hydrogenation with only the AlH_4_^−^ anion (Scheme 4) is clearly not competitive with that calculated for LiAlH_4_ (Scheme 2). Especially the imine insertion barriers, which range from +33.5 to +36.3 kcal mol^−1^, are affected by loss of the alkali metal cation and are much higher than barriers calculated for the contact‐ion‐pair LiAlH_4_, ranging from +17.2 kcal mol^−1^ to +23.2 kcal mol^−1^. This is obviously related to the fact that both, Al and Li, play a role in the imine insertion step. The hydrogenolysis step, in which only the Al center plays a role, is less affected by ion separation. Considering the high barriers along the pathway, it is unlikely that the AlH_4_^−^ anion alone can be the catalyst. This is in agreement with the observation that [*n*Bu_4_N^+^][AlH_4_^−^] is not active in imine hydrogenation.^[16]^

**Scheme 5:**
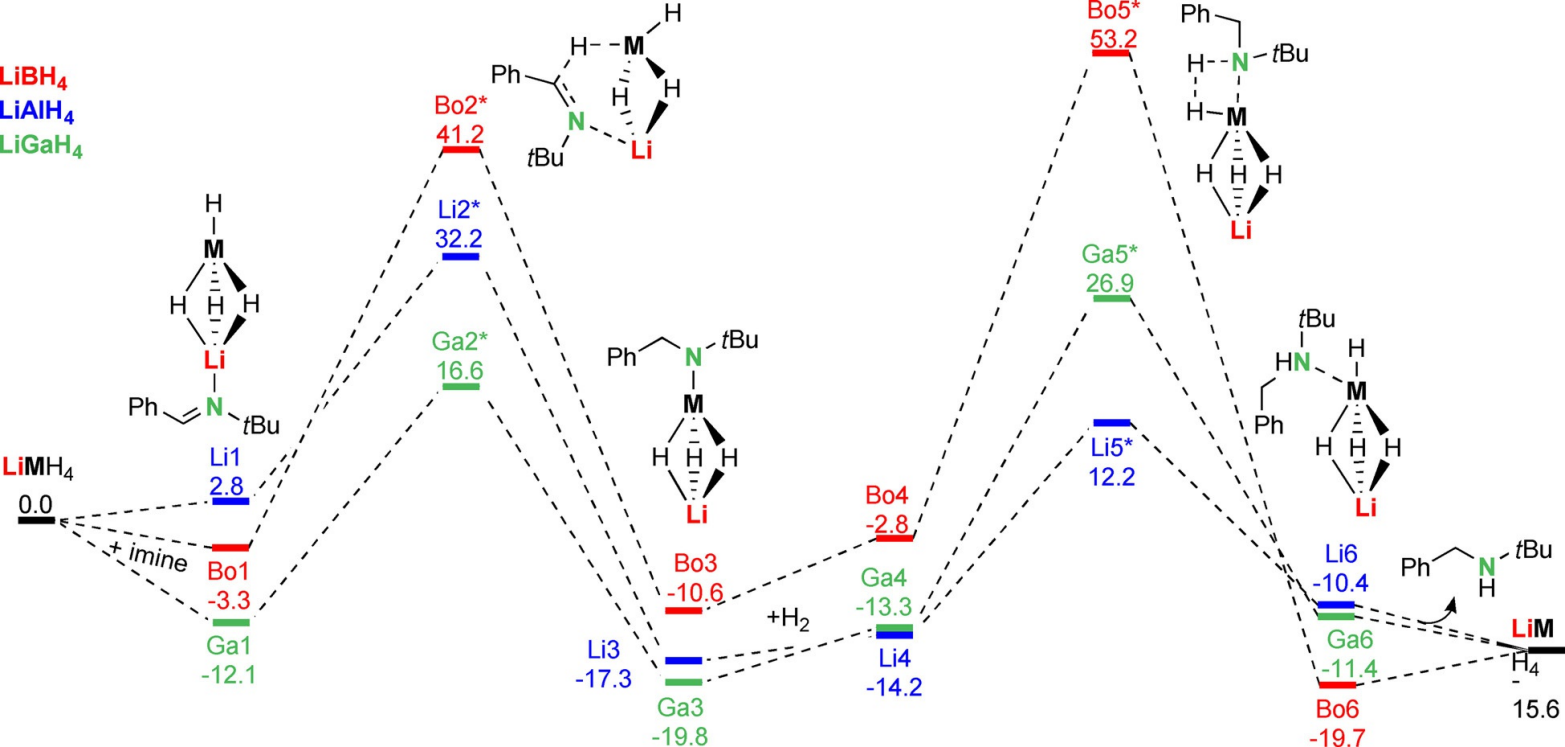
Comparison of the (half)metal influence on hydrogenation of imines with LiMH_4_ catalysts (M=B, Al, Ga); B3PW91/6–311++G**(PCM=THF)//6–31+G**; ΔH in kcal mol^−1^.

The influence of the *p*‐block metal in LiAlH_4_ was evaluated by comparing energy profiles for the catalysts LiMH_4_ (M=B, Al, Ga); Scheme 5. Imine insertion with the borate LiBH_4_ (Bo1→Bo2*) has a very high energy barrier of +44.5 kcal mol^−1^, which is in agreement with the fact that imines cannot be reduced by borates. The barrier for LiGaH_4_ (Ga1→Ga2*: +28.7 kcal mol^−1^) is slightly lower than that for LiAlH_4_ (+29.4 kcal mol^−1^). The second hydrogenolysis step, however, has a much higher energy barrier for LiGaH_4_ (Ga4→Ga5*: +40.2 kcal mol^−1^) than for LiAlH_4_ (+26.4 kcal mol^−1^). This explains why for gallanate catalysts only sub‐stoichiometric conversion was observed in catalytic imine hydrogenation (Table 2, entries 5 and 6). Catalyst activity therefore decreases in order LiAlH_4_>LiGaH_4_>LiBH_4_, that is, with decreasing bond polarity: Al−H>Ga−H>B−H.

**Scheme 6:**
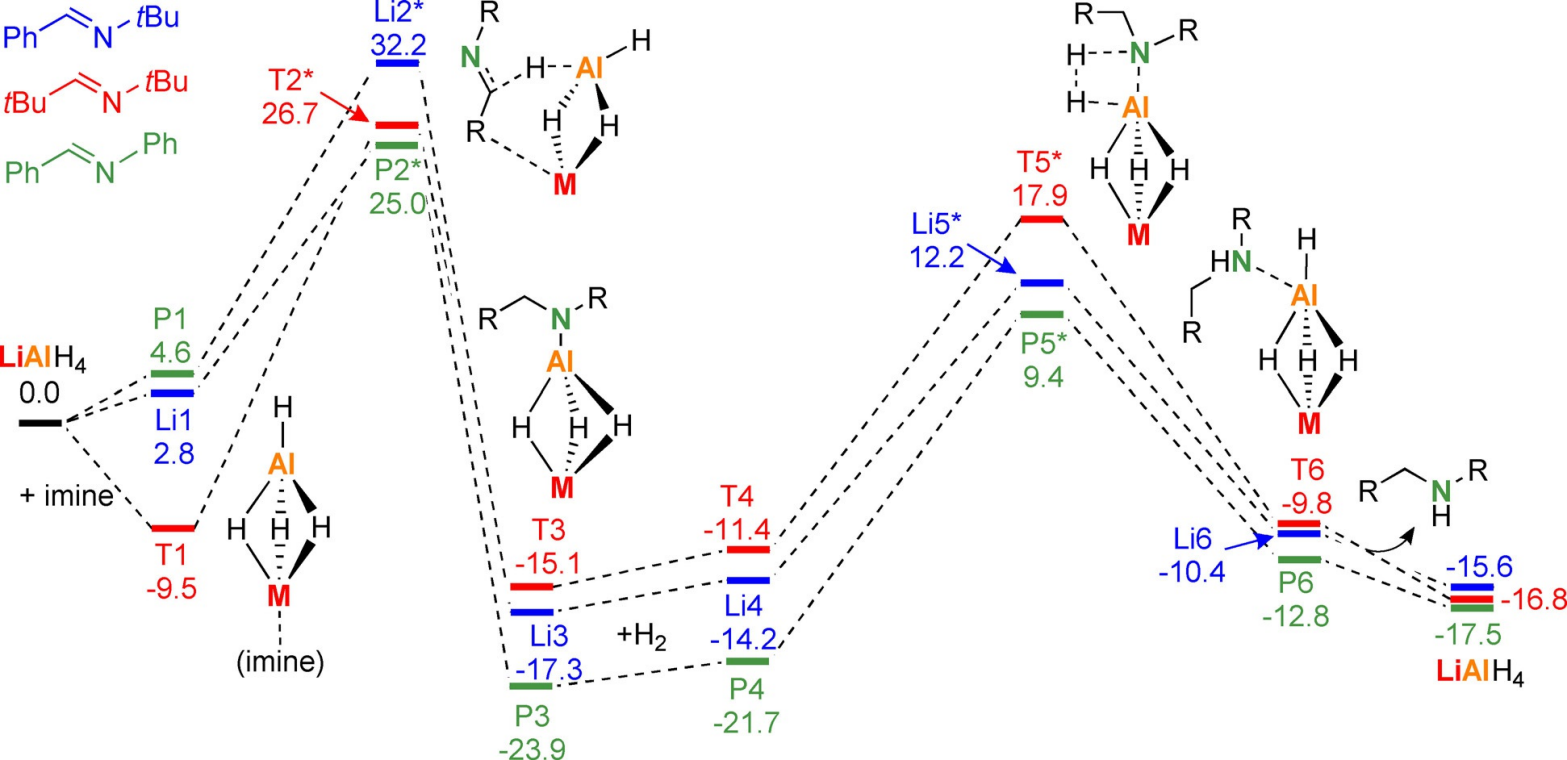
Comparison of imine substituent effects in hydrogenation with a LiAlH_4_ catalyst; B3PW91/6–311++G**(PCM=THF)//6–31+G**; Δ*H* in kcal mol^−1^.

The effect of different imine substitution was investigated by comparing energy profiles for PhC(H)=N*t*Bu, *t*BuC(H)=N*t*Bu and PhC(H)=NPh using a LiAlH_4_ catalyst. To reduce computation time, we omitted solvation by additional imine ligands (but included PCM corrections in THF) and only calculated the first imine insertion and hydrogenolysis steps (Scheme 6). The nature of the imine substituents (phenyl or alkyl) have an enormous effect on the barriers for imine insertion. The C=N double bond in PhC(H)=NPh is conjugated with two Ph rings and highly activated for insertion which is in agreement with a very low barrier (P1→P2*: +20.4 kcal mol^−1^). The highest barrier is found for the non‐conjugated C=N double bond in *t*BuC(H)=N*t*Bu (T1→T2*: +36.2 kcal mol^−1^) while that for PhC(H)=N*t*Bu is intermediate (+29.4 kcal mol^−1^). The very facile imine insertion of the highly activated C=N bond in PhC(H)=NPh is underscored by the experimental observation that LiAlH_4_ can insert four of these imines (vide supra) while for PhC(H)=N*t*Bu a maximum of three insertions can be achieved. For the least activated C=N double bond in *t*BuC(H)=N*t*Bu only two insertions are feasible (this is supported by DFT calculation; see Scheme S2). Also the stability of the products (**T3**, **P3** and **Li3**) is strongly affected by the substituents. Charge delocalization is possible for the amide anion PhCH_2_(Ph)N^−^ in **P3** but not for RCH_2_(*t*Bu)N^−^ in **Li3** and **T3**. Although this form of stabilization is advantageous for the imine insertion step, it is a disadvantage for the subsequent hydrogenolysis reaction. The highest barrier is found for reaction of resonance‐stabilized PhCH_2_(Ph)N^−^ with H_2_ (P4→P5*: +31.1 kcal mol^−1^). Amides with a *t*Bu substituent at N (RCH_2_(*t*Bu)N^−^) are much more reactive which is underscored by lower barriers for their reaction with H_2_ (Li4→Li5*: +26.4 kcal mol^−1^; T4→T5*: +29.3 kcal mol^−1^). For this reason, the best combination of substituents is a Ph group at C, to give facile imine hydrogenation, and a *t*Bu group at N, to give facile hydrogenolysis.

## Conclusions

Exchanging the alkali metal in LiAlH_4_ for heavier group 1 metals was found to be detrimental for its catalytic activity in imine hydrogenation. The following order of catalyst activity was observed: LiAlH_4_>NaAlH_4_>KAlH_4_. This implies that the *s*‐block metal should be relatively Lewis acidic. Indeed, the *s*‐block metal‐free catalyst [*n*Bu_4_N^+^][AlH_4_^−^] is inactive. Exchanging the *p*‐block metal in LiAlH_4_ for either B or Ga also led to loss of activity. The nature of both, the *s*‐ and *p*‐block metal, is essential for catalysis. This is a clear indication for a heterobimetallic mechanism in which synergy between both metals is key to success.

Reaction of LiAlH_4_ with one equivalent of PhC(H)=N*t*Bu gave a mixture of single and double addition products which implies that the second addition is faster than the first. Addition of a third imine is difficult but can be achieved with an excess of imine and longer reaction times. Addition of the fourth imine could only be observed for highly activated imines like PhC(H)=NPh. Several of these intermediates could be isolated and have been structurally characterized.

Reaction of LiAlH_4_ with excess PhC(H)=N*t*Bu led under forced conditions (85 °C, several days) to double imine insertion and *ortho*‐metallation in the Ph ring. The product does not react with H_2_ and this reaction could therefore be a catalyst deactivation pathway. However, DFT calculations demonstrate that the transition state for *ortho*‐metallation is much higher than that for imine insertion which means that this side‐reaction only plays a role at the end of the reaction when imine concentrations are low. Sequential double imine insertion/double *ortho*‐metallation may, however, be an attractive reactivity that could be exploited in synthesis.

Calculations are complicated by many variables (two different metal cations, two different anions), the high dynamics of these polar molecules and the importance of solvation. Treating solvation only with the PCM method is insufficient and additional coordination of imine to Li is needed to reduce the barriers.

The catalyst LiAlH_4_ can insert several equivalents of PhC(H)=N*t*Bu and form intermediates like LiAlH_3_[N], LiAlH_2_[N]_2_ or LiAlH[N]_3_; [N]=PhCH_2_(*t*Bu)N. Calculations show that the lowest energy barriers are found for a catalytic cycle based on LiAlH_2_[N]_2_. The cycle consists of two steps: (1) imine insertion gives LiAlH[N]_3_ with a barrier of +23.2 kcal mol^−1^ (Δ*H*) and (2) the rate‐determining step: hydrogenolysis of LiAlH[N]_3_ with H_2_ to give LiAlH_2_[N]_2_ and [N]−H with a barrier of +28.6 kcal mol^−1^.

Both metals, Li and Al, actively participate in the transition state for imine insertion: Li activates the imine by coordination and Al delivers the hydride for reduction. In the hydrogenolysis step only the Al center is involved. A catalytic cycle based only on AlH_4_^−^ shows very high barriers and is not feasible.

Calculation of the energy profiles for the catalysts MAlH_4_ (M=Li, Na, K) confirm the experimental observation that the activities decrease along the row Li>Na>K, that is, with decreasing Lewis acidity. On a similar note and in agreement with experiment, calculations of the energy profiles for catalysts LiMH_4_ (M=B, Al, Ga) show an activity order Al>Ga>B, that is, with decreasing polarity of the M−H bond.

Calculation of the energy profiles for the LiAlH_4_ catalyzed hydrogenation of PhC(H)=N*t*Bu, *t*BuC(H)=N*t*Bu and PhC(H)=NPh demonstrates the influences of the C‐ and *N*‐substituents on the energy barriers for insertion and hydrogenolysis. While Ph‐substituents activate the C=N double bond for insertion, the resulting resonance‐stabilized amide PhCH_2_(Ph)N^−^ is much less reactive in the subsequent hydrogenolysis step than PhCH_2_(*t*Bu)N^−^. The most favorable combination is therefore found in the imine substrate PhC(H)=N*t*Bu which is often used as the benchmark imine.

## Experimental Section

All experiments were carried out using standard Schlenk techniques or a glovebox (MBraun, Labmaster SP) and freshly dried solvents. THF (THF AnalaR Normapur, VWR) was dried over molecular sieves (3 Å) and distilled from sodium. All other solvents were degassed with nitrogen, dried over activated aluminum oxide (Innovative Technology, Pure Solv 400‐4‐MD, Solvent Purification System) and stored over molecular sieves (3 Å) under inert atmosphere. LiAlH_4_ (Sigma–Aldrich, 95 %) was purified by extraction with diethyl ether and dried under reduced pressure. NaAlH_4_ (Sigma–Aldrich, 90 %) was purified by extraction with THF and dried under reduced pressure. 18‐Crown‐6 (TCI, 98 %) was dissolved in diethyl ether, stirred over CaH_2_, filtered, and dried under reduced pressure. Starting materials were used as delivered unless noted otherwise. Imine PhC(H)=N*t*Bu was purchased from Sigma–Aldrich, stirred over CaH_2_, and distilled prior to use. Imine *t*BuC(H)=N*t*Bu was prepared according to Momiyama et al.^[31]^ and stirred over CaH_2_ and distilled prior to use and imine PhC(H)=NPh was prepared according to Cattoën et al.^[32]^ and dissolved in pentane, stirred over CaH_2_, filtered, and dried under reduced pressure.

NMR spectra were measured on Bruker Avance III HD 400 MHz and Bruker Avance III HD 600 MHz spectrometers. Elemental analysis was performed with an Hekatech Eurovector EA3000 analyzer. Crystal structures have been measured on a SuperNova (Agilent) diffractometer with dual Cu and Mo microfocus sources and an Atlas S2 detector.

Deposition Numbers 2024356 (for **1**), 2024357 (for **2**), 2024358 (for **3**), 2024359 (for **4**), 2024360 (for **5**), 2024361 (for **6**), 2024362 (for **7**) and 2024363 (for **8**) contain the supplementary crystallographic data for this paper. These data are provided free of charge by the joint Cambridge Crystallographic Data Centre and Fachinformationszentrum Karlsruhe Access Structures service www.ccdc.cam. ac.uk/structures. 

**Synthesis of LiAlH[N(*t*Bu)CH_2_Ph]_3_⋅(THF)_4_ (1)**: LiAlH_4_ (75.9 mg, 2.00 mmol) and PhC(H)=N*t*Bu (1.81 g, 2.00 mL, 11.2 mmol) were dissolved in THF (2 mL) and stirred overnight at room temperature. The solution was concentrated under reduced pressure to afford a yellow oil from which crystals formed. The crude product was washed with cold pentane (−20 °C 3×1 mL). The residual oil was layered with pentane (1 mL) to afford a second crop of crystals. The title compound was received as white crystals. (681 mg, 0.840 mmol, 42 %). Elemental analysis: Calcd. for C_41_H_65_N_3_O_2_AlLi: N=6.31 %, C=73.95 %, H=9.84 %. Found N=6.52 %, C=73.76 %, H=10.09 %. ^1^H NMR (600 MHz, [D_8_]THF) *δ* 7.44 (6 H, m, Ph), 7.05 (6 H, m, Ph), 6.92 (3 H, m, Ph), 4.19, (6 H, s, CH_2_), 3.65 (br., Al‐H), 3.62 and 3.58 (THF/[D_8_]THF), 1.77 and 1.73 (THF/[D_8_]THF), 1.10 (27 H, s, *t*Bu). ^13^C NMR (101 MHz, [D_8_]THF) *δ* 152.5 (Ph), 127.4 (Ph), 126.5 (Ph), 123.8 (Ph), 67.2 and 66.4 (THF), 53.2 (Ph*C*H_2_N), 51.8 (*C*Me_3_), 31.6 (C*Me_3_*), 25.4 and 24.3 (THF). ^7^Li NMR (156 MHz, [D_8_]THF) δ−0.38. ^27^Al NMR (156 MHz, [D_8_]THF) *δ* 115.2.

**Synthesis of NaAlH_2_[N(*t*Bu)CH_2_Ph]_2_⋅(TMEDA)_2_ (2)**: NaAlH_4_ (27.0 mg; 0.500 mmol), PhC(H)=N*t*Bu imine (161 mg; 178 μL; 1.00 mmol) and TMEDA (116 mg; 151 μL; 1.00 mmol) were placed in 1 mL of hexane and allowed to stand for 4 hours at 80 °C. After allowing to cool to room temperature off‐white crystals formed. The mother liquor was removed, the crystals were washed with cold (−20 °C) pentane (3×1 mL) and dried under reduced pressure. The mother liquor was allowed to stand at −20 °C for 1 hour were a second crop of crystals formed, which were isolated in similar fashion. The product was obtained as off‐white crystals (231 mg; 0.380 mmol; 76 %). Elemental Analysis: Calcd. for C_34_H_66_N_6_AlNa: N=13.80 %; C=67.07 %; H=10.93 %; Found: N=13.63 %; C=67.42 %; H=10.65 %. ^1^H NMR (600 MHz, [D_8_]THF) *δ* 7.43 (4 H, m, Ph), 7.07 (4 H, m, Ph), 6.94 (2 H, m, Ph), 4.50–3.25 (br., AlH_2_), 4.21 (4 H, s, CH_2_), 2.30 (8 H, s, CH_2_), 2.15 (24 H, s, CH_3_), 1.11 (18 H, s, *t*Bu). ^13^C NMR (101 MHz, [D_8_]THF) *δ* 152.3 (Ph), 127.1 (Ph), 126.7 (Ph), 123.9 (Ph), 57.9 (TMEDA *C*H_2_), 53.1 (Ph*C*H_2_N), 51.8 (*C*Me_3_), 45.2 (TMEDA *Me*), 31.6 (C*Me_3_*). ^23^Na NMR (159 MHz, [D_8_]THF) δ−2.09. ^27^Al NMR (156 MHz, [D_8_]THF) *δ* 115.5.

**Synthesis of NaAlH_2_[N(*t*Bu)CH_2_Ph]_2_⋅PMDTA (3)**: NaAlH_4_ (27.0 mg; 0.500 mmol), PhC(H)=N*t*Bu imine (161 mg; 178 μL; 1.00 mmol) and PMDTA (86.7 mg; 105 μL; 0.500 mmol) were placed in 1 mL of hexane and allowed to stand for 4 hours at 80 °C. After allowing to cool to room temperature off‐white crystals formed. The mother liquor was removed, the crystals were washed with cold (−20 °C) pentane (3*1 mL) and dried under reduced pressure. The mother liquor was allowed to stand at −20 °C for 1 hour were a second crop of crystals formed, which were isolated in similar fashion. The product was obtained as off‐white crystals (177 mg; 0.322 mmol; 64 %). Elemental Analysis: Calcd. for C_31_H_57_N_5_AlNa : N=12.74 %; C=67.72 %; H=10.45 %; Found: N=12.77 %; C=67.71 %; H=10.52 %. ^1^H NMR (600 MHz, [D_8_]THF) *δ* 7.43 (4 H, m, Ph), 7.07 (4 H, m, Ph), 6.94 (2 H, m, Ph), 4.50–3.00 (br., AlH_2_), 4.21 (4 H, s, CH_2_), 2.42 (4 H, t, 6.4 Hz, CH_2_), 2.31 (4 H, t, 6.4 Hz, CH_2_), 2.20 (3 H, s, CH_3_), 2.15 (12 H, s, CH_3_), 1.11 (18 H, s, *t*Bu). ^13^C NMR (151 MHz, [D_8_]THF) *δ* 152.3 (Ph), 127.1 (Ph), 126.7 (Ph), 123.9 (Ph), 57.9 (PMDTA), 56.4 (PMDTA), 53.1 (Ph*C*H_2_N), 51.8 (*C*Me_3_), 45.2 (PMDTA), 42.4 (PMDTA), 31.6 (C*Me_3_*). ^23^Na NMR (159 MHz, [D_8_]THF) δ−1.89. ^27^Al NMR (156 MHz, [D_8_]THF) *δ* 115.4.

Synthesis of KAlH_2_[N(*t*Bu)CH_2_Ph]_2_⋅18‐crown‐6 (4): KH (20.1 mg; 0.500 mmol), 18‐crown‐6 (127.2 mg, 0.500 mmol), AlH_3_(THF)_2_ (87.1 mg, 0.500 mmol) were placed in 5 mL THF. PhC(H)=N*t*Bu imine (161 mg, 178 μL, 1.00 mmol) were added and the mixture was stirred at 85 °C overnight. After allowing the mixture to cool to room temperature, the solvent was removed to approximately have its volume under reduced pressure. The solution was layered with hexane and allowed to stand at room temperature for two days. Colorless crystals of the target compound were obtained and washed with 1 mL cold (−20 °C pentane) and dried in vacuo (84.0 mg; 0.128 mmol; 26 %). Elemental Analysis: Calcd. for C_34_H_58_N_2_O_6_AlK : N=4.26 %; C=62.16 %; H=8.90 %; Found: N=4.09 %; C=61.90 %; H=9.07 %. ^1^H NMR (600 MHz, [D_8_]THF) *δ* 7.50 (4 H, m, Ph), 7.02 (4 H, m, Ph), 6.88 (2 H, m, Ph), 4.52 (br., AlH_2_), 4.22 (4 H, s, CH_2_), 3.56 (24 H, s, 18‐crown‐6), 1.09 (18 H, s, *t*Bu). ^13^C NMR (151 MHz, [D_8_]THF) *δ* 153.8 (Ph), 127.7 (Ph), 126.3 (Ph), 123.4 (Ph), 70.2 (18‐crown‐6), 53.3 (Ph*C*H_2_N), 52.4 (*C*Me_3_), 31.9 (C*Me_3_*). ^27^Al NMR (156 MHz, [D_8_]THF) *δ* 119.8.

Synthesis of LiAlH_2_[N(*t*Bu)CH_2_ *t*Bu]_2_⋅(TMEDA) (5): LiAlH_4_ (19.0 mg, 0.500 mmol), *t*BuC(H)=N*t*Bu (212 mg, 268 μL, 1.50 mmol) and TMEDA (116 mg, 151 μL, 1.00 mmol) were placed in an NMR tube. Hexane (400 μL) was added and the tube was allowed to stand at 80 °C for two days. All volatiles were removed under reduced pressure. The crude product was recrystallized form cold (−20 °C) pentane, giving the target compound as colorless crystals (86.1 mg, 0.197 mmol, 39 %). Elemental analysis: Calcd. for C_24_H_58_N_4_AlLi : N=12.83 %; C=66.01 %; H=12.39 %; Found: N=12.82 %; C=65.99 %; H=13.38 %. ^1^H NMR (600 MHz, C_6_D_6_) *δ* 3.79 (2 H, br, AlH_2_), 3.03 (4 H, s, CH_2_), 1.84 (12 H, s, CH_3_), 1.62 (18 H, s, *t*Bu), 1.56 (4 H, s, CH_2_), 1.25 (18 H, s, *t*Bu). ^7^Li NMR (156 MHz, C_6_D_6_) δ−0.68. ^13^C NMR (151 MHz, C_6_D_6_) *δ* 58.7 (TMEDA *C*H_2_), 55.9 (CH_2_), 53.2 (*C*Me_3_), 45.1 (TMEDA *Me*), 33.4 (*C*Me_3_), 32.8 (C*Me_3_*), 30.5 (C*Me_3_*). ^27^Al NMR (104 MHz, C_6_D_6_) *δ* 117.5.

Synthesis of LiAl[N(Ph)CH_2_Ph]_4_⋅(THF)_4_ (6): LiAlH_4_ (19.0 mg; 0.500 mmol) and PhC(H)=NPh (544 mg; 3.00 mmol) were placed in 2 mL of hexane and stirred at 85 °C for three days. The solution was allowed to cool to room temperature. The solvent was removed under reduced pressure. The remaining solids were dissolved in 4 mL THF and layered with 4 mL hexane. After 5 days at room temperature the product was obtained as colorless crystals which were washed with cold (−20 °C) pentane and subsequently dried in vacuo (258 mg; 0.245 mmol; 49 %). Elemental analysis: Calcd. for C_68_H_80_N_4_O_4_AlLi : N=5.33 %; C=77.69 %; H=7.67 %; Found: N=5.30 %; C=77.71 %; H=7.61 %. ^1^H NMR (600 MHz, [D_8_]THF) *δ* 7.19 (8 H, m, Ph), 6.99 (8 H,m, Ph), 6.89 (4 H, m, Ph), 6.52 (8 H, m, Ph), 6.42 (8 H, m, Ph), 5.93 (4 H, m, Ph), 4.84 (8 H, s, br. CH_2_), 3.58 (m, THF, overlaps with [D_8_]THF), 1.74 (m, THF, overlaps with [D_8_]THF). ^7^Li NMR (156 MHz, [D_8_]THF) δ−0.61. ^13^C NMR (151 MHz, [D_8_]THF) *δ* 154.0 (Ph), 144.3 (Ph), 127.1 (Ph), 127.0 (Ph), 126.7 (Ph), 124.1 (Ph), 116.3 (Ph), 112.5 (Ph), 66.4 (THF), 50.8 (*C*H_2_), 24.3 (THF). ^27^Al NMR (156 MHz, [D_8_]THF) *δ* 103.5.

**Synthesis of 7**: LiAlH_4_ (19.0 mg; 0.500 mmol) and PhC(H)=N*t*Bu imine (484 mg; 534 μL; 3.00 mmol) were placed in 1 mL of hexane and allowed to stand for 3 days at 85 °C. After allowing to cool to room temperature, colorless crystals formed. The mother liquor was removed, and the crystals were washed with cold (−20 °C) pentane (3×1 mL) and dried under reduced pressure. The product was obtained as colorless crystals (62.2 mg; 0.130 mmol; 24 %). The product contains one additional coordinated PhC(H)=N*t*Bu ligand which in the crystal structure is disordered with the amine PhCH_2_(*t*Bu)NH. The latter amine is formed by deprotonation of a phenyl ring by an amide ligand. Elemental analysis: Calc (**7** with an imine/amine ratio of 1/1 C_66_H_92_N_6_Al_2_Li_2_): N=8.10 %; C=76.42 %; H=8.76 %; Found: N=8.26 %; C=77.08 %; H=9.12 %. ^1^H NMR (600 MHz, C_6_D_6_) *δ* 8.14 (1 H, s, PhC*H*=N), 7.78 (3 H, m, Ph), 7.33 (4 H, m, Ph) 7.20 (4 H, m, Ph), 7.17 (4 H, m, Ph), 7.11 (3 H, m, Ph), 4.71 (2 H, d,14.8 Hz, C*H*_2_), 4.62 (2 H, d,14.8 Hz, C*H*_2_), 3.60 (2 H, d, 7.6 Hz, C*H*_2_ Amine), 1.58 (18 H, s, C*Me_3_*), 1.22 (9 H, s, C*Me_3_* imine), 1.02 (9 H, s, C*Me_3_* amine), 0.66 (1 H, t (br) 7.6 Hz, NH amine). ^7^Li NMR (156 MHz, C_6_D_6_) δ−0.99. ^13^C NMR (151 MHz, C_6_D_6_) *δ* 154.9 (Ph*C*H=N), 154.1 (Ph), 142.2 (Ph), 137.5 (Ph), 135.4 (Ph), 133.3 (Ph), 129.9 (Ph), 128.2 (Ph), 128.1 (Ph), 128.0 (Ph), 126.4 (Ph), 124.3 (Ph), 123.6 (Ph), 123.3 (Ph), 56.8 (Ph*C*H_2_N), 53.0 (Ph*C*H_2_N), 51.6 (*C*Me_3_), 49.9 (*C*Me_3_), 47.0 (*C*Me_3_), 31.0 (C*Me_3_*), 29.3 (C*Me_3_*), 28.8 (C*Me_3_*). ^27^Al NMR (104 MHz, C_6_D_6_) *δ* 139.4.

**Synthesis of 8**: LiAlH_4_ (75.9 mg; 2.00 mmol) and PhC(H)=N*t*Bu imine (1.94 g; 2.14 mL; 12.0 mmol) were placed in 8 mL of hexane and allowed to stand for 3 days at 85 °C. After allowing to cool to room temperature, an off‐white precipitate formed, which was dissolved in 4 mL THF and layered with 3 mL hexane. After three days white crystals formed which were washed with cold (−20 °C) pentane (3×2 mL) and dried under reduced pressure. The product was obtained as colorless crystals (612 mg; 0.949 mmol; 48 %). Elemental analysis: Calcd. for C_38_H_62_N_2_O_4_AlLi : N=4.34 %; C=70.78 %; H=9.69 %; Found: N=4.52 %; C=70.48 %; H=9.77 %. ^1^H NMR (600 MHz, [D_8_]THF) *δ* 7.33 (2 H, d, 6.7 Hz, Ph), 6.86 (2 H, d, 7.5 Hz, Ph), 6.77 (2 H, t, 7.3 Hz, Ph), 6.69 (2 H, t, 6.9 Hz, Ph), 4.17 (4 H, m, CH_2_), 3.62–3.58(16 H, m, THF, not integrated due to exchange with THF‐D8), 1.78–1.73 (16 H, m, THF, not integrated due to exchange with THF‐D8), 1.12 (18 H, s, *t*Bu). ^13^C NMR (151 MHz, [D_8_]THF) *δ* 153.9 (Ph), 135.4 (Ph), 122.7 (Ph), 122.2 (Ph) 122.1 (Ph), 67.2 (THF), 66.6 ([D_8_]THF), 52.5 (*C*H_2_), 51.0 (*C*Me_3_), 30.5 (C*Me_3_*), 25.4 (THF), 24.3 ([D_8_]THF). ^7^Li NMR (156 MHz, [D_8_]THF) δ−2.48. ^27^Al NMR (104 MHz, [D_8_]THF) *δ* 132.8.

**DFT calculations**: All calculations were carried out using Gaussian 16A.^[33]^ All structures were fully optimized on a B3PW91/6–31+G** level of theory. Harmonic frequency calculations were carried out on the same level of theory to characterize the structures as minima (NIMAG=0) or transition states (NIMAG=1). For higher accuracy, single point energies were determined on B3PW91/6–311++G**//6–31+G** level of theory.[^34^, ^35^, ^36^, ^37^] Solvent effects were modeled via polarizable continuum model (PCM) simulating THF (*ϵ*=7.4257).^[38]^ Throughout the calculational study, Δ*H* values are given in kcal mol^−1^.

## Conflict of interest

The authors declare no conflict of interest.

## Supporting information

As a service to our authors and readers, this journal provides supporting information supplied by the authors. Such materials are peer reviewed and may be re‐organized for online delivery, but are not copy‐edited or typeset. Technical support issues arising from supporting information (other than missing files) should be addressed to the authors.

Supplementary
