# Supplementary material for: Insights into LiAlH4 Catalyzed Imine Hydrogenation
Source: Chemistry. 2020 Nov 26;27(1):401–11. doi: 10.1002/chem.202003862 (PMC7839755; doi:10.1002/chem.202003862)
Supplement: Supplementary file 1 — Supplementary [file CHEM-27-401-s001.pdf]

Chemistry–A European Journal

Supporting Information

Insights into $LiAlH_4$ Catalyzed Imine Hydrogenation

Holger Elsen, Jens Langer, Gerd Ballmann, Michael Wiesinger, and Sjoerd Harder*[a]

Table of contents

General……………………………………………………………………………………………S2
NMR-Spectra…………………………………………………………………………………..S3
Crystallographic Information………………………………………………………..S23
DFT Calculations……………………………………………………………………………..S34
References……………………………………………………………………………………..S59

General

All experiments were carried out in dry glassware under N_2 using standard Schlenk techniques and freshly dried and degassed solvents (all solvents were dried over a column except for THF which was dried over Na-benzophenone and redistilled). Commercially available $LiAlH_4$ was extracted with diethyl ether and after removal of all solvents dried in vacuo to give a white fluffy material. Commercially available $NaAlH_4$ was purified via a similar method using THF·$KAlH_4$·[18-crown-6/THF] was prepared according to Trzaska.[S1] Ph(H)C=N*t*Bu was obtained commercially from Sigma-Aldrich, 97 %. Following imine substrates were prepared according to literature procedures:

PhC(H)=NPh[S2]
*t*BuC(H)=N*t*Bu[S3]

Hydrogen of quality N5 has been used for catalytic hydrogenation. Deuterated solvents were dried over molsieves (3 Å) in a glovebox. All substrates were dried by stirring over freshly powdered CaH_2 at 60 °C. Reactors were dried by heating overnight in an oven at 70 °C. The products of catalysis have been analyzed by NMR. Conversions were determined by integration of characteristic signals.

NMR spectra were measured on Bruker Avance III HD 400 MHz and Bruker Avance III HD 600 MHz spectrometers. Elemental analysis was performed with an Hekatech Eurovector EA3000 analyzer. Crystal structures have been measured on a SuperNova (Agilent) diffractometer with dual Cu and Mo microfocus sources and an Atlas S2 detector. Crystallographic data have been deposited with the Cambridge Crystallographic Data Centre as supplementary publication 2024356-2024363.

NMR-Spectra

Reference Spectra

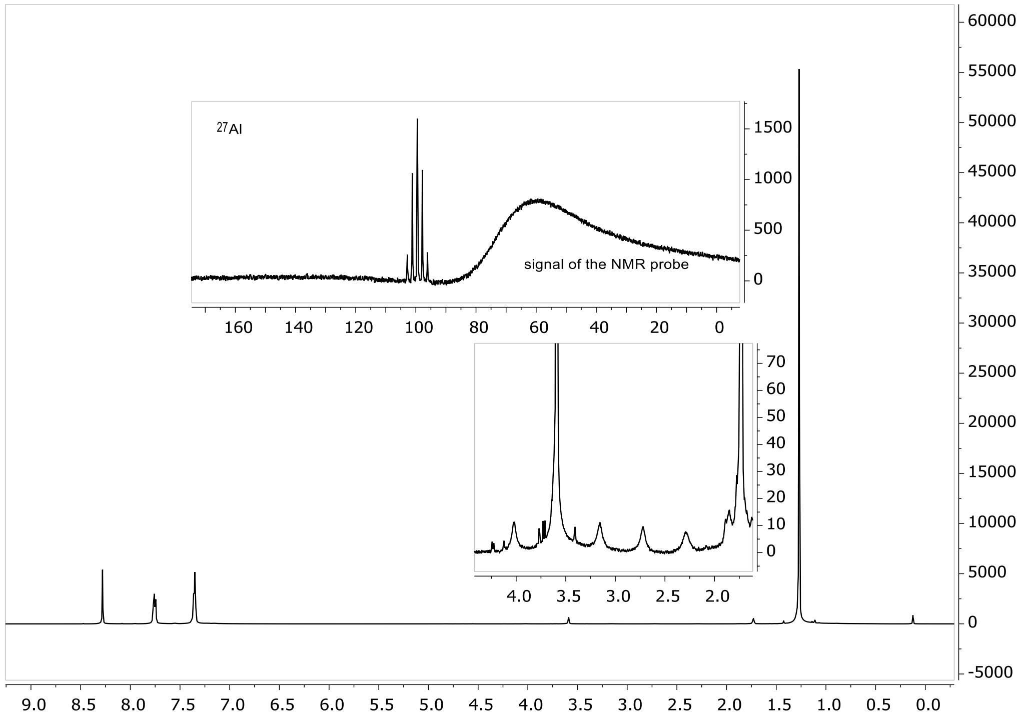

Figure S1: Reference spectra of $KAlH_4$ produced by the reaction of KH with AlH_3THF_2 in pure PhC(H)=N*t*Bu (no-D-NMR).

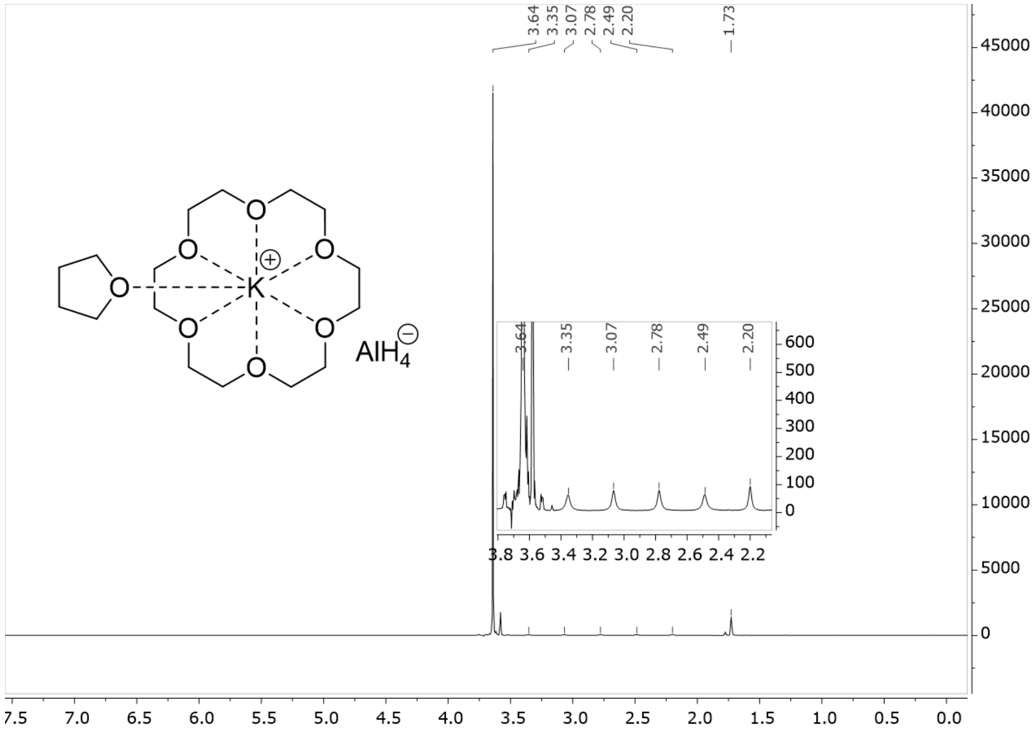

Figure S2: Reference ^{1}H-NMR of $KAlH_4$·[18-crown-6/THF] in THF-d_8.

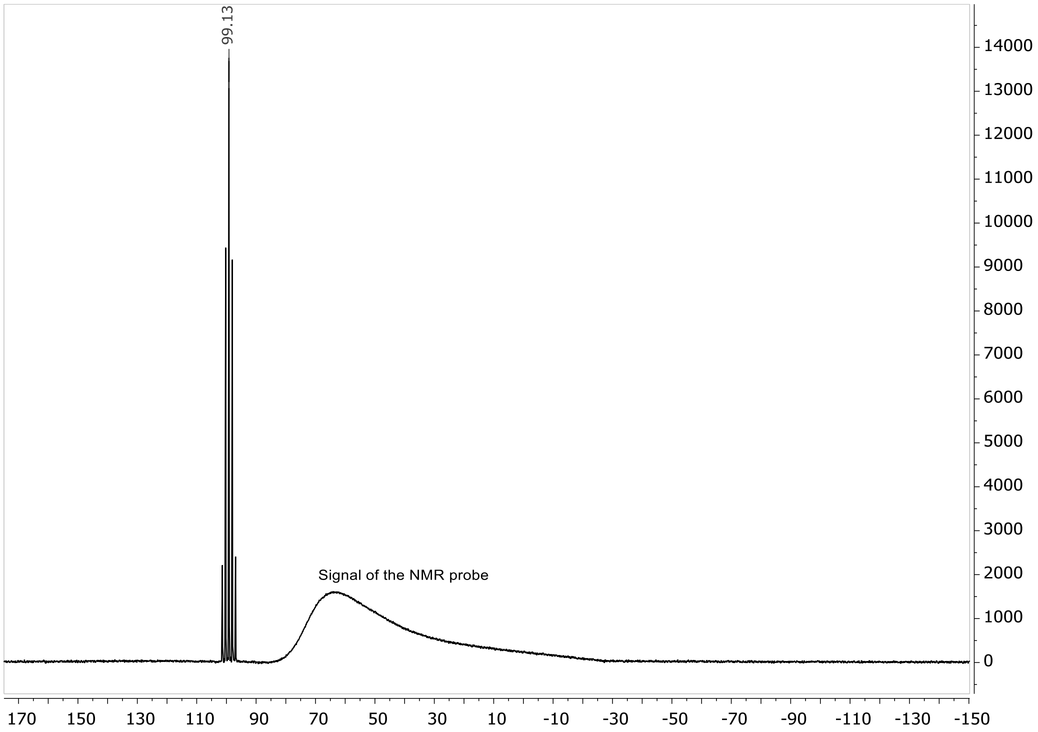

Figure S3: Reference ^{27}Al-NMR of $KAlH_4$·[18-crown-6/THF] in THF-d_8.

Catalytic reductions

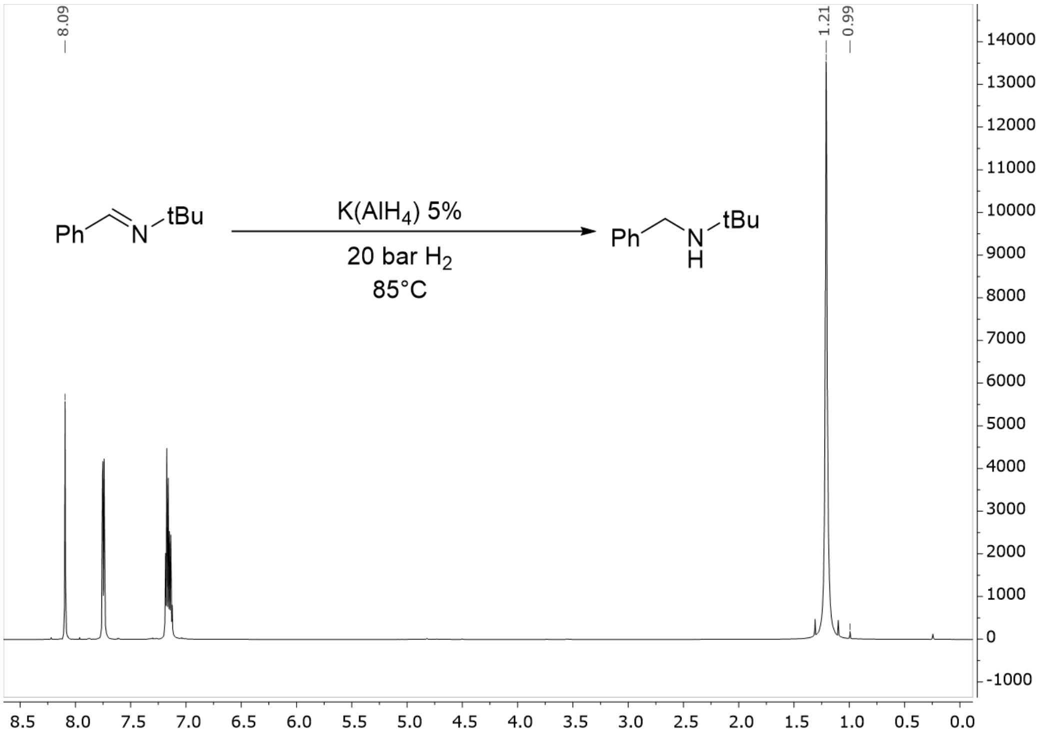

Figure S4: Catalytic reduction of PhC(H)=N*t*Bu using 5 mol% $KAlH_4$ at 20 bar H_2 and 85°C in neat imine after 24h. NMR in C_6D_6.

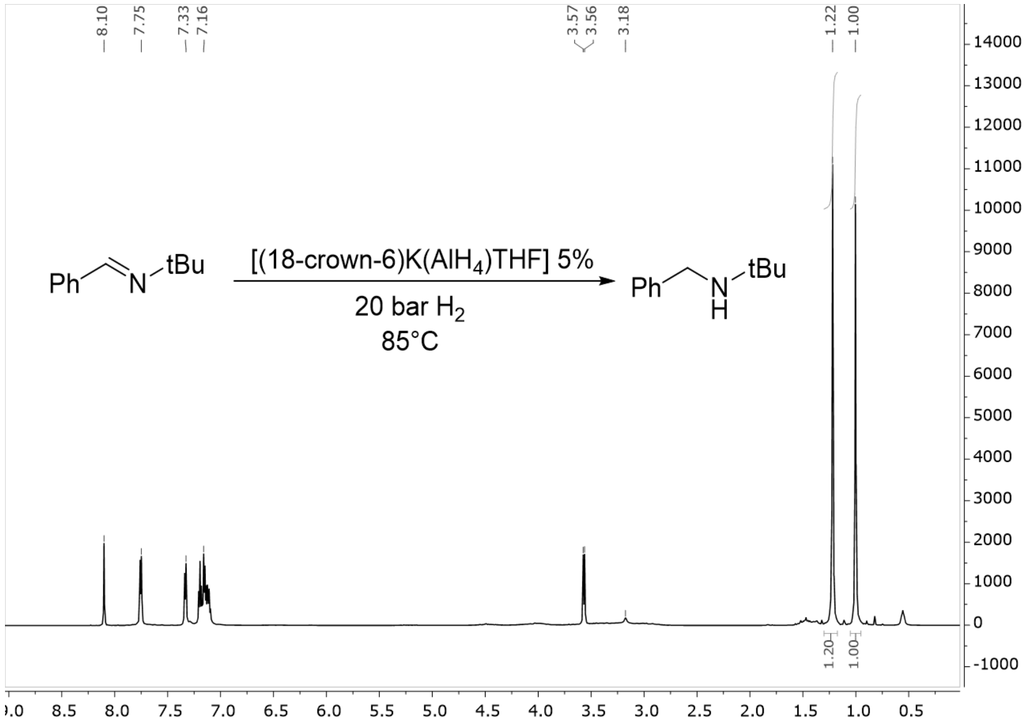

Figure S5: Catalytic reduction of PhC(H)=N*t*Bu using 5 mol% [(18-crown-6)K(AlH_4)THF] at 20 bar H_2 and 85°C in neat imine after 24h. NMR in C_6D_6.

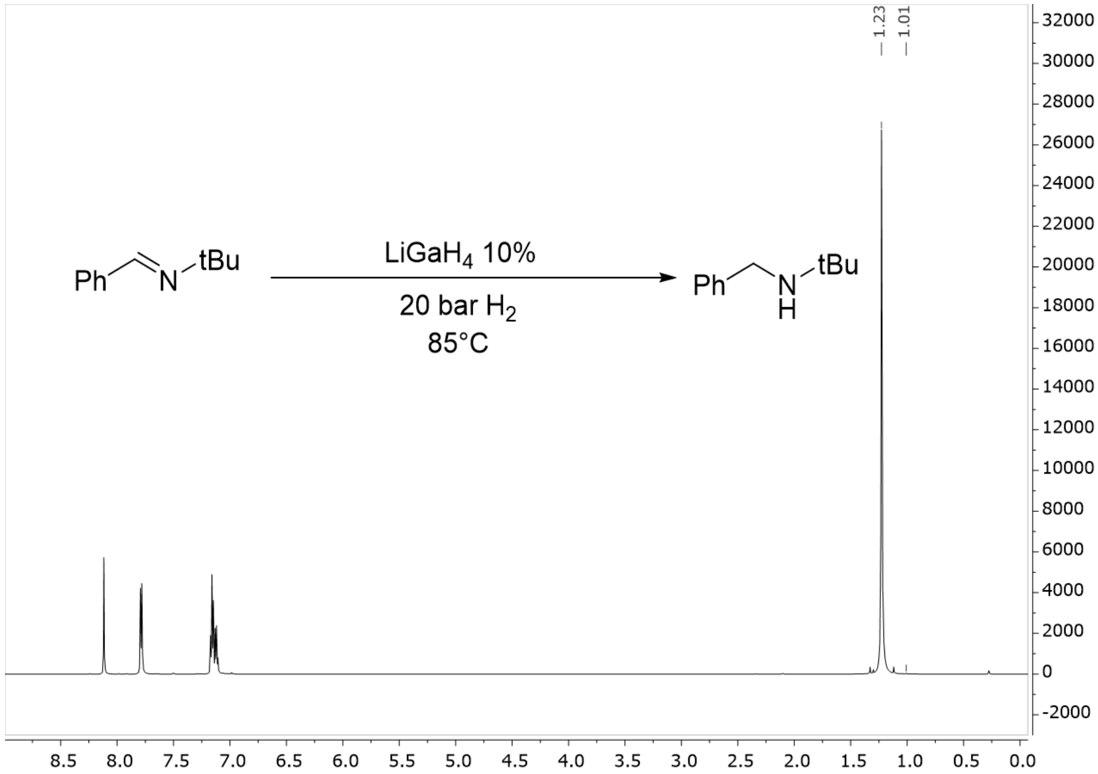

Figure S6: Catalytic reduction of PhC(H)=N*t*Bu using 10 mol% $LiGaH_4$·$(TMEDA)_2$ at 20 bar H_2 and 85°C in neat imine after 24h. NMR in C_6D_6.

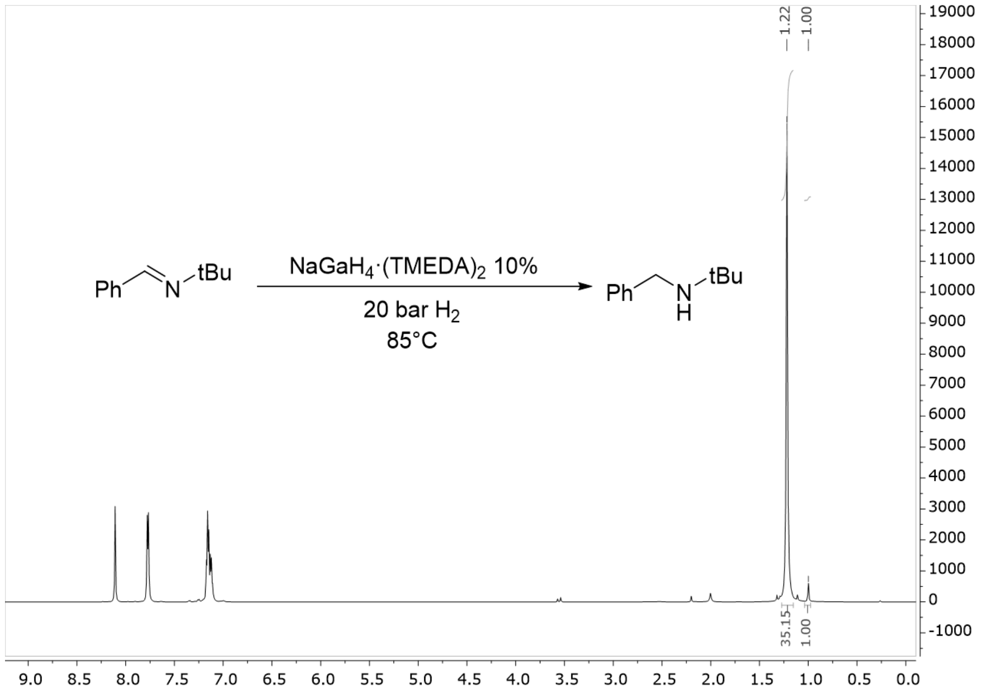

Figure S7: Catalytic reduction of PhC(H)=N*t*Bu using 10 mol% $NaGaH_4$·$(TMEDA)_2$ at 20 bar H_2 and 85°C in neat imine after 24h. NMR in C_6D_6.

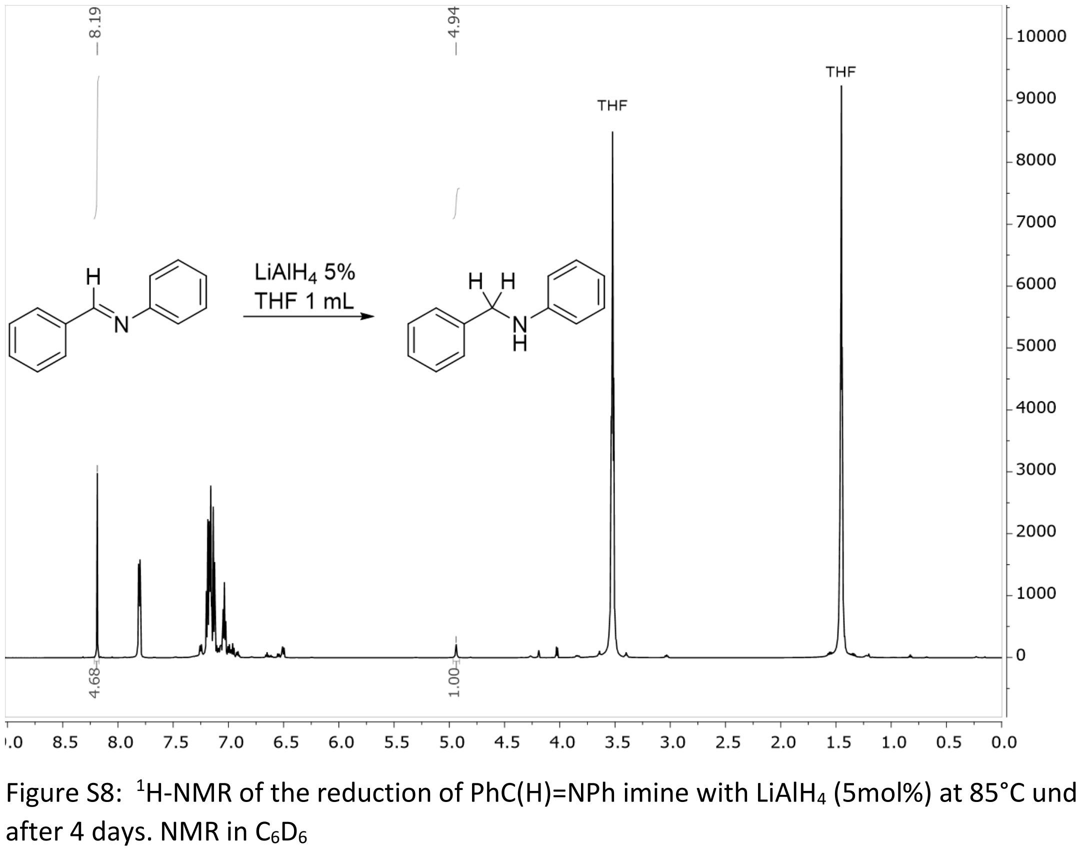

Figure S8: ^{1}H-NMR of the reduction of PhC(H)=NPh imine with $LiAlH_4$ (5mol%) at 85°C under 20 atm. H_2 after 4 days. NMR in C_6D_6

Complex Spectra

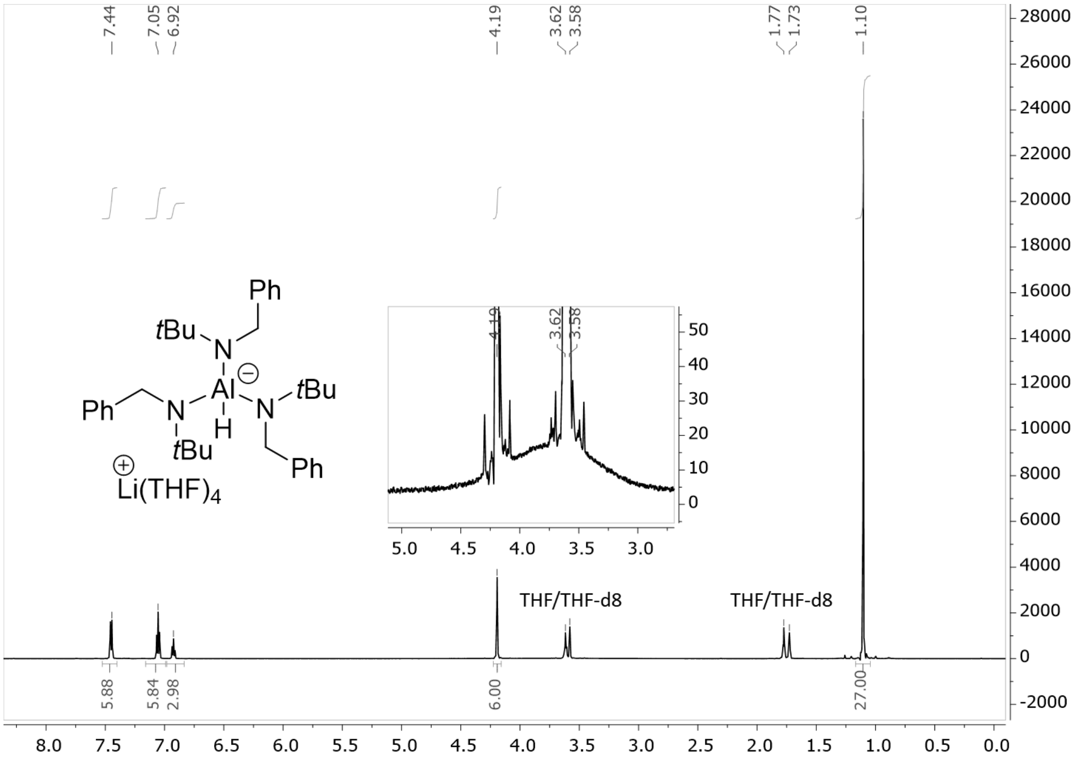

Figure S9: ^{1}H-NMR of $LiAlH[N(tBu)CH_2Ph]_3\cdot(THF)_4$ (**1**). NMR in THF-d_8.

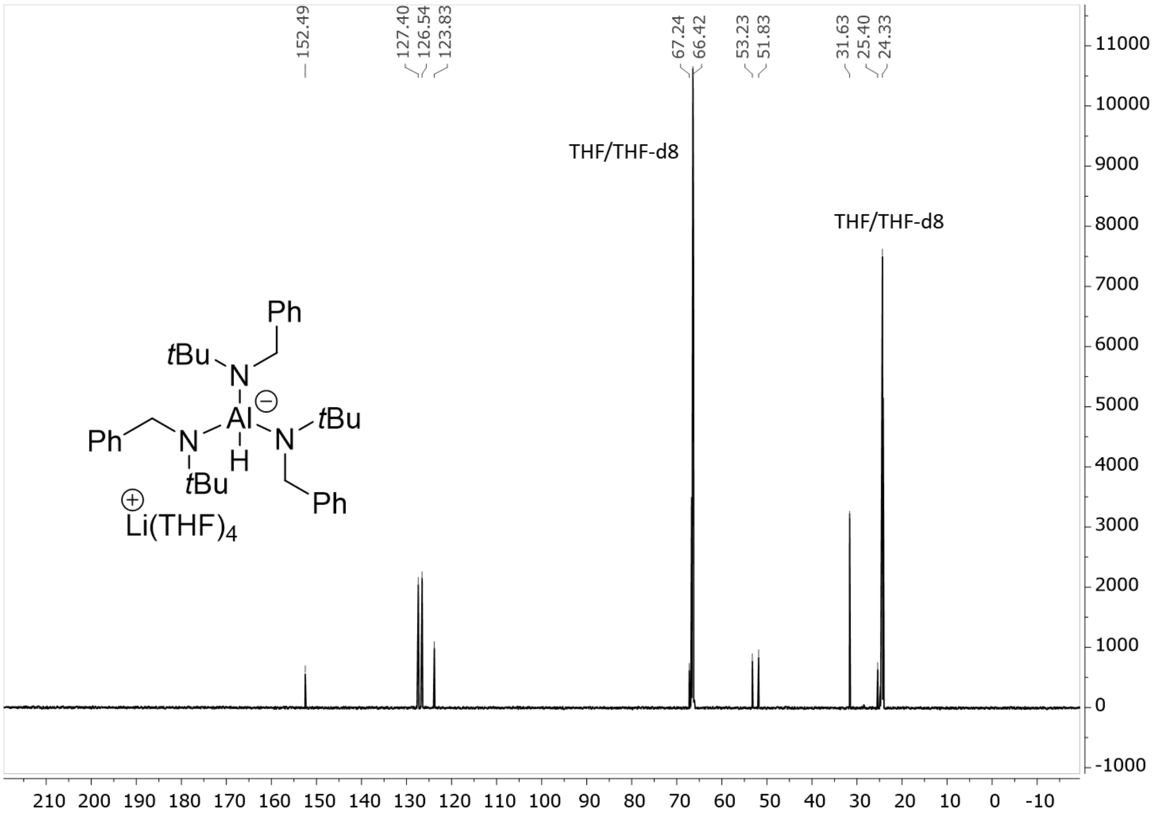

Figure S10: ^{13}C-NMR of $LiAlH[N(tBu)CH_2Ph]_3\cdot(THF)_4$ (**1**). NMR in THF-d_8.

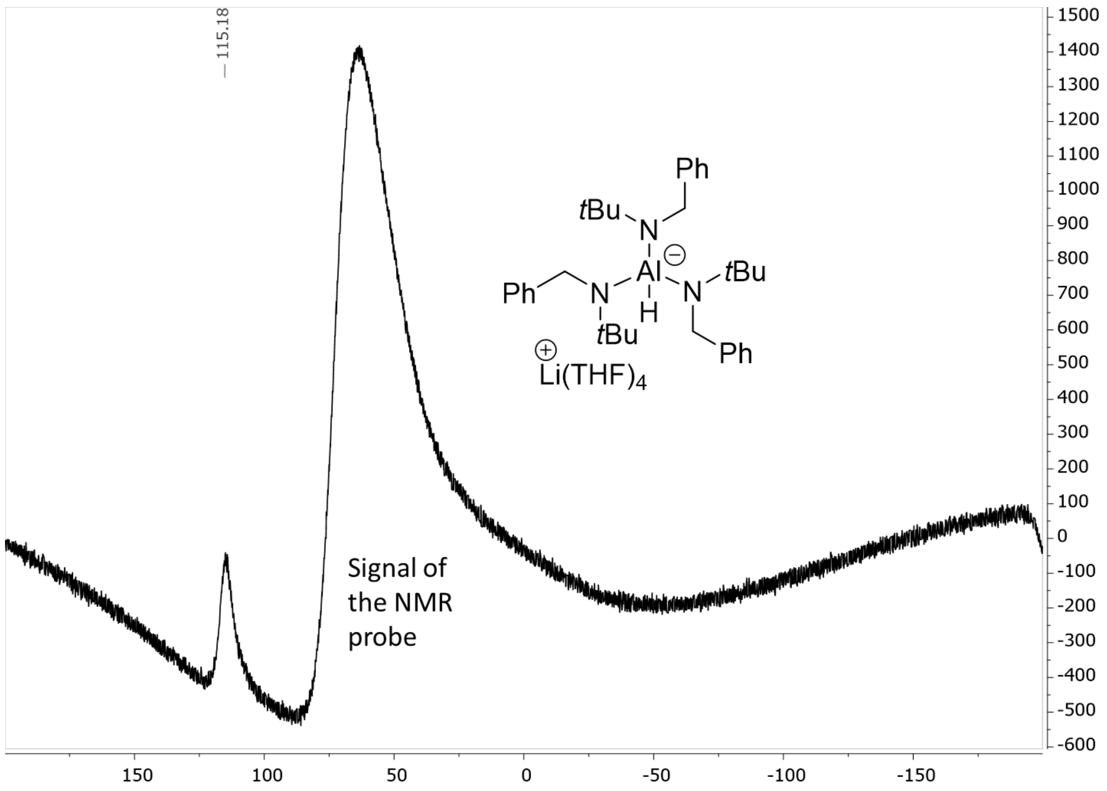

Figure S11: ^{27}Al-NMR of LiAlH[N(*t*Bu)CH$_2$Ph]$_3$·(THF)$_4$ (**1**). NMR in THF-*d*$_8$.

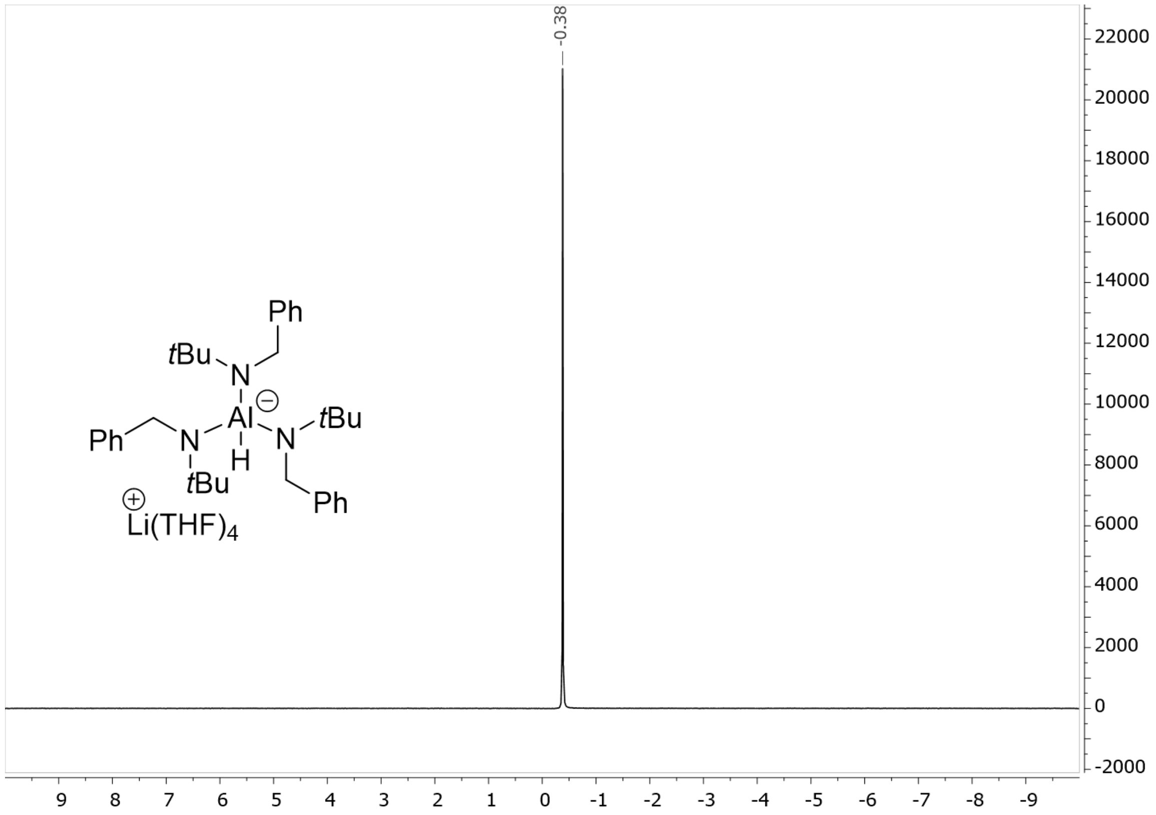

Figure S12: ^{7}Li-NMR of LiAlH[N(*t*Bu)CH$_2$Ph]$_3$·(THF)$_4$ (**1**). NMR in THF-*d*$_8$.

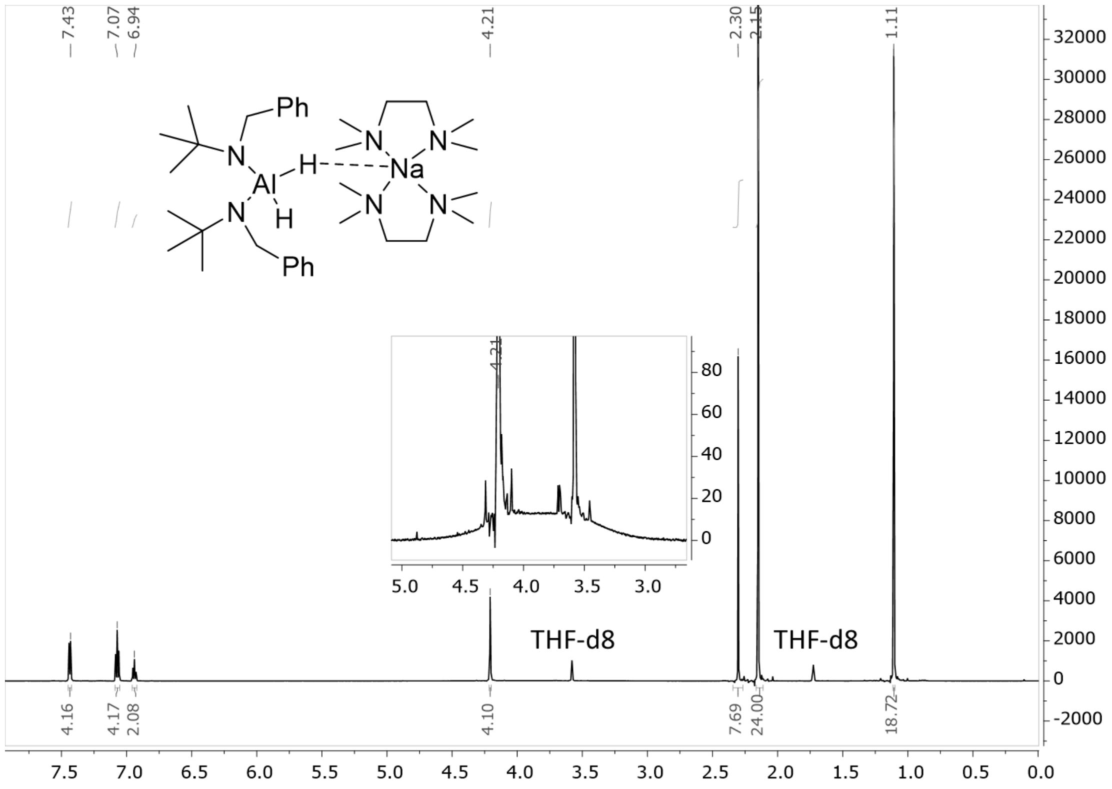

Figure S13: ^{1}H-NMR of $NaAlH_2[N(tBu)CH_2Ph]_2\cdot(TMEDA)_2$ (**2**). NMR in THF-d_8.

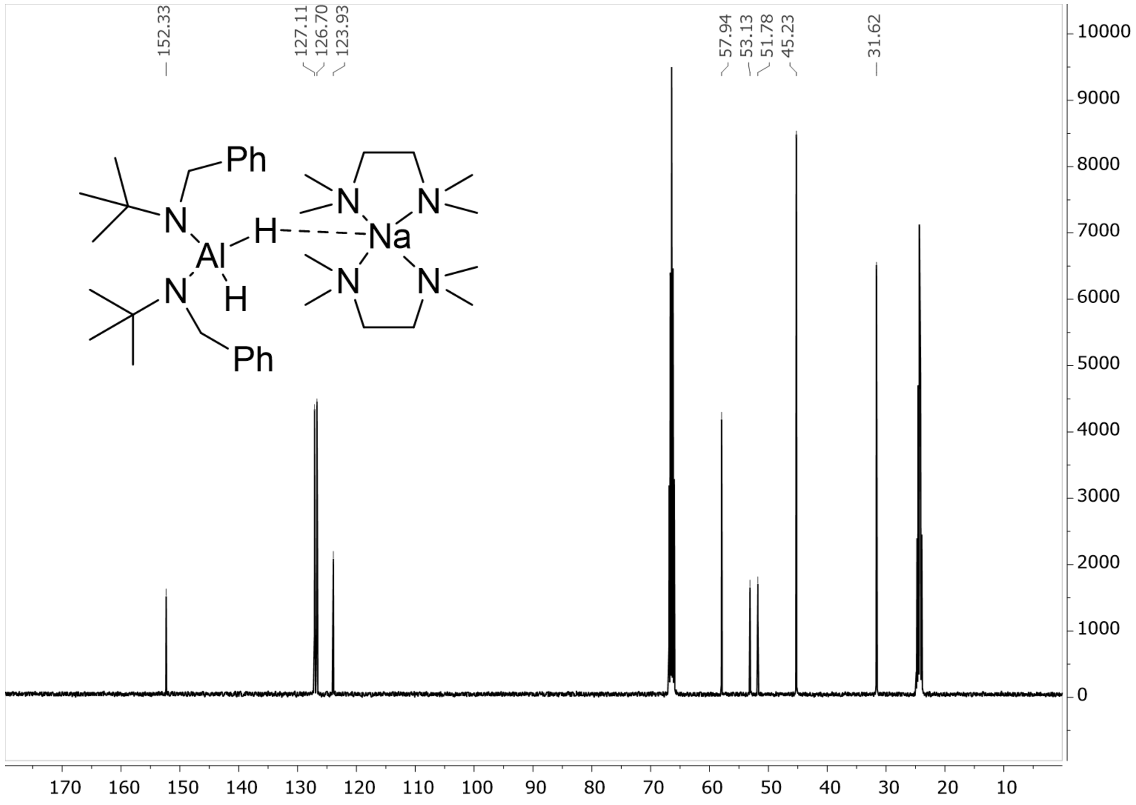

Figure S14: ^{13}C-NMR of $NaAlH_2[N(tBu)CH_2Ph]_2\cdot(TMEDA)_2$ (**2**). NMR in THF-d_8.

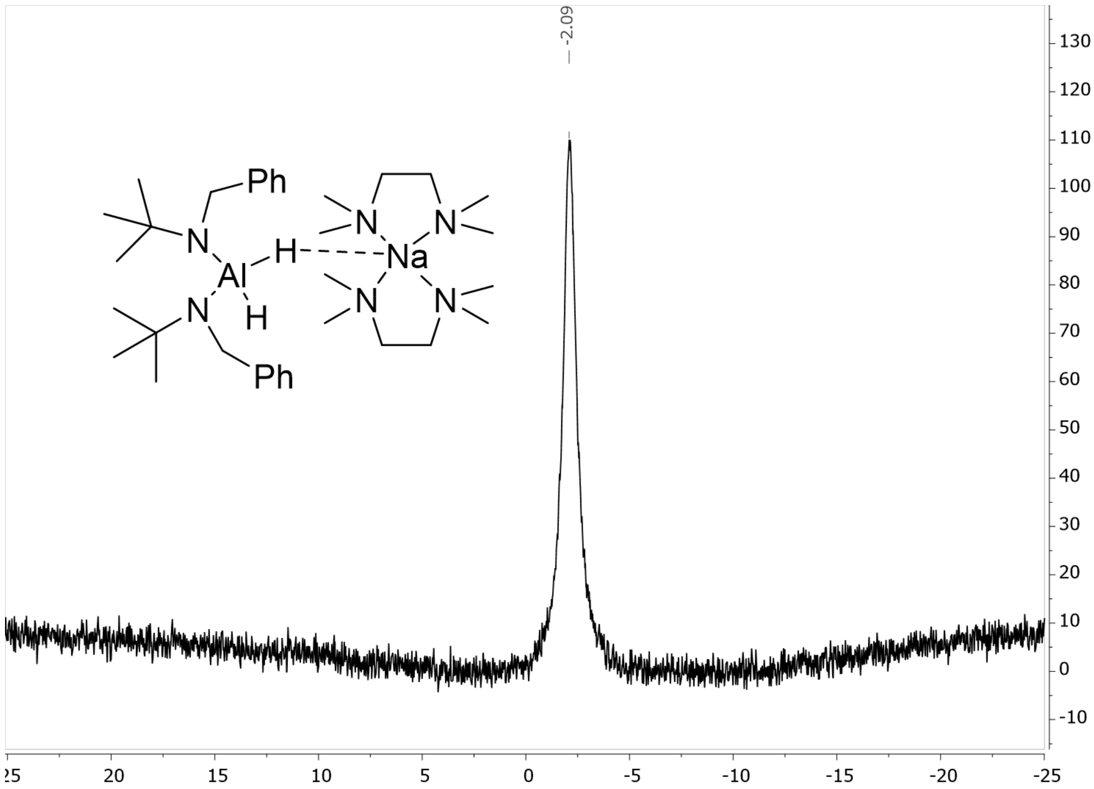

Figure S15: ^{23}Na-NMR of $NaAlH_2[N(tBu)CH_2Ph]_2\cdot(TMEDA)_2$ (**2**). NMR in THF-d_8.

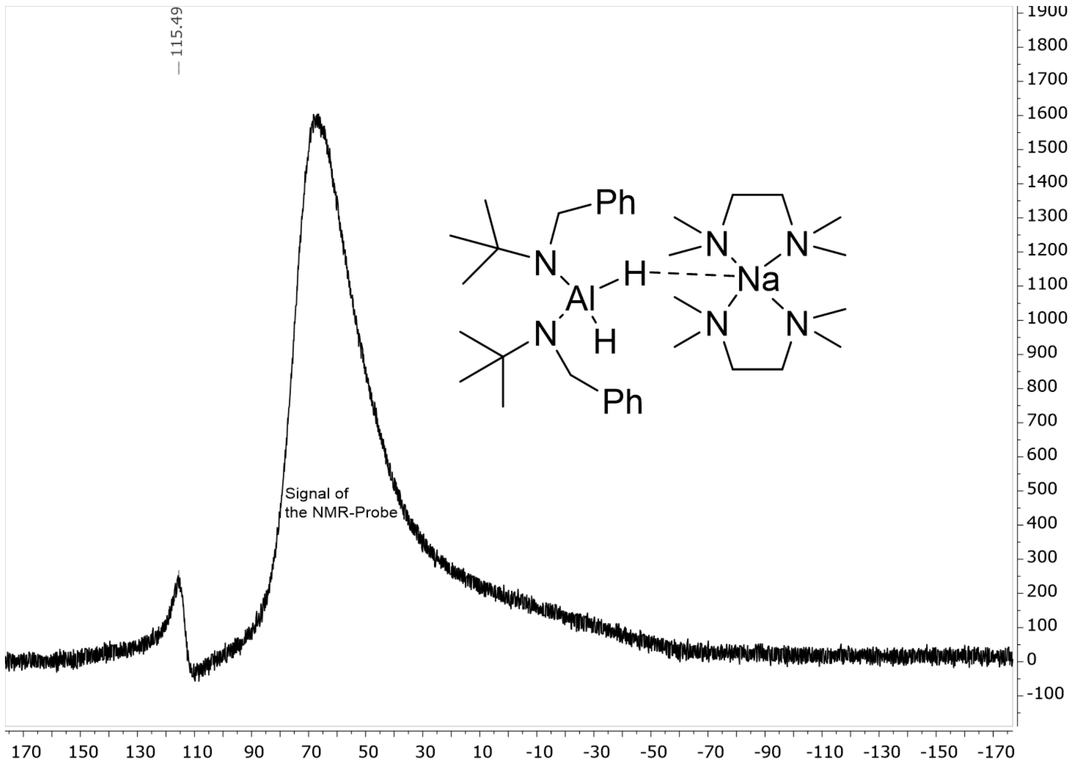

Figure S16: ^{27}Al-NMR of $NaAlH_2[N(tBu)CH_2Ph]_2\cdot(TMEDA)_2$ (**2**). NMR in THF-d_8.

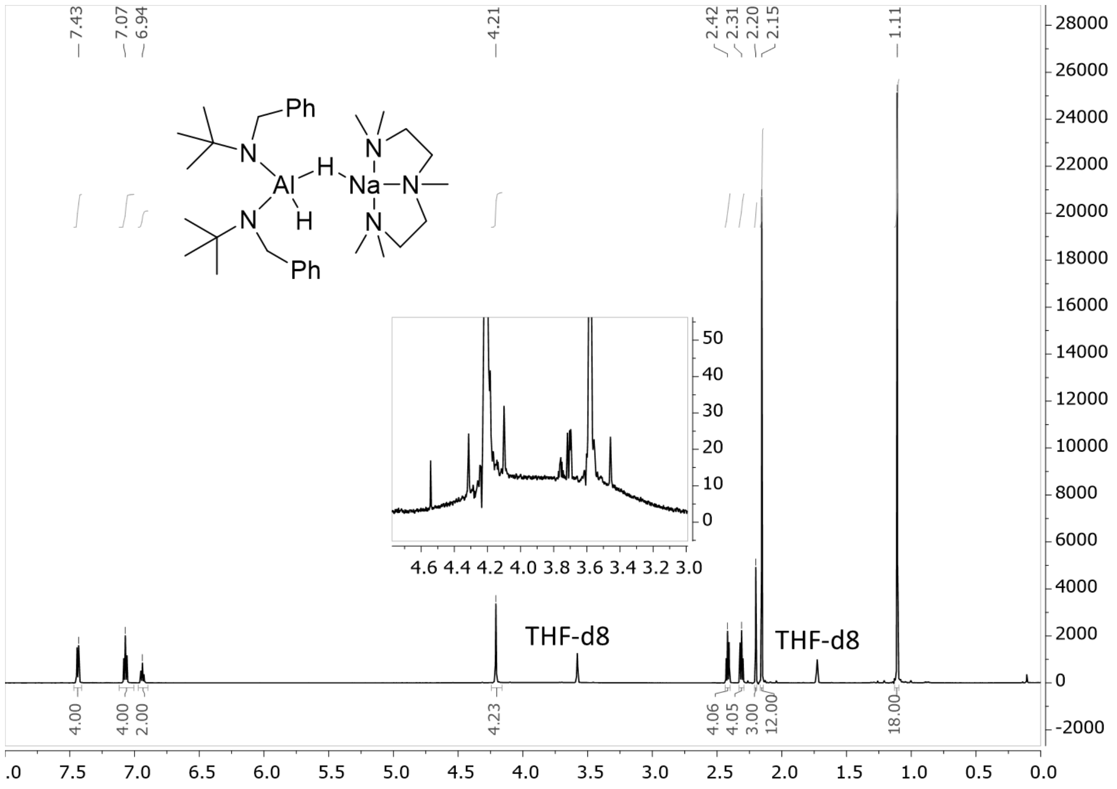

Figure S17: ^{1}H-NMR of $NaAlH_2[N(tBu)CH_2Ph]_2$·PMDTA (**3**). NMR in THF-d_8.

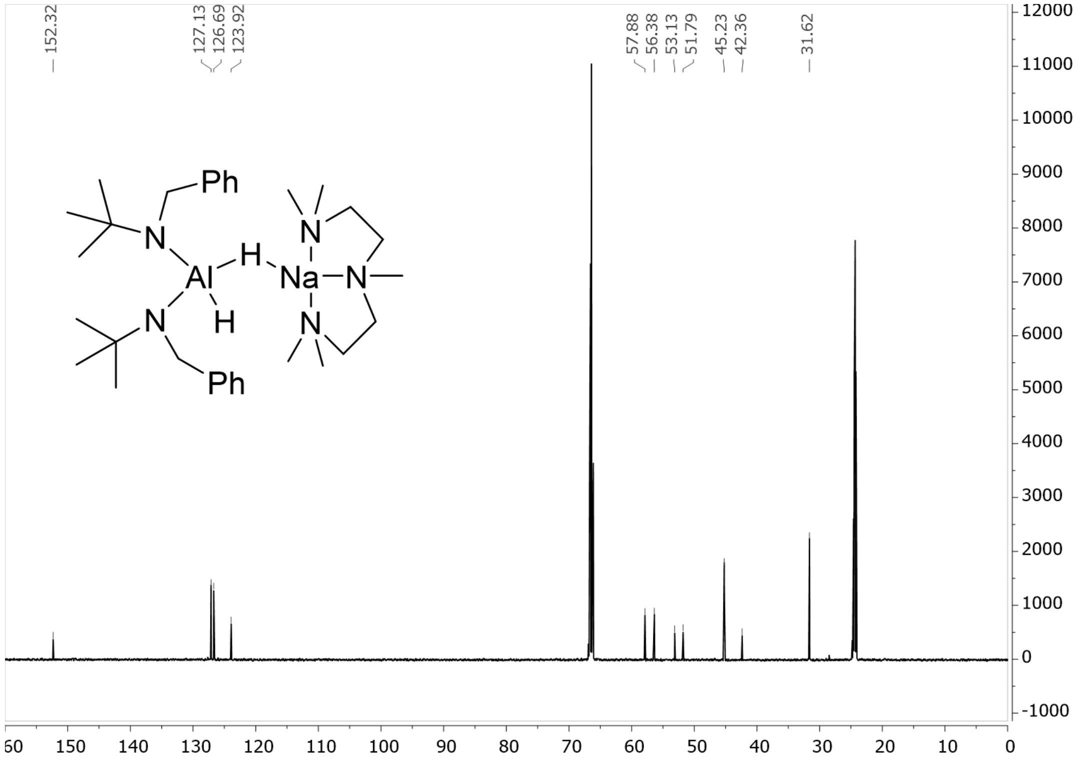

Figure S18: ^{13}C-NMR of $NaAlH_2[N(tBu)CH_2Ph]_2$·PMDTA (**3**). NMR in THF-d_8.

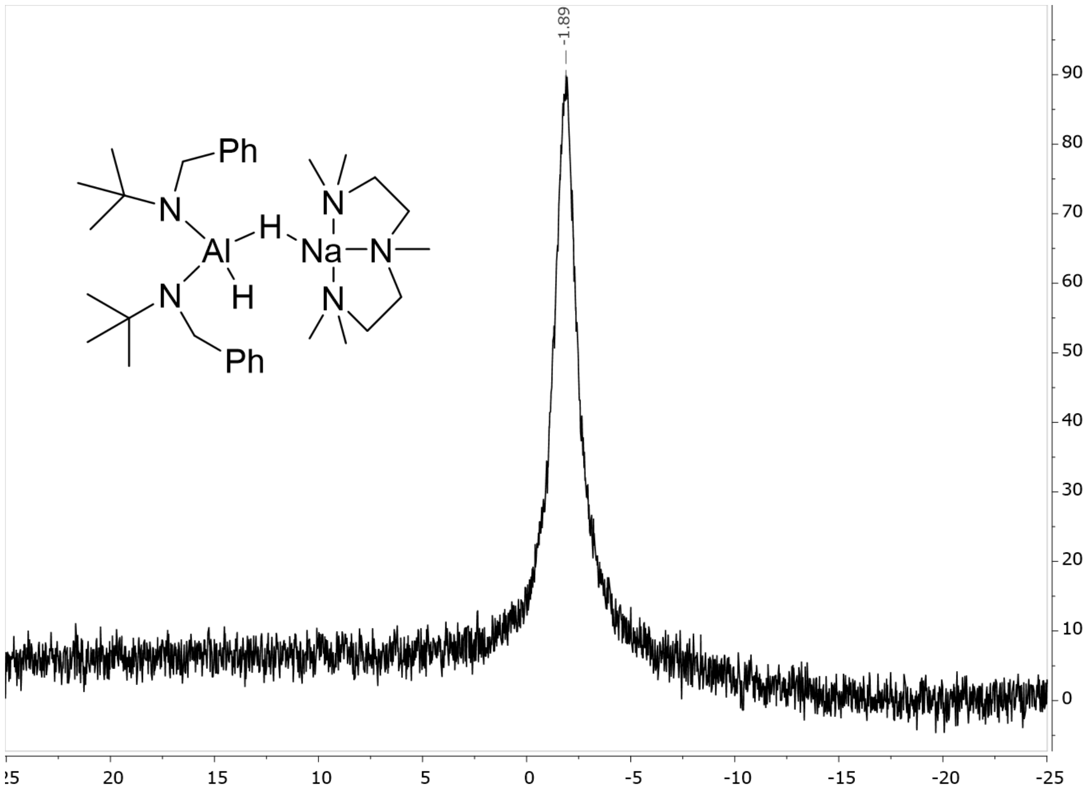

Figure S19: ^{23}Na-NMR of $NaAlH_2[N(tBu)CH_2Ph]_2$·PMDTA (**3**). NMR in THF-d_8.

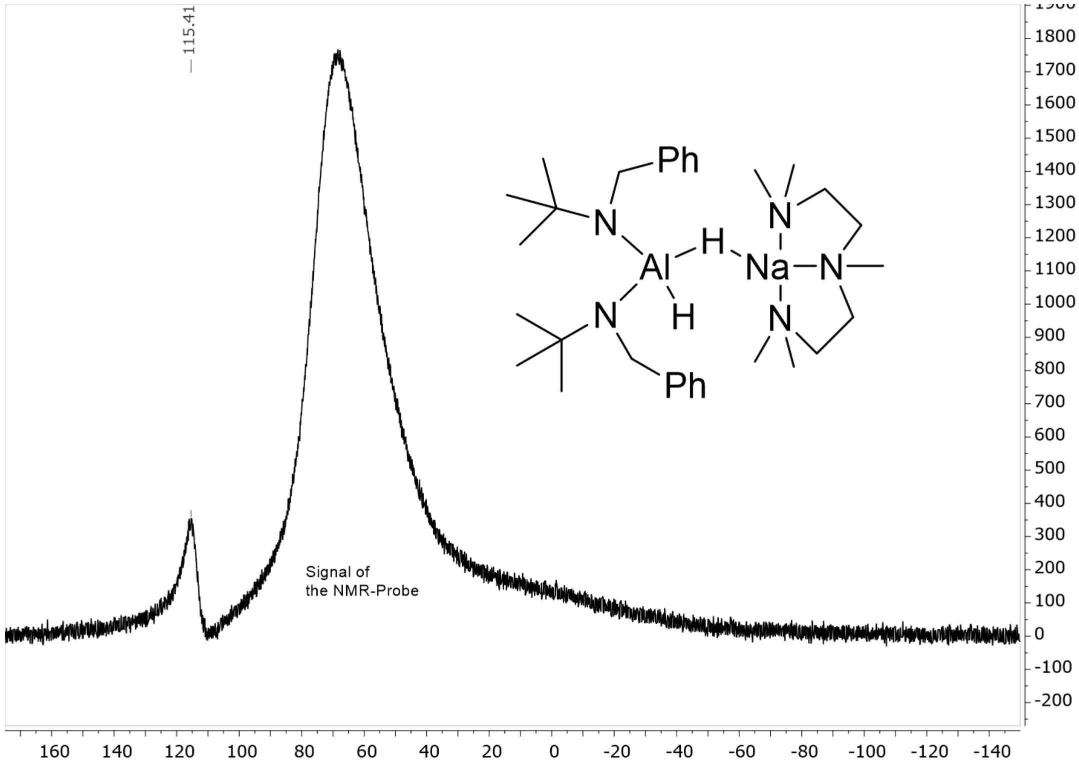

Figure S20: ^{27}Al-NMR of $NaAlH_2[N(tBu)CH_2Ph]_2$·PMDTA (**3**). NMR in THF-d_8.

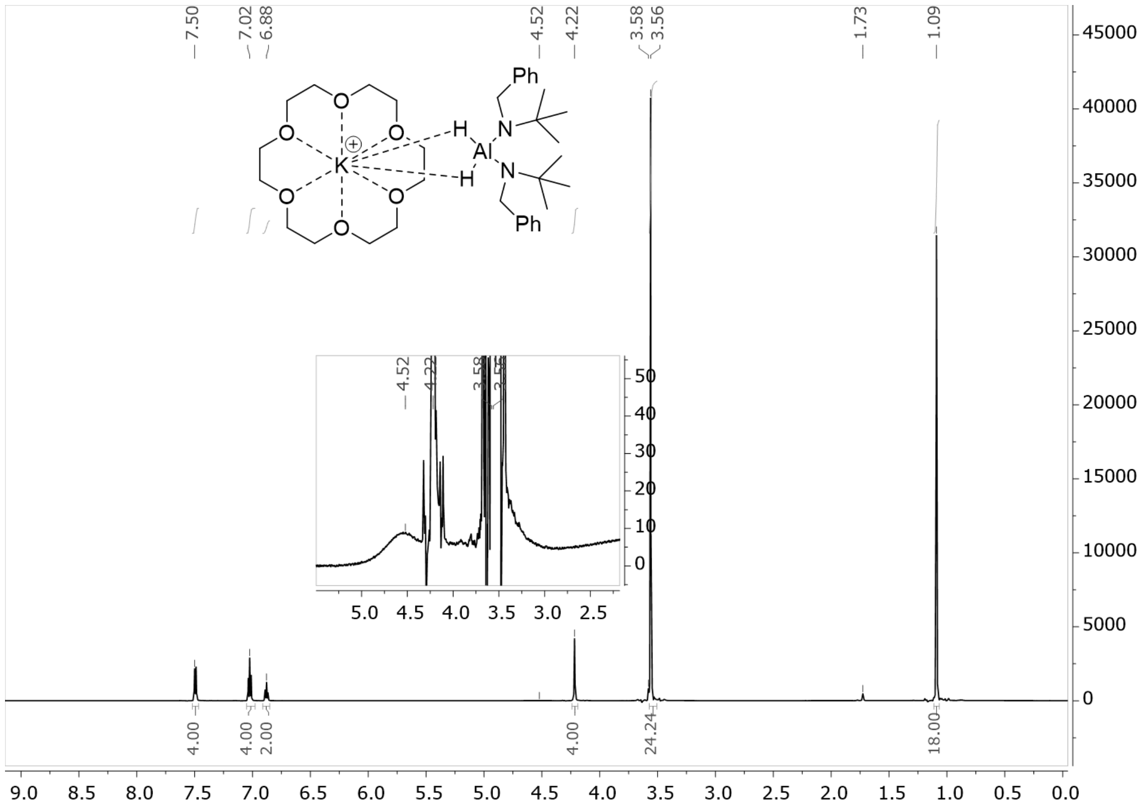

Figure S21: ^{1}H-NMR of $KAlH_2[N(tBu)CH_2Ph]_2$·18-crown-6 (**4**). NMR in THF-d_8.

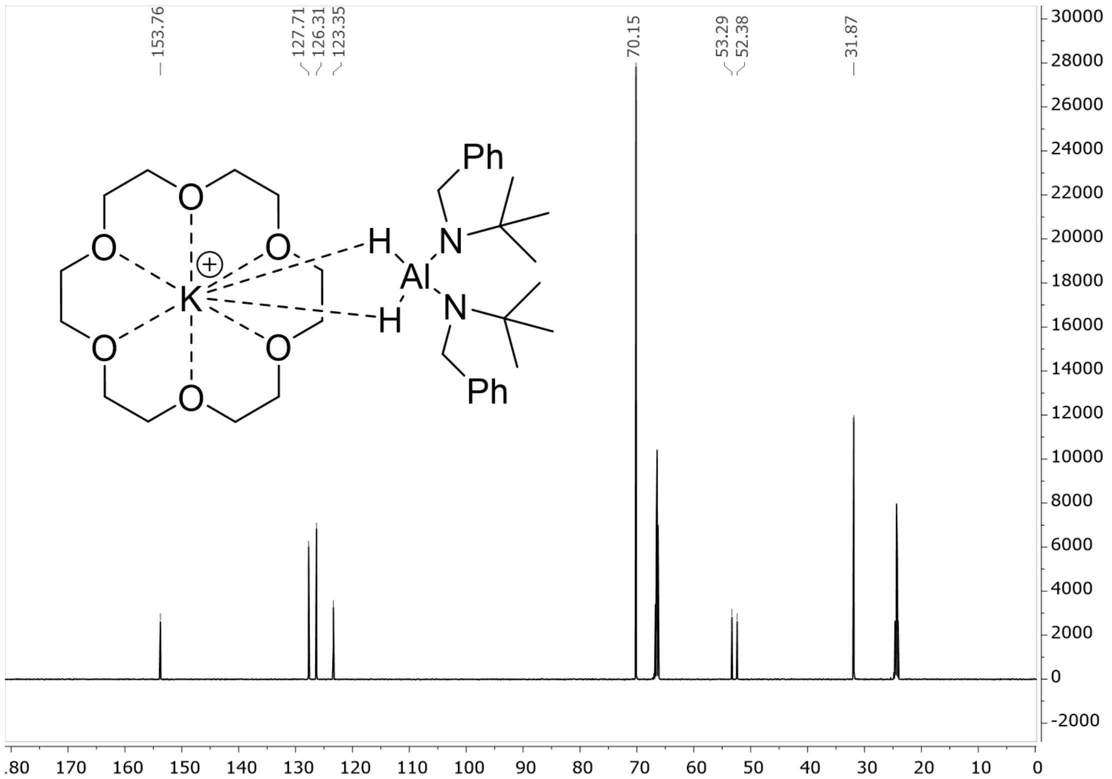

Figure S22: ^{13}C-NMR of $KAlH_2[N(tBu)CH_2Ph]_2$·18-crown-6 (**4**). NMR in THF-d_8.

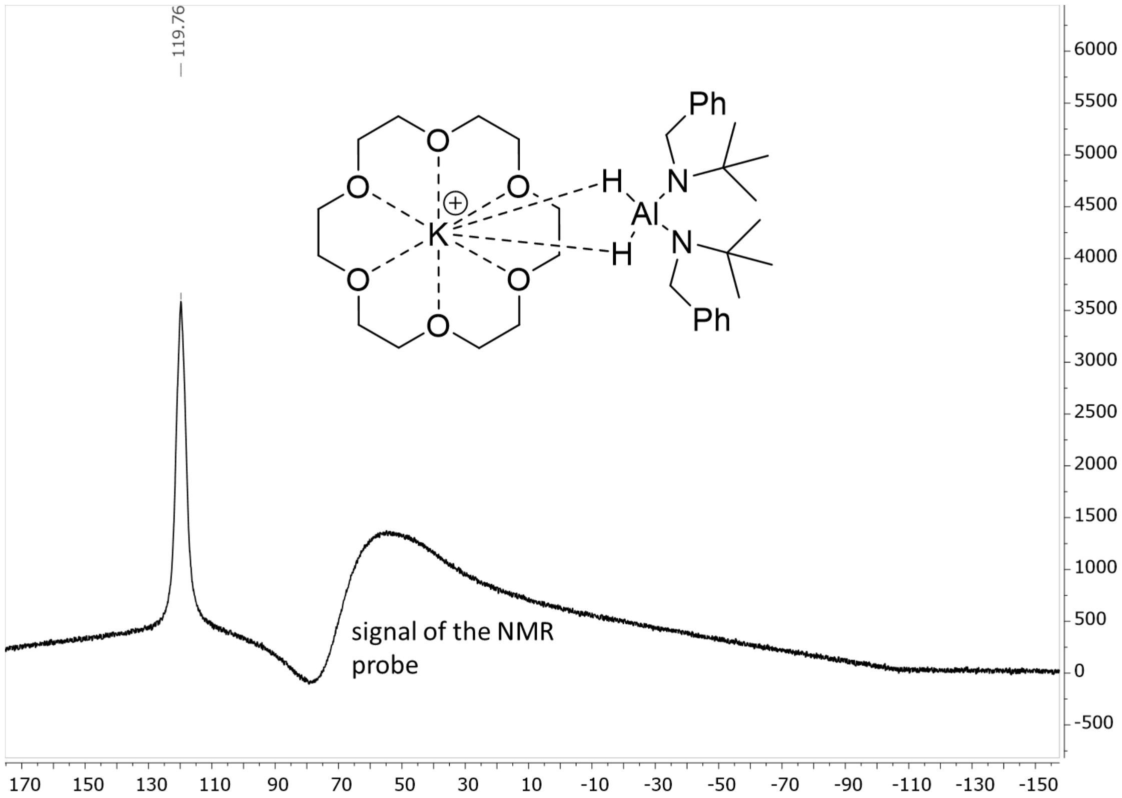

Figure S23: ^{27}Al-NMR of $KAlH_2[N(tBu)CH_2Ph]_2$·18-crown-6 (**4**). NMR in THF-d_8.

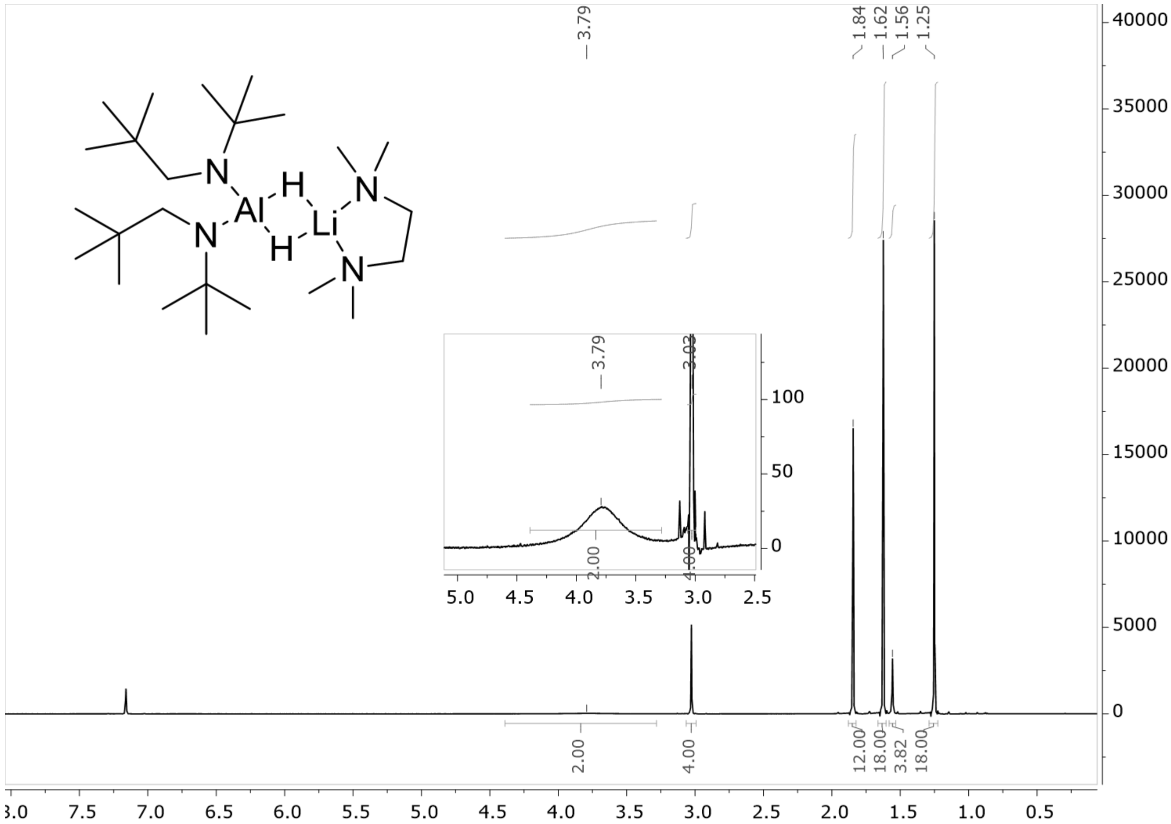

Fig. S24: ^{1}H-NMR of $LiAlH_2[N(tBu)CH_2tBu]_2$·(TMEDA) (**5**). NMR in C_6D_6.

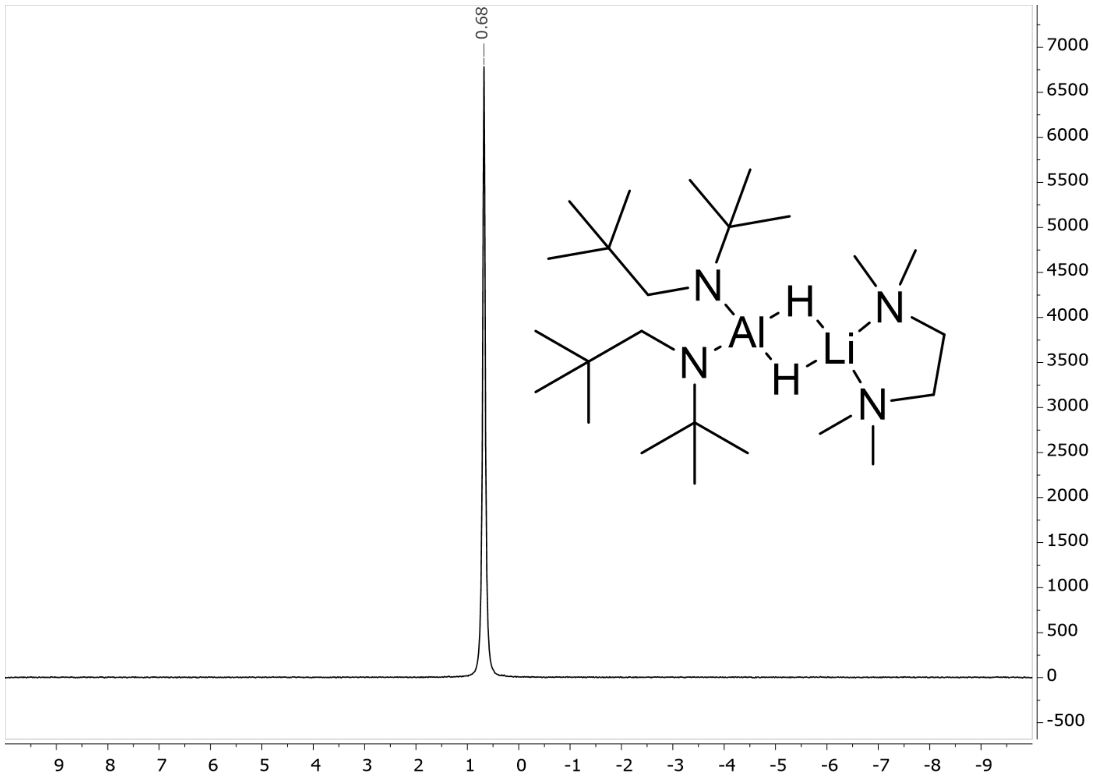

Fig. S25: ^{7}Li-NMR of $LiAlH_2[N(tBu)CH_2tBu]_2$·(TMEDA) (**5**). NMR in C_6D_6.

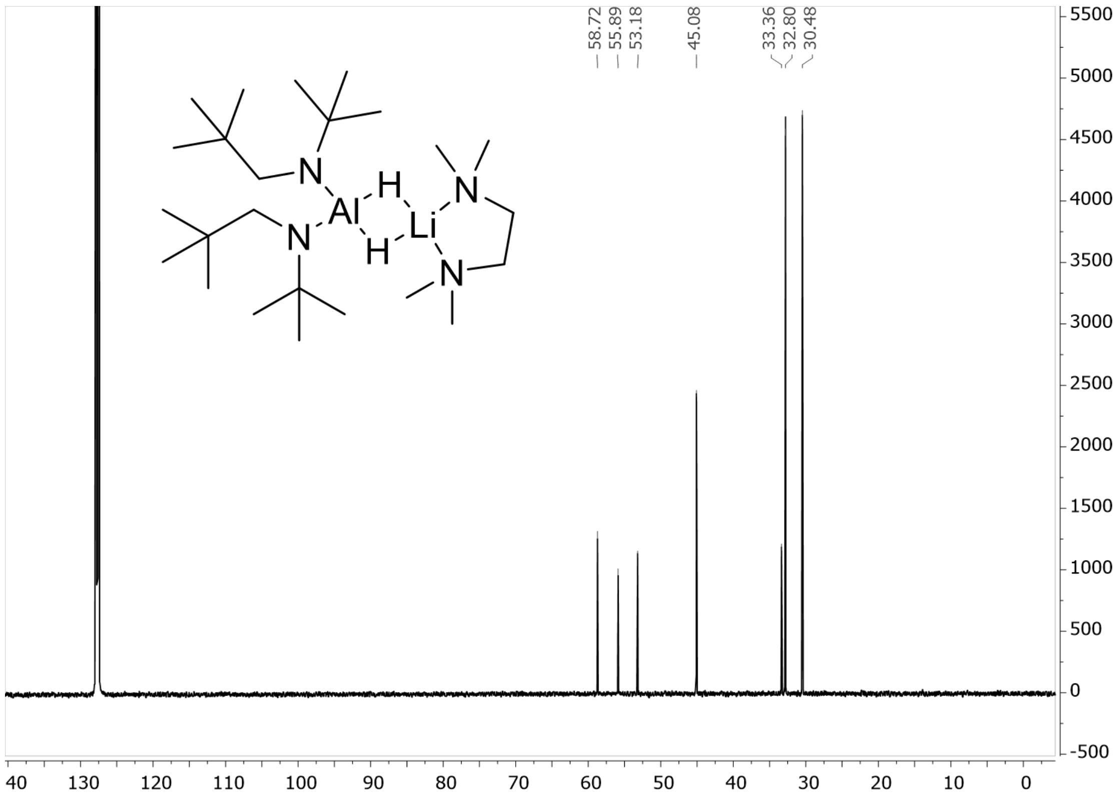

Fig. S26: ^{13}C-NMR of $LiAlH_2[N(tBu)CH_2tBu]_2$·(TMEDA) (**5**). NMR in C_6D_6.

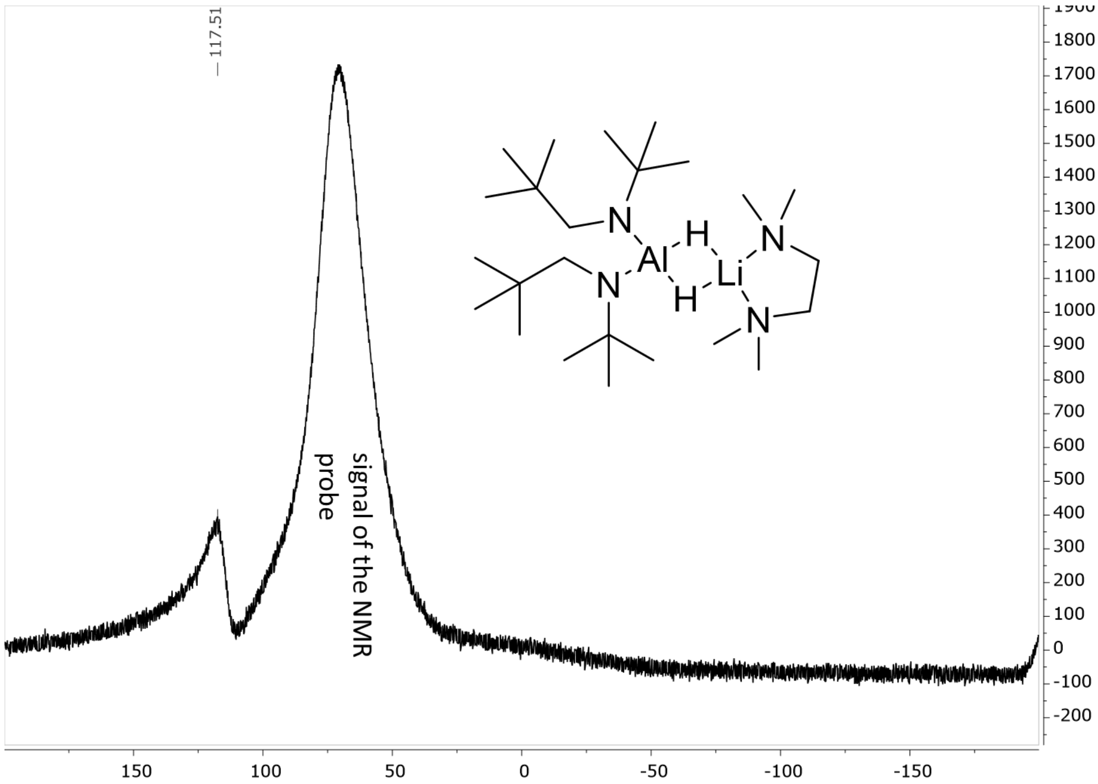

Fig. S27: ^{27}Al-NMR of $LiAlH_2[N(\textit{t}Bu)CH_2\textit{t}Bu]_2\cdot(TMEDA)$ (**5**). NMR in C_6D_6.

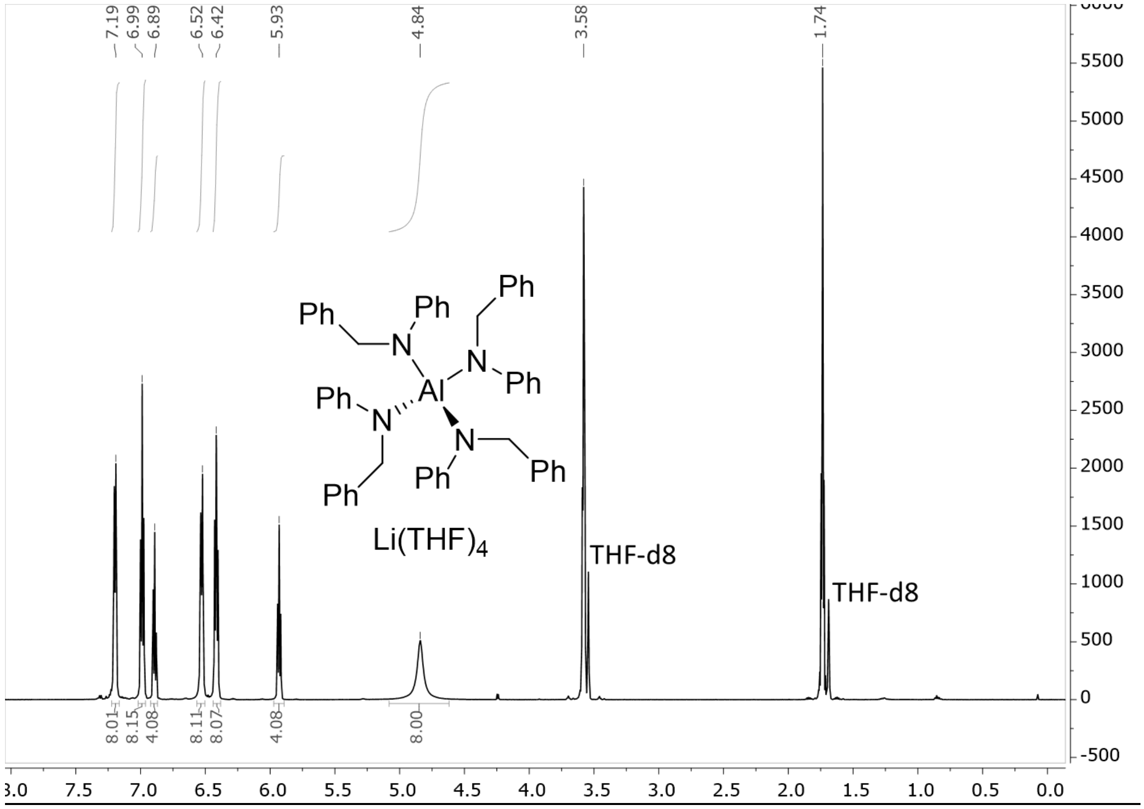

Figure S28: ^{1}H-NMR of $LiAl[N(Ph)CH_2Ph]_4\cdot(THF)_4$ (**6**). NMR in THF-d_8.

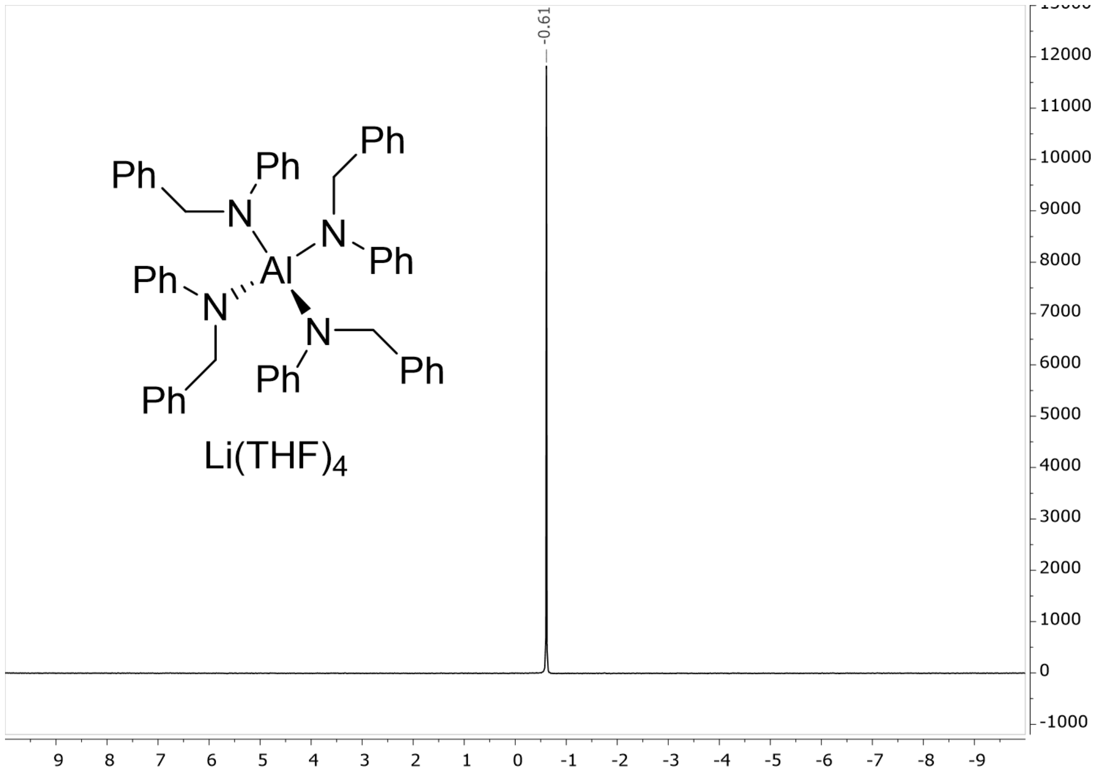

Figure S29: ^{7}Li-NMR of LiAl[N(Ph)CH$_2$Ph]$_4$·(THF)$_4$ (**6**). NMR in THF-d_8.

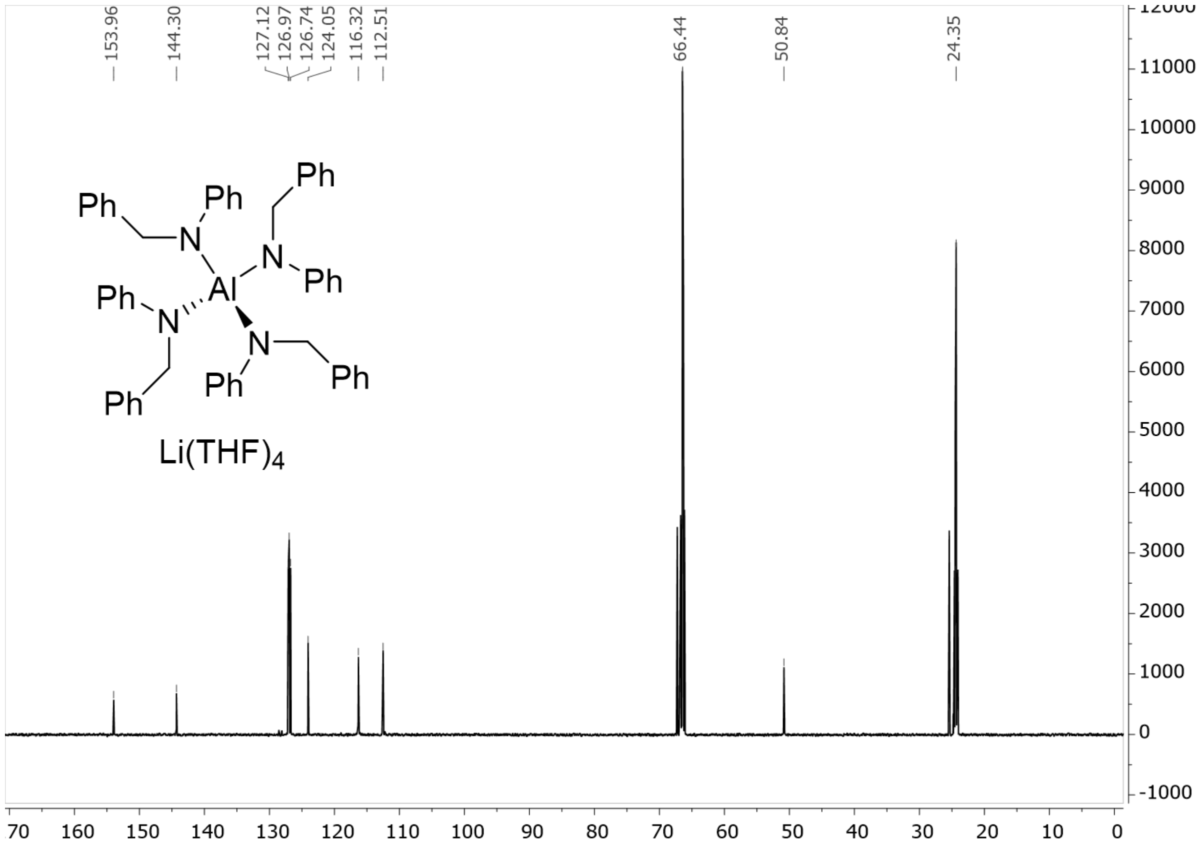

Figure S30: ^{13}C-NMR of LiAl[N(Ph)CH$_2$Ph]$_4$·(THF)$_4$ (**6**). NMR in THF-d_8.

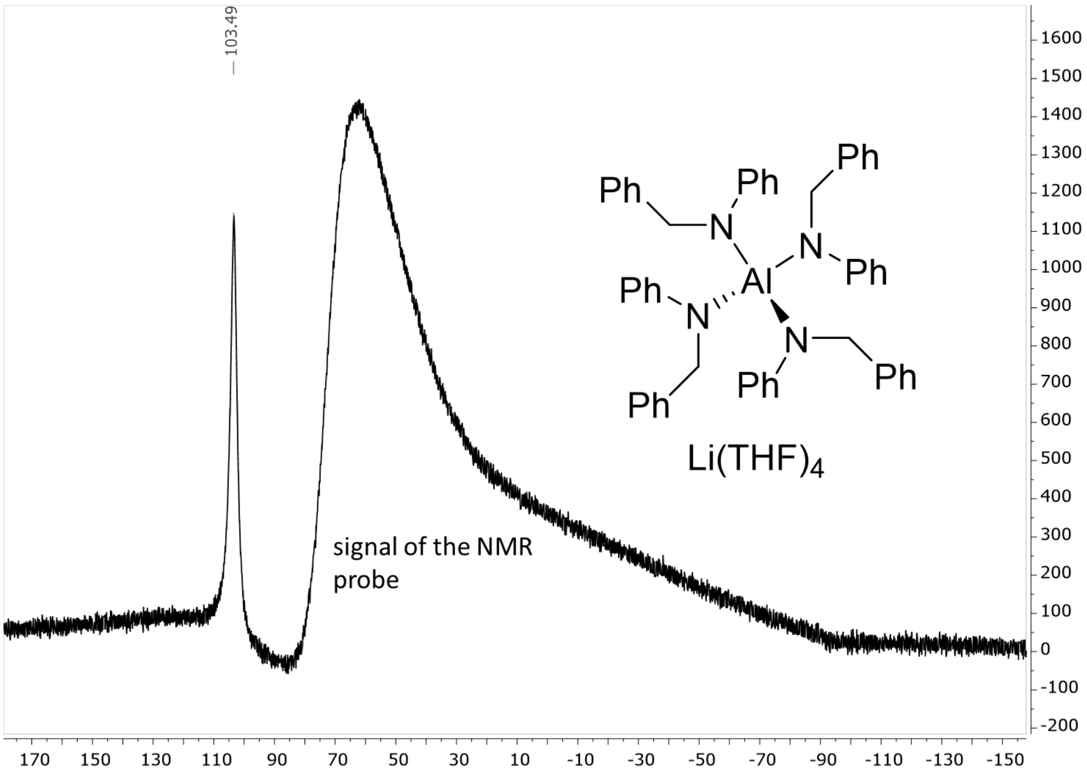

Figure S31: ^{27}Al-NMR of $LiAl[N(Ph)CH_2Ph]_4 \cdot (THF)_4$ (**6**). NMR in THF-d_8.

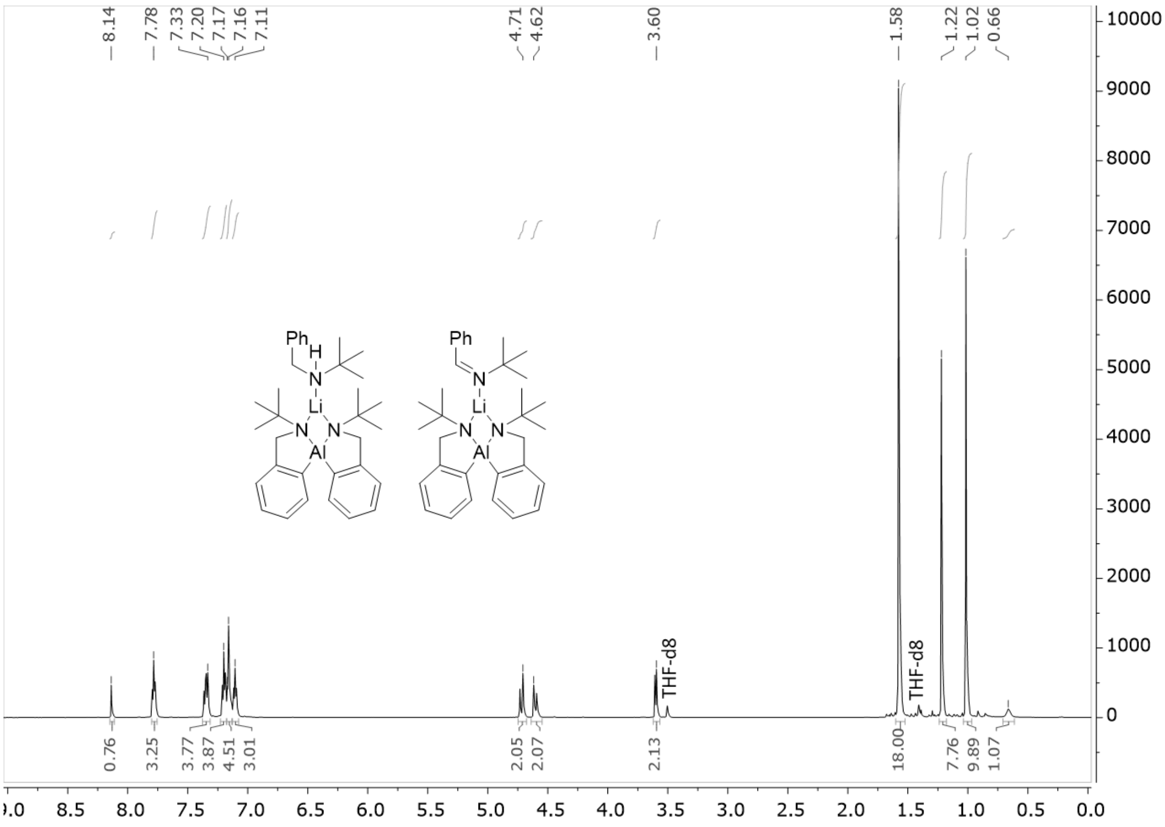

Figure S32: ^{1}H-NMR of complex **7**. NMR in C_6D_6/THF-d_8 5/1.

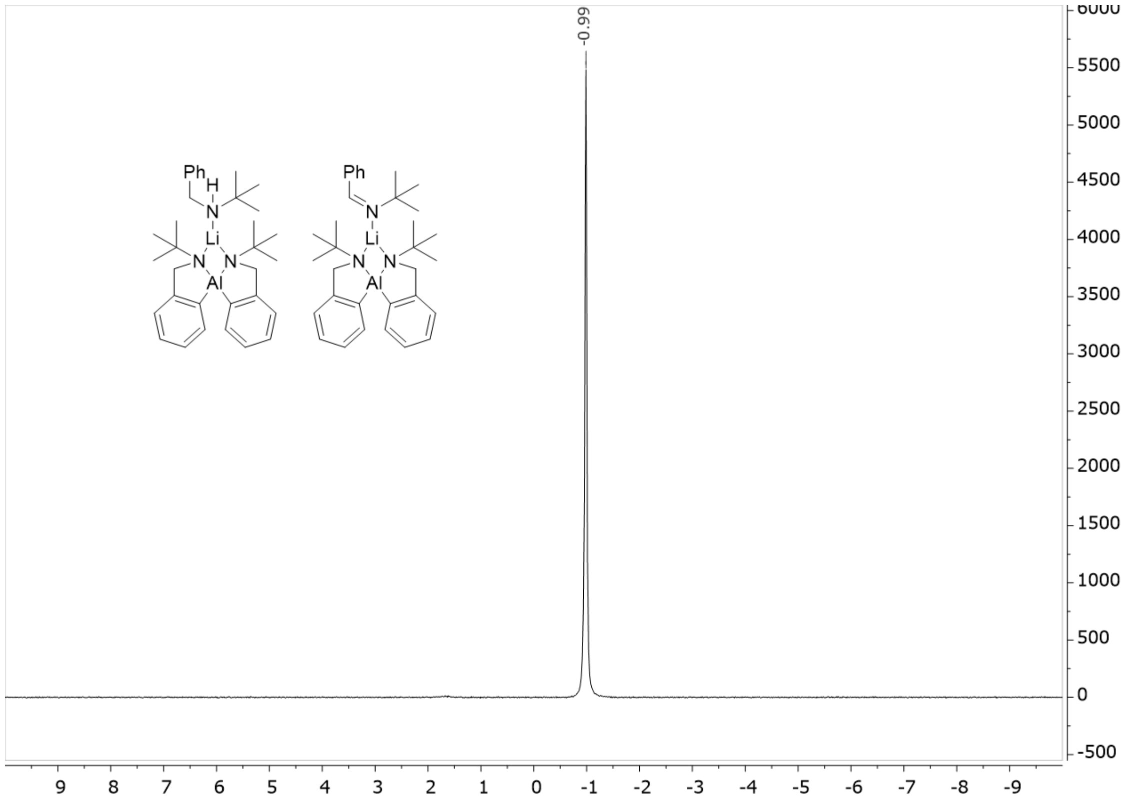

Figure S33: ^{7}Li-NMR of **7**. NMR in C_6D_6/THF-d_8 5/1.

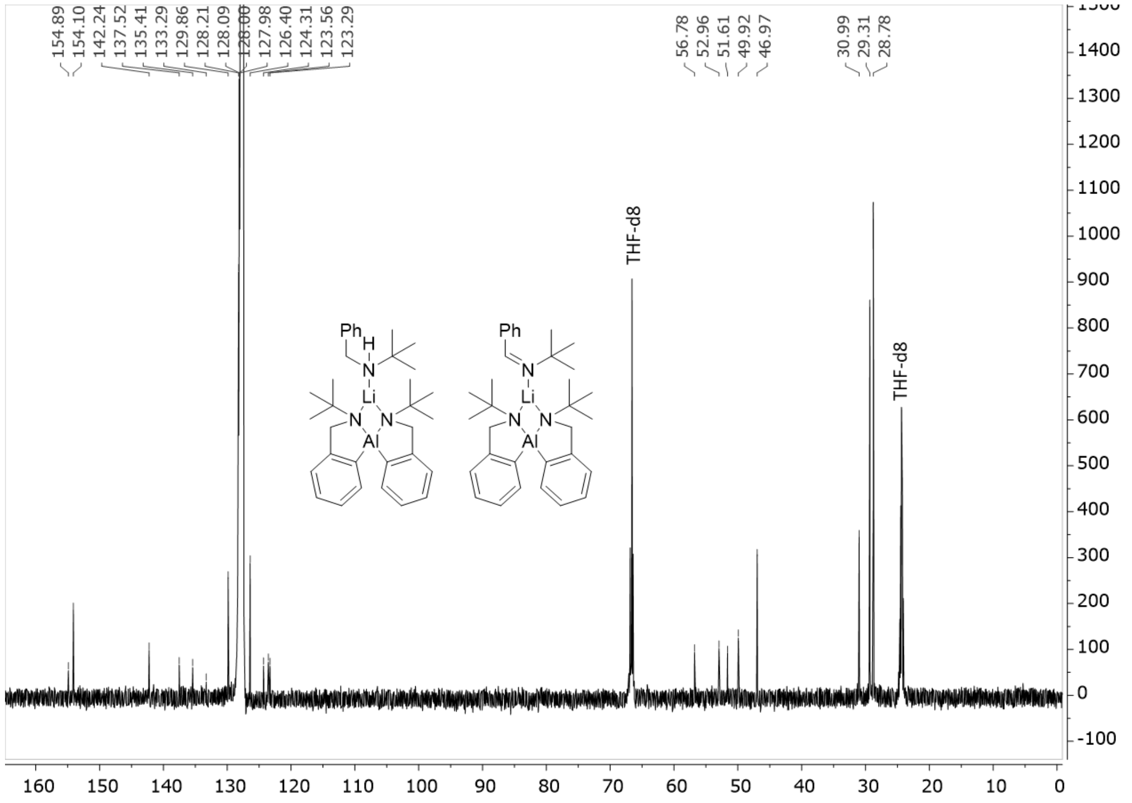

Figure S34: ^{13}C-NMR of **7**. NMR in C_6D_6/THF-d_8 5/1.

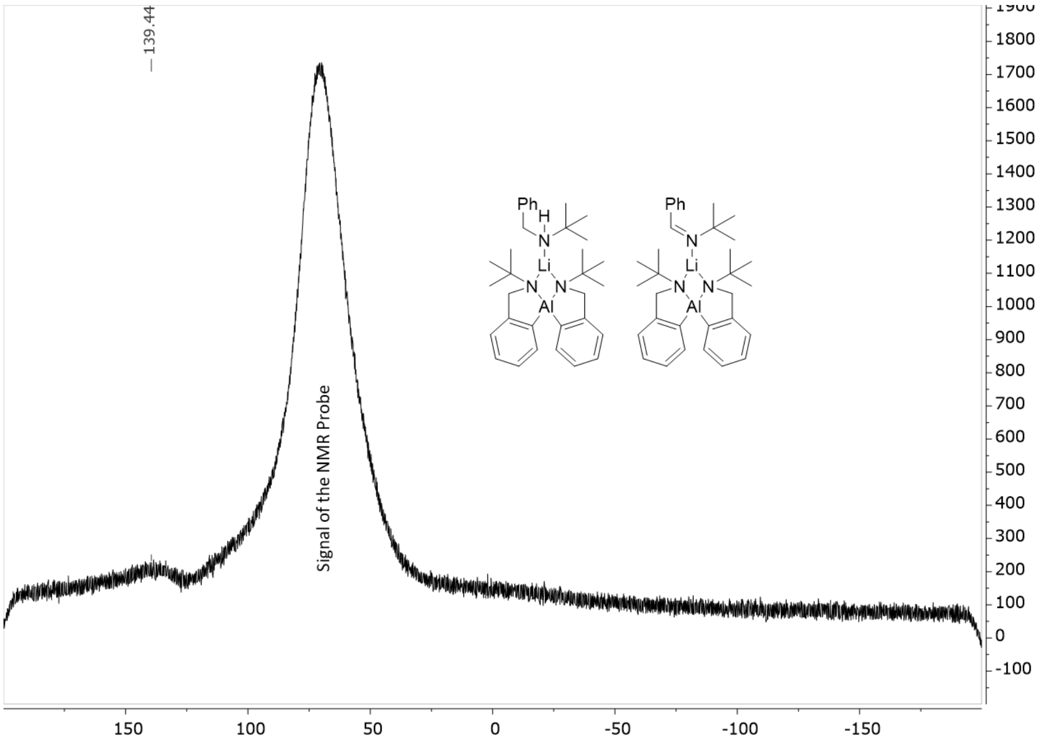

Figure S35: ^{27}Al-NMR of **7**. NMR in C_6D_6/THF-d_8 5/1.

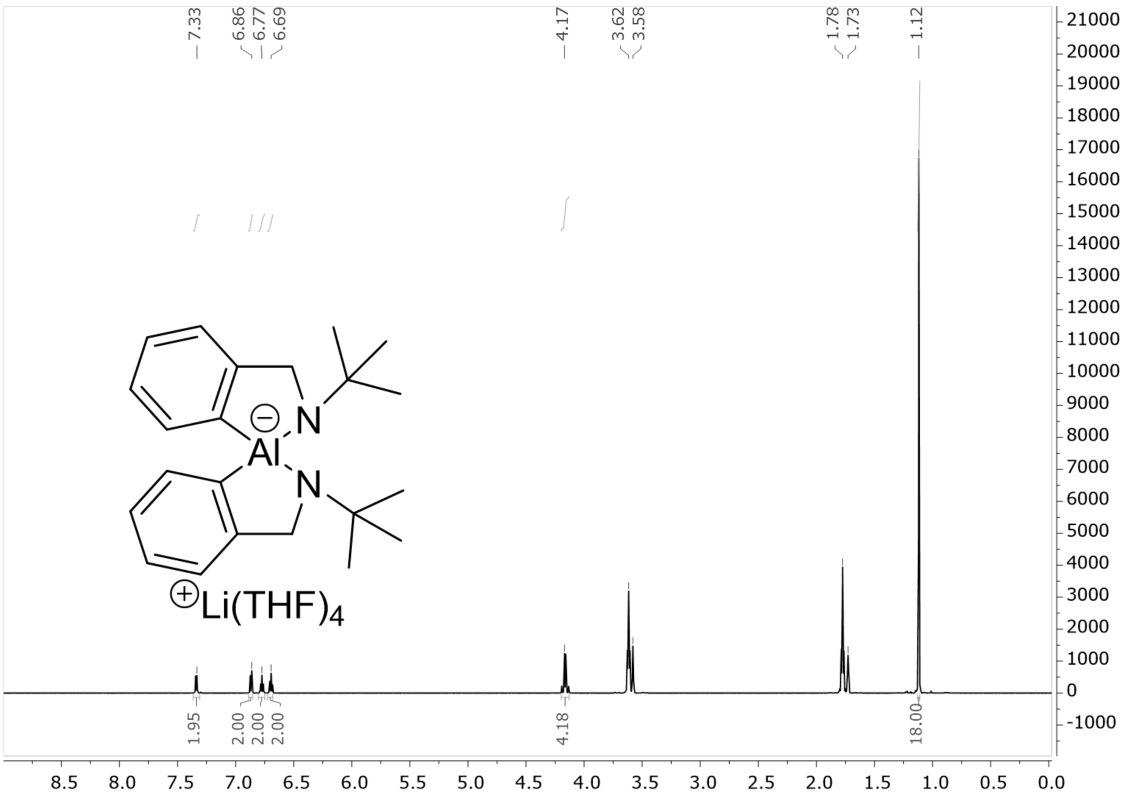

Figure S36: ^{1}H-NMR of complex **8**. NMR in THF-d_8.

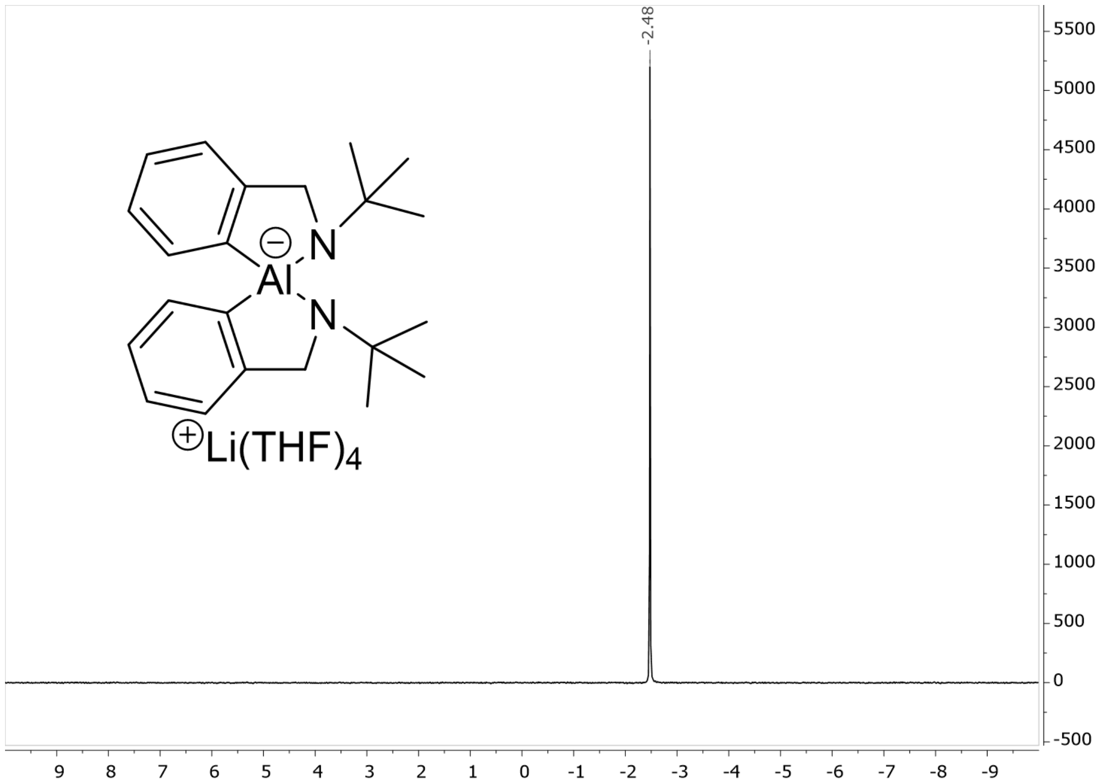

Figure S37: ^{7}Li-NMR of complex **8**. NMR in THF-d_8.

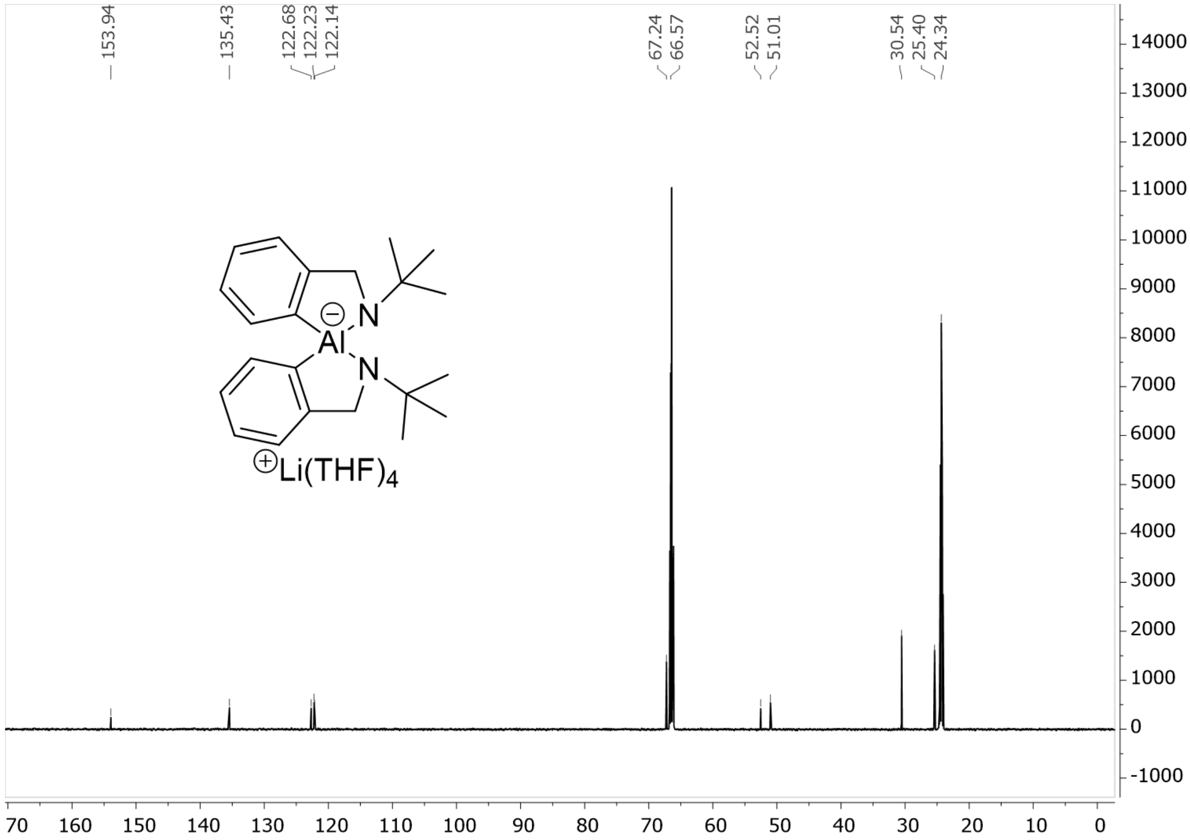

Figure S38: ^{13}C-NMR of complex **8**. NMR in THF-d_8.

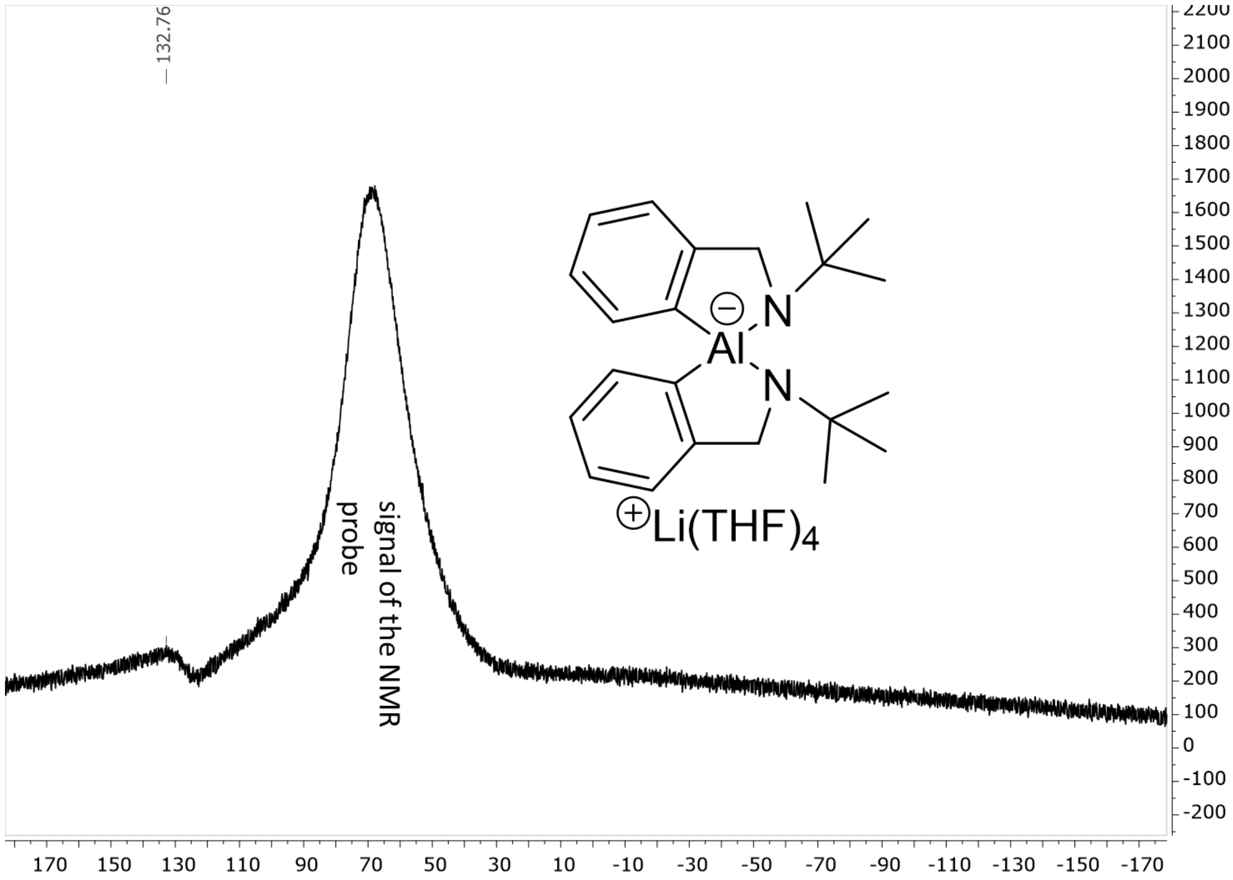

Figure S39: ^{27}Al-NMR of complex **8**. NMR in THF-d_8.

Crystal Structures

Structure determination of LiAlH[N(*t*Bu)CH$_2$Ph]$_3$·(THF)$_4$ (1)

A colorless crystal of LiAlH[N(*t*Bu)CH$_2$Ph]$_3$·(THF)$_4$ was embedded in inert perfluoropolyalkylether (viscosity 1800 cSt; ABCR GmbH) and mounted using a Hampton Research CryoLoop. The crystal was then flash cooled to 100.0(1) K in a nitrogen gas stream and kept at this temperature during the experiment. The crystal structure was measured on a SuperNova diffractometer with Atlas S2 detector using a CuKα microfocus source. The measured data was processed with the CrysAlisPro (v40.67a) software package.[S9] Using Olex2,[S5] the structure was solved with the ShelXT[S6] structure solution program using Intrinsic Phasing and refined with the ShelXL[S7] refinement package using Least Squares Minimization. Most hydrogen atoms were placed in ideal positions and refined as riding atoms with relative isotropic displacement parameters. The metal bound hydride was observed from difference Fourier maps and refined.

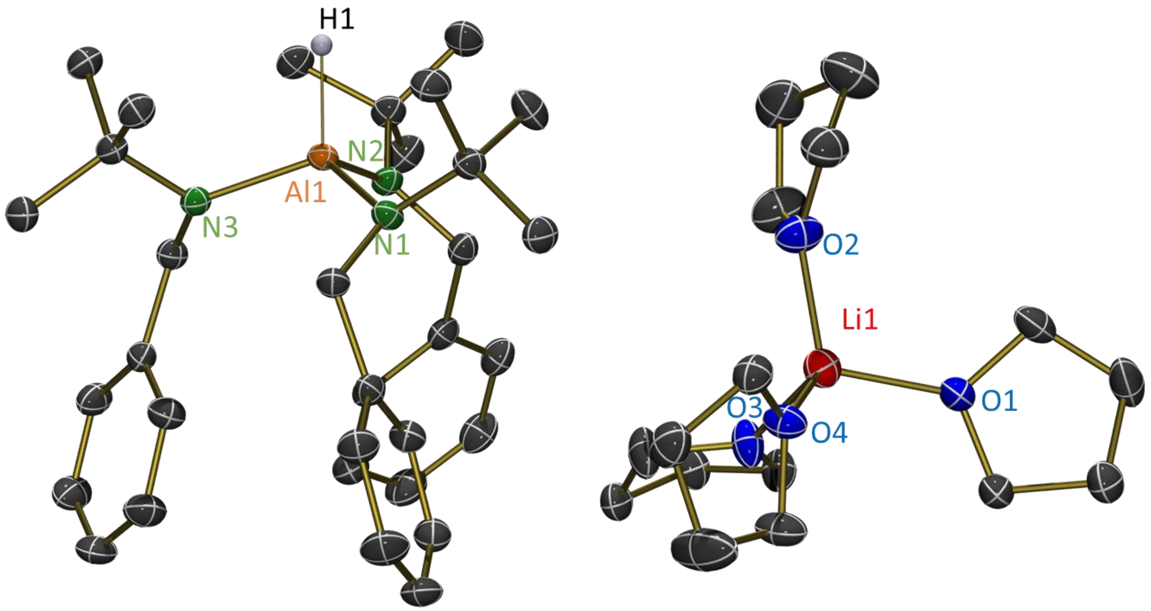

Figure S40: ORTEP-plot (50% probability) of LiAlH[N(*t*Bu)CH$_2$Ph]$_3$·(THF)$_4$ (**1**). H atoms except for the one bound to Al are omitted for clarity.

Structure determination of $NaAlH_2[N(tBu)CH_2Ph]_2{\cdot}(TMEDA)_2$ (2)

A colorless crystal of compound **1** was embedded in inert perfluoropolyalkylether (viscosity 1800 cSt; ABCR GmbH) and mounted using a Hampton Research CryoLoop. The crystal was then flash cooled to 100.00(11) K in a nitrogen gas stream and kept at this temperature during the experiment. The crystal structure was measured on a SuperNova diffractometer with Atlas S2 detector using a CuKα microfocus source. The measured data was processed with the CrysAlisPro (v40.53) software package.[S4] Using Olex2 [S5], the structure was solved with the ShelXT [S6] structure solution program using Intrinsic Phasing and refined with the ShelXL [S7] refinement package using Least Squares minimization. All non-hydrogen atoms were refined anisotropically. Most hydrogen atoms were placed in ideal positions and refined as riding atoms with relative isotropic displacement parameters. The positions of the aluminum bound hydrides were observed from difference Fourier maps and refined.
Disorder of one TMEDA ligand was observed and was modeled with the help of a rigid bond restraint (RIGU)[S8]. The relative occupancies of the two alternative orientations were refined to 0.39/0.61.

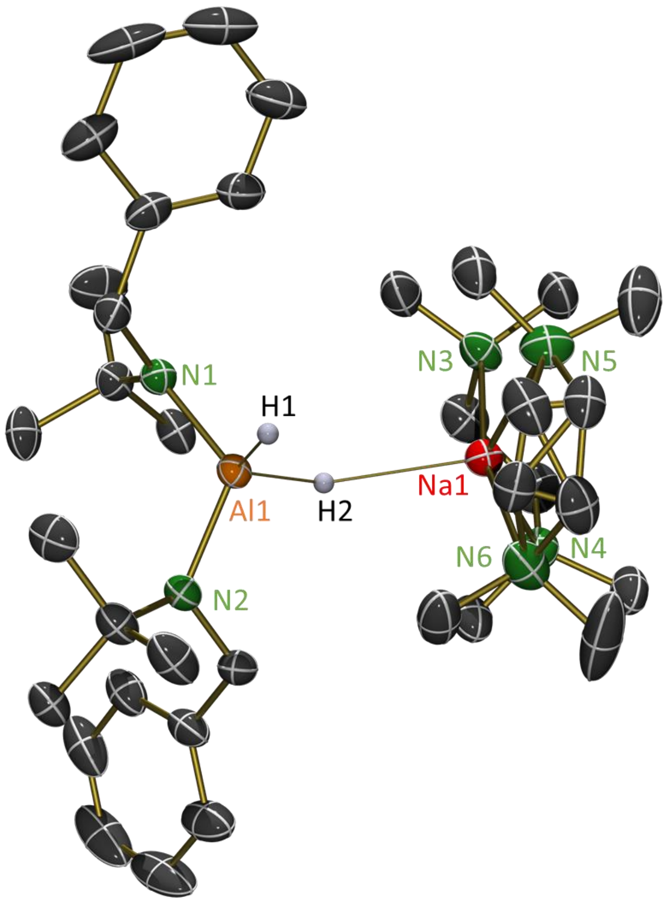

Figure S41: ORTEP-plot (50% probability) of $NaAlH_2[N(tBu)CH_2Ph]_2{\cdot}(TMEDA)_2$ (**2**). H atoms except for those bound to Al are omitted for clarity.

Structure determination of $NaAlH_2[N(tBu)CH_2Ph]_2{\cdot}PMDTA$ (3)

A colorless crystal of $[(pmdta)Na(H)_2Al(tBuNCH_2C_6H_5)_2]$ was embedded in inert perfluoropolyalkylether (viscosity 1800 cSt; ABCR GmbH) and mounted using a Hampton Research CryoLoop. The crystal was then

flash cooled to 100.0(2) K in a nitrogen gas stream and kept at this temperature during the experiment. The crystal structure was measured on a SuperNova diffractometer with Atlas S2 detector using a CuKα microfocus source. The measured data was processed with the CrysAlisPro (v40.53) software package.[S4] Using Olex2,[S5] the structure was solved with the ShelXT[S6] structure solution program using Intrinsic Phasing and refined with the ShelXL[S7] refinement package using Least Squares Minimization. All non-hydrogen atoms were refined anisotropically. Most hydrogen atoms were placed in ideal positions and refined as riding atoms with relative isotropic displacement parameters. The metal bound hydrides were observed from difference Fourier maps and refined.

Disorder of one benzyl group and of the pmdta ligand was observed. The disorder was modeled with the help of similarity restraints (SIMU, SADI) and rigid bond restraints (RIGU).[S8] The relative occupancies of the two alternative orientations of each group were refined to 0.60(2)/0.40(2) (benzyl) and 0.646(5)/0.354(5) (pmdta), respectively.

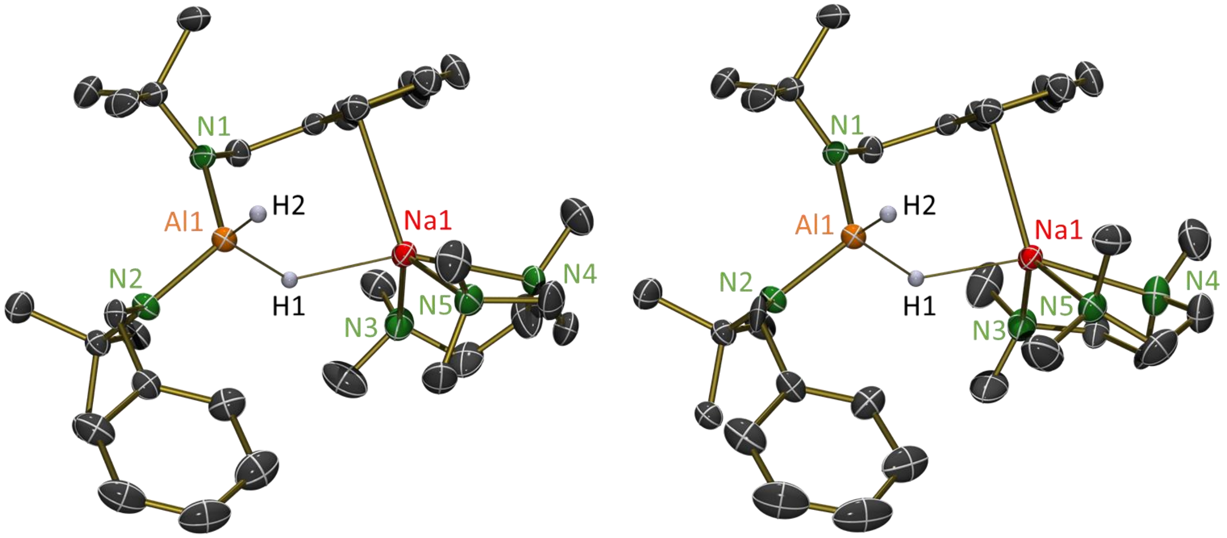

Figure S42: ORTEP-plot (50% probability) of $NaAlH_2[N(tBu)CH_2Ph]_2 \cdot PMDTA$ (**3**). H atoms except for those bound to Al are omitted for clarity. Due to disorder, the different orientations of the amide and PMDTA are shown left and right respectively.

Structure determination of $KAlH_2[N(tBu)CH_2Ph]_2 \cdot$18-crown-6 (4)

A colorless crystal of $[(18\text{-crown-6})K(H)_2Al(tBuNCH_2C_6H_5)_2]$ was embedded in inert perfluoropolyalkylether (viscosity 1800 cSt; ABCR GmbH) and mounted using a Hampton Research CryoLoop. The crystal was then flash cooled to 100.0(2) K in a nitrogen gas stream and kept at this temperature during the experiment. The crystal structure was measured on a SuperNova diffractometer with Atlas S2 detector using a CuKα microfocus source. The measured data was processed with the CrysAlisPro (v40.53) software package.[S4] Using Olex2,[S5] the structure was solved with the ShelXT[S6] structure solution program using Intrinsic Phasing and refined with the ShelXL[S7] refinement package using Least Squares Minimization. All non-hydrogen atoms were refined anisotropically. Most hydrogen atoms were placed in ideal positions and

refined as riding atoms with relative isotropic displacement parameters. The metal bound hydrides were observed from difference Fourier maps and refined.

Disorder of one benzyl group and of the whole K(18-crown-6) fragment was observed. The disorder was modeled with the help of similarity restraints (SIMU, SADI) and rigid bond restraints (RIGU).[S8] Additionally, a FLAT restraint was used for the aromatic ring of the less occupied orientation of the benzyl moiety. The relative occupancies of the two alternative orientations of the benzyl group were refined to 0.681(18) and 0.319(18), respectively. The disorder of the K(18-crown-6) fragment was more severe. While the potassium ion has two alternative positions with relative occupancies of 0.7986(14) and 0.2013(14), at least the 18-crown-6 ligand, which corresponds to the major potassium position, is additionally disordered (disorder within a disorder). Therefore, it was necessary to model the disorder of two sections of this 18-crown-6 as three alternative orientations. The related site occupancy factors are 0.460(3)/0.2013(14)/0.339(3) (section 1) and 0.455(3)/0.2013(14)/0.344(3) (section 2), respectively

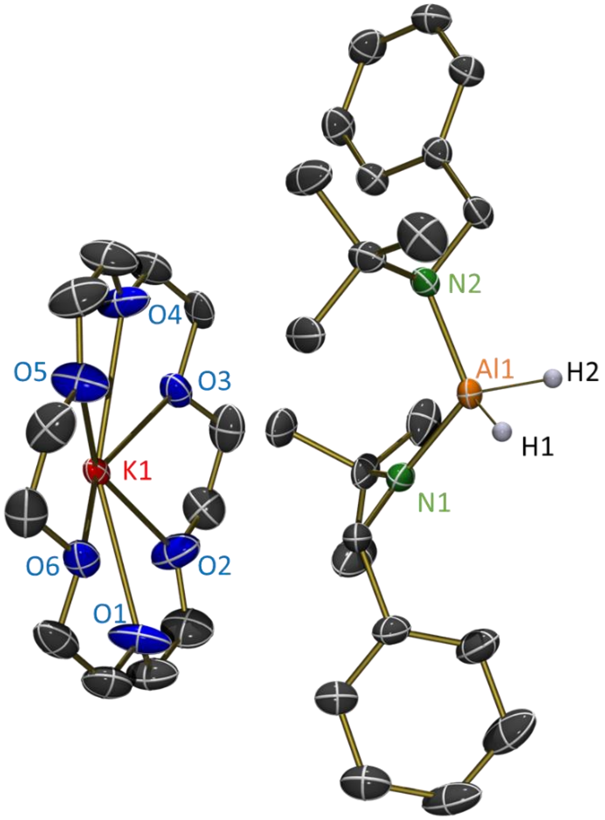

Figure S43: ORTEP-plot (50% probability) of $KAlH_2[N(tBu)CH_2Ph]_2$·18-crown-6 (**4**). H atoms except for those bound to Al are omitted for clarity. Disorder in the 18-crown-6 ligand and the phenyl ring of one amide omitted for clarity.

Structure Determination of $LiAlH_2[N(tBu)CH_2tBu]_2$·(TMEDA) (5)

A colorless crystal of $[(tmeda)Li(H)_2Al(tBuNCH_2tBu)_2]$ was embedded in inert perfluoropolyalkylether (viscosity 1800 cSt; ABCR GmbH) and mounted using a Hampton Research CryoLoop. The crystal was then flash cooled to 100.0(2) K in a nitrogen gas stream and kept at this temperature during the experiment. The crystal structure was measured on a SuperNova diffractometer with Atlas S2 detector using a CuKα microfocus source. The measured data was processed with the CrysAlisPro (v40.67a) software package.[S9] Using Olex2,[S5] the structure was solved with the ShelXT[S6] structure solution program using Intrinsic Phasing and refined with the ShelXL[S7] refinement package using Least Squares Minimization. Most hydrogen atoms were placed in ideal positions and refined as riding atoms with relative isotropic displacement parameters. The metal bound hydrides were observed from difference Fourier maps and refined.

Disorder of one $tBuNCH_2tBu$ ligand was observed and modeled with the help of similarity restraints (SIMU, SADI) and rigid bond restraints (RIGU).[S8] The relative occupancies of the two alternative orientations were refined to 0.787(2) and 0.213(2).

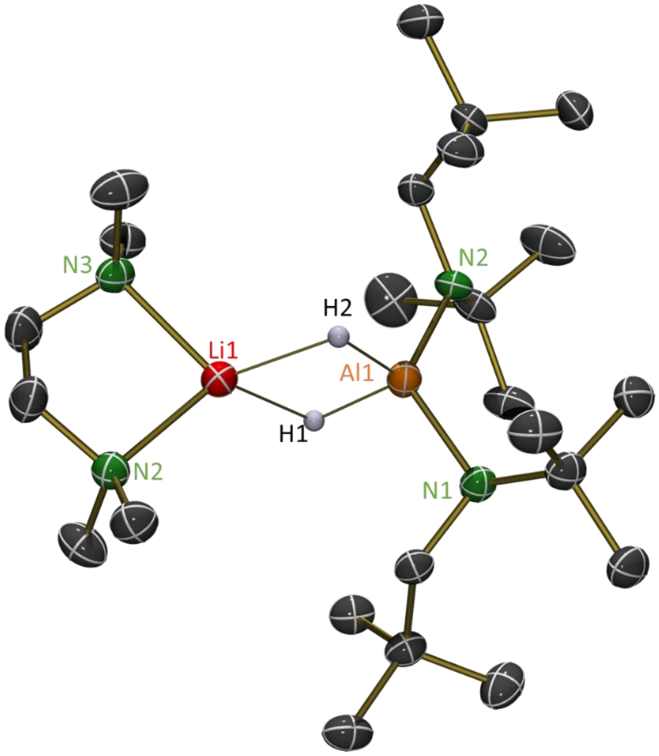

Figure S44: ORTEP-plot (50% probability) of $LiAlH_2[N(tBu)CH_2tBu]_2$·(TMEDA) (**5**). H atoms except for those bound to Al are omitted for clarity. Disorder in one amide ligand omitted for clarity.

Structure Determination of $LiAl[N(Ph)CH_2Ph]_4$·$(THF)_4$ (6)

A colorless crystal of $LiAl[N(Ph)CH_2Ph]_4$·$(THF)_4$ was embedded in inert perfluoropolyalkylether (viscosity 1800 cSt; ABCR GmbH) and mounted using a Hampton Research CryoLoop. The crystal was then flash cooled to 100.0(2) K in a nitrogen gas stream and kept at this temperature during the experiment. The

crystal structure was measured on a SuperNova diffractometer with Atlas S2 detector using a CuKα microfocus source. The measured data was processed with the CrysAlisPro (v40.53) software package.[S4] Using Olex2,[S5] the structure was solved with the ShelXT[S6] structure solution program using Intrinsic Phasing and refined with the ShelXL[S7] refinement package using Least Squares Minimization. All hydrogen atoms were placed in ideal positions and refined as riding atoms with relative isotropic displacement parameters. The crystal under investigation suffered from non-merohedral twinning. The fractional contributions of the two twin domains were refined to 0.6944(17) and 0.3056(17).

Both, the lithium and the aluminum atom reside on a crystallographic mirror plane, while the thf and amide ligands are disordered about this symmetry element. The disorder was modeled with the help of similarity restraints (SIMU, SADI) and an additional FLAT restraint to keep the carbon atoms C34 – C39 of a phenyl group in-plane.

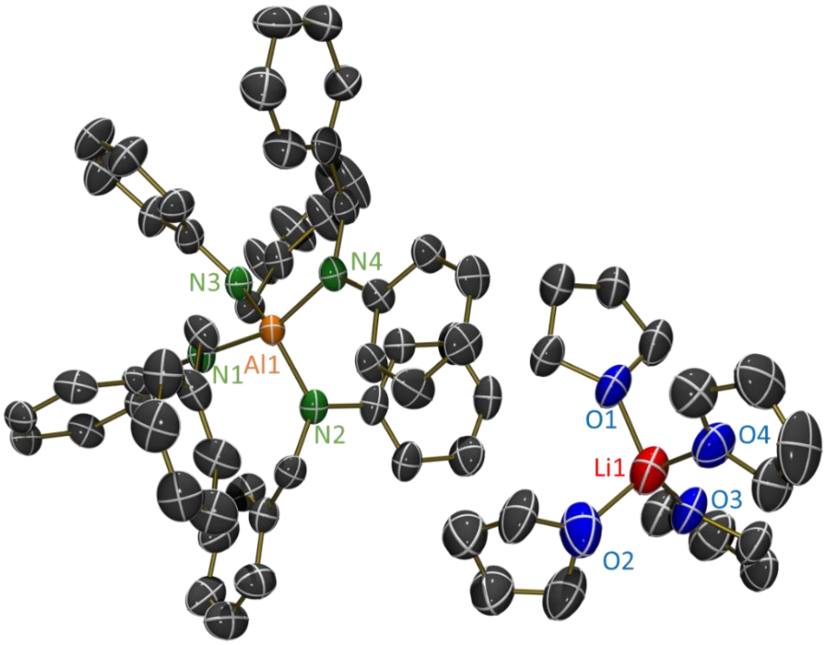

Figure S45: ORTEP-plot (50% probability) of $LiAl[N(Ph)CH_2Ph]_4 \cdot (THF)_4$ (**6**). H are omitted for clarity.

Structure Determination of 7

A colorless crystal of **7** was embedded in inert perfluoropolyalkylether (viscosity 1800 cSt; ABCR GmbH) and mounted using a Hampton Research CryoLoop. The crystal was then flash cooled to 100.0(1) K in a nitrogen gas stream and kept at this temperature during the experiment. The crystal structure was measured on a SuperNova diffractometer with Atlas S2 detector using a CuKα microfocus source. The measured data was processed with the CrysAlisPro (v39.46) software package.[S4] Using Olex2[S5], the structure was solved with the ShelXT [S6] structure solution program using Intrinsic Phasing and refined with the ShelXL [S7] refinement package using Least Squares minimization. Except of the NH hydrogen atom, all hydrogen atoms were placed in ideal positions and refined as riding atoms with relative isotropic displacement parameters. The position of the NH hydrogen atom was observed from difference Fourier

maps and refined. The crystal shows disorder concerning coordination of substrate/product (imine/amine). The disorder was refined over two positions (ratio ~ 50:50).

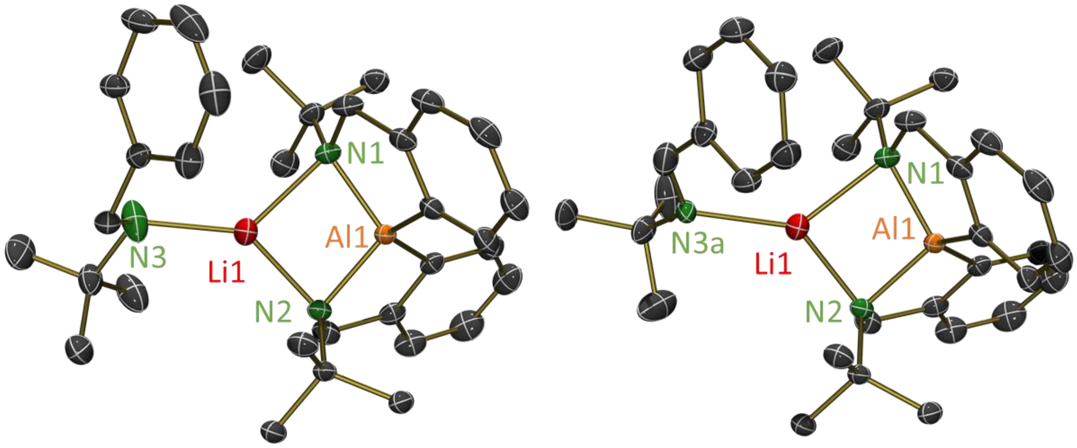

Figure S46: ORTEP-plot (50% probability) of **7**. H atoms except for those bound to Al are omitted for clarity. Due to disorder, the coordination of imine and amine are shown left and right respectively.

Structure determination of 8

A colorless crystal of $[Li(thf)_4][Al(tBuNCH_2C_6H_4)_2]$ was embedded in inert perfluoropolyalkylether (viscosity 1800 cSt; ABCR GmbH) and mounted using a Hampton Research CryoLoop. The crystal was then flash cooled to 100.0(3) K in a nitrogen gas stream and kept at this temperature during the experiment. The crystal structure was measured on a SuperNova diffractometer with Atlas S2 detector using a CuKα microfocus source. The measured data was processed with the CrysAlisPro (v40.53) software package.[S4] Using Olex2,[S5] the structure was solved with the ShelXT[S6] structure solution program using Intrinsic Phasing and refined with the ShelXL[S7] refinement package using Least Squares Minimization. All non-hydrogen atoms were refined anisotropically. All hydrogen atoms were placed in ideal positions and refined as riding atoms with relative isotropic displacement parameters. The crystal under investigation suffered from non-merohedral twinning. The fractional contributions of the two twin domains were refined to 0.6321(9) and 0.3679(9).

Additionally, disorder of one thf ligand was observed and modeled with the help of similarity restraints (SIMU, SADI). The relative occupancies of its two alternative orientations were refined to 0.79(2) and 0.21(2), respectively.

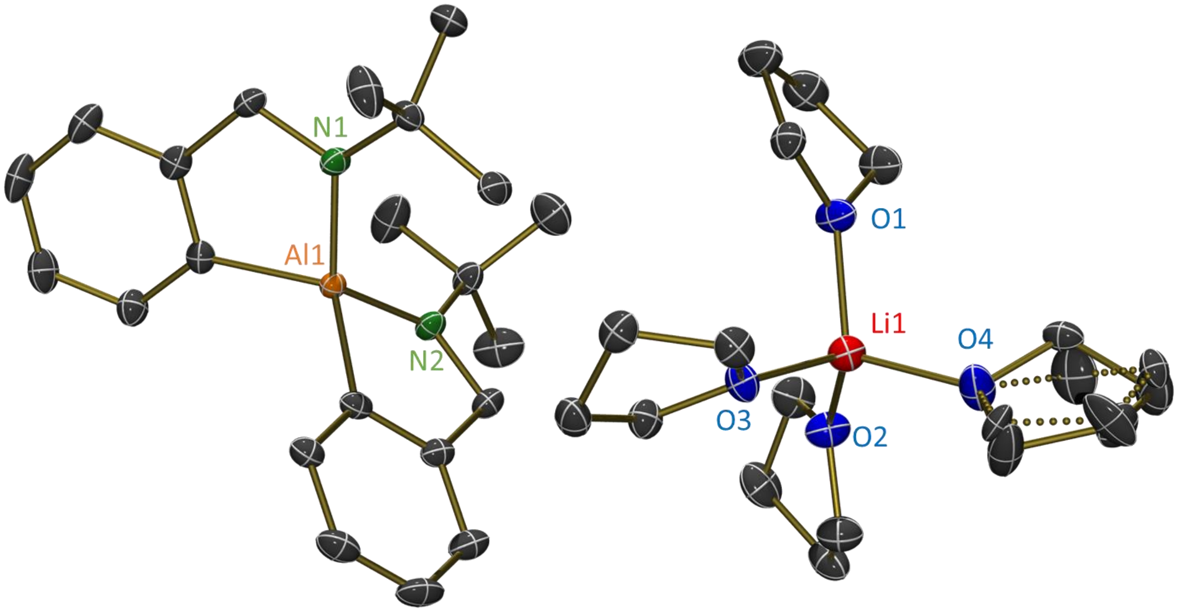

Figure S47: ORTEP-plot (50% probability) of **8**. H atoms are omitted for clarity.

Table S1: Crystal data.

Identification code (CCDC)	**1** (2024356)	**2** (2024357)	**3** (2024358)
Empirical formula	$C_{49}H_{81}AlLiN_3O_4$	$C_{34}H_{66}AlN_6Na$	$C_{31}H_{57}AlN_5Na$
Formula weight	810.08	608.89	549.78
Temperature/K	100.0(1)	100.00(11)	100.0(2)
Crystal system	orthorhombic	monoclinic	orthorhombic
Space group	Aea2	$P2_1/n$	Pbca
a/Å	28.8649(2)	11.4625(2)	13.0409(2)
b/Å	19.2621(2)	16.9597(2)	17.1247(3)
c/Å	17.41050(10)	20.1935(3)	30.7112(4)
α/°	90	90	90
β/°	90	95.190(2)	90
γ/°	90	90	90
Volume/Å^3	9680.21(13)	3909.53(10)	6858.45(19)
Z	8	4	8
ρ_{calc}g/cm^3	1.112	1.034	1.065
μ/mm^{-1}	0.696	0.767	0.822
F(000)	3552.0	1344.0	2416.0
Crystal size/mm^3	0.503 × 0.247 × 0.202	0.399 × 0.294 × 0.199	0.206 × 0.111 × 0.083
Radiation	Cu Kα (λ = 1.54184)	Cu Kα (λ = 1.54184)	Cu Kα (λ = 1.54184)
2Θ range for data collection/°	6.124 to 145.608	8.554 to 144.766	8.896 to 145.426
Index ranges	-35 ≤ h ≤ 35, -21 ≤ k ≤ 23, -17 ≤ l ≤ 21	-12 ≤ h ≤ 14, -20 ≤ k ≤ 20, -17 ≤ l ≤ 24	-15 ≤ h ≤ 14, -20 ≤ k ≤ 20, -37 ≤ l ≤ 31
Reflections collected	43938	21932	15730
Independent reflections	8559 [R_{int} = 0.0257, R_{sigma} = 0.0192]	7549 [R_{int} = 0.0273, R_{sigma} = 0.0253]	6630 [R_{int} = 0.0313, R_{sigma} = 0.0342]
Data/restraints/parameters	8559/1/536	7549/9/420	6630/399/522
Goodness-of-fit on F^2	1.027	1.086	1.022
Final R indexes [I>=2σ (I)]	R_1 = 0.0274, wR_2 = 0.0697	R_1 = 0.0591, wR_2 = 0.1557	R_1 = 0.0505, wR_2 = 0.1334
Final R indexes [all data]	R_1 = 0.0281, wR_2 = 0.0705	R_1 = 0.0653, wR_2 = 0.1614	R_1 = 0.0608, wR_2 = 0.1448
Largest diff. peak/hole / e Å^{-3}	0.20/-0.18	0.54/-0.28	0.46/-0.34

Table S1: Continued.

Identification code (CCDC)	**4** (2024359)	**5** (2024360)	**6** (2024361)
Empirical formula	$C_{34}H_{58}AlKN_2O_6$	$C_{24}H_{58}AlLiN_4$	$C_{68}H_{80}AlLiN_4O_4$
Formula weight	656.90	436.66	1051.28
Temperature/K	100.0(2)	100.0(1)	100.0(2)
Crystal system	orthorhombic	monoclinic	monoclinic
Space group	Pbca	$P2_1/c$	$P2_1/m$
a/Å	10.4344(3)	11.7729(3)	11.7199(6)
b/Å	16.9623(5)	11.0678(3)	19.4526(9)
c/Å	42.5886(9)	22.8213(6)	12.8802(8)
α/°	90	90	90
β/°	90	92.037(2)	95.899(5)
γ/°	90	90	90
Volume/Å^3	7537.8(3)	2971.74(13)	2920.9(3)
Z	8	4	2
ρ_{calc}g/cm^3	1.158	0.976	1.195
μ/mm^{-1}	1.792	0.688	0.705
F(000)	2848.0	984.0	1128.0
Crystal size/mm^3	0.194 × 0.094 × 0.075	0.32 × 0.171 × 0.143	0.168 × 0.159 × 0.061
Radiation	Cu Kα (λ = 1.54184)	Cu Kα (λ = 1.54184)	Cu Kα (λ = 1.54184)
2Θ range for data collection/°	8.304 to 145.424	7.514 to 145.692	7.584 to 144.734
Index ranges	-6 ≤ h ≤ 12, -18 ≤ k ≤ 20, -52 ≤ l ≤ 36	-14 ≤ h ≤ 13, -13 ≤ k ≤ 13, -26 ≤ l ≤ 28	-14 ≤ h ≤ 14, -23 ≤ k ≤ 23, -15 ≤ l ≤ 15
Reflections collected	17113	22800	9908
Independent reflections	7273 [R_{int} = 0.0287, R_{sigma} = 0.0331]	5811 [R_{int} = 0.0358, R_{sigma} = 0.0262]	9908 [R_{int} = ?, R_{sigma} = 0.0251]
Data/restraints/parameters	7273/3216/679	5811/159/392	9908/2597/695
Goodness-of-fit on F^2	1.020	1.028	1.043
Final R indexes [I>=2σ (I)]	R_1 = 0.0574, wR_2 = 0.1386	R_1 = 0.0446, wR_2 = 0.1162	R_1 = 0.0848, wR_2 = 0.2308
Final R indexes [all data]	R_1 = 0.0691, wR_2 = 0.1477	R_1 = 0.0492, wR_2 = 0.1215	R_1 = 0.1004, wR_2 = 0.2388
Largest diff. peak/hole / e Å^{-3}	0.64/-0.39	0.60/-0.73	0.37/-0.36

Table S1: Continued.

Identification code (CCDC)	**7** (2024362)	**8** (2024363)
Empirical formula	$C_{33}H_{46}AlLiN_3$	$C_{38}H_{62}AlLiN_2O_4$
Formula weight	518.66	644.81
Temperature/K	100	100.0(3)
Crystal system	Monoclinic	monoclinic
Space group	$P2_1/n$	$P2_1$
a/Å	10.59708(6)	10.7366(11)
b/Å	17.20443(9)	16.7030(13)
c/Å	16.60664(10)	11.1891(11)
α/°	90	90
β/°	90.7848(6)	109.155(11)
γ/°	90	90
Volume/Å^3	3027.39(3)	1895.5(3)
Z	4	2
ρ_{calc}g/cm^3	1.138	1.130
μ/mm^{-1}	0.759	0.764
F(000)	1124.0	704.0
Crystal size/mm^3	0.576 × 0.435 × 0.353	0.171 × 0.147 × 0.083
Radiation	CuKα (λ = 1.54184)	Cu Kα (λ = 1.54184)
2Θ range for data collection/°	7.4 to 145.868	8.366 to 145.286
Index ranges	-13 ≤ h ≤ 13, -21 ≤ k ≤ 21, -20 ≤ l ≤ 20	-13 ≤ h ≤ 13, -20 ≤ k ≤ 20, -13 ≤ l ≤ 13
Reflections collected	55738	13786
Independent reflections	5994 [R_{int} = 0.0369, R_{sigma} = 0.0148]	13786 [R_{int} = ?, R_{sigma} = 0.0222]
Data/restraints/parameters	5994/0/459	13786/230/459
Goodness-of-fit on F^2	1.045	1.019
Final R indexes [I>=2σ (I)]	R_1 = 0.0352, wR_2 = 0.0920	R_1 = 0.0412, wR_2 = 0.1081
Final R indexes [all data]	R_1 = 0.0367, wR_2 = 0.0932	R_1 = 0.0431, wR_2 = 0.1092
Largest diff. peak/hole / e Å^{-3}	0.24/-0.25	0.28/-0.28

Calculations

Computational Details

All calculations were carried out using Gaussian 16A.[S10] All structures were fully optimized on a B3PW91/6-31+G** level of theory.[S11-S15] Harmonic frequency calculations were carried out on the same level of theory to characterize the structures as minima (NIMAG=0) or transition states (NIMAG=1). For higher accuracy, single point energies were determined on B3PW91/6-311++G**//6-31+G** level of theory. Solvent effects were modeled via polarizable continuum model (PCM) simulating THF (ε = 7.4257).[S16] In all energy profiles ΔH is given in kcal/mol.

Effect of solvation

Initial calculations were carried out using a simple model system with imine MeC(H)=NMe and $LiAlH_4$·TMEDA as catalyst. This allowed for the identification of stationary point geometries and the evaluation of solvent effects. TMEDA was chosen as explicit solvent as experimental results found that it does not have a negative effect on catalytic performance. It also provides a more realistic solvation effect than continuum based solvent models like PCM.

Imine to amide reduction was found to have activation barriers of **M1**-**M2***: 18.1, **M7**-**M8***: 12.1, **M10**-**M11***: 11.0 kcal/mol. Imine insertion into the metal hydride bond becomes easier with an increasing number of amide ligands present. Four imine insertions, however, are not possible. The rate determining step is generally the hydrogenolysis step with **M4**-**M5***: 31.2 and **M13**-**M14***: 32.2 kcal/mol. These energies are slightly too high for a reaction at 85 °C. This may be partially attributed to the fact that imines with alkyl substituents on C and N are difficult to convert.

Removal of TMEDA (energy levels **M1'**-**M2'*** and **M10'**-**M11'*** marked in red) shows an increase for the imine insertion barriers. Without the explicit solvent molecule coordinated to lithium, the activation energies for these two steps increased from 18.1 to 20.0 and from 11.0 to 24.6 kcal/mol, respectively. This is an effective increase in activation barrier of 18 and 55 %. It shows that, for strongly ionic species such as $LiAlH_4$, it is very important to model solvation explicitly with solvent molecules (or in this case a polar imine). For further calculations (Mechanisms A-D) imine PhC(H)=NtBu was chosen as both substrate and coordinated solvent molecule (the majority of catalytic experiments were carried out in neat imine without additional solvent).

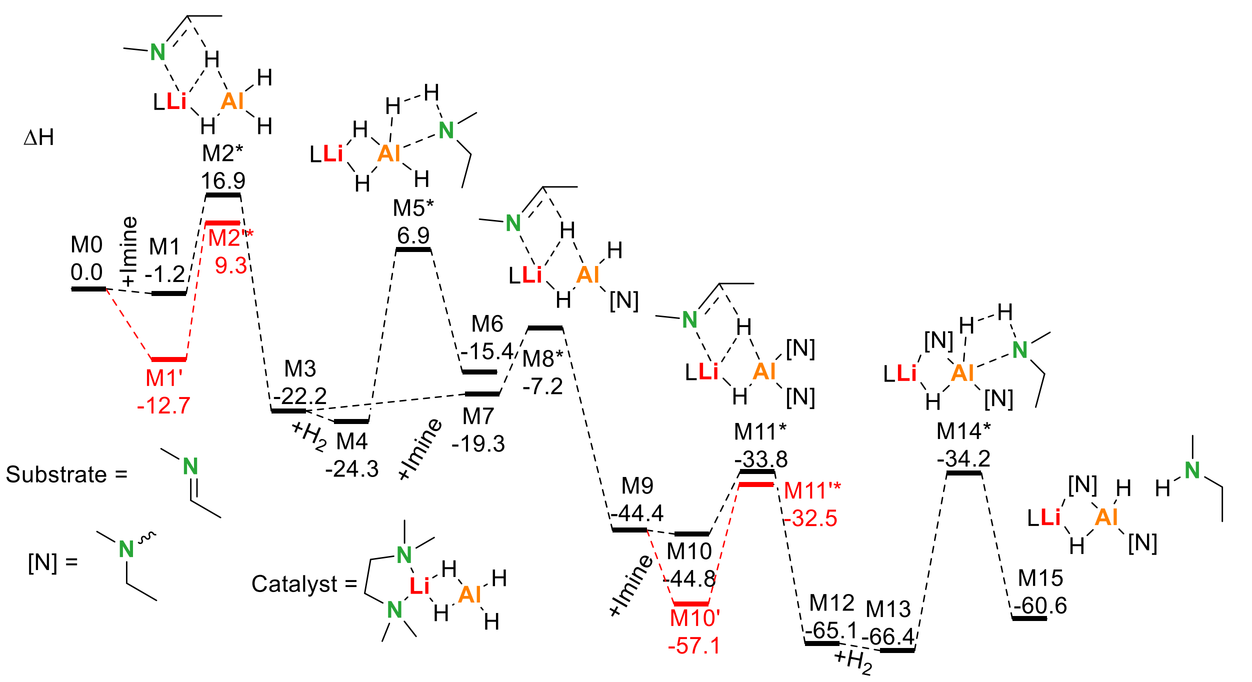

Scheme S1: Energy profile (B3PW91/6-311++G**(PCM=THF)//6-31+G**) for the hydrogenation of MeC(H)=NMe with the catalyst $LiAlH_4$·TMEDA (L = TMEDA, [N] = N(Me)Et). The energy levels in red (**M1'**, **M2'***, **M10'** and **M11'***) represent optimized structures without the TMEDA ligand.

Thermodynamics of sequential imine insertion in $LiAlH_4$

The reaction energies for insertion of different imines in the Al-H bonds of $LiAlH_4$ have been calculated. It can be concluded that *t*BuC(H)=N*t*Bu can realistically insert twice and PhC(H)=N*t*Bu three times. More insertions are endothermic and endergonic. After four imine insertions the complex dissociates in Al amide and Li amide species. The highly activated imine, PhC(H)=NPh, can insert four times and the resulting complex is stable die to Ph···Li interactions.

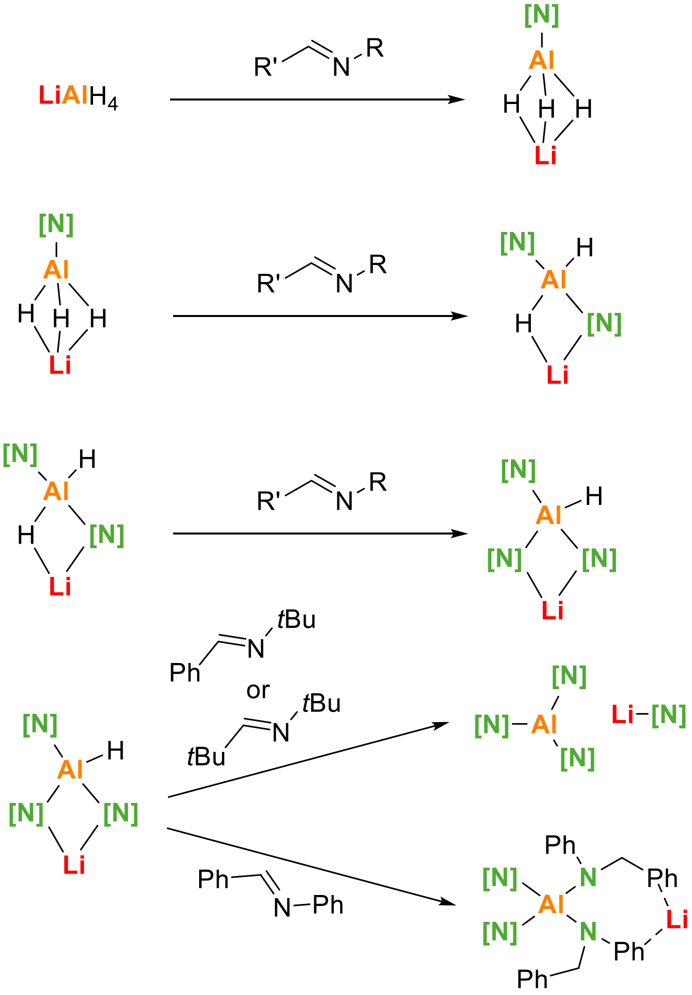

	ΔH	ΔG
R=tBu, R'=Ph	-25.1*	-7.0
R=R' = *t*Bu	-15.1	-1.6
R=R' = Ph	-23.9	-13.3
R=tBu, R'=Ph	-21.1*	-0.1
R=R' = *t*Bu	-6.5	+10.1
R=R' = Ph	-32.3	-17.2
R=tBu, R'=Ph	-17.3*	+12.0
R=R' = *t*Bu	+20.4	+39.5
R=R' = Ph	-23.2	-9.8
R=*t*Bu, R'=Ph	+15.0*	n.d.
R=R' = *t*Bu	-0.1	+9.4
R=R' = Ph	-9.4	+9.8

Scheme S2: Reaction energy for the reaction of $LiAlH_4$ with one, two, three, and four equivalents of PhC(H)=N*t*Bu, *t*BuC(H)=N*t*Bu, and Ph(H)C=NPh. ΔH and ΔG (85 °C, 6 bar, PCM correction for the polar solvent THF) given in kcal/mol. *For PhC(H)=NtBu only ΔE was calculated instead of ΔH.[S17]

Energy profiles with DG values (kcal/mol)

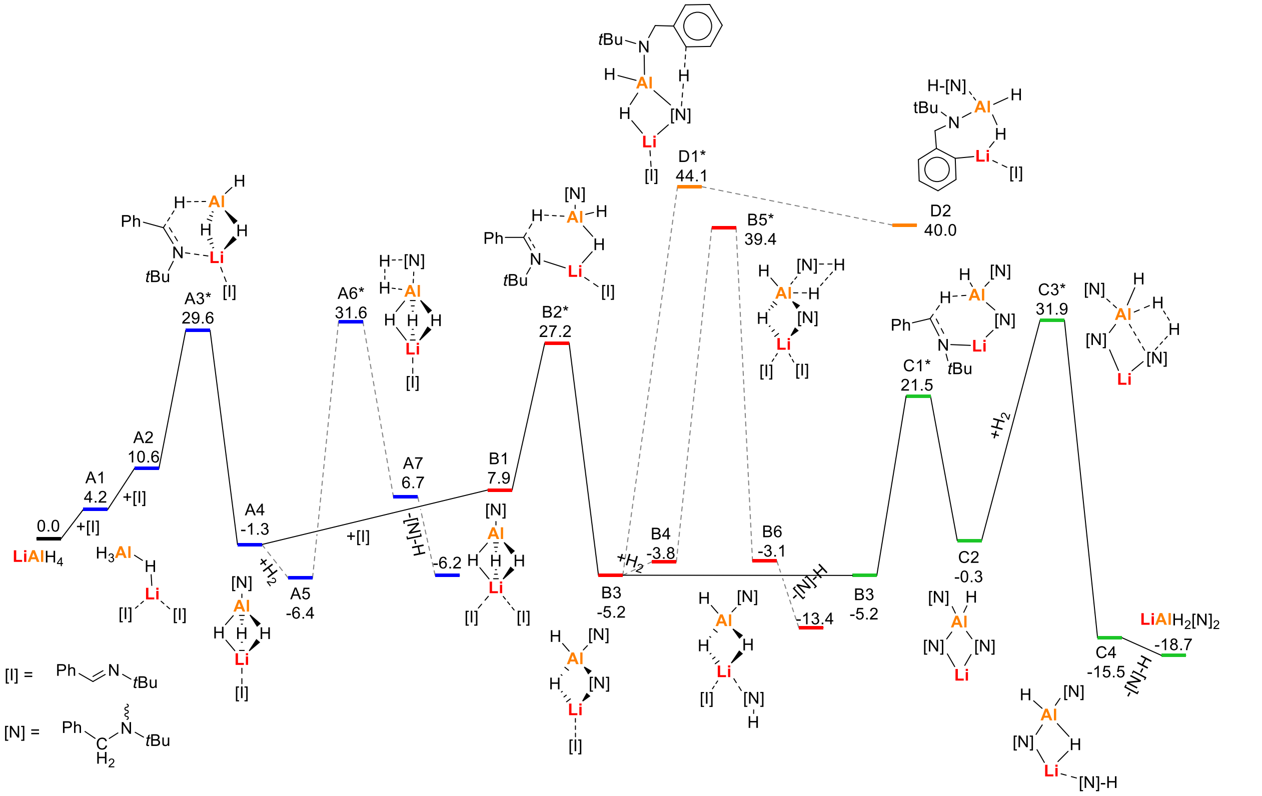

Scheme S3: Full energy profile (ΔG (358 K, 6 bar)) for the hydrogenation of PhC(H)=N*t*Bu with $LiAlH_4$ using the imine as explicit solvent (B3PW91/6-311++G**(PCM=THF)//6-31+G**). Pathways A (blue) and B (red) show the formation of the active catalyst: $LiAlH_2[N]_2$ (B3). Pathway C (green) represents the catalytic cycle for **B3**. is the catalytic cycle. Path D (grey) shows a pathway for catalyst decomposition by ortho-metalation. The most likely intermediates are connected by solid lines.

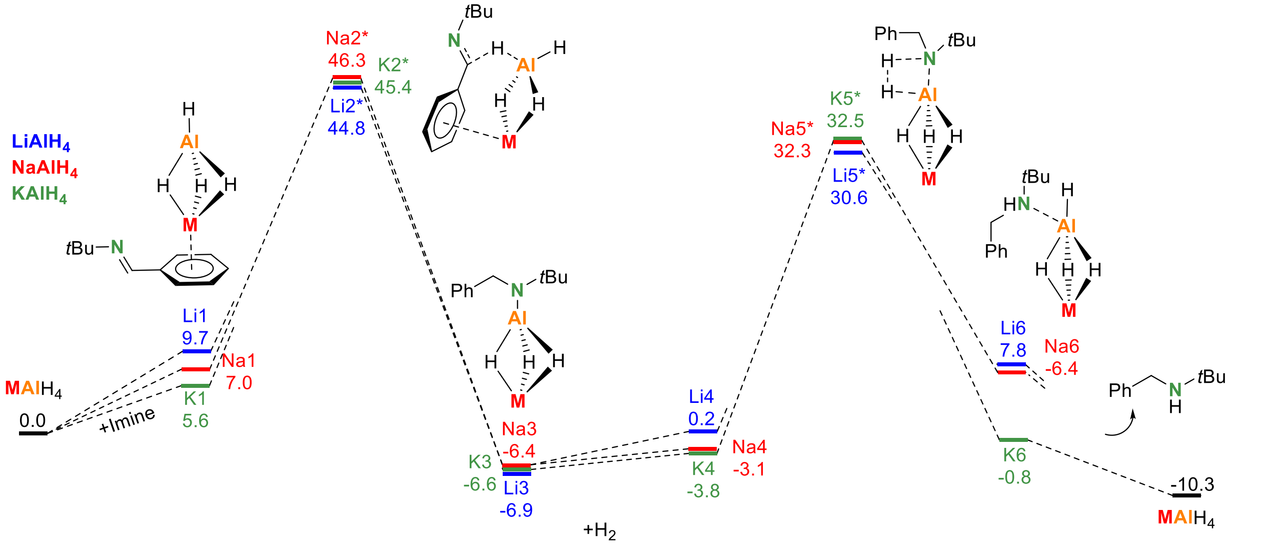

Scheme S4: Comparison of the metal influence on hydrogenation of imines with $MAlH_4$ catalysts (M = Li, Na, K) (ΔG (358 K, 6 bar)); B3PW91/6-311++G**(PCM=THF)//6-31+G**.

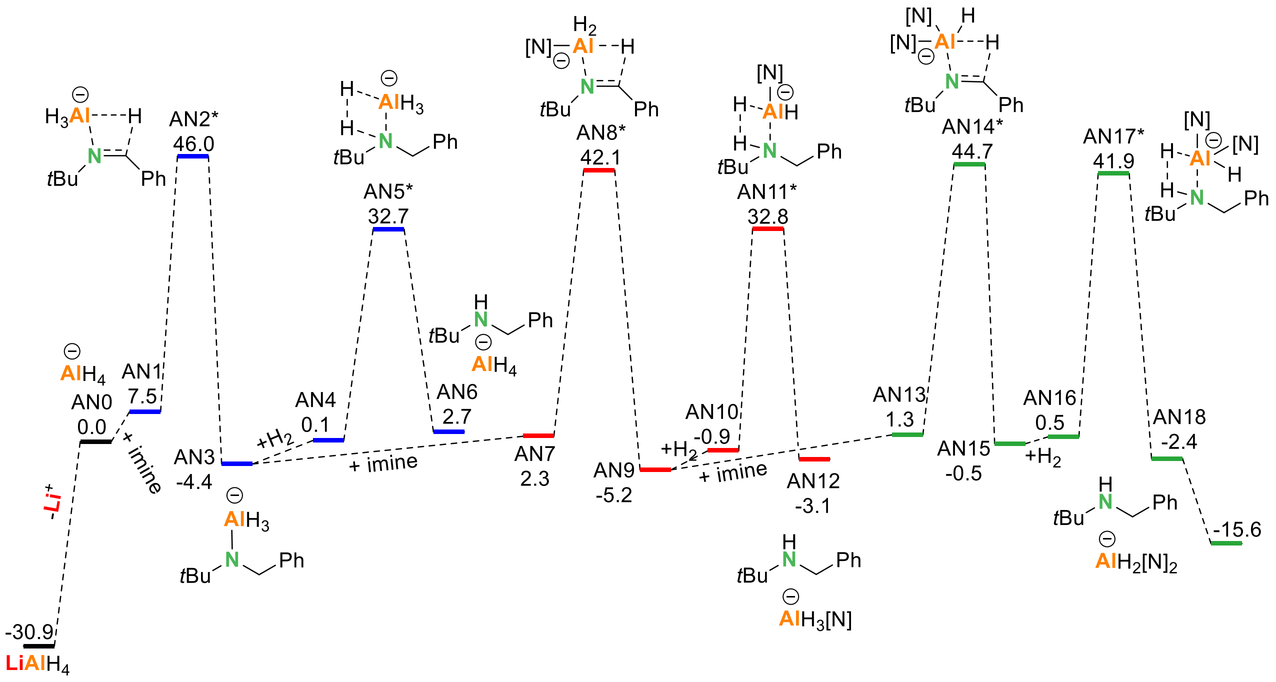

Scheme S5: Full energy profile (ΔG (358 K, 6 bar)) for the hydrogenation of PhC(H)=N*t*Bu with the anion AlH_4^- (B3PW91/6-311++G**(PCM=THF)//6-31+G**).

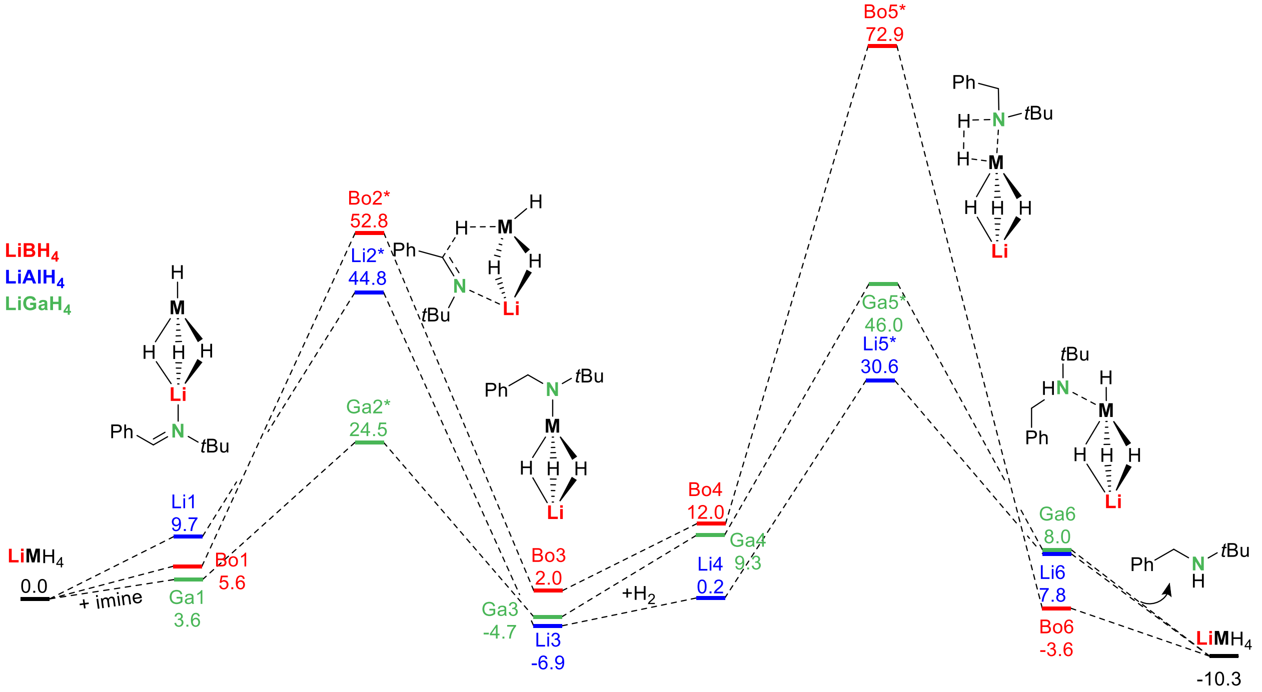

Scheme S6: Comparison of the (half)metal influence on hydrogenation of imines with LiMH4 catalysts (M = B, Al, Ga); (ΔG (358 K, 6 bar)) B3PW91/6-311++G**(PCM=THF)//6-31+G**.

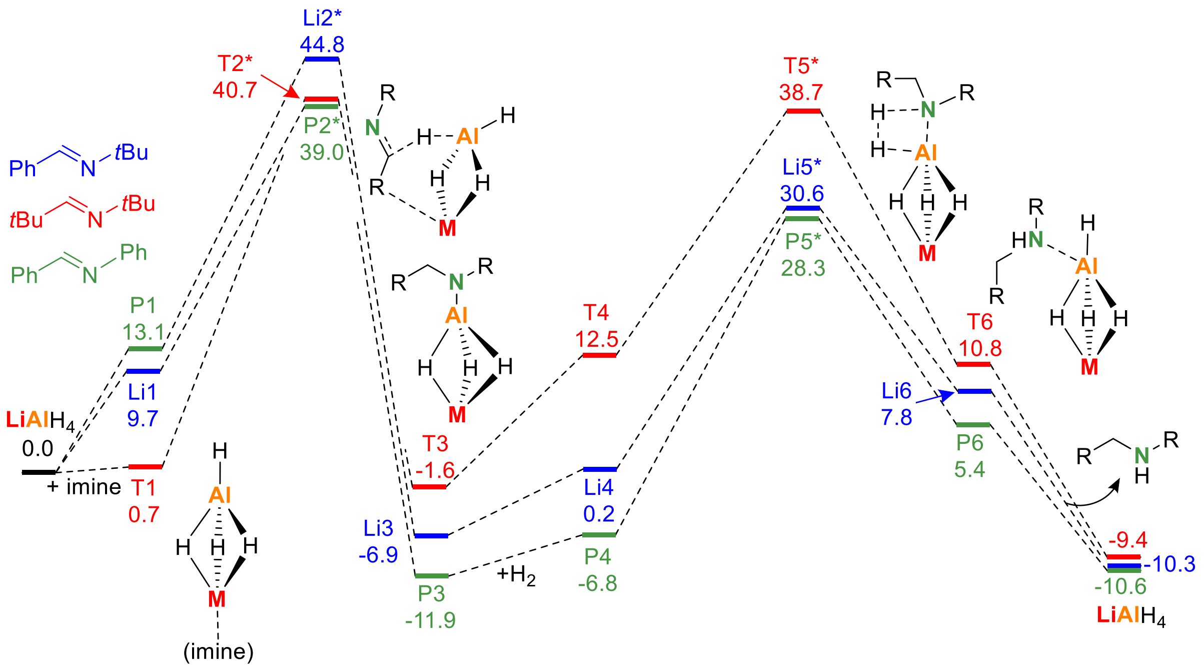

Scheme S7: Comparison of imine substituent effects in hydrogenation with a LiAlH4 catalyst; (ΔG (358 K, 6 bar)) B3PW91/6-311++G**(PCM=THF)//6-31+G**.

XYZ Coordinates for Mechanisms A, B, C, D

6

LiAlH4

Al	-0.394792	0.000000	-0.000009
H	0.498692	1.231531	-0.678644
H	0.498691	-1.203310	-0.727513
H	-1.978527	-0.000010	0.000611
H	0.499419	-0.028214	1.405391
Li	1.871340	0.000000	0.000092

2

H2

H	0.000000	0.000000	0.371710
H	0.000000	0.000000	-0.371710

27

Imine

C	0.238296	-0.492128	0.000000
N	1.123037	0.422695	-0.000001
C	-1.205667	-0.201036	0.000001
C	-1.688438	1.116783	0.000000
C	-3.057023	1.360276	0.000000
C	-3.963550	0.294652	0.000000
C	-2.119865	-1.262532	0.000000
C	-3.492686	-1.017798	0.000000
H	-0.969761	1.930599	0.000000
H	-3.423924	2.383212	0.000000
H	-5.032606	0.489478	-0.000001
H	-1.751135	-2.286401	0.000001
H	-4.192281	-1.849111	0.000000
H	0.488384	-1.566114	0.000001
C	2.554597	0.058791	0.000000
C	3.334942	1.378130	-0.000001
C	2.904528	-0.744658	1.262799
C	2.904528	-0.744660	-1.262798
H	3.981902	-0.937596	-1.305348
H	2.390571	-1.711465	-1.283267
H	2.620434	-0.187844	-2.161743
H	3.981902	-0.937594	1.305349
H	2.620435	-0.187841	2.161742
H	2.390571	-1.711463	1.283269
H	4.415464	1.197111	-0.000001
H	3.080405	1.970618	-0.884449
H	3.080405	1.970619	0.884447

33

A1

Al	1.372813	3.735635	-0.190778
H	0.487368	3.014431	-1.393634
H	0.588023	2.999887	1.071548
H	1.614142	5.307810	-0.189123
H	2.651705	2.682616	-0.254138
Li	1.002075	1.428164	-0.241676
C	-0.172758	-1.262026	0.048535
N	0.901521	-0.567136	0.024512
C	-1.529067	-0.696700	0.064731
C	-2.589040	-1.521374	-0.343872
C	-3.893373	-1.035758	-0.379841
C	-4.155881	0.277380	0.011855
C	-1.807420	0.615913	0.477517
C	-3.112014	1.097744	0.445923
H	-2.384206	-2.545726	-0.647280
H	-4.703064	-1.680520	-0.709041
H	-5.172845	0.658705	-0.010640
H	-1.020475	1.261697	0.860939
H	-3.313870	2.114561	0.769122
H	-0.136994	-2.356444	0.041906
C	2.235479	-1.214745	0.041520
C	2.962132	-0.658213	1.277073
C	2.211303	-2.744699	0.102175
C	2.958703	-0.759833	-1.236845
H	3.975326	-1.165034	-1.257190
H	2.431624	-1.109740	-2.130509
H	3.041951	0.331955	-1.285493
H	3.240407	-3.114477	0.124607
H	1.712726	-3.114743	1.004932
H	1.726550	-3.187535	-0.775107
H	3.986347	-1.042963	1.315220
H	3.015535	0.435153	1.247512
H	2.447926	-0.953558	2.197303

60

A2

Li	-0.012358	-0.027706	0.516108
C	2.157252	1.920446	-0.426117
N	0.904612	1.745425	-0.227547
C	3.205752	0.956216	-0.065819
C	3.046756	0.021673	0.967968
C	4.069030	-0.873488	1.269141
C	5.263950	-0.841916	0.547149
C	4.421858	1.000106	-0.766556
C	5.440553	0.098217	-0.470348
H	2.145423	0.011922	1.577376
H	3.921664	-1.582622	2.078564
H	6.062315	-1.539100	0.786376
H	4.562675	1.738153	-1.553490
H	6.373567	0.133459	-1.025806
H	2.530402	2.833750	-0.901331
C	-0.054533	2.828145	-0.559673
C	-0.768741	3.179719	0.755436
C	0.567347	4.098423	-1.151363
C	-1.056658	2.244416	-1.565787
H	-1.831189	2.981997	-1.798649
H	-0.554846	1.965439	-2.498330
H	-1.557137	1.357626	-1.166339
H	-0.228875	4.824129	-1.341572
H	1.279083	4.570093	-0.465166
H	1.070560	3.910643	-2.106638
H	-1.557029	3.916899	0.570676
H	-1.225937	2.296870	1.211791
H	-0.063099	3.598037	1.479704
Al	0.099919	-1.078685	3.469661
H	1.469172	-1.855488	3.039079
H	-1.218284	-2.012056	3.290248
H	-0.071907	0.097314	2.277373
H	0.198485	-0.283134	4.870222
C	-2.187486	-1.610857	-0.891594
N	-0.936948	-1.559499	-0.624513
C	-3.206982	-0.752244	-0.268585
C	-3.092380	-0.305421	1.056208
C	-4.088605	0.497676	1.607635
C	-5.202479	0.861115	0.848775
C	-4.348094	-0.414155	-1.013623
C	-5.333266	0.401290	-0.463536
H	-2.257646	-0.632500	1.673467
H	-3.999613	0.822468	2.640324
H	-5.977706	1.485212	1.284937
H	-4.455628	-0.783990	-2.031191
H	-6.206671	0.668669	-1.051690
H	-2.579131	-2.319167	-1.629117
C	-0.007403	-2.540144	-1.240768
C	0.611902	-3.326312	-0.074536
C	-0.651920	-3.520300	-2.227691
C	1.074446	-1.732675	-1.971505
H	1.824738	-2.406853	-2.396432
H	0.640241	-1.143659	-2.786403
H	1.596815	-1.051269	-1.293606
H	0.122048	-4.191754	-2.610840
H	-1.415614	-4.145532	-1.752715
H	-1.097951	-3.011819	-3.090307
H	1.380964	-4.009357	-0.450575
H	1.073844	-2.666316	0.666059
H	-0.151045	-3.913104	0.445637

60

A3*

Al	-0.807755	-1.677967	-2.539407
H	-1.721293	-1.602328	-3.854075
H	0.427943	-2.707529	-2.494869
H	-2.546404	-2.578241	0.423066
H	-0.361581	-0.213920	-1.943833
Li	-0.006498	0.165261	-0.095988
C	-2.077320	-1.617270	0.158479
N	-1.008466	-1.219497	0.861114
C	-3.122737	-0.580914	-0.187633
C	-3.318623	0.502321	0.676567
C	-4.332855	1.432757	0.439187
C	-5.172155	1.287459	-0.663660
C	-3.976071	-0.720093	-1.289830
C	-4.991437	0.203017	-1.525745
H	-2.679466	0.583865	1.550459
H	-4.471671	2.265479	1.124266
H	-5.962715	2.009260	-0.850904
H	-3.829923	-1.548642	-1.978606
H	-5.638340	0.080880	-2.390326
H	-1.785217	-2.103667	-1.151422
C	-0.356331	-2.230148	1.718492
C	0.880022	-1.561254	2.331070
C	-1.294896	-2.655700	2.863521
C	0.090452	-3.472547	0.927784
H	0.623810	-4.174625	1.579257
H	-0.761700	-4.008402	0.495700
H	0.755827	-3.194215	0.104252
H	-0.799886	-3.369105	3.532910
H	-1.597622	-1.782845	3.451876
H	-2.202797	-3.137942	2.484807
H	1.389325	-2.232999	3.030833
H	1.603870	-1.285159	1.554766
H	0.592983	-0.654113	2.874721
C	2.577495	1.775376	-0.033700
N	1.300081	1.791627	0.040680
C	3.384853	0.599663	-0.387526
C	2.873492	-0.484798	-1.115831
C	3.683148	-1.576554	-1.413097
C	5.014699	-1.598290	-0.991645
C	4.731935	0.580402	0.006727
C	5.539547	-0.516357	-0.283298
H	1.853379	-0.463854	-1.489765
H	3.269789	-2.404474	-1.981485
H	5.644603	-2.452078	-1.225350
H	5.142900	1.425922	0.554199
H	6.577269	-0.524865	0.037668
H	3.165437	2.673078	0.185363
C	0.587816	3.047780	0.381304
C	-0.069497	2.822471	1.752223
C	1.465869	4.302854	0.438558
C	-0.488988	3.232997	-0.699401
H	-1.091585	4.121296	-0.484442
H	-0.032144	3.355171	-1.686346
H	-1.170000	2.376990	-0.746811
H	0.830765	5.169557	0.644035
H	2.213806	4.256638	1.237646
H	1.977630	4.488224	-0.512340
H	-0.700461	3.677997	2.013998
H	-0.698170	1.926754	1.751727
H	0.690113	2.701747	2.531450

60

A4

Al	-0.666451	-0.022095	0.660733
H	0.595803	-0.572778	1.601379
H	-0.154308	1.561760	0.658433
H	-3.628121	-2.100036	0.191637
H	-0.001818	-0.432686	-0.796171
Li	1.524607	0.552954	0.168621
C	-3.081747	-1.218855	-0.180700
N	-2.410488	-0.516507	0.898951
C	-4.037269	-0.406725	-1.051735
C	-3.880588	0.970696	-1.228298
C	-4.723693	1.683228	-2.081928
C	-5.741771	1.028034	-2.775761
C	-5.062279	-1.053821	-1.752350
C	-5.908565	-0.347380	-2.606991
H	-3.096584	1.482627	-0.676552
H	-4.586076	2.755006	-2.203890
H	-6.401094	1.583240	-3.437783
H	-5.202657	-2.126240	-1.623530
H	-6.701986	-0.869407	-3.136348
H	-2.318186	-1.628704	-0.859443
C	-3.086276	-0.494873	2.209875
C	-2.343685	0.506217	3.104119
C	-4.554099	-0.046415	2.102542
C	-3.028016	-1.884727	2.877857
H	-3.522977	-1.878171	3.856618
H	-3.525800	-2.643966	2.264794
H	-1.986516	-2.193630	3.018117
H	-5.011704	-0.012085	3.098172
H	-4.626311	0.948276	1.652877
H	-5.147974	-0.732422	1.490629
H	-2.790291	0.542268	4.103663
H	-1.290532	0.220832	3.232765
H	-2.381503	1.513484	2.675227
C	4.438877	0.471706	-0.324986
N	3.427986	1.191342	-0.012013
C	4.419364	-0.992930	-0.441662
C	3.434387	-1.799171	0.150473
C	3.465202	-3.179572	-0.018174
C	4.479146	-3.775364	-0.771951
C	5.448299	-1.604230	-1.175447
C	5.474268	-2.985471	-1.348323
H	2.656484	-1.366321	0.775746
H	2.698310	-3.791720	0.446956
H	4.498063	-4.853901	-0.900612
H	6.225384	-0.988294	-1.622765
H	6.269923	-3.444519	-1.927871
H	5.408565	0.929451	-0.546734
C	3.545866	2.664999	0.109874
C	3.090760	3.011047	1.537219
C	4.950507	3.224785	-0.132902
C	2.566296	3.268408	-0.910224
H	2.567495	4.360161	-0.830957

H	2.849931	2.997712	-1.932493
H	1.539618	2.928911	-0.731658
H	4.926640	4.310185	-0.000125
H	5.682940	2.825653	0.577565
H	5.305057	3.030742	-1.151353
H	3.089046	4.096684	1.678245
H	2.075789	2.647269	1.729570
H	3.763039	2.567628	2.278781

62

A5

Al	0.908979	0.406382	0.908529
H	-0.553609	-0.323943	1.357745
H	0.167769	1.229564	-0.366937
H	3.921450	-1.540442	1.497185
H	1.363805	1.453059	2.034148
Li	-1.489940	0.636987	0.103063
C	3.533559	-0.575779	1.131474
N	2.273776	-0.736579	0.425120
C	4.664369	0.132817	0.389484
C	4.409796	1.075197	-0.610769
C	5.454422	1.762872	-1.229608
C	6.777209	1.518509	-0.858220
C	5.995274	-0.103888	0.754227
C	7.044372	0.579289	0.139083
H	3.379920	1.253817	-0.908387
H	5.234557	2.491742	-2.006226
H	7.591517	2.051252	-1.342393
H	6.213544	-0.837384	1.529361
H	8.070652	0.374789	0.434586
H	3.347775	0.015919	2.040442
C	2.125499	-1.966607	-0.372656
C	0.886030	-1.821590	-1.265791
C	3.334904	-2.224199	-1.290235
C	1.921521	-3.191811	0.545166
H	1.801677	-4.114468	-0.036494
H	2.777221	-3.335010	1.213745
H	1.030057	-3.052695	1.166444
H	3.170867	-3.131154	-1.884262
H	3.491892	-1.384936	-1.974234
H	4.258647	-2.366174	-0.721324
H	0.740720	-2.719323	-1.876868
H	-0.019251	-1.692517	-0.658093
H	0.988614	-0.960164	-1.933830
H	-0.314358	1.721730	5.514775
H	-0.712799	1.842191	6.131512
C	-4.434289	0.790710	-0.175330
N	-3.335307	1.435026	-0.056815
C	-4.553506	-0.671413	-0.076718
C	-3.615684	-1.470538	0.596180
C	-3.773243	-2.852356	0.636980
C	-4.867950	-3.454834	0.012397
C	-5.665047	-1.284175	-0.675643
C	-5.816827	-2.667987	-0.640957
H	-2.779076	-1.020159	1.126897
H	-3.044583	-3.459012	1.166470
H	-4.986684	-4.534048	0.046909
H	-6.407169	-0.670706	-1.181670
H	-6.675369	-3.130939	-1.118648
H	-5.375083	1.311765	-0.380932
C	-3.301328	2.916088	-0.148096
C	-2.555781	3.400120	1.106070
C	-2.487252	3.261680	-1.405878
C	-4.675458	3.588392	-0.220205
H	-4.538466	4.673411	-0.233355
H	-5.224112	3.323131	-1.130668
H	-5.296722	3.346339	0.649341
H	-2.366485	4.346607	-1.486613
H	-1.486342	2.817605	-1.369287
H	-2.993629	2.903954	-2.308523
H	-2.447182	4.488946	1.082520
H	-3.100934	3.128123	2.015436
H	-1.548565	2.973092	1.167294

62

A6*

Al	0.720847	-1.123705	0.199701
H	-0.400879	-0.086911	-0.620778
H	-0.472210	-1.444154	1.344029
H	3.057669	0.761504	-1.618955
H	0.941272	-2.331081	-0.834500
Li	-1.785766	-0.734944	0.311705
C	3.184095	-0.073277	-0.916606
N	2.313657	0.096298	0.256603
C	4.667199	-0.279672	-0.657745
C	5.118347	-1.141607	0.349157
C	6.481255	-1.372435	0.531021
C	7.419656	-0.748773	-0.293547
C	5.616306	0.335468	-1.481855
C	6.981613	0.105926	-1.304448
H	4.395945	-1.636483	0.994154
H	6.811743	-2.045069	1.318483
H	8.481670	-0.928449	-0.149339
H	5.283525	1.006812	-2.271560
H	7.701434	0.598672	-1.953195
H	2.825624	-0.962133	-1.454554
C	2.248344	1.447863	0.871188
C	1.326866	1.342800	2.095943
C	3.627477	1.934479	1.344491
C	1.653887	2.476378	-0.106673
H	1.518937	3.441608	0.395729
H	2.309326	2.649374	-0.966604
H	0.684038	2.132042	-0.477773
H	3.526854	2.901122	1.851855
H	4.075527	1.222551	2.044384
H	4.323294	2.066562	0.511753
H	1.258670	2.311524	2.601881
H	0.310169	1.049683	1.806846
H	1.700843	0.607048	2.814820
H	2.386053	-0.903366	1.116725
H	1.974562	-1.755879	1.542482
C	-4.685889	-0.465616	-0.217111
N	-3.701453	-1.265859	-0.056785
C	-4.555018	0.997595	-0.293145
C	-3.362801	1.636547	-0.669232
C	-3.293530	3.026001	-0.702836
C	-4.410176	3.795465	-0.367425
C	-5.679258	1.779289	0.013581
C	-5.605620	3.169732	-0.012943
H	-2.494468	1.056589	-0.977604
H	-2.368278	3.509093	-1.002681
H	-4.351163	4.879806	-0.395685
H	-6.612541	1.291615	0.286210
H	-6.479795	3.763454	0.238074
H	-5.713097	-0.837660	-0.292162
C	-3.903173	-2.734974	-0.015000
C	-2.938882	-3.323637	-1.057621
C	-3.496114	-3.195871	1.394220
C	-5.331771	-3.196882	-0.317123
H	-5.360348	-4.290170	-0.304324
H	-6.051384	-2.847510	0.431662
H	-5.667791	-2.871702	-1.308156
H	-3.553624	-4.286914	1.461080
H	-2.468155	-2.900786	1.631498
H	-4.162673	-2.769397	2.151324
H	-2.990758	-4.416859	-1.041395
H	-3.196705	-2.981648	-2.065065
H	-1.899301	-3.041760	-0.856354

62

A7

Al	-0.103670	-1.591640	1.557270
H	-0.432462	-3.111502	1.960298
H	1.536741	-1.424590	2.105057
H	-2.164077	-0.568602	-1.484093
H	0.416367	-1.315725	-0.026520
Li	2.136096	-1.023093	0.488336
C	-2.308786	-0.245094	-0.449367
N	-2.417835	-1.437161	0.405108
C	-3.441908	0.760917	-0.372925
C	-3.813426	1.314330	0.860255
C	-4.827833	2.267182	0.941912
C	-5.483873	2.695721	-0.213542
C	-4.099291	1.208847	-1.523711
C	-5.111550	2.166802	-1.448962
H	-3.291468	1.008632	1.765411
H	-5.101309	2.681850	1.908799
H	-6.274950	3.438054	-0.151087
H	-3.818229	0.800923	-2.492696
H	-5.611742	2.496025	-2.356191
H	-1.380973	0.268479	-0.163938
C	-3.130154	-2.626176	-0.142343
C	-3.422340	-3.544812	1.049965
C	-4.456492	-2.279549	-0.839284
C	-2.197365	-3.345960	-1.123403
H	-2.683861	-4.244491	-1.518466
H	-1.937030	-2.709871	-1.976558
H	-1.271141	-3.637871	-0.621173
H	-4.966095	-3.199884	-1.146995
H	-5.125762	-1.728085	-0.170545
H	-4.304490	-1.672980	-1.737168
H	-3.885617	-4.475743	0.707112
H	-2.500458	-3.784387	1.586306
H	-4.118927	-3.066208	1.750119
H	-2.861759	-1.170094	1.280435
H	-0.782168	-0.301535	2.267802
C	4.201747	0.856432	-0.305727
N	3.957639	-0.381694	-0.095332
C	3.260889	1.936203	0.023937
C	2.252064	1.791316	0.990450
C	1.361559	2.832145	1.234744
C	1.473402	4.032515	0.529514
C	3.383557	3.157360	-0.656866
C	2.489778	4.196687	-0.412677
H	2.177303	0.887085	1.593505
H	0.583427	2.701964	1.980512
H	0.774475	4.841611	0.721767
H	4.174955	3.284236	-1.392362
H	2.586117	5.132865	-0.955015
H	5.139502	1.182603	-0.768584
C	4.953639	-1.433091	-0.394813
C	4.249376	-2.452289	-1.304787
C	6.234157	-0.938776	-1.074057
C	5.297877	-2.087695	0.953681
H	5.958120	-2.947195	0.798466
H	5.805911	-1.375625	1.611726
H	4.395674	-2.435296	1.467232
H	6.892537	-1.792343	-1.259865
H	6.030491	-0.465318	-2.041114
H	6.788992	-0.232687	-0.446719
H	4.917238	-3.291513	-1.522708
H	3.352304	-2.865262	-0.829311
H	3.953009	-1.993018	-2.253344

29

Amine

C	-0.192853	0.635106	0.466537
N	-1.093284	-0.249422	-0.262015
C	1.255373	0.276622	0.210141
C	1.676142	-1.059555	0.218445
C	3.016557	-1.383155	0.014994
C	3.959720	-0.376161	-0.201019
C	2.206685	1.277120	-0.014096
C	3.550463	0.956544	-0.215154
H	0.936977	-1.839653	0.376850
H	3.327057	-2.424861	0.024990
H	5.004277	-0.629573	-0.360921
H	1.892893	2.318966	-0.033120
H	4.274581	1.748183	-0.389121
C	-2.545782	-0.066518	-0.081554
C	-3.218774	-1.053339	-1.042940
C	-2.929284	-0.430232	1.358656
C	-3.022766	1.362746	-0.404253
H	-4.112683	1.439044	-0.320463
H	-2.590229	2.099822	0.280534
H	-2.744346	1.644584	-1.426851
H	-4.018094	-0.418149	1.475021
H	-2.564098	-1.431454	1.608007
H	-2.518239	0.277516	2.086093
H	-4.308733	-1.005318	-0.952184
H	-2.962032	-0.824126	-2.084733
H	-2.893493	-2.076223	-0.829308
H	-0.873384	-0.195967	-1.253913
H	-0.332126	1.705681	0.235705
H	-0.398222	0.522414	1.538980

87

B1

Al	1.095285	-0.068903	-1.401157
H	1.139821	0.980204	-2.626290
H	0.627833	0.736538	-0.023767
H	3.528008	-1.959895	0.682073
H	-0.326569	-0.947473	-1.527622
Li	-1.217491	0.117041	-0.120272
C	3.162376	-0.977383	0.342325
N	2.621775	-1.042246	-0.999724
C	4.271293	0.041886	0.605302
C	4.450025	1.164378	-0.207347
C	5.430484	2.113287	0.087469
C	6.251479	1.955979	1.204612
C	5.102387	-0.106587	1.723017
C	6.083444	0.837903	2.023773
H	3.820156	1.275667	-1.086315
H	5.556632	2.976804	-0.561650
H	7.018316	2.692102	1.431629
H	4.982688	-0.979152	2.364387
H	6.722116	0.697882	2.892748
H	2.348801	-0.727092	1.040020
C	3.243982	-2.018015	-1.910315
C	2.674802	-1.793480	-3.316938
C	4.774731	-1.861904	-1.983490
C	2.909404	-3.464719	-1.484464
H	3.359753	-4.195998	-2.167125
H	3.279919	-3.687059	-0.477881
H	1.824385	-3.613477	-1.484037
H	5.200638	-2.589699	-2.684706
H	5.045741	-0.856926	-2.320560
H	5.248408	-2.025468	-1.010199

H	3.088337	-2.522058	-4.023094
H	1.582878	-1.913898	-3.327403
H	2.910801	-0.788418	-3.680507
C	-3.009482	2.501104	-1.309413
N	-2.759504	1.245537	-1.296718
C	-2.107021	3.557293	-0.827974
C	-0.711523	3.428605	-0.864606
C	0.097099	4.467487	-0.410047
C	-0.474327	5.643704	0.078597
C	-2.673403	4.756979	-0.367110
C	-1.863222	5.789426	0.096917
H	-0.257483	2.530832	-1.272867
H	1.176911	4.357371	-0.447142
H	0.161296	6.451764	0.430442
H	-3.755299	4.873506	-0.363739
H	-2.311836	6.708477	0.463603
H	-3.958570	2.879557	-1.704891
C	-3.718259	0.318571	-1.955599
C	-3.900386	-0.880345	-1.018890
C	-5.096083	0.914375	-2.274600
C	-3.039820	-0.137954	-3.258106
H	-3.647557	-0.906814	-3.747763
H	-2.924390	0.704054	-3.948376
H	-2.047949	-0.549584	-3.052016
H	-5.731020	0.126504	-2.690569
H	-5.593949	1.301143	-1.377699
H	-5.050169	1.712643	-3.022812
H	-4.582755	-1.611553	-1.463441
H	-2.951602	-1.397766	-0.846394
H	-4.313601	-0.570517	-0.053303
C	-2.009690	-1.820075	2.308037
N	-1.586905	-0.670873	1.934026
C	-2.086222	-3.029178	1.475438
C	-1.294503	-3.227706	0.335268
C	-1.409223	-4.405330	-0.398334
C	-2.306625	-5.399663	-0.003403
C	-2.966142	-4.046192	1.880401
C	-3.085895	-5.219325	1.140526
H	-0.581462	-2.469847	0.024021
H	-0.788004	-4.549065	-1.277438
H	-2.388700	-6.317945	-0.578425
H	-3.565533	-3.909242	2.778139
H	-3.777961	-5.993765	1.459016
H	-2.358928	-1.978993	3.334011
C	-1.468650	0.410117	2.950806
C	-2.034184	1.685452	2.317116
C	-2.197261	0.148443	4.275979
C	0.034926	0.587825	3.218155
H	0.198099	1.433506	3.894768
H	0.453941	-0.311065	3.682341
H	0.574600	0.779079	2.286585
H	-2.100375	1.035242	4.909142
H	-3.267623	-0.035854	4.127123
H	-1.770797	-0.689583	4.837371
H	-1.954874	2.524414	3.015354
H	-1.475237	1.967690	1.419804
H	-3.088682	1.561190	2.049616

87

B2*

Al	0.243670	-1.283948	-1.142107
H	-1.354597	-2.270848	-0.109345
H	-0.510315	-1.766811	-2.449524
H	2.743476	-1.921869	1.380029
H	0.186361	0.283373	-0.809735
Li	-1.213389	0.926138	0.438959
C	2.519370	-1.338692	0.475267
N	1.706694	-2.108603	-0.460793
C	3.828595	-0.759440	-0.050530
C	3.964993	-0.314703	-1.368452
C	5.153006	0.268345	-1.808859
C	6.230088	0.419236	-0.934229
C	4.915023	-0.601676	0.818141
C	6.106258	-0.018748	0.384775
H	3.136121	-0.445617	-2.059047
H	5.239325	0.602218	-2.839960
H	7.158900	0.865824	-1.279137
H	4.830876	-0.949984	1.846551
H	6.940791	0.082915	1.074212
H	1.918919	-0.488019	0.828213
C	2.034537	-3.550132	-0.582734
C	1.222764	-4.149367	-1.735853
C	3.526184	-3.775457	-0.883868
C	1.659612	-4.303240	0.708560
H	1.889844	-5.370574	0.613766
H	2.210242	-3.928271	1.577137
H	0.590247	-4.197741	0.916133
H	3.721171	-4.846625	-1.007639
H	3.822849	-3.261823	-1.803085
H	4.169564	-3.414843	-0.076245
H	1.455944	-5.213603	-1.844701
H	0.144174	-4.067752	-1.557031
H	1.449613	-3.650429	-2.683769
C	-2.144018	-1.853015	0.604626
N	-1.739879	-0.669380	1.294553
C	-3.414727	-1.782080	-0.240025
C	-4.517484	-1.038263	0.193747
C	-5.710224	-1.034636	-0.529194
C	-5.817979	-1.766180	-1.713532
C	-3.534011	-2.509626	-1.430611
C	-4.721409	-2.502165	-2.163714
H	-4.419962	-0.461434	1.108065
H	-6.559560	-0.460089	-0.166396
H	-6.744968	-1.759980	-2.281113
H	-2.682538	-3.081260	-1.793896
H	-4.787932	-3.068333	-3.089551
H	-2.272875	-2.724069	1.272604
C	-1.604470	-0.820236	2.746518
C	-1.100267	0.521941	3.293613
C	-2.945505	-1.138876	3.447593
C	-0.574223	-1.898666	3.156020
H	-0.436365	-1.933095	4.244551
H	-0.886696	-2.900279	2.840871
H	0.394144	-1.689313	2.689521
H	-2.817031	-1.281360	4.528535
H	-3.658101	-0.320318	3.295237
H	-3.397096	-2.054402	3.048573
H	-1.037122	0.519674	4.387716
H	-0.094577	0.734372	2.907955
H	-1.776697	1.336424	3.001710
C	-0.640587	3.763068	-0.559776
N	-1.538567	2.893210	-0.289836
C	0.745325	3.705199	-0.075873
C	1.106337	3.019742	1.092434
C	2.433310	2.988739	1.508405
C	3.417386	3.642438	0.763675
C	1.735530	4.387161	-0.798316
C	3.065752	4.345563	-0.388605
H	0.337853	2.545751	1.695424
H	2.702265	2.459229	2.417726
H	4.454209	3.606606	1.084477
H	1.460782	4.938614	-1.694823
H	3.827352	4.861140	-0.966418
H	-0.860412	4.625626	-1.197916
C	-2.921179	3.067850	-0.799484
C	-3.840973	2.919238	0.421792
C	-3.198360	4.410466	-1.485165
C	-3.173994	1.924562	-1.794409
H	-4.199438	1.975162	-2.173309
H	-2.487527	1.990685	-2.644683
H	-3.056720	0.942544	-1.325716
H	-4.255629	4.452257	-1.762787
H	-2.998689	5.260116	-0.822384
H	-2.619606	4.538964	-2.406600
H	-4.890195	2.950951	0.111177
H	-3.665433	1.964062	0.926698
H	-3.668174	3.728441	1.139346

87

B3

Al	0.821777	-1.104670	-0.507204
H	-1.340587	-2.237555	-1.637361
H	0.587019	-1.393192	-2.077786
H	3.870133	-1.082504	1.657649
H	0.800412	0.541372	-0.264837
Li	-1.038680	0.627599	-0.138363
C	3.281529	-0.651708	0.832588
N	2.452998	-1.654296	0.189031
C	4.241266	0.156800	-0.040935
C	4.018333	0.353960	-1.406412
C	4.876112	1.151953	-2.164845
C	5.975716	1.771060	-1.569254
C	5.349665	0.781271	0.545201
C	6.210229	1.581394	-0.206317
H	3.172777	-0.140994	-1.877401
H	4.687463	1.285661	-3.227572
H	6.647184	2.387679	-2.161156
H	5.544668	0.633508	1.606762
H	7.068659	2.049386	0.269740
H	2.624204	0.089267	1.313350
C	3.035405	-3.005181	0.078507
C	2.075085	-3.880301	-0.736479
C	4.398468	-3.003613	-0.641169
C	3.218075	-3.654747	1.467743
H	3.612225	-4.673679	1.373402
H	3.922125	-3.094305	2.091940
H	2.263019	-3.703969	2.000105
H	4.786870	-4.025800	-0.726708
H	4.305413	-2.583931	-1.647242
H	5.143071	-2.411172	-0.100400
H	2.448975	-4.907879	-0.801171
H	1.082609	-3.923065	-0.269703
H	1.957689	-3.493509	-1.753546
C	-1.747270	-2.354028	-0.623875
N	-0.978847	-1.438636	0.236630
C	-3.254989	-2.174179	-0.729039
C	-3.843624	-0.931588	-0.992846
C	-5.222790	-0.797662	-1.140973
C	-6.052074	-1.915966	-1.035282
C	-4.100713	-3.286054	-0.634543
C	-5.483099	-3.163833	-0.785108
H	-3.216523	-0.047547	-1.083887
H	-5.653560	0.179987	-1.343486
H	-7.127779	-1.814306	-1.149339
H	-3.667716	-4.264509	-0.435541
H	-6.115074	-4.044351	-0.702171
H	-1.564782	-3.408219	-0.364228
C	-1.164055	-1.665021	1.704347
C	-2.568399	-1.256078	2.192047
C	-0.931170	-3.131008	2.115256
C	-0.139381	-0.800305	2.453471
H	-0.266369	-0.914869	3.535478
H	0.883848	-1.095537	2.199308
H	-0.255798	0.266952	2.228347
H	-0.954986	-3.225289	3.206825
H	-1.701650	-3.799899	1.719365
H	0.044499	-3.482491	1.767899
H	-2.622372	-1.333497	3.284763
H	-2.796460	-0.219398	1.919234
H	-3.354868	-1.887493	1.773882
C	-1.074476	3.489391	-0.290957
N	-1.474195	2.515783	-1.017534
C	-1.100381	3.456828	1.179667
C	-2.069612	2.720433	1.877156
C	-2.084852	2.720453	3.269591
C	-1.132526	3.454086	3.978967
C	-0.163878	4.212273	1.900849
C	-0.172001	4.199795	3.292844
H	-2.834329	2.185105	1.319844
H	-2.844533	2.154907	3.801064
H	-1.145051	3.452888	5.065196
H	0.582199	4.795159	1.366128
H	0.567571	4.774004	3.843215
H	-0.684897	4.414167	-0.729274
C	-1.537228	2.661495	-2.493712
C	-3.029684	2.785018	-2.843625
C	-0.775783	3.869140	-3.052872
C	-0.964319	1.375708	-3.099386
H	-1.036959	1.405907	-4.191076
H	0.085578	1.237983	-2.827241
H	-1.517466	0.492818	-2.762491
H	-0.829946	3.846557	-4.145134
H	-1.203219	4.825515	-2.732231
H	0.283355	3.842706	-2.774370
H	-3.157014	2.820146	-3.930500
H	-3.594064	1.928855	-2.463819
H	-3.457777	3.697659	-2.415597

89

B4

Al	-1.406604	-1.054093	0.477940
H	0.133948	-3.112605	1.480489
H	-1.423092	-1.473430	2.035197
H	-4.104947	-0.231301	-1.988144
H	-0.977357	0.542553	0.353703
Li	0.866903	0.291025	0.176626
C	-3.514482	0.024339	-1.094662
N	-3.032403	-1.165434	-0.417352
C	-4.324755	1.040031	-0.288284
C	-4.192118	1.167631	1.097359
C	-4.895858	2.148465	1.797361
C	-5.746872	3.024495	1.122037
C	-5.183381	1.923479	-0.954553
C	-5.888689	2.907511	-0.261515
H	-3.543477	0.476763	1.629879
H	-4.782698	2.224941	2.876268
H	-6.299517	3.784521	1.668245
H	-5.306031	1.835289	-2.033424
H	-6.555458	3.576569	-0.800273
H	-2.647033	0.579646	-1.484491
C	-3.955071	-2.318014	-0.399924
C	-3.325722	-3.431361	0.447341
C	-5.323433	-1.972106	0.220284
C	-4.193665	-2.871711	-1.822006
H	-4.851194	-3.748673	-1.792349
H	-4.672287	-2.133806	-2.474400
H	-3.248978	-3.168615	-2.288021

H	-5.967414	-2.859695	0.242677
H	-5.203491	-1.606272	1.244342
H	-5.846059	-1.198916	-0.351483
H	-3.954342	-4.328315	0.432738
H	-2.339268	-3.716949	0.060568
H	-3.206317	-3.114177	1.487770
C	0.910137	-2.718640	0.808126
N	0.311020	-1.752777	-0.135447
C	2.002078	-2.148231	1.695537
C	1.735688	-1.049957	2.528751
C	2.713772	-0.539384	3.382921
C	3.982085	-1.122089	3.425947
C	3.274834	-2.727283	1.758229
C	4.256468	-2.222978	2.614306
H	0.727706	-0.635322	2.548990
H	2.479799	0.302583	4.029357
H	4.744140	-0.728825	4.093138
H	3.498165	-3.591430	1.135582
H	5.235918	-2.693916	2.650399
H	1.318264	-3.591147	0.284717
C	0.589933	-2.054793	-1.567737
C	2.099427	-2.109163	-1.859133
C	-0.048165	-3.383153	-2.016738
C	-0.015428	-0.929291	-2.418152
H	0.160522	-1.117676	-3.482801
H	-1.097020	-0.860303	-2.263784
H	0.433737	0.047183	-2.190115
H	0.163380	-3.583246	-3.073924
H	0.333336	-4.233727	-1.442033
H	-1.132531	-3.344260	-1.887231
H	2.276536	-2.296203	-2.924568
H	2.588819	-1.164949	-1.599865
H	2.600600	-2.906373	-1.301309
C	2.863203	2.600410	-0.477560
N	1.762189	2.273639	0.087273
C	3.924042	1.670601	-0.895104
C	4.210248	0.489560	-0.196606
C	5.266282	-0.323127	-0.603474
C	6.038985	0.026149	-1.711314
C	4.726691	2.030482	-1.989758
C	5.766401	1.205368	-2.407450
H	3.634478	0.224896	0.686159
H	5.484346	-1.228611	-0.046470
H	6.860436	-0.612243	-2.024585
H	4.525012	2.958714	-2.519831
H	6.370276	1.485925	-3.265673
H	3.092314	3.649932	-0.689331
C	0.835724	3.365078	0.517726
C	0.322723	2.966503	1.908532
C	1.484223	4.753401	0.610800
C	-0.324726	3.412111	-0.487320
H	-1.060621	4.158936	-0.172758
H	0.038066	3.684697	-1.484485
H	-0.834017	2.446439	-0.544323
H	0.760399	5.446538	1.049082
H	2.371040	4.748194	1.254822
H	1.760545	5.164273	-0.366253
H	-0.390723	3.712038	2.273538
H	-0.196233	2.004189	1.875415
H	1.149825	2.903468	2.623693
H	1.318724	4.178778	-5.909018
H	1.555820	3.480052	-6.004025

89

B5*-Bridging

Al	-1.515263	-1.261285	0.470298
H	0.808426	-3.360471	1.134488
H	-1.072498	-1.494851	1.986032
H	-4.251425	-0.717320	-1.883021
H	-0.965268	0.160253	-0.104061
Li	0.915431	0.072385	0.043845
C	-3.660481	-0.264605	-1.070537
N	-3.277445	-1.249832	-0.074045
C	-4.408999	0.972857	-0.579460
C	-4.332315	1.405055	0.746742
C	-4.975561	2.573619	1.158215
C	-5.705836	3.335037	0.245183
C	-5.146687	1.743657	-1.486900
C	-5.789116	2.913833	-1.083404
H	-3.775775	0.799952	1.457466
H	-4.911316	2.886990	2.197757
H	-6.211615	4.242106	0.565659
H	-5.223487	1.419274	-2.523940
H	-6.361820	3.492920	-1.803780
H	-2.750751	0.111789	-1.562040
C	-4.265253	-2.321484	0.185500
C	-3.880773	-3.044971	1.483039
C	-5.691710	-1.771663	0.369630
C	-4.274359	-3.344338	-0.970463

H	-5.010546	-4.138139	-0.795023
H	-4.528351	-2.865613	-1.922829
H	-3.288726	-3.809207	-1.077837
H	-6.380533	-2.596594	0.584947
H	-5.735937	-1.061660	1.200438
H	-6.060720	-1.262574	-0.526025
H	-4.596075	-3.846367	1.698827
H	-2.887816	-3.503857	1.416975
H	-3.876919	-2.349128	2.328354
C	1.549570	-2.816861	0.513825
N	0.843585	-1.907675	-0.399438
C	2.525626	-2.147318	1.462336
C	2.077977	-1.227371	2.423192
C	2.969356	-0.620600	3.309200
C	4.329555	-0.931247	3.262869
C	3.891892	-2.455560	1.435507
C	4.786474	-1.858087	2.325540
H	1.011282	-1.024035	2.503416
H	2.597210	0.080878	4.051620
H	5.023231	-0.466104	3.957893
H	4.258439	-3.182967	0.713941
H	5.840925	-2.121383	2.290931
H	2.096539	-3.602690	-0.023376
C	1.109172	-2.158364	-1.844854
C	2.590858	-1.947657	-2.199957
C	0.698297	-3.576377	-2.307613
C	0.276150	-1.150121	-2.648391
H	0.429422	-1.293314	-3.723741
H	-0.794486	-1.263304	-2.445819
H	0.553709	-0.113805	-2.416134
H	0.933707	-3.714879	-3.369085
H	1.228598	-4.358707	-1.754334
H	-0.375498	-3.745869	-2.182448
H	2.762445	-2.105618	-3.271955
H	2.913000	-0.931958	-1.951600
H	3.238920	-2.644771	-1.658711
C	2.510980	2.749502	-0.305702
N	1.510307	2.184961	0.258219
C	3.682200	2.061791	-0.868777
C	4.186929	0.872162	-0.326537
C	5.341816	0.300206	-0.855405
C	5.996544	0.898614	-1.932314
C	4.364507	2.674231	-1.932702
C	5.504101	2.087593	-2.474248
H	3.699923	0.414983	0.530347
H	5.729690	-0.614291	-0.418168
H	6.895923	0.446499	-2.341235
H	3.991149	3.611233	-2.340157
H	6.014564	2.561527	-3.307786
H	2.559295	3.840432	-0.394753
C	0.459092	3.065011	0.854885
C	0.966907	4.453989	1.272697
C	-0.667079	3.221229	-0.177141
C	-0.050925	2.338625	2.105847
H	-0.852271	2.915855	2.577549
H	-0.458973	1.355042	1.856208
H	0.755535	2.210428	2.835376
H	-1.490546	3.802653	0.248968
H	-0.301436	3.743402	-1.068330
H	-1.065361	2.249146	-0.477494
H	0.179234	4.959815	1.838876
H	1.850523	4.382968	1.916936
H	1.204106	5.100668	0.421600
H	-0.504593	-2.697851	-0.240085
H	-1.239519	-3.096427	-0.008222

89

B5*-Terminal

Al	-1.363101	-0.842841	-0.416612
H	-0.290409	-2.574272	1.354089
H	-1.412065	-0.699946	1.212535
H	-4.892427	-0.326082	-1.962383
H	-0.926482	0.654540	-0.936335
Li	0.915568	0.387987	-0.327843
C	-4.054274	-0.004556	-1.328604
N	-3.333861	-1.158389	-0.799599
C	-4.575153	1.050071	-0.365918
C	-3.920946	1.368419	0.829576
C	-4.403785	2.382598	1.656672
C	-5.547428	3.101167	1.304055
C	-5.725110	1.773322	-0.706680
C	-6.208555	2.791698	0.115538
H	-3.041447	0.800187	1.123668
H	-3.887027	2.606870	2.586745
H	-5.924090	3.887610	1.952800
H	-6.252141	1.534063	-1.628868
H	-7.105640	3.336110	-0.168650
H	-3.349566	0.493467	-2.017687
C	-4.104986	-2.263842	-0.181003

C	-3.153486	-3.467289	-0.101440
C	-4.587027	-1.920644	1.238273
C	-5.313498	-2.660740	-1.045463
H	-5.775232	-3.568878	-0.643001
H	-6.085642	-1.884859	-1.055237
H	-5.009486	-2.862658	-2.078861
H	-5.083262	-2.788354	1.690642
H	-3.740999	-1.635764	1.870586
H	-5.300246	-1.092082	1.229060
H	-3.654590	-4.324375	0.360263
H	-2.806849	-3.769481	-1.095286
H	-2.277481	-3.236456	0.517865
C	0.638568	-2.531821	0.772295
N	0.411458	-1.688506	-0.420164
C	1.704096	-2.046676	1.743764
C	1.543823	-0.814766	2.396384
C	2.473753	-0.368501	3.335649
C	3.588665	-1.150049	3.645628
C	2.815720	-2.827024	2.081250
C	3.752246	-2.385332	3.018455
H	0.645757	-0.231116	2.198892
H	2.320170	0.583130	3.838565
H	4.314185	-0.805690	4.377669
H	2.949431	-3.798286	1.609721
H	4.607103	-3.011056	3.263519
H	0.863298	-3.570035	0.493616
C	1.103466	-2.205821	-1.644983
C	2.566596	-2.596111	-1.368957
C	0.389652	-3.438603	-2.232919
C	1.132729	-1.085577	-2.697254
H	1.557961	-1.456074	-3.636520
H	0.130002	-0.704412	-2.907391
H	1.778121	-0.253033	-2.376977
H	0.942471	-3.837446	-3.092257
H	0.317704	-4.240427	-1.489308
H	-0.619018	-3.178431	-2.562455
H	3.069663	-2.823319	-2.315724
H	3.113649	-1.782806	-0.885118
H	2.650490	-3.485823	-0.738762
C	2.992873	2.674446	-0.575889
N	1.810697	2.343382	-0.213924
C	4.117554	1.749369	-0.778957
C	4.267236	0.573393	-0.031036
C	5.387056	-0.234496	-0.216345
C	6.359683	0.113960	-1.154260
C	5.117156	2.108937	-1.697445
C	6.222391	1.287591	-1.898140
H	3.532522	0.312983	0.726186
H	5.499310	-1.134071	0.381024
H	7.230129	-0.520610	-1.295853
H	5.018954	3.033489	-2.262123
H	6.981273	1.567200	-2.623290
H	3.248254	3.724591	-0.754784
C	0.815525	3.431353	0.030282
C	1.442713	4.785858	0.392471
C	-0.036236	3.582803	-1.239169
C	-0.051447	2.960761	1.204985
H	-0.833151	3.696901	1.416121
H	-0.550821	2.013477	0.978312
H	0.554694	2.835567	2.108672
H	-0.822350	4.326551	-1.072902
H	0.581765	3.916669	-2.080066
H	-0.515807	2.637320	-1.506704
H	0.646546	5.469403	0.701739
H	2.147729	4.693950	1.226408
H	1.955156	5.262470	-0.449766
H	-2.552128	-1.475331	-1.825415
H	-1.689872	-1.401191	-2.326567

89

B6

Al	0.963355	-1.450026	-0.825110
H	0.301977	-0.550719	0.404802
H	0.779444	-0.284201	-2.000977
H	-3.574420	0.359202	0.446484
Li	-0.515946	0.707552	-0.913010
C	-2.886591	-0.486163	0.350318
N	-2.323419	-0.480693	-1.015138
C	-3.562614	-1.749898	0.852993
C	-2.936456	-2.998086	0.742306
C	-3.541522	-4.144275	1.256721
C	-4.776997	-4.061816	1.901200
C	-4.796410	-1.677800	1.509329
C	-5.402069	-2.821954	2.030221
H	-1.966472	-3.079170	0.254792
H	-3.041510	-5.104246	1.158737
H	-5.246454	-4.956132	2.301806
H	-5.293098	-0.714459	1.611214
H	-6.363104	-2.744317	2.532098

H	-2.050454	-0.275683	1.028155
C	-3.272532	-0.336033	-2.159772
C	-2.453834	-0.591964	-3.430309
C	-4.443877	-1.331194	-2.124627
C	-3.819024	1.096322	-2.178646
H	-4.446462	1.248407	-3.063000
H	-4.437834	1.311686	-1.301340
H	-3.003542	1.826246	-2.208159
H	-5.046869	-1.223705	-3.033406
H	-4.085274	-2.364079	-2.075992
H	-5.101421	-1.165429	-1.267449
H	-3.082058	-0.469678	-4.318342
H	-1.610419	0.099169	-3.510208
H	-2.050400	-1.610300	-3.438156
H	-1.806973	-1.355718	-1.147608
H	-0.107630	-2.619580	-1.208636
C	-0.641005	3.620629	-0.052818
N	-0.143027	2.818723	-0.916713
C	-1.215954	3.204582	1.236275
C	-0.748612	2.077733	1.928101
C	-1.290274	1.742901	3.166796
C	-2.301752	2.525150	3.726878
C	-2.210928	4.003510	1.821238
C	-2.762223	3.657542	3.051922
H	0.057910	1.479377	1.511795
H	-0.913776	0.874100	3.698989
H	-2.720370	2.261071	4.694040
H	-2.557923	4.894597	1.302514
H	-3.541145	4.275203	3.489931
H	-0.653651	4.702386	-0.223941
C	0.553071	3.363254	-2.111775
C	2.026078	2.951629	-1.955939
C	-0.041731	2.672992	-3.345444
C	0.458227	4.883328	-2.285588
H	0.985890	5.165437	-3.201272
H	-0.578402	5.224785	-2.387785
H	0.929845	5.430006	-1.461719
H	0.479025	3.005330	-4.249135
H	0.079702	1.586871	-3.278523
H	-1.104561	2.910409	-3.459599
H	2.596483	3.262847	-2.837550
H	2.470010	3.420421	-1.072087
H	2.113223	1.866380	-1.854420
H	3.458201	-2.763014	1.449057
C	3.070875	-1.815214	1.039723
N	2.682146	-1.947826	-0.351009
C	4.076641	-0.718080	1.383642
C	4.289273	0.375308	0.541328
C	5.170451	1.396515	0.900752
C	5.854374	1.342154	2.115663
C	4.769960	-0.763801	2.599882
C	5.650067	0.253949	2.966404
H	3.762618	0.398422	-0.408718
H	5.328649	2.235611	0.226936
H	6.544192	2.134433	2.394690
H	4.621913	-1.612146	3.267052
H	6.182624	0.194750	3.912625
H	2.173188	-1.600935	1.640371
C	3.463767	-2.899788	-1.163940
C	3.063545	-2.701488	-2.632203
C	4.979635	-2.661892	-1.046464
C	3.151360	-4.359201	-0.766507
H	3.720629	-5.071174	-1.376895
H	3.406657	-4.550816	0.281694
H	2.083888	-4.566082	-0.897648
H	5.523735	-3.370535	-1.682027
H	5.241256	-1.646297	-1.358352
H	5.337524	-2.798220	-0.020991
H	3.609161	-3.394821	-3.281935
H	1.992857	-2.893921	-2.784462
H	3.280424	-1.678638	-2.957917
	87		

C1*

Al	-0.119389	-1.013292	-0.647857
H	-1.192982	-1.278916	-1.778741
H	0.147812	0.965940	-0.481707
N	1.668760	-1.276185	-1.059098
H	-1.878179	-3.043560	1.830889
C	-1.912390	-2.457804	0.906722
N	-0.969797	-1.306723	1.019941
C	-3.363750	-2.080756	0.647439
C	-4.154096	-1.488156	1.646488
C	-5.499337	-1.191333	1.418455
C	-6.081025	-1.484773	0.185674
C	-3.962480	-2.357837	-0.588892
C	-5.307308	-2.067946	-0.817794
H	-3.726113	-1.287778	2.625651
H	-6.093432	-0.741548	2.209308
H	-7.127821	-1.256471	0.006955
H	-3.364375	-2.804450	-1.378614
H	-5.749312	-2.293102	-1.784427
H	-1.611392	-3.157548	0.113660
C	-0.203064	-1.258572	2.302670
C	0.735111	-0.051392	2.232484
C	-1.132183	-1.059795	3.512373
C	0.630507	-2.534651	2.518904
H	1.266124	-2.437458	3.405928
H	0.001625	-3.419238	2.666779
H	1.273307	-2.716639	1.652914
H	-0.541865	-0.990065	4.432249
H	-1.707933	-0.130545	3.421330
H	-1.830639	-1.892843	3.644627
H	1.288577	0.062723	3.169840
H	1.483182	-0.171651	1.442453
H	0.180955	0.876334	2.056370
Li	-2.349287	0.229950	0.707601
C	-0.518579	1.847932	-0.791157
N	-1.863519	1.582638	-0.435735
C	0.221369	3.016951	-0.125149
C	-0.220796	3.543735	1.090115
C	0.469851	4.585191	1.709871
C	1.616735	5.117372	1.118534
C	1.368495	3.561218	-0.714039
C	2.063207	4.603410	-0.100120
H	-1.133588	3.135248	1.517522
H	0.109699	4.987743	2.653751
H	2.153710	5.931454	1.598166
H	1.713731	3.173712	-1.671866
H	2.947977	5.019785	-0.575355
H	-0.323684	1.917049	-1.879778
C	-2.863475	2.236286	-1.290124
C	-4.230470	1.956723	-0.652098
C	-2.689570	3.767065	-1.387578
C	-2.878001	1.653033	-2.721851
H	-3.659466	2.116635	-3.337281
H	-1.924477	1.819826	-3.236243
H	-3.053272	0.572866	-2.686652
H	-3.486756	4.215411	-1.994154
H	-2.715086	4.223534	-0.392710
H	-1.734255	4.036736	-1.851169
H	-5.046966	2.409498	-1.226430
H	-4.427587	0.877507	-0.607571
H	-4.265046	2.364524	0.366052
C	2.638237	-0.188997	-1.001205
C	1.925884	-2.305255	-2.112212
C	3.920642	-0.396609	-0.200089
H	2.930259	0.139290	-2.012727
H	2.155012	0.696418	-0.564039
C	1.556381	-1.775953	-3.513697
C	3.391102	-2.769778	-2.137347
C	1.061933	-3.535221	-1.792350
H	1.222999	-4.324540	-2.534683
H	1.314180	-3.936600	-0.804994
H	-0.010502	-3.300520	-1.818701
H	1.761104	-2.526488	-4.286337
H	0.495312	-1.512487	-3.564984
H	2.140584	-0.883491	-3.763401
H	3.510192	-3.543112	-2.904625
H	4.081073	-1.957163	-2.381734
H	3.695871	-3.190770	-1.175876
C	5.020122	0.432273	-0.457282
C	6.191563	0.328174	0.291119
C	6.285931	-0.614888	1.315943
C	4.028199	-1.335973	0.827008
C	5.198673	-1.447646	1.578992
H	4.958273	1.168789	-1.256902
H	7.032893	0.980422	0.071193
H	7.198751	-0.702067	1.898948
H	3.187171	-1.993510	1.021109
H	5.261664	-2.190219	2.370754
	87		

C2

Al	0.574730	-0.370735	0.383818
H	0.946274	2.266656	0.498097
H	0.934223	0.549077	1.645437
H	3.754074	-1.790161	1.398677
C	3.212329	-0.908348	1.026038
N	2.182201	-1.294659	0.059451
C	4.252731	0.122372	0.600598
C	3.978617	1.104500	-0.353980
C	4.923671	2.080932	-0.672276
C	6.165450	2.093502	-0.036705
C	5.502943	0.145321	1.232172
C	6.451835	1.118536	0.920393
H	3.017724	1.085179	-0.858871
H	4.690281	2.833908	-1.421507
H	6.903320	2.851451	-0.286050
H	5.737674	-0.613474	1.977549
H	7.417473	1.112367	1.420162
H	2.722104	-0.482635	1.912333
C	2.575075	-2.462614	-0.758852
C	1.555243	-2.690981	-1.872574
C	3.945984	-2.262216	-1.436987
C	2.628352	-3.758582	0.082281
H	2.954453	-4.607411	-0.530753
H	3.327360	-3.678793	0.920604
H	1.640398	-3.990357	0.490603
H	4.175939	-3.118520	-2.082099
H	3.947870	-1.355986	-2.050217
H	4.758061	-2.171385	-0.710288
H	1.813545	-3.583641	-2.452462
H	0.553641	-2.855845	-1.461207
H	1.529763	-1.843294	-2.562310
H	-1.702830	-3.191487	0.317872
C	-1.762506	-2.140497	-0.001826
N	-1.134130	-1.229578	0.956869
C	-3.212226	-1.843952	-0.395304
C	-3.985036	-0.838475	0.194886
C	-5.280543	-0.555541	-0.252121
C	-5.831767	-1.286625	-1.299692
C	-3.787852	-2.573473	-1.447082
C	-5.079053	-2.305557	-1.891258
H	-3.602875	-0.307506	1.067511
H	-5.857217	0.228822	0.231026
H	-6.837140	-1.072274	-1.650433
H	-3.207214	-3.359492	-1.926726
H	-5.499781	-2.887371	-2.707205
H	-1.207719	-2.127807	-0.945844
C	-1.202493	-1.747931	2.376862
C	-1.119117	-0.580349	3.369769
C	-2.515272	-2.490538	2.705408
C	-0.041764	-2.720853	2.647253
H	-0.076086	-3.102102	3.674778
H	-0.090430	-3.583455	1.973512
H	0.925951	-2.233741	2.494064
H	-2.472413	-2.822808	3.747992
H	-3.393471	-1.847738	2.605911
H	-2.677931	-3.384315	2.096560
H	-1.118615	-0.961137	4.397219
H	-0.213651	0.011231	3.223606
H	-1.992524	0.075552	3.267046
Li	-1.877432	0.521247	0.193521
C	0.176082	2.383141	-0.272343
N	-0.204285	1.041235	-0.797506
C	-0.976527	3.149559	0.358189
C	-1.608111	2.632843	1.501367
C	-2.681102	3.294187	2.102666
C	-3.138055	4.502712	1.576868
C	-1.430579	4.377812	-0.136519
C	-2.498684	5.046992	0.462414
H	-1.198161	1.737794	1.969209
H	-3.140843	2.878331	2.995906
H	-3.970656	5.024009	2.040786
H	-0.940971	4.821398	-1.000100
H	-2.832756	5.998325	0.056334
H	0.626709	3.003957	-1.054555
C	-0.343523	1.059763	-2.287974
C	-1.001218	-0.236732	-2.764025
C	-1.260921	2.201114	-2.772934
C	1.022629	1.199442	-2.986123
H	0.909530	1.196521	-4.076724
H	1.521938	2.135740	-2.715454
H	1.685215	0.376051	-2.705259
H	-1.435363	2.102054	-3.850034
H	-2.235080	2.171952	-2.271270
H	-0.828018	3.190481	-2.609347
H	-1.079802	-0.240928	-3.856370
H	-0.418098	-1.114243	-2.483831
H	-2.017135	-0.342918	-2.367310
	89		

C3*-Terminal

Al	0.142018	-0.215064	0.825939
H	0.585510	2.321696	1.251040
H	0.166742	0.591894	2.220985
H	3.414856	0.398006	1.632105
C	3.006986	0.048456	0.672157
N	2.024142	-1.018054	0.874182
C	4.168258	-0.218845	-0.276062
C	4.067802	-1.096929	-1.359434
C	5.130732	-1.263275	-2.247469
C	6.316213	-0.549386	-2.069856
C	5.363520	0.491266	-0.107555
C	6.428712	0.331332	-0.993676
H	3.152795	-1.666402	-1.499776
H	5.032112	-1.954595	-3.080688

H	7.144981	-0.681049	-2.760161
H	5.463455	1.175729	0.733267
H	7.348462	0.889753	-0.839166
H	2.473208	0.919104	0.264358
C	2.404147	-2.071728	1.855104
C	1.363830	-3.192553	1.741040
C	3.785024	-2.685084	1.569484
C	2.408961	-1.519625	3.292692
H	2.616580	-2.322445	4.009462
H	3.183003	-0.756609	3.427137
H	1.448605	-1.064637	3.546045
H	3.983018	-3.482452	2.295196
H	3.832703	-3.117006	0.566008
H	4.591436	-1.951828	1.658461
H	1.567958	-3.979880	2.474023
H	0.352682	-2.820800	1.930329
H	1.383220	-3.642914	0.743045
H	-1.895880	-3.207621	0.934809
C	-1.802884	-2.384762	0.208726
N	-1.670497	-1.088621	0.879110
C	-2.903745	-2.565647	-0.835141
C	-3.855079	-1.599955	-1.169355
C	-4.802885	-1.818583	-2.172373
C	-4.817682	-3.024642	-2.867995
C	-2.936672	-3.775164	-1.546488
C	-3.877659	-4.006635	-2.545973
H	-3.897988	-0.666501	-0.610070
H	-5.533431	-1.046993	-2.402010
H	-5.552618	-3.200511	-3.648342
H	-2.203817	-4.545457	-1.312046
H	-3.876160	-4.953977	-3.078850
H	-0.866217	-2.589931	-0.324517
C	-2.459288	-1.003532	2.169315
C	-2.763051	0.472308	2.462311
C	-3.820151	-1.727204	2.113767
C	-1.679740	-1.601393	3.353306
H	-2.261924	-1.531667	4.279720
H	-1.464143	-2.662219	3.184622
H	-0.737926	-1.069263	3.504645
H	-4.310770	-1.617741	3.086936
H	-4.490672	-1.305140	1.361839
H	-3.728804	-2.799271	1.919697
H	-3.288222	0.579302	3.417806
H	-1.848287	1.067958	2.512257
H	-3.435684	0.890239	1.696139
Li	-2.033736	0.518321	-0.257615
C	0.304098	2.564067	0.217704
N	-0.222979	1.329423	-0.390572
C	-0.598123	3.788384	0.307079
C	-1.949059	3.705304	0.656495
C	-2.736372	4.848359	0.795763
C	-2.179940	6.111551	0.594332
C	-0.052920	5.064738	0.118632
C	-0.829684	6.214487	0.259094
H	-2.393471	2.732729	0.846592
H	-3.783830	4.752475	1.070840
H	-2.790157	7.004137	0.700490
H	0.997866	5.158675	-0.150240
H	-0.380686	7.191592	0.100203
H	1.237020	2.881607	-0.274693
C	-0.125627	1.348057	-1.894798
C	-1.067923	0.277766	-2.470736
C	-0.578287	2.690234	-2.500349
C	1.302219	1.064655	-2.387429
H	1.361352	1.151022	-3.478819
H	2.017475	1.779867	-1.968228
H	1.620290	0.058578	-2.111091
H	-0.607230	2.597999	-3.591979
H	-1.577441	2.978563	-2.157501
H	0.103171	3.510142	-2.261307
H	-0.921098	0.178511	-3.550556
H	-0.894168	-0.709064	-2.031761
H	-2.129718	0.554499	-2.355967
H	0.531511	-1.422390	-0.795256
H	1.318262	-1.410203	-0.242298

89

C3*-Bridging

Al	0.786044	-0.877379	-0.979209
H	-1.401702	-2.331180	1.605262
H	0.000590	-2.133696	-0.458478
H	3.549378	-2.805244	-0.287600
C	3.057037	-1.911260	0.119093
N	2.594046	-1.025506	-0.952676
C	3.966628	-1.314111	1.183695
C	3.678542	-0.090229	1.798325
C	4.489965	0.410508	2.813924
C	5.611154	-0.307466	3.236054
C	5.091841	-2.023559	1.615695
C	5.909849	-1.528098	2.633152
H	2.815258	0.480454	1.467150
H	4.247089	1.362428	3.278992
H	6.245157	0.082978	4.027321
H	5.331344	-2.978535	1.151343
H	6.780545	-2.095573	2.951135
H	2.162121	-2.294667	0.635015
C	3.586123	-0.599093	-1.968080
C	2.822194	-0.233210	-3.245879
C	4.402247	0.625528	-1.512506
C	4.558949	-1.738446	-2.318813
H	5.190260	-1.432602	-3.159641
H	5.226066	-1.983272	-1.487109
H	4.017949	-2.644655	-2.613460
H	5.095845	0.940081	-2.301371
H	3.744021	1.469911	-1.284335
H	4.987072	0.403092	-0.617467
H	3.506371	0.115977	-4.026287
H	2.280980	-1.102962	-3.639501
H	2.107598	0.574291	-3.059680
H	1.008148	2.213569	-2.219571
C	0.596526	1.910047	-1.247912
N	-0.194761	0.663067	-1.364112
C	-0.085169	3.117676	-0.631608
C	-0.409584	3.134497	0.730425
C	-1.011915	4.249864	1.312328
C	-1.286752	5.378316	0.539834
C	-0.348013	4.265076	-1.389241
C	-0.945968	5.385702	-0.812584
H	-0.156473	2.279928	1.358097
H	-1.257731	4.239153	2.369966
H	-1.753464	6.248789	0.991911
H	-0.075036	4.286888	-2.441775
H	-1.142814	6.265143	-1.419796
H	1.468163	1.672380	-0.623497
C	-1.249981	0.630547	-2.440146
C	-1.537840	-0.836333	-2.782721
C	-2.561555	1.279674	-1.954904
C	-0.802297	1.336680	-3.729879
H	-1.552887	1.169062	-4.508941
H	-0.705805	2.418509	-3.606651
H	0.149589	0.940149	-4.097163
H	-3.288480	1.325556	-2.772969
H	-3.046978	0.685738	-1.167838
H	-2.402447	2.301433	-1.599060
H	-2.333997	-0.903359	-3.530481
H	-0.652154	-1.324199	-3.218534
H	-1.866044	-1.413786	-1.913235
Li	-1.627337	0.567281	0.404491
C	-2.413785	-1.875896	1.633891
N	-2.367444	-0.436850	1.781399
C	-3.153825	-2.405012	0.408323
C	-4.269415	-1.742300	-0.116944
C	-4.976389	-2.265445	-1.201002
C	-4.573016	-3.463999	-1.792386
C	-2.759288	-3.606367	-0.197450
C	-3.457544	-4.133189	-1.284510
H	-4.571217	-0.806548	0.345606
H	-5.848087	-1.738240	-1.582809
H	-5.120196	-3.872034	-2.638462
H	-1.887741	-4.131644	0.189704
H	-3.128791	-5.065255	-1.738501
H	-2.896165	-2.368412	2.496292
C	-2.924164	0.052854	3.044636
C	-2.744091	1.574503	3.073488
C	-4.436913	-0.238158	3.187331
C	-2.196686	-0.518416	4.286952
H	-2.592552	-0.092333	5.217996
H	-2.305433	-1.606175	4.357102
H	-1.125544	-0.293526	4.234563
H	-4.825241	0.097839	4.157808
H	-4.997640	0.278053	2.399604
H	-4.650326	-1.309493	3.102575
H	-3.204988	2.020018	3.962986
H	-1.678098	1.830705	3.088082
H	-3.205820	2.034548	2.190220
H	0.602255	-0.444364	1.842566
H	-0.162586	-0.391109	1.913407

89

C4-bridging

Al	-0.878580	-1.109610	-0.406459
H	2.711613	-2.170571	-0.514290
H	0.139673	-2.330493	-0.712924
H	-3.802634	-0.563020	-2.361003
C	-3.379039	-0.282474	-1.381605
N	-2.685349	-1.395161	-0.755540
C	-4.485433	0.405216	-0.583260
C	-4.645873	0.209163	0.790135
C	-5.633610	0.892725	1.502384
C	-6.479023	1.789665	0.849846
C	-5.342430	1.306735	-1.229010
C	-6.329350	1.993951	-0.523717
H	-3.989719	-0.500233	1.286958
H	-5.744884	0.720561	2.570428
H	-7.250103	2.320109	1.402271
H	-5.237860	1.467561	-2.301458
H	-6.986535	2.684769	-1.046423
H	-2.641275	0.499067	-1.624848
C	-3.247901	-2.736952	-1.040027
C	-2.667130	-3.741064	-0.035889
C	-4.780520	-2.777222	-0.898213
C	-2.870059	-3.194014	-2.466223
H	-3.296992	-4.176873	-2.701834
H	-3.237938	-2.486817	-3.218326
H	-1.781120	-3.258939	-2.565507
H	-5.142938	-3.792008	-1.098622
H	-5.092135	-2.496362	0.111828
H	-5.283132	-2.106778	-1.602171
H	-3.043794	-4.750064	-0.238622
H	-1.574264	-3.779138	-0.098182
H	-2.952601	-3.472161	0.986674
H	-1.207069	1.267478	2.424456
C	-0.996167	1.061460	1.367874
N	-0.274912	-0.227033	1.225711
C	-0.258590	2.275503	0.820294
C	-0.905308	3.172527	-0.041758
C	-0.256448	4.312346	-0.513650
C	1.062303	4.579003	-0.140074
C	1.066792	2.557120	1.188148
C	1.723034	3.696604	0.713303
H	-1.930418	2.970073	-0.342073
H	-0.779980	4.991701	-1.181129
H	1.568523	5.466337	-0.509780
H	1.577763	1.895690	1.884839
H	2.744818	3.899076	1.023944
H	-1.986159	1.035476	0.891254
C	-0.107677	-0.955227	2.516468
C	0.642656	-2.266170	2.256121
C	0.726014	-0.145681	3.528469
C	-1.466400	-1.299547	3.159583
H	-1.328839	-1.887109	4.074802
H	-2.032632	-0.402085	3.430611
H	-2.077671	-1.884189	2.465524
H	0.806890	-0.689676	4.476142
H	1.743446	0.013970	3.154320
H	0.285863	0.831087	3.755303
H	0.831553	-2.784173	3.202636
H	0.074883	-2.940792	1.610090
H	1.608318	-2.078766	1.775176
Li	1.150304	0.445085	-0.161950
C	3.423132	-1.488772	-0.997022
N	2.607092	-0.347343	-1.466908
C	4.527218	-1.158724	-0.017454
C	4.283334	-0.417609	1.144444
C	5.301264	-0.161499	2.060328
C	6.588859	-0.651172	1.833064
C	5.820187	-1.649473	-0.229181
C	6.844366	-1.400558	0.685907
H	3.283385	-0.039612	1.339417
H	5.088828	0.414555	2.957086
H	7.383363	-0.453234	2.547063
H	6.029451	-2.233527	-1.123012
H	7.841311	-1.790604	0.499450
H	3.856479	-2.049225	-1.836273
C	3.205350	0.567683	-2.493409
C	2.059940	1.458877	-2.989982
C	4.287078	1.450215	-1.863020
C	3.790593	-0.204029	-3.687106
H	4.106206	0.496838	-4.466733
H	4.669984	-0.792783	-3.408700
H	3.046807	-0.879054	-4.126728
H	4.631581	2.185895	-2.597758
H	3.892013	1.992946	-0.998759
H	5.154095	0.872601	-1.534649
H	2.426636	2.148989	-3.756381
H	1.247510	0.867735	-3.426160
H	1.637531	2.062347	-2.178875
H	-0.349667	0.149373	-1.347843
H	1.797669	-0.781131	-1.918848

89

C4-terminal

Al	0.257644	-0.783182	-0.210643
H	1.554726	-2.659042	-1.767855
H	-0.022302	-2.363833	-0.110899
H	-5.438661	0.927436	1.859882
C	-4.616551	0.426141	1.337186

N -3.995632 1.376409 0.424850
C -5.121539 -0.905919 0.796436
C -4.254002 -1.786245 0.136043
C -4.707583 -3.016166 -0.336220
C -6.040190 -3.393051 -0.152118
C -6.450827 -1.299265 0.982727
C -6.911370 -2.531090 0.512356
H -3.213129 -1.506067 -0.013494
H -4.017680 -3.684944 -0.844482
H -6.393761 -4.352279 -0.520786
H -7.137119 -0.632168 1.501593
H -7.949711 -2.814995 0.664582
H -3.863619 0.212112 2.107321
C -4.778389 2.041722 -0.630621
C -3.861728 3.135288 -1.198618
C -5.210289 1.110259 -1.781506
C -6.016224 2.702555 -0.013082
H -6.513966 3.333103 -0.757195
H -6.748878 1.963214 0.326963
H -5.733474 3.330631 0.838591
H -5.721559 1.679462 -2.567640
H -4.338050 0.623007 -2.231629
H -5.889496 0.326911 -1.435716
H -4.361075 3.686117 -2.003316
H -3.575085 3.839715 -0.411453
H -2.946199 2.696434 -1.612694
H 0.538553 1.594415 2.465185
C 0.944972 1.372155 1.468675
N 1.247320 -0.062407 1.321548
C 2.107560 2.330585 1.220211
C 3.449459 1.964069 1.383071
C 4.486783 2.871083 1.138940
C 4.197478 4.171042 0.734325
C 1.836280 3.645881 0.811584
C 2.862967 4.556217 0.577787
H 3.690911 0.970002 1.757342
H 5.518404 2.559700 1.281705
H 4.999045 4.879224 0.544764
H 0.801995 3.952922 0.670518
H 2.623576 5.568009 0.261224
H 0.138367 1.668228 0.781822
C 1.220242 -0.827749 2.609042
C 1.823274 -2.215698 2.363606
C 2.060357 -0.164039 3.716494
C -0.226645 -0.991277 3.114351
H -0.258725 -1.550715 4.056548
H -0.703293 -0.020652 3.291133
H -0.830159 -1.536354 2.380165
H 1.956894 -0.735462 4.645206
H 3.124415 -0.155071 3.458773
H 1.754075 0.863324 3.936110
H 1.819151 -2.800549 3.290216
H 1.257609 -2.769834 1.610343
H 2.861979 -2.133207 2.023792
Li 2.740531 0.059569 -0.028917
C 2.238842 -1.819719 -1.956010
N 1.620758 -0.521467 -1.603696
C 3.534490 -2.109812 -1.211892
C 3.739419 -3.347851 -0.589559
C 4.934037 -3.633679 0.070739
C 5.950736 -2.679677 0.133333
C 4.570773 -1.163519 -1.150095
C 5.765827 -1.441449 -0.481361
H 2.946050 -4.090853 -0.618253
H 5.066771 -4.600670 0.548638
H 6.878752 -2.900327 0.653149
H 4.456605 -0.212868 -1.670189
H 6.556839 -0.696241 -0.457345
H 2.466173 -1.859716 -3.028069
C 1.313156 0.332959 -2.791550
C 0.785879 1.686469 -2.301274
C 2.570874 0.628375 -3.632116
C 0.243478 -0.321589 -3.686698
H 0.006191 0.311605 -4.549591
H 0.576625 -1.291269 -4.072694
H -0.679868 -0.482203 -3.119785
H 2.307031 1.254622 -4.491319
H 3.311920 1.178901 -3.040722
H 3.049938 -0.271962 -4.029824
H 0.571764 2.340434 -3.153545
H -0.136983 1.570942 -1.726987
H 1.528219 2.192163 -1.673541
H -1.079423 0.100037 -0.422244
H -3.137729 0.991488 0.036794

87

D1*

Al 1.943059 -0.228470 -0.427582
H 0.829521 -2.356273 -1.440652
H 1.625876 -0.006023 -1.980705
H 5.308732 -0.317100 1.210104
H 0.475536 1.526132 -0.001759
Li -0.905513 0.448633 -0.234249
C 4.233112 -0.110532 1.172179
N 3.563762 -0.934016 0.173047
C 4.048585 1.365545 0.889893
C 2.859472 1.801540 0.280688
C 2.746826 3.144630 -0.094262
C 3.779120 4.055567 0.128318
C 5.070134 2.286417 1.143279
C 4.941740 3.623813 0.766339
H 1.572531 1.494503 0.229109
H 1.814295 3.478015 -0.549885
H 3.676149 5.091630 -0.184642
H 5.991217 1.946437 1.614413
H 5.754398 4.320874 0.955296
H 3.867409 -0.332166 2.194265
C 4.435437 -1.874334 -0.553442
C 3.576529 -2.706929 -1.511098
C 5.500973 -1.132085 -1.389148
C 5.134121 -2.855471 0.410851
H 5.742559 -3.577415 -0.146730
H 5.801721 -2.343765 1.111831
H 4.391952 -3.409952 0.994753
H 6.154613 -1.837030 -1.917838
H 5.016945 -0.485496 -2.128481
H 6.139233 -0.500655 -0.762054
H 4.202203 -3.397447 -2.086932
H 2.843372 -3.301734 -0.956616
H 3.049614 -2.066960 -2.228893
C 0.081747 -2.312579 -0.636925
N 0.371141 -1.159638 0.249705
C -1.266311 -2.298654 -1.338399
C -1.534850 -1.331189 -2.317831
C -2.739931 -1.332250 -3.021433
C -3.700457 -2.311623 -2.764927
C -2.234785 -3.282243 -1.102422
C -3.440288 -3.291414 -1.805805
H -0.765741 -0.598978 -2.562528
H -2.921781 -0.579167 -3.783671
H -4.636774 -2.319006 -3.316068
H -2.036327 -4.061914 -0.370142
H -4.173639 -4.070172 -1.611376
H 0.183205 -3.264292 -0.101890
C 0.291323 -1.487944 1.708486
C -1.118037 -1.954350 2.101167
C 1.305696 -2.567311 2.137635
C 0.599355 -0.209228 2.498707
H 0.532478 -0.404614 3.574208
H 1.612800 0.159157 2.303404
H -0.098015 0.599999 2.257590
H 1.278194 -2.703291 3.225199
H 1.085492 -3.542949 1.690184
H 2.315443 -2.275114 1.834581
H -1.167317 -2.154330 3.177789
H -1.871583 -1.195198 1.868600
H -1.401471 -2.876326 1.585293
C -3.523491 2.055340 0.249149
N -2.552492 1.889495 -0.567352
C -4.132858 0.996453 1.065918
C -4.148059 -0.342835 0.650324
C -4.795413 -1.305143 1.420915
C -5.424104 -0.946490 2.614436
C -4.791216 1.353442 2.253399
C -5.419010 0.385744 3.032365
H -3.682307 -0.619410 -0.291429
H -4.810517 -2.337129 1.083807
H -5.926936 -1.701472 3.212517
H -4.801996 2.394650 2.568236
H -5.912743 0.670204 3.957173
H -3.992711 3.040108 0.375649
C -2.167738 3.067434 -1.410257
C -3.396973 3.674317 -2.106613
C -1.473977 4.114614 -0.527804
C -1.199421 2.561777 -2.483158
H -0.880767 3.387924 -3.127126
H -0.302147 2.126739 -2.029933
H -1.682427 1.809460 -3.115153
H -1.112817 4.945497 -1.143108
H -2.161754 4.524872 0.220001
H -0.621541 3.664655 -0.010679
H -3.074136 4.455412 -2.802205
H -3.935817 2.911501 -2.678630
H -4.097840 4.136775 -1.404523

85

D2

Al -1.761906 -0.111780 0.148584
H -1.101949 -2.731612 -0.336770
H -0.826745 -0.783678 1.347517
H -4.962782 1.146082 1.035131
Li 0.826227 -0.388566 0.409661
C -4.257958 1.037571 0.192637
N -3.608180 -0.265829 0.204023
C -3.258833 2.174377 0.285348
C -1.884484 1.864420 0.296259
C -0.987118 2.939990 0.411126
C -1.420723 4.262844 0.498338
C -3.705761 3.500357 0.360743
C -2.789964 4.544098 0.466950
H 0.086919 2.753648 0.443184
H -0.700275 5.073025 0.590421
H -4.773615 3.717022 0.343197
H -3.139188 5.571964 0.531487
H -4.867649 1.188786 -0.718271
C -4.498821 -1.398250 0.469609
C -3.710329 -2.695708 0.273039
C -5.018736 -1.381944 1.925069
C -5.702835 -1.418270 -0.493926
H -6.312356 -2.315558 -0.335125
H -6.354550 -0.550751 -0.347892
H -5.360371 -1.414347 -1.534722
H -5.666645 -2.244745 2.122831
H -4.176593 -1.411665 2.624592
H -5.603050 -0.481192 2.138964
H -4.333760 -3.570828 0.486908
H -3.350565 -2.778430 -0.758039
H -2.851880 -2.727216 0.955661
C -0.354454 -2.301331 -1.012994
N -0.570989 -0.852641 -1.210542
C 1.002291 -2.741725 -0.469069
C 1.068005 -3.857149 0.381767
C 2.288342 -4.345850 0.840072
C 3.480699 -3.718541 0.468980
C 2.205614 -2.118318 -0.823656
C 3.433577 -2.599887 -0.358998
H 0.144503 -4.347704 0.682218
H 2.310562 -5.213610 1.494264
H 4.434135 -4.098763 0.825533
H 2.186930 -1.260535 -1.490803
H 4.351470 -2.100774 -0.658086
H -0.498674 -2.842110 -1.957780
C -0.756792 -0.486507 -2.649825
C 0.452675 -0.885740 -3.515122
C -2.027145 -1.139106 -3.235300
C -0.923777 1.031706 -2.768963
H -0.969363 1.319637 -3.825233
H -1.838208 1.385454 -2.286298
H -0.086335 1.562083 -2.305822
H -2.194705 -0.806544 -4.266517
H -1.961859 -2.232754 -3.256814
H -2.900065 -0.862520 -2.635221
H 0.250630 -0.661690 -4.568753
H 1.345944 -0.323405 -3.223354
H 0.685890 -1.953779 -3.451562
C 3.378871 1.068029 1.265938
N 2.268984 0.560056 1.648536
C 3.721582 1.412125 -0.122202
C 2.756020 1.688974 -1.100172
C 3.141324 2.034996 -2.392129
C 4.495348 2.104809 -2.726983
C 5.079767 1.512590 -0.462233
C 5.465562 1.842908 -1.758847
H 1.701607 1.674465 -0.840322
H 2.383475 2.263338 -3.135608
H 4.792088 2.376861 -3.735951
H 5.836156 1.322968 0.296291
H 6.520216 1.906360 -2.011052
H 4.176877 1.282685 1.983754
C 2.031802 0.328025 3.100083
C 1.704760 -1.163492 3.264928
C 3.207186 0.689153 4.015802
C 0.811237 1.181671 3.477768
H 0.534620 0.991761 4.520217
H 1.032179 2.248288 3.369120
H -0.049599 0.943411 2.847774
H 2.917448 0.482930 5.050118
H 4.101351 0.091493 3.804007
H 3.469702 1.751575 3.960768
H 1.499455 -1.384808 4.317373
H 0.812976 -1.439626 2.693100
H 2.541438 -1.788409 2.936648

87

D1* (Amide)

Al -1.490911 1.334004 -1.080115
H 0.109674 1.109836 -0.764205
H -1.864685 1.683505 2.632035

H -0.429083 0.217607 -3.131379
Li 0.778803 -0.254555 0.306244
C -1.527784 1.206444 1.704655
N -2.227841 1.777705 0.553002
C -1.645208 -0.305810 1.891352
C -1.155886 -1.203072 0.903110
C -1.252409 -2.574014 1.210987
C -1.792432 -3.060557 2.405271
C -2.193172 -0.786118 3.089333
C -2.273121 -2.155233 3.350010
H -1.372379 -0.936596 -0.551094
H -0.897317 -3.303918 0.480190
H -1.844366 -4.131080 2.594917
H -2.564083 -0.082212 3.833931
H -2.707128 -2.508514 4.282606
H -0.446758 1.451732 1.653027
C -3.226992 2.814473 0.866128
C -3.899441 3.276156 -0.432598
C -4.331881 2.282935 1.803674
C -2.566212 4.051088 1.514726
H -3.308573 4.827250 1.737890
H -2.065238 3.798492 2.455487
H -1.816824 4.474566 0.837098
H -5.079898 3.060591 1.998958
H -4.838149 1.423085 1.354151
H -3.933574 1.965184 2.772346
H -4.658899 4.035380 -0.216171
H -3.173438 3.709207 -1.127013
H -4.398461 2.442838 -0.940401
H -1.697631 2.264016 -2.368237
C -0.757448 -0.756760 -2.738288
N -1.776044 -0.548522 -1.677765
C 0.487790 -1.540041 -2.356327
C 0.444337 -2.917904 -2.093203
C 1.612625 -3.646427 -1.880894
C 2.856517 -3.013084 -1.926855
C 1.743141 -0.916252 -2.387856
C 2.917662 -1.643682 -2.176120
H -0.513334 -3.428253 -2.072684
H 1.552599 -4.714671 -1.689076
H 3.767809 -3.585057 -1.773849
H 1.800839 0.148508 -2.600739
H 3.877320 -1.135686 -2.217770
H -1.215237 -1.269565 -3.590156
C -3.144391 -1.065208 -2.021559
C -4.065522 -0.853369 -0.814399
C -3.718092 -0.303218 -3.227203
C -3.122562 -2.571784 -2.328071
H -4.142283 -2.921611 -2.521822
H -2.527314 -2.819122 -3.213549
H -2.734892 -3.133071 -1.471892
H -4.725890 -0.666744 -3.457465
H -3.778440 0.769645 -3.020328
H -3.108915 -0.432732 -4.128172
H -5.085743 -1.152683 -1.077586
H -3.741640 -1.446746 0.044250
H -4.078184 0.192388 -0.500039
C 3.726067 0.597269 1.052514
N 2.682876 -0.068040 1.383397
C 3.808893 1.641383 0.020585
C 2.707143 2.394968 -0.405633
C 2.862554 3.378107 -1.378868
C 4.119345 3.627151 -1.934233
C 5.073985 1.919296 -0.523444
C 5.227846 2.896355 -1.503179
H 1.728041 2.218293 0.025424
H 1.999015 3.953110 -1.700353
H 4.236163 4.396443 -2.692547
H 5.939962 1.357413 -0.179392
H 6.209960 3.093030 -1.923715
H 4.683524 0.408884 1.548693
C 2.785485 -1.045555 2.503594
C 2.437218 -2.425082 1.928725
C 4.156943 -1.122639 3.186860
C 1.734327 -0.623018 3.540146
H 1.726059 -1.331419 4.374874
H 1.958622 0.373232 3.935872
H 0.730006 -0.608397 3.108152
H 4.096918 -1.849916 4.001681
H 4.947467 -1.463705 2.508747
H 4.458230 -0.165681 3.627579
H 2.444857 -3.174993 2.726427
H 1.436841 -2.435840 1.485965
H 3.160999 -2.723190 1.164020

87
D2 (Amide)
Al -1.360185 1.046109 -1.198582
H 0.226336 0.785149 -0.922382
H -1.988110 2.320606 2.282984
H -0.534458 -0.560930 -3.101490
Li 0.851135 -0.144632 0.590885
C -1.563552 1.647545 1.529757
N -2.119872 1.966385 0.202022
C -1.797717 0.208417 1.970330
C -0.938194 -0.814303 1.485157
C -1.277851 -2.118651 1.913091
C -2.350325 -2.409399 2.766642
C -2.867648 -0.061553 2.836393
C -3.148649 -1.368047 3.239657
H -1.565608 -1.320780 -0.566567
H -0.668042 -2.964689 1.582146
H -2.553685 -3.435452 3.071022
H -3.493815 0.752801 3.200426
H -3.978197 -1.567929 3.914472
H -0.474564 1.842923 1.552206
C -2.953402 3.182587 0.140177
C -3.478740 3.380453 -1.286745
C -4.182737 3.081489 1.069042
C -2.136397 4.436694 0.519212
H -2.752988 5.342698 0.469812
H -1.736531 4.366104 1.536167
H -1.291356 4.556874 -0.167747
H -4.810340 3.975472 0.973838
H -4.787420 2.206494 0.810007
H -3.902440 2.995453 2.123140
H -4.130871 4.259737 -1.327355
H -2.664337 3.527468 -2.001495
H -4.065203 2.514919 -1.616260
H -1.557110 1.572606 -2.699273
C -0.870173 -1.430077 -2.523007
N -1.847995 -0.955008 -1.492323
C 0.332950 -2.170088 -1.982081
C 0.204894 -3.433158 -1.387200
C 1.328255 -4.143159 -0.968678
C 2.604363 -3.604914 -1.143038
C 1.618203 -1.639426 -2.148434
C 2.746211 -2.350068 -1.733370
H -0.780702 -3.869323 -1.253251
H 1.206797 -5.121157 -0.510859
H 3.480513 -4.162200 -0.822820
H 1.736577 -0.662416 -2.609152
H 3.733486 -1.919614 -1.876060
H -1.390134 -2.083375 -3.229110
C -3.285691 -1.366704 -1.736503
C -4.119515 -0.840521 -0.566445
C -3.778764 -0.757045 -3.051670
C -3.417844 -2.896026 -1.771895
H -4.470488 -3.164165 -1.907725
H -2.861336 -3.357730 -2.594075
H -3.083781 -3.335677 -0.826102
H -4.833408 -1.012545 -3.194957
H -3.684510 0.332142 -3.043744
H -3.228587 -1.134381 -3.919971
H -5.171411 -1.099842 -0.724915
H -3.796597 -1.278781 0.382869
H -4.034673 0.244671 -0.468868
C 3.907349 0.721637 0.957263
N 2.831985 0.246963 1.463488
C 4.040803 1.380159 -0.351463
C 2.963860 1.945725 -1.047644
C 3.169195 2.566201 -2.276534
C 4.451309 2.634747 -2.826754
C 5.329385 1.476166 -0.901887
C 5.533806 2.088727 -2.135693
H 1.964364 1.907513 -0.628305
H 2.324924 3.002736 -2.802631
H 4.606995 3.122038 -3.785339
H 6.175296 1.060181 -0.358399
H 6.535058 2.147091 -2.553096
H 4.855007 0.656352 1.502078
C 2.880633 -0.342111 2.830659
C 2.466927 -1.814722 2.695007
C 4.242224 -0.263883 3.532487
C 1.839346 0.420653 3.663880
H 1.790535 0.002418 4.674730
H 2.104723 1.480447 3.741575
H 0.843127 0.335955 3.219298
H 4.142773 -0.699858 4.530730
H 5.019816 -0.832123 3.009159
H 4.586052 0.768693 3.661552
H 2.436764 -2.286217 3.682796
H 1.467415 -1.904417 2.258820
H 3.179222 -2.363914 2.070461

Mechanism Li

33
Li1
Al 3.356795 2.759321 -0.038546
H 4.261770 1.479798 0.499479
H 2.912023 2.066501 -1.478643
H 3.929045 4.242393 0.008703
H 1.972613 2.358374 0.783189
Li 2.505544 0.598386 -0.136546
C -0.935548 -0.412574 0.575374
N -1.797019 -0.222584 -0.338537
C 0.410854 -0.937327 0.261420
C 0.814869 -1.168319 -1.065315
C 2.095681 -1.647516 -1.339015
C 2.993222 -1.905569 -0.293437
C 1.318107 -1.192767 1.302452
C 2.600894 -1.676113 1.028910
H 0.111225 -0.951401 -1.862821
H 2.403421 -1.807158 -2.368160
H 3.995099 -2.263281 -0.509924
H 1.022748 -0.996075 2.329993
H 3.297958 -1.852894 1.842078
H -1.117992 -0.205521 1.638231
C -3.132742 0.308386 -0.048952
C -4.125488 -0.737563 -0.581734
C -3.424857 0.601551 1.427437
C -3.255013 1.601213 -0.872622
H -4.267292 2.010217 -0.787845
H -2.546018 2.356634 -0.519143
H -3.044327 1.402681 -1.927322
H -4.442644 0.990738 1.526986
H -3.358449 -0.301253 2.045462
H -2.744756 1.356543 1.837369
H -5.151751 -0.365159 -0.497060
H -3.918413 -0.958536 -1.632752
H -4.049976 -1.671250 -0.014429

33
Li2*
Al -0.277295 2.004944 -0.552043
H -1.643529 2.762756 -0.824696
H 0.605131 1.306617 -1.780523
H -0.556737 -0.214767 1.748603
H 1.099981 3.050768 -0.272752
Li 2.162229 1.777638 -0.981514
C -0.286085 -0.023476 0.699751
N -1.143306 -0.167943 -0.292513
C 1.182665 -0.261081 0.417203
C 1.542874 -1.213410 -0.547105
C 2.883692 -1.443950 -0.849492
C 3.889227 -0.724418 -0.195950
C 2.195932 0.434742 1.097460
C 3.542055 0.209344 0.786360
H 0.746678 -1.725220 -1.077723
H 3.146509 -2.175834 -1.608297
H 4.933968 -0.896997 -0.439413
H 1.920445 1.177375 1.842549
H 4.315998 0.758881 1.316131
H -0.159408 1.593575 1.111096
C -2.559296 -0.436908 0.038457
C -3.339900 -0.311662 -1.274015
C -2.677951 -1.877256 0.566085
C -3.147638 0.539941 1.072337
H -4.205692 0.311450 1.241721
H -2.642099 0.472807 2.042459
H -3.075159 1.573301 0.721278
H -3.724513 -2.135609 0.765170
H -2.281301 -2.586598 -0.167947
H -2.120665 -2.007483 1.501626
H -4.397076 -0.560451 -1.128373
H -3.272840 0.709655 -1.661279
H -2.921845 -0.986999 -2.027489

33
Li3
Al 0.810996 1.872647 -0.158819
H 1.857537 2.987225 -0.853310
H 0.658331 2.779163 1.237035
H 0.258447 -1.826812 -0.638704
H -0.517413 2.550458 -0.887150
Li 0.434529 4.083727 -0.176724
C 0.042771 -0.768200 -0.841885
N 1.057583 0.063645 -0.204484
C -1.371360 -0.505446 -0.356482
C -1.645329 -0.318543 1.004017
C -2.952936 -0.128597 1.449260
C -4.011905 -0.122381 0.539735
C -2.440387 -0.494026 -1.258956
C -3.750573 -0.306221 -0.818178
H -0.819549 -0.314454 1.710309
H -3.146011 0.014342 2.509551

H	-5.030968	0.028558	0.885951
H	-2.242140	-0.624774	-2.320951
H	-4.566227	-0.295181	-1.536747
H	0.048447	-0.679609	-1.942044
C	2.339846	-0.591689	0.114500
C	3.282151	0.464247	0.704620
C	2.152766	-1.698117	1.171432
C	2.996299	-1.183516	-1.147613
H	3.965552	-1.638838	-0.912326
H	2.370432	-1.959666	-1.600869
H	3.155769	-0.398788	-1.895165
H	3.111678	-2.172648	1.409888
H	1.739042	-1.275351	2.092898
H	1.477840	-2.489779	0.830114
H	4.236710	0.013109	0.994763
H	3.498972	1.256359	-0.021836
H	2.845873	0.920515	1.602109

35

Li4

Al	2.108621	1.391261	-0.205249
H	3.716455	1.483732	-0.669160
H	2.374456	2.297589	1.174003
H	-0.157415	-0.985479	-1.780416
H	1.646805	2.696337	-1.121734
Li	3.287804	3.305096	-0.197869
C	-0.100069	-0.070951	-1.170180
N	1.063122	-0.093872	-0.298327
C	-1.459831	0.144154	-0.510977
C	-1.593353	0.832263	0.697813
C	-2.852846	1.075896	1.247112
C	-4.003765	0.633280	0.594412
C	-2.621281	-0.296246	-1.157021
C	-3.882908	-0.056049	-0.613137
H	-0.696865	1.164139	1.214721
H	-2.934903	1.611351	2.189887
H	-4.984626	0.819487	1.023652
H	-2.536757	-0.838537	-2.097737
H	-4.771215	-0.412178	-1.128951
H	0.024533	0.743807	-1.899740
C	1.475081	-1.401896	0.248982
C	2.542945	-1.142930	1.319013
C	0.304047	-2.151326	0.906238
C	2.090811	-2.285740	-0.854923
H	2.406490	-3.257072	-0.455881
H	1.373172	-2.480847	-1.659028
H	2.964088	-1.792006	-1.294673
H	0.653327	-3.101898	1.325513
H	-0.137015	-1.557790	1.712324
H	-0.489300	-2.379731	0.188015
H	2.908153	-2.084438	1.742552
H	3.415451	-0.625343	0.895676
H	2.138089	-0.535690	2.136050
H	4.259761	5.257737	0.057594
H	4.716988	4.949505	-0.446529

35

Li5*

Al	2.637732	-1.432105	-0.072240
H	3.516673	-0.941604	1.325243
H	4.073807	-1.595003	-0.894781
H	0.184093	-0.377883	1.842285
H	2.577608	-2.987306	0.503931
Li	4.454169	-2.426521	0.838124
C	0.046649	-0.734071	0.813085
N	1.099402	-0.206636	-0.070722
C	-1.393543	-0.513022	0.390258
C	-1.834870	-0.905929	-0.879359
C	-3.169385	-0.751514	-1.248081
C	-4.089140	-0.205385	-0.349521
C	-2.324269	0.023137	1.285390
C	-3.663209	0.179129	0.920821
H	-1.124143	-1.332828	-1.583518
H	-3.494780	-1.061044	-2.237889
H	-5.129692	-0.084166	-0.638037
H	-1.998541	0.325406	2.278839
H	-4.370688	0.602403	1.629089
H	0.202791	-1.823642	0.859589
C	1.301582	1.265838	-0.161751
C	2.571282	1.475822	-1.000983
C	0.132531	1.966654	-0.872140
C	1.510712	1.886965	1.227533
H	1.776518	2.945578	1.129589
H	0.601002	1.837235	1.834918
H	2.316388	1.375156	1.763368
H	0.366963	3.028959	-1.006612
H	-0.042908	1.527059	-1.859060
H	-0.796792	1.899113	-0.302676
H	2.757135	2.544226	-1.151034
H	3.455560	1.058144	-0.503109
H	2.474723	1.012409	-1.989272
H	1.169735	-0.974701	-1.180857
H	1.605667	-1.759880	-1.651711

35

Li6

Al	-2.527746	-1.907149	0.039302
H	-3.846830	-0.978036	0.478427
H	-3.628365	-3.252819	-0.154756
H	-0.162317	0.186720	-1.749933
H	-2.675940	-1.624103	-1.603955
Li	-4.517922	-2.028223	-1.059115
C	0.005768	-0.439637	-0.870511
N	-1.009203	-0.096857	0.155208
C	1.454183	-0.365126	-0.433047
C	1.895641	-1.108486	0.670036
C	3.232921	-1.077722	1.062631
C	4.156742	-0.312437	0.349080
C	2.392505	0.386384	-1.148064
C	3.733340	0.414768	-0.762894
H	1.191339	-1.735512	1.214369
H	3.556001	-1.661816	1.920225
H	5.199518	-0.289668	0.653281
H	2.072627	0.956784	-2.017752
H	4.445780	1.007030	-1.331044
H	-0.196674	-1.469136	-1.195825
C	-1.420783	1.341317	0.258132
C	-2.198047	1.473690	1.573508
C	-0.222980	2.302368	0.291259
C	-2.337322	1.686974	-0.918986
H	-2.677435	2.724251	-0.832297
H	-1.824020	1.588434	-1.881149
H	-3.209880	1.029248	-0.928496
H	-0.577385	3.325013	0.461700
H	0.474140	2.046757	1.096125
H	0.334638	2.300642	-0.649363
H	-2.564989	2.498106	1.692456
H	-3.052126	0.791885	1.592184
H	-1.554142	1.251976	2.433809
H	-0.623940	-0.362035	1.060766
H	-1.352919	-2.590680	0.894325

Mechanism Na

6

NaAlH4

Al	-1.046664	0.000002	-0.000032
H	-0.244737	1.272555	-0.711510
H	-0.244760	-1.252393	-0.746444
H	-2.638144	-0.000004	0.000641
H	-0.244177	-0.020174	1.457525
Na	1.543496	0.000000	0.000019

33

Na1

C	1.149232	-0.505050	0.599522
N	1.963341	-0.195415	-0.325638
C	-0.136182	-1.171831	0.298563
C	-1.041610	-1.438059	1.337852
C	-2.272138	-2.045475	1.072757
C	-2.609242	-2.397217	-0.236419
C	-0.480864	-1.535531	-1.016086
C	-1.708421	-2.141191	-1.279461
H	-0.784265	-1.158940	2.356948
H	-2.966789	-2.238058	1.884965
H	-3.565717	-2.867515	-0.444936
H	0.231279	-1.331166	-1.810138
H	-1.965364	-2.420683	-2.297531
H	1.328949	-0.298352	1.663098
C	3.237774	0.475036	-0.047237
C	4.327811	-0.439811	-0.629123
C	3.197635	1.794640	-0.835527
C	3.528956	0.759112	1.430956
H	4.500694	1.253635	1.521724
H	2.781537	1.425585	1.875724
H	3.574186	-0.161473	2.024094
H	4.160809	2.310049	-0.760710
H	2.984729	1.602606	-1.891005
H	2.420272	2.459233	-0.445102
H	5.308177	0.041757	-0.552498
H	4.368193	-1.391148	-0.088228
H	4.123960	-0.653342	-1.682298
Al	-3.437640	2.972417	0.016623
H	-4.397310	1.623508	0.136136
H	-2.223137	2.549396	1.067079
H	-4.123116	4.401752	0.202611
H	-2.683463	2.650669	-1.427575
Na	-2.298673	0.618294	-0.313070

33

Na2*

Al	-0.348257	2.111261	-0.074318
H	-1.734699	2.880891	-0.035629
H	0.406108	1.688609	-1.470072
H	-0.782458	-0.623203	1.672328
H	0.986400	3.035001	0.501110
Na	2.407056	1.710059	-0.552748
C	-0.481328	-0.221864	0.689586
N	-1.331778	-0.152384	-0.326122
C	0.968271	-0.543583	0.388663
C	1.330139	-1.029402	-0.873518
C	2.669456	-1.295675	-1.172088
C	3.662544	-1.093898	-0.206988
C	1.964573	-0.362428	1.360010
C	3.303362	-0.635492	1.067421
H	0.537016	-1.152525	-1.605033
H	2.938829	-1.667203	-2.157491
H	4.702314	-1.312785	-0.435462
H	1.686310	0.021647	2.339371
H	4.063951	-0.498489	1.831838
H	-0.220932	1.133310	1.355532
C	-2.753838	-0.433979	-0.057264
C	-3.524024	-0.006202	-1.311459
C	-2.932886	-1.949284	0.153707
C	-3.325024	0.317937	1.160111
H	-4.390704	0.090742	1.278256
H	-2.829252	0.030963	2.095100
H	-3.218668	1.399451	1.036364
H	-3.989113	-2.208379	0.296458
H	-2.554894	-2.499584	-0.714428
H	-2.384107	-2.294200	1.038753
H	-4.590532	-0.243856	-1.223269
H	-3.418612	1.071374	-1.471944
H	-3.122246	-0.518904	-2.191643

33

Na3

Al	-2.097997	-0.959830	-0.167773
H	-3.692410	-0.740255	-0.623630
H	-2.397460	-1.749812	1.272560
H	0.467137	1.084375	-1.779474
H	-1.740498	-2.276099	-1.112470
Na	-3.718102	-2.967892	-0.117131
C	0.285227	0.191849	-1.159903
N	-0.855814	0.379956	-0.282148
C	1.607512	-0.203893	-0.506584
C	1.651114	-0.876948	0.717291
C	2.867039	-1.288357	1.265026
C	4.064273	-1.033068	0.595449
C	2.814980	0.047548	-1.169577
C	4.033467	-0.361010	-0.627605
H	0.719008	-1.061517	1.244864
H	2.879346	-1.808093	2.220199
H	5.011803	-1.349632	1.023498
H	2.801228	0.575233	-2.122358
H	4.959468	-0.149354	-1.156756
H	0.046600	-0.605175	-1.880479
C	-1.082940	1.741601	0.237484
C	-2.153469	1.655969	1.332896
C	0.188469	2.348640	0.856071
C	-1.598897	2.673971	-0.878703
H	-1.777757	3.688177	-0.501178
H	-0.876918	2.753031	-1.698817
H	-2.536811	2.286844	-1.291191
H	-0.026087	3.346379	1.256522
H	0.564107	1.721663	1.670085
H	0.991209	2.455047	0.119832
H	-2.376838	2.649061	1.737635
H	-3.094844	1.250136	0.938052
H	-1.817146	1.017087	2.156566

35

Na4

Al	-2.105601	-0.603254	-0.165006
H	-3.655991	-0.173350	-0.621271
H	-2.505192	-1.343561	1.277159
H	0.703899	1.083375	-1.781206
H	-1.923098	-1.955113	-1.108347
Na	-3.980255	-2.381074	-0.109031
C	0.408034	0.223884	-1.158821
N	-0.696890	0.561241	-0.279551
C	1.668498	-0.339949	-0.506733
C	1.625165	-1.012492	0.717464
C	2.777615	-1.578647	1.264249
C	3.997273	-1.481853	0.593381
C	2.897766	-0.248120	-1.170902
C	4.053172	-0.811880	-0.629907

H	0.677296	-1.073705	1.245667
H	2.723097	-2.095199	2.219679
H	4.895877	-1.919109	1.020688
H	2.951904	0.276591	-2.123895
H	4.998343	-0.722870	-1.160026
H	0.065821	-0.537079	-1.876634
C	-0.742939	1.941951	0.236937
C	-1.812405	1.999420	1.335306
C	0.598418	2.379622	0.850858
C	-1.136014	2.931225	-0.880449
H	-1.180212	3.960959	-0.505121
H	-0.412095	2.913630	-1.702491
H	-2.117474	2.668611	-1.289660
H	0.516433	3.397428	1.249897
H	0.891745	1.710411	1.665077
H	1.406110	2.379699	0.112354
H	-1.902972	3.014114	1.738026
H	-2.799752	1.718884	0.943925
H	-1.560053	1.323860	2.159706
H	-5.680554	-4.453580	0.226067
H	-6.066786	-4.087543	-0.296614

35

Na5*

Al	2.488747	-0.843892	-0.340970
H	3.292265	-0.327400	1.082782
H	3.817674	-0.617301	-1.308280
H	-0.024573	-0.407290	1.747635
H	2.585211	-2.457660	0.018752
Na	4.784613	-1.783363	0.417981
C	-0.153695	-0.664493	0.688240
N	0.758471	0.120870	-0.157807
C	-1.629768	-0.636566	0.337942
C	-2.062022	-0.947864	-0.957446
C	-3.420136	-0.977027	-1.266376
C	-4.372465	-0.699578	-0.282482
C	-2.591414	-0.369918	1.317368
C	-3.954002	-0.398450	1.012650
H	-1.324372	-1.165712	-1.726766
H	-3.738119	-1.219862	-2.277083
H	-5.431607	-0.721092	-0.524612
H	-2.271538	-0.133802	2.330527
H	-4.686032	-0.183287	1.786859
H	0.175761	-1.711298	0.598881
C	0.716360	1.606726	-0.082957
C	1.893510	2.117957	-0.927557
C	-0.583220	2.180055	-0.671600
C	0.888735	2.096883	1.362802
H	0.978096	3.189176	1.381182
H	0.028060	1.832468	1.986198
H	1.789864	1.665573	1.809936
H	-0.529173	3.274949	-0.686630
H	-0.732512	1.830512	-1.698087
H	-1.461525	1.896791	-0.087661
H	1.891035	3.212536	-0.960071
H	2.854976	1.802298	-0.504615
H	1.830394	1.750809	-1.957783
H	0.875334	-0.475221	-1.326855
H	1.395722	-1.140042	-1.928347

35

Na6

Al	-2.813439	-1.474207	0.574625
H	-3.741722	-0.114903	0.791878
H	-4.117712	-2.571151	0.675913
H	0.091457	-0.106537	-1.639326
H	-2.690416	-1.528260	-1.080721
Na	-5.002491	-1.293838	-0.816199
C	0.250276	-0.503502	-0.632247
N	-0.589329	0.248722	0.301642
C	1.740850	-0.584791	-0.345300
C	2.205338	-1.046013	0.893365
C	3.570648	-1.161734	1.150021
C	4.501460	-0.826588	0.164973
C	2.684793	-0.264109	-1.326839
C	4.053119	-0.381772	-1.078195
H	1.492549	-1.332914	1.664265
H	3.908704	-1.522624	2.117998
H	5.565870	-0.917182	0.363709
H	2.344972	0.084793	-2.300311
H	4.767973	-0.123130	-1.855204
H	-0.144008	-1.528945	-0.654976
C	-0.662364	1.724103	0.185986
C	-1.409980	2.209898	1.433977
C	0.709006	2.418910	0.120512
C	-1.483728	2.079111	-1.060322
H	-1.622919	3.163620	-1.127873
H	-0.988451	1.755315	-1.982074
H	-2.464454	1.597386	-1.009455
H	0.580113	3.507742	0.113532
H	1.327600	2.155259	0.984929
H	1.263823	2.144657	-0.781306
H	-1.560864	3.293673	1.393380
H	-2.384128	1.718889	1.513006
H	-0.836417	1.989711	2.343125
H	-0.345300	-0.002650	1.255479
H	-1.675751	-1.987968	1.575009

Mechanism K

6

KAlH4

Al	-1.588720	-0.000077	0.000000
H	-0.825651	-0.737361	-1.276920
H	-0.825677	-0.736884	1.277237
H	-3.186913	-0.000303	-0.000035
H	-0.827192	1.475205	-0.000254
K	1.385199	0.000018	-0.000001

33

K1

C	-0.089551	-1.326222	0.627710
N	0.833924	-1.159721	-0.236802
C	-1.492382	-0.963994	0.343810
C	-2.360950	-0.675654	1.405699
C	-3.679660	-0.298432	1.157818
C	-4.150581	-0.224553	-0.153152
C	-1.979572	-0.902912	-0.972935
C	-3.300015	-0.535065	-1.218139
H	-1.994398	-0.729355	2.428036
H	-4.338803	-0.060982	1.987593
H	-5.179903	0.063770	-0.345843
H	-1.318434	-1.182493	-1.789672
H	-3.673913	-0.505362	-2.238004
H	0.099049	-1.726256	1.631410
C	2.224609	-1.570562	0.031782
C	2.519513	-2.711649	-0.955922
C	3.109066	-0.359192	-0.299299
C	2.504343	-2.034902	1.465346
H	3.563257	-2.292318	1.561006
H	2.291169	-1.246530	2.195488
H	1.929247	-2.928759	1.731803
H	4.166491	-0.616003	-0.181654
H	2.960860	-0.045084	-1.338682
H	2.892712	0.487705	0.361352
H	3.571549	-3.007331	-0.887937
H	1.900194	-3.587873	-0.737235
H	2.314682	-2.395327	-1.983216
Al	1.256801	3.967872	0.586803
H	-0.356807	3.973272	0.212630
H	1.432962	2.492165	1.320903
H	1.808240	5.234807	1.400591
H	1.943842	3.688746	-0.896631
K	0.209642	1.607249	-0.999951

33

K2*

Al	-0.514093	2.106588	0.049676
H	-1.952935	2.768967	0.159157
H	0.319434	1.745691	-1.304777
H	-1.004482	-0.724828	1.628263
H	0.672222	3.128250	0.732165
K	2.649172	1.747076	-0.222749
C	-0.666832	-0.302110	0.666104
N	-1.520366	-0.112663	-0.339036
C	0.733635	-0.776493	0.339799
C	1.084922	-1.081639	-0.979291
C	2.379911	-1.510496	-1.284776
C	3.335168	-1.653719	-0.273133
C	1.691394	-0.931338	1.352355
C	2.982985	-1.370520	1.052456
H	0.316220	-0.959625	-1.737070
H	2.640342	-1.748825	-2.313264
H	4.334970	-2.009727	-0.508582
H	1.418458	-0.701471	2.381168
H	3.708326	-1.509318	1.850629
H	-0.251638	0.948979	1.347737
C	-2.951670	-0.362602	-0.089062
C	-3.712731	0.245924	-1.272565
C	-3.189108	-1.885351	-0.068550
C	-3.496104	0.244672	1.219201
H	-4.569228	0.040679	1.311217
H	-3.013485	-0.181054	2.107221
H	-3.351682	1.328327	1.236912
H	-4.254168	-2.122584	0.047519
H	-2.831484	-2.335357	-1.000690
H	-2.649721	-2.356244	0.762722
H	-4.786143	0.032111	-1.207326
H	-3.573095	1.331065	-1.295646
H	-3.329817	-0.164443	-2.212883

33

K3

Al	-1.953524	-0.242769	-0.140882
H	-3.423900	0.404403	-0.579334
H	-2.377161	-0.918999	1.318077
H	1.037692	1.089330	-1.785924
H	-1.894454	-1.585384	-1.107001
K	-4.301249	-2.054279	-0.071704
C	0.638856	0.277616	-1.155835
N	-0.409543	0.751017	-0.273079
C	1.823392	-0.435665	-0.506613
C	1.700073	-1.090499	0.721986
C	2.774325	-1.793298	1.269424
C	3.994470	-1.854957	0.594905
C	3.052168	-0.503969	-1.174622
C	4.129701	-1.204979	-0.633039
H	0.752972	-1.027136	1.251487
H	2.658904	-2.292721	2.228709
H	4.832726	-2.398570	1.023037
H	3.168392	0.004101	-2.131177
H	5.076936	-1.238507	-1.166120
H	0.201584	-0.440478	-1.866314
C	-0.283250	2.130585	0.228678
C	-1.321529	2.327087	1.341305
C	1.109289	2.414146	0.820369
C	-0.572676	3.150198	-0.894064
H	-0.488404	4.182196	-0.531137
H	0.131072	3.037250	-1.726216
H	-1.584576	3.000550	-1.285561
H	1.154148	3.439028	1.207655
H	1.332354	1.723946	1.639538
H	1.901502	2.310189	0.072274
H	-1.280735	3.348583	1.735662
H	-2.340368	2.164132	0.965619
H	-1.141262	1.630670	2.167165

35

K4

Al	-1.851199	0.034186	-0.183413
H	-3.235167	0.849599	-0.621729
H	-2.365194	-0.624711	1.254388
H	1.292650	1.063966	-1.760253
H	-1.925857	-1.284167	-1.181235
K	-4.385357	-1.509388	-0.181341
C	0.797798	0.288169	-1.153455
N	-0.206075	0.855523	-0.274250
C	1.887034	-0.564270	-0.505183
C	1.670811	-1.233735	0.702592
C	2.652964	-2.061953	1.247551
C	3.872267	-2.236775	0.591502
C	3.114096	-0.746617	-1.154587
C	4.099896	-1.573265	-0.615329
H	0.726127	-1.082698	1.218500
H	2.466406	-2.570950	2.190469
H	4.639161	-2.878306	1.017891
H	3.302352	-0.229065	-2.094448
H	5.048247	-1.694353	-1.133525
H	0.295265	-0.362811	-1.885116
C	0.063929	2.201290	0.261191
C	-0.964341	2.488016	1.363482
C	1.469395	2.315495	0.878619
C	-0.092301	3.270747	-0.841471
H	0.098496	4.279040	-0.453130
H	0.609082	3.099167	-1.665562
H	-1.107623	3.241962	-1.251225
H	1.620592	3.319892	1.291793
H	1.601657	1.585902	1.683214
H	2.257388	2.141975	0.139022
H	-0.816945	3.489860	1.782080
H	-1.988600	2.447422	0.969918
H	-0.875954	1.758260	2.175413
H	-6.965879	-3.642989	1.046927
H	-7.275583	-3.544314	0.376746

35

K5*

Al	2.204657	-0.438689	-0.464737
H	2.946568	0.105087	0.968757
H	3.438520	0.012650	-1.465789
H	-0.290518	-0.413220	1.683674
H	2.427396	-2.052821	-0.197314
K	4.958999	-1.312332	0.269960
C	-0.427288	-0.614255	0.612853
N	0.363936	0.318572	-0.201937
C	-1.908855	-0.729855	0.306237
C	-2.347008	-1.010849	-0.994295

C	-3.703180	-1.173309	-1.268467
C	-4.647555	-1.061837	-0.244695
C	-2.861410	-0.629704	1.324953
C	-4.222102	-0.792008	1.055085
H	-1.614097	-1.099241	-1.793486
H	-4.025807	-1.390355	-2.283655
H	-5.705438	-1.186971	-0.459810
H	-2.535976	-0.418660	2.341931
H	-4.947525	-0.705051	1.860065
H	0.011989	-1.609003	0.441929
C	0.157894	1.781800	-0.034487
C	1.246614	2.473611	-0.869392
C	-1.213387	2.240007	-0.558773
C	0.315154	2.200912	1.435322
H	0.286255	3.293547	1.520583
H	-0.494721	1.806150	2.058168
H	1.269512	1.843114	1.834123
H	-1.281700	3.333238	-0.507717
H	-1.351274	1.936441	-1.601409
H	-2.038622	1.826148	0.024825
H	1.116394	3.560685	-0.836809
H	2.247589	2.246269	-0.484211
H	1.200121	2.158775	-1.917521
H	0.505649	-0.182377	-1.388260
H	1.073354	-0.764212	-2.058213

35
K6

Al	-3.437555	-0.226023	0.328863
H	-4.430173	1.022810	0.769475
H	-4.196992	-1.509779	1.047321
H	2.232623	0.791087	-2.043027
H	-3.871248	-0.441396	-1.254045
K	-6.339025	-0.549036	-0.254055
C	1.446943	0.323957	-1.438382
N	0.753337	1.361553	-0.691768
C	2.048919	-0.861388	-0.692216
C	1.253747	-1.671274	0.129800
C	1.797888	-2.771236	0.789731
C	3.149936	-3.087933	0.635455
C	3.399106	-1.193610	-0.844247
C	3.949943	-2.296067	-0.186895
H	0.200276	-1.432491	0.261419
H	1.164617	-3.385502	1.424997
H	3.573922	-3.945490	1.151236
H	4.030495	-0.579767	-1.484450
H	5.003119	-2.533153	-0.316103
H	0.715458	-0.069250	-2.157162
C	1.470731	2.246185	0.240449
C	0.466484	3.343195	0.623913
C	1.958498	1.548947	1.527014
C	2.662693	2.889990	-0.477448
H	3.106164	3.665646	0.155773
H	3.450583	2.161880	-0.697936
H	2.342165	3.352269	-1.417334
H	2.414781	2.275807	2.210495
H	1.120137	1.074818	2.049372
H	2.700640	0.775589	1.312335
H	0.914033	4.054541	1.326954
H	0.135048	3.885334	-0.267194
H	-0.417880	2.908459	1.104254
H	-0.087338	0.993983	-0.253874
H	-1.873432	-0.050871	0.628646

Mechanism P

25
P-Imine

C	-0.396713	0.389084	-0.176968
N	0.457833	-0.51719	0.116094
C	-1.843875	0.173991	-0.085838
C	-2.379222	-1.043309	0.366116
C	-3.756131	-1.215696	0.440703
C	-4.618168	-0.178626	0.068097
C	-2.714788	1.2082	-0.457385
C	-4.095391	1.033896	-0.381102
H	-1.695637	-1.836824	0.652008
H	-4.164337	-2.159808	0.790973
H	-5.694103	-0.317666	0.129028
H	-2.304818	2.15294	-0.808253
H	-4.761305	1.841656	-0.671418
H	-0.082257	1.383081	-0.531342
C	1.828004	-0.224688	0.067944
C	2.369005	0.987741	0.527953
C	3.744215	1.207826	0.47487
C	4.597252	0.229312	-0.036594
C	2.695399	-1.218467	-0.411666
C	4.065403	-0.984493	-0.478988
H	1.713547	1.739279	0.959718
H	4.151368	2.145116	0.845044
H	5.668679	0.403913	-0.075088
H	2.270637	-2.163007	-0.738406
H	4.723461	-1.756974	-0.867771

31
P1

Al	3.534562	2.978696	-0.479510
H	4.582800	1.925695	0.255951
H	3.226729	2.019354	-1.797793
H	3.906416	4.514233	-0.655606
H	2.192847	2.521037	0.383254
Li	2.988973	0.734142	-0.256431
C	-0.327253	-0.535609	0.558490
N	-1.196106	-0.651790	-0.370646
C	1.069986	-0.949921	0.361889
C	1.538093	-1.368404	-0.897316
C	2.871556	-1.739354	-1.064324
C	3.758362	-1.699885	0.020787
C	1.966922	-0.907320	1.443167
C	3.302295	-1.282253	1.275381
H	0.841863	-1.380421	-1.729950
H	3.227373	-2.045953	-2.043390
H	4.799904	-1.973940	-0.115778
H	1.619483	-0.563484	2.413915
H	3.989008	-1.229161	2.114325
H	-0.577966	-0.137131	1.551818
C	-2.503701	-0.184529	-0.170872
C	-2.792597	1.006875	0.515159
C	-4.113560	1.424443	0.660154
C	-5.157096	0.663412	0.131195
C	-3.553015	-0.924845	-0.735557
C	-4.871111	-0.512082	-0.566976
H	-1.981600	1.623423	0.892927
H	-4.326942	2.355362	1.178261
H	-6.185383	0.993853	0.245974
H	-3.313503	-1.827193	-1.290094
H	-5.677794	-1.101796	-0.993440

31
P2*

Al	-0.488189	1.781047	1.406058
H	0.822110	2.508719	1.887343
H	-1.205023	0.558454	2.208051
H	0.067990	0.417810	-1.695204
H	-1.899085	2.603576	1.071149
Li	-2.859005	0.817741	1.242494
C	-0.218445	0.218196	-0.640636
N	0.612240	-0.525450	0.136933
C	-1.678937	-0.179241	-0.559909
C	-2.056089	-1.299723	0.193628
C	-3.408067	-1.591832	0.397129
C	-4.400929	-0.775525	-0.162826
C	-2.676289	0.613751	-1.149952
C	-4.031097	0.320111	-0.953223
H	-1.254734	-1.876441	0.646589
H	-3.689903	-2.450160	1.000642
H	-5.451175	-0.998989	0.002010
H	-2.390987	1.489223	-1.728393
H	-4.792627	0.949713	-1.404369
H	-0.264830	1.475014	-0.357303
C	1.966536	-0.362323	-0.050915
C	2.569250	0.627872	-0.866594
C	3.958171	0.716399	-0.985205
C	4.794849	-0.162755	-0.302598
C	2.838630	-1.237424	0.644337
C	4.215303	-1.141562	0.517398
H	1.958017	1.347040	-1.405847
H	4.386534	1.490651	-1.618446
H	5.874266	-0.088483	-0.399001
H	2.389037	-1.994531	1.281552
H	4.850900	-1.835951	1.062651

31
P3

Al	-0.193830	1.770558	-0.491973
H	0.731309	2.899161	-1.296060
H	-0.611562	2.835399	0.718231
H	-0.009507	-1.836324	0.449831
H	-1.540561	2.065348	-1.406360
Li	-0.903261	3.853235	-0.925856
C	-0.343322	-1.097500	-0.295518
N	0.505718	0.086422	-0.198855
C	-1.797508	-0.781052	-0.032177
C	-2.187224	-0.234561	1.198951
C	-3.527109	0.048565	1.459463
C	-4.499382	-0.215903	0.493393
C	-2.781269	-1.044013	-0.990890
C	-4.123369	-0.765583	-0.732210
H	-1.432066	-0.035799	1.955827
H	-3.813037	0.469780	2.419627
H	-5.544488	0.002003	0.696512
H	-2.490775	-1.463543	-1.951362
H	-4.874390	-0.973642	-1.489686
H	-0.256815	-1.587202	-1.279199
C	1.875765	-0.129612	-0.049847
C	2.716838	0.936831	0.338196
C	4.085529	0.761257	0.492961
C	4.675350	-0.488595	0.281849
C	2.483920	-1.384547	-0.262062
C	3.859086	-1.552779	-0.092971
H	2.282937	1.915634	0.534727
H	4.696600	1.608265	0.795167
H	5.744797	-0.626328	0.410147
H	1.890560	-2.234982	-0.579857
H	4.292719	-2.534731	-0.267164

33
P4

Al	1.507320	2.306655	-0.098075
H	2.984639	2.810519	-0.675420
H	1.824023	2.797667	1.457632
H	-0.470523	0.073675	-2.143168
H	0.695577	3.675944	-0.539210
Li	2.368046	4.333128	0.370575
C	-0.535524	0.708256	-1.245234
N	0.729694	0.699854	-0.528987
C	-1.770260	0.308397	-0.448293
C	-1.771300	0.270694	0.947710
C	-2.933229	-0.055404	1.649327
C	-4.112009	-0.346738	0.963868
C	-2.956491	0.007237	-1.128981
C	-4.119749	-0.314863	-0.431927
H	-0.850772	0.484824	1.483283
H	-2.913877	-0.086660	2.735689
H	-5.015634	-0.602552	1.510502
H	-2.968982	0.023495	-2.217766
H	-5.030846	-0.546056	-0.977881
H	-0.700706	1.728105	-1.618760
C	1.334412	-0.528284	-0.262089
H	3.046776	6.282135	1.100132
H	3.479536	6.211435	0.495073
C	2.603515	-0.554386	0.355798
C	3.254839	-1.750241	0.629517
C	2.666552	-2.973538	0.297758
C	0.748027	-1.768956	-0.583284
C	1.411716	-2.964484	-0.307670
H	3.094424	0.383048	0.619710
H	4.232754	-1.724783	1.103842
H	3.174417	-3.909295	0.510996
H	-0.238873	-1.809474	-1.030278
H	0.927913	-3.903048	-0.567214

33
P5*

Al	-2.157025	-2.365909	0.149845
H	-2.832060	-2.170007	-1.412867
H	-3.657069	-2.364711	0.839791
H	0.272375	-1.199067	-1.743193
H	-2.026541	-4.002319	-0.110287
Li	-3.835453	-3.548641	-0.803894
C	0.404447	-1.318216	-0.659392
N	-0.801112	-0.894614	0.054504
C	1.704165	-0.667240	-0.221830
C	1.923118	-0.286372	1.105295
C	3.148025	0.254239	1.495086
C	4.174221	0.417720	0.564087
C	2.737654	-0.497248	-1.149500
C	3.965685	0.038536	-0.761975
H	1.128904	-0.405257	1.837313
H	3.299834	0.549897	2.529711
H	5.127496	0.840626	0.868865
H	2.580190	-0.785868	-2.187268
H	4.756208	0.164608	-1.497022
H	0.496159	-2.402939	-0.486326
C	-1.221584	0.457242	-0.037350
C	-0.845163	-1.512795	1.197564
H	-1.195532	-2.345860	1.743110
C	-2.292484	0.874989	0.772724
C	-2.754189	2.185521	0.719911
C	-2.162913	3.112377	-0.141533
C	-0.631709	1.390944	-0.899570
C	-1.102584	2.703912	-0.946931
H	-2.766269	0.165930	1.448129
H	-3.582622	2.483317	1.357165
H	-2.524424	4.135669	-0.182099
H	0.201493	1.104882	-1.531282
H	-0.628626	3.410519	-1.623257

33

P6

Al	2.409988	2.498703	0.356451
H	2.359509	2.412193	-1.319975
H	3.495914	3.842161	0.215444
H	-0.022294	0.974099	-1.678222
H	1.137070	3.596071	0.469034
Li	2.267502	4.342041	-0.971261
C	-0.385777	1.234318	-0.682024
N	0.668945	0.864446	0.292427
C	-1.725814	0.595066	-0.404946
C	-2.658694	1.249088	0.410238
C	-3.886859	0.658736	0.707633
C	-4.200744	-0.597386	0.187895
C	-2.054372	-0.663420	-0.924747
C	-3.281820	-1.256046	-0.630007
H	-2.422596	2.233582	0.810099
H	-4.600404	1.182126	1.338420
H	-5.158113	-1.058391	0.414840
H	-1.343089	-1.182522	-1.562060
H	-3.521803	-2.232644	-1.041749
H	-0.466911	2.325217	-0.642419
C	1.198093	-0.459344	0.194358
H	0.301177	1.001538	1.231774
H	3.038912	1.568082	1.486936
C	1.817109	-0.883486	-0.987708
C	2.370761	-2.159282	-1.066099
C	2.330907	-3.020777	0.031545
C	1.162300	-1.320608	1.294600
C	1.730664	-2.591107	1.214055
H	1.888399	-0.206467	-1.833691
H	2.850855	-2.475058	-1.988434
H	2.769293	-4.012311	-0.032982
H	0.688619	-0.995262	2.217827
H	1.695967	-3.247305	2.079331

27

P-Amine

H	-0.262771	1.239790	-1.776009
C	-0.528844	1.515751	-0.742749
N	0.643461	1.542934	0.104369
C	-1.648406	0.606850	-0.260977
C	-1.747104	0.213593	1.075594
C	-2.810379	-0.582865	1.502709
C	-3.789816	-0.993853	0.598642
C	-2.629931	0.183880	-1.164478
C	-3.696059	-0.607742	-0.739383
H	-0.975373	0.526998	1.773034
H	-2.870681	-0.886097	2.544677
H	-4.616427	-1.615532	0.931524
H	-2.558431	0.473537	-2.211573
H	-4.449549	-0.928493	-1.453922
H	-0.914113	2.541588	-0.790295
C	1.652511	0.591100	0.013756
H	0.962998	2.462955	0.363950
C	2.911965	0.860494	0.582540
C	3.932590	-0.080966	0.526070
C	3.731126	-1.315444	-0.097198
C	1.450834	-0.652605	-0.608242
C	2.485080	-1.587272	-0.658916
H	3.083548	1.817768	1.071261
H	4.896128	0.154169	0.970888
H	4.530160	-2.049213	-0.140845
H	0.483977	-0.900145	-1.033820
H	2.304897	-2.543400	-1.143658

Mechanism T

29

T-Imine

C	-0.463095	0.396425	-0.000176
N	0.497006	-0.431030	0.000116
C	-1.920997	-0.019695	-0.000001
H	-0.310625	1.488419	-0.000512
C	1.900390	0.004845	-0.000005
C	2.535169	-0.615121	-1.254583
C	2.133541	1.521439	0.000167
C	2.535433	-0.615409	1.254289
H	3.614375	-0.426732	1.274680
H	2.094380	-0.190977	2.162632
H	2.367169	-1.696245	1.268225
H	3.208883	1.726324	0.000101
H	1.707743	2.001283	-0.888393
H	1.707896	2.001013	0.888946
H	3.614084	-0.426303	-1.275219
H	2.367048	-1.695976	-1.268663
H	2.093820	-0.190573	-2.162726
C	-2.579500	0.583965	-1.255199
C	-2.579035	0.583489	1.255671
C	-2.076684	-1.540805	-0.000269
H	-3.653084	0.363960	-1.264635
H	-2.462117	1.673824	-1.286006
H	-2.140029	0.169045	-2.168718
H	-3.139094	-1.810194	-0.000291
H	-1.603667	-1.984565	-0.880940
H	-1.603664	-1.984893	0.880241
H	-3.652554	0.363178	1.265556
H	-2.138995	0.168460	2.168862
H	-2.461941	1.673371	1.286681

35

T1

Al	3.620055	-1.908551	0.000001
H	2.589841	-1.380562	1.225085
H	2.589867	-1.380451	-1.225065
H	3.712123	-3.511138	-0.000126
H	4.970770	-1.040060	0.000074
Li	1.355599	-0.752354	0.000008
C	-1.380773	0.259790	0.000007
N	-0.148456	0.579921	0.000009
C	-1.902444	-1.159005	0.000007
H	-2.167589	1.024527	-0.000016
C	0.293462	1.997066	0.000004
C	1.156749	2.176369	-1.258700
C	-0.839750	3.026436	-0.000157
C	1.156503	2.176470	1.258859
H	1.584026	3.183720	1.281975
H	0.558744	2.035715	2.165255
H	1.985962	1.461496	1.285952
H	-0.407503	4.031198	-0.000185
H	-1.472701	2.944461	-0.890712
H	-1.472856	2.944585	0.890299
H	1.584236	3.183633	-1.281838
H	1.986243	1.461427	-1.285563
H	0.559175	2.035496	-2.165200
C	-2.778498	-1.325266	-1.258105
C	-2.778575	-1.325271	1.258057
C	-0.783964	-2.202999	0.000032
H	-3.238322	-2.319106	-1.265054
H	-3.585612	-0.584663	-1.286045
H	-2.185848	-1.219347	-2.172751
H	-1.208829	-3.211267	0.000053
H	-0.158122	-2.134669	-0.899145
H	-0.158123	-2.134634	0.899209
H	-3.238429	-2.319094	1.264955
H	-2.185977	-1.219391	2.172742
H	-3.585662	-0.584637	1.285981

35

T2*

Al	-0.017900	2.034805	0.344969
H	-1.372499	2.425659	1.096070
H	-0.066758	2.532830	-1.305114
H	0.240403	-1.007160	1.374015
H	1.044703	3.453270	0.587258
Li	0.751606	4.012223	-0.993789
C	0.450748	-0.380020	0.492576
N	-0.535714	0.048504	-0.267216
C	1.878182	-0.603172	-0.012903
H	0.919556	1.137329	1.427906
C	-1.911387	-0.476379	-0.037429
C	-2.864911	0.396129	-0.859646
C	-1.958478	-1.914092	-0.581163
C	-2.352255	-0.475373	1.436207
H	-3.384881	-0.833583	1.510024
H	-1.739672	-1.139266	2.056269
H	-2.305315	0.532984	1.855615
H	-2.972262	-2.323317	-0.503522
H	-1.655808	-1.937243	-1.633137
H	-1.287205	-2.572347	-0.017757
H	-3.883544	-0.004854	-0.818261
H	-2.881903	1.420624	-0.477286
H	-2.543001	0.424722	-1.905505
C	1.822149	-1.995319	-0.684039
C	2.321116	0.432846	-1.044734
C	2.862959	-0.650507	1.159465
H	3.863464	-0.917069	0.801865
H	2.919150	0.320062	1.661653
H	2.563554	-1.399392	1.902387
H	3.288715	0.147539	-1.472567
H	1.590860	0.523746	-1.854081
H	2.450732	1.415375	-0.571400
H	2.822478	-2.275808	-1.032037
H	1.485426	-2.768435	0.016302
H	1.142950	-1.989006	-1.541420

35

T3

Al	-0.974719	1.887609	0.005933
H	-2.410882	2.521346	0.607676
H	-1.075892	2.795895	-1.391480
H	0.658714	-1.252021	1.179579
H	-0.062147	3.024504	0.811651
Li	-1.479140	4.076982	0.004883
C	0.732347	-0.225972	0.795538
N	-0.499677	0.123546	0.085410
C	2.064677	-0.130759	-0.004042
H	0.864873	0.405200	1.691669
C	-1.561562	-0.907526	0.009258
C	-2.665745	-0.374846	-0.914859
C	-1.064539	-2.233210	-0.593270
C	-2.165370	-1.183490	1.401936
H	-2.958816	-1.939366	1.354608
H	-1.401483	-1.548180	2.097358
H	-2.590981	-0.264886	1.821151
H	-1.891741	-2.950076	-0.646211
H	-0.678610	-2.080427	-1.604925
H	-0.275603	-2.695552	0.007105
H	-3.476673	-1.104567	-1.011771
H	-3.115009	0.546475	-0.520768
H	-2.267145	-0.170719	-1.914682
C	2.320040	1.308828	-0.467299
C	3.193499	-0.555801	0.947216
C	2.058787	-1.049659	-1.229331
H	3.283756	1.380030	-0.986280
H	1.549605	1.645355	-1.171732
H	2.338662	2.007299	0.376639
H	3.000684	-0.956042	-1.783319
H	1.943644	-2.101634	-0.944922
H	1.239603	-0.786384	-1.905251
H	4.168459	-0.496313	0.449613
H	3.232711	0.089015	1.833788
H	3.057967	-1.589027	1.289990

37

T4

Al	-1.335850	1.489079	0.026328
H	-2.880906	1.745372	0.633917
H	-1.658409	2.352362	-1.365985
H	1.011443	-1.172156	1.171817
H	-0.720456	2.803687	0.838958
Li	-2.361111	3.490166	0.036213
C	0.833578	-0.156214	0.795022
N	-0.448280	-0.109696	0.088866
C	2.101532	0.263513	-0.005088
H	0.812494	0.482424	1.695433
C	-1.228685	-1.366554	0.004947
C	-2.430432	-1.111066	-0.915748
C	-0.426647	-2.528707	-0.606382
C	-1.745873	-1.790225	1.395528
H	-2.332375	-2.715663	1.342809
H	-0.915385	-1.963360	2.088731
H	-2.380980	-1.004983	1.820667
H	-1.056346	-3.423720	-0.664922
H	-0.089856	-2.280367	-1.616631
H	0.451004	-2.791050	-0.008424
H	-3.039878	-2.015242	-1.018745
H	-3.089172	-0.329266	-0.514949
H	-2.094395	-0.808508	-1.913608
C	2.000394	1.725368	-0.457607
C	3.302181	0.116565	0.941815
C	2.314285	-0.620511	-1.237532
H	2.916454	2.030647	-0.978049
H	1.168436	1.870584	-1.157456
H	1.852949	2.401691	0.391655
H	3.205012	-0.298957	-1.790794
H	2.455876	-1.671487	-0.961503
H	1.454530	-0.556355	-1.911280
H	4.232562	0.413725	0.444001
H	3.186731	0.744931	1.833549
H	3.421279	-0.921375	1.276400
H	-3.470757	5.379287	0.240462
H	-3.174497	5.474109	-0.438743

37

T5*

Al	1.789625	1.679060	0.181272
H	2.328663	1.785846	-1.453064
H	3.329536	2.043990	0.695273
H	-0.638441	0.281875	-1.631840
H	1.255367	3.251650	0.104243
Li	3.024108	3.320456	-0.761902
C	-0.666513	0.583513	-0.575782
N	0.562815	0.145171	0.116634
C	-2.086581	0.273579	-0.006932
H	-0.604941	1.681965	-0.598673

C 1.112491 -1.234752 -0.040045
C 2.629143 -1.146946 0.205039
C 0.536056 -2.194650 1.015228
C 0.885280 -1.790426 -1.451097
H 1.398507 -2.752779 -1.555878
H -0.173775 -1.959802 -1.665162
H 1.287212 -1.107306 -2.206931
H 0.988609 -3.188062 0.915027
H 0.749211 -1.825879 2.024102
H -0.544341 -2.313996 0.922867
H 3.083440 -2.142935 0.168757
H 3.118912 -0.530810 -0.558331
H 2.850888 -0.726600 1.193440
H 0.711451 0.683670 1.356492
H 1.149841 1.381371 1.949185
C -3.012433 1.367191 -0.573685
C -2.641909 -1.078116 -0.482034
C -2.102413 0.357387 1.523003
H -3.666920 -1.216727 -0.118175
H -2.672522 -1.125471 -1.577412
H -2.056760 -1.929802 -0.128535
H -3.124612 0.239483 1.901512
H -1.482854 -0.418859 1.980364
H -1.725598 1.326040 1.870106
H -4.052907 1.185843 -0.281317
H -2.728446 2.359703 -0.205518
H -2.975403 1.391562 -1.669968

37
T6
Al -1.209083 2.210670 -0.384833
H -2.579705 1.966382 0.542012
H -1.617991 3.911187 -0.415214
H 0.750512 -1.146676 1.238608
H -0.231337 2.557876 0.935534
Li -1.809849 3.601822 1.313538
C 0.749534 -0.181020 0.718901
N -0.440557 -0.070565 -0.157303
C 2.119577 -0.040708 0.015232
H 0.671154 0.599048 1.485438
C -1.490014 -1.127521 0.038585
C -2.609698 -0.852851 -0.972420
C -0.941475 -2.541238 -0.223781
C -2.044867 -1.034078 1.463095
H -2.861431 -1.752450 1.589684
H -1.283109 -1.265420 2.214818
H -2.431785 -0.029787 1.654132
H -1.743343 -3.277700 -0.104466
H -0.556727 -2.631451 -1.245854
H -0.141027 -2.819860 0.468067
H -3.376781 -1.630855 -0.900279
H -3.084565 0.113559 -0.785066
H -2.226982 -0.853846 -1.999565
H -0.140281 -0.146161 -1.128246
H -0.958599 1.922198 -1.941334
C 2.251046 1.302718 -0.716805
C 3.188867 -0.112148 1.117610
C 2.357415 -1.190831 -0.975603
H 4.192610 -0.019579 0.688168
H 3.061420 0.697239 1.845395
H 3.144539 -1.064852 1.658960
H 3.353907 -1.104847 -1.423214
H 2.295534 -2.166518 -0.480333
H 1.637323 -1.187208 -1.802109
H 3.248793 1.392795 -1.162288
H 1.521349 1.409853 -1.527564
H 2.105743 2.142917 -0.030839

31
T-Amine
H 0.518124 -0.533998 -1.528388
C 0.522546 -0.427556 -0.427279
N -0.521131 0.464394 0.060543
C 1.941126 0.006742 0.005331
H 0.339894 -1.428538 -0.019024
C -1.919328 -0.004594 -0.002739
C -2.790923 1.198109 0.377617
C -2.326833 -0.506394 -1.401371
C -2.131215 -1.117540 1.032222
H -3.186743 -1.407826 1.067683
H -1.555556 -2.017620 0.793083
H -1.829867 -0.772598 2.026015
H -3.384641 -0.792153 -1.422613
H -2.175058 0.275355 -2.155545
H -1.742746 -1.382954 -1.701050
H -3.850438 0.923205 0.395620
H -2.510483 1.575035 1.366277
H -2.672763 2.014574 -0.346094
H -0.462273 1.348342 -0.437351

C 2.059662 -0.020397 1.534387
C 2.942420 -0.982155 -0.608027
C 2.248604 1.420105 -0.511920
H 3.968079 -0.729914 -0.315430
H 2.743937 -2.007329 -0.272648
H 2.895976 -0.972254 -1.703775
H 3.283360 1.698918 -0.281764
H 2.123180 1.482915 -1.600412
H 1.600232 2.169675 -0.044735
H 3.053430 0.314551 1.854311
H 1.307383 0.629195 1.990891
H 1.908536 -1.035256 1.921814

Mechanism Ga

6
Ga0
Ga -0.198877 0.000000 -0.000009
H 0.633474 1.232732 -0.697327
H 0.633468 -1.220331 -0.718821
H -1.741862 0.000001 0.000399
H 0.633962 -0.012417 1.415997
Li 2.002052 0.000002 0.000007

33
Ga1
Ga -1.362443 2.993704 -0.142659
H -1.335582 1.958433 1.145021
H -0.079244 3.916711 -0.067476
H -2.778842 3.672870 -0.252885
H -1.161796 1.885377 -1.342086
Li -0.916939 0.649339 -0.036337
C 0.574287 -1.778147 0.015108
N -0.607710 -1.294533 0.082325
C 1.783722 -0.945459 0.078797
C 1.794834 0.317175 0.693457
C 2.944270 1.100100 0.668852
C 4.105252 0.620370 0.058693
C 2.964009 -1.430588 -0.504149
C 4.116021 -0.648583 -0.521825
H 0.920632 0.685274 1.227551
H 2.934916 2.079204 1.137688
H 5.002137 1.232869 0.042936
H 2.969363 -2.415797 -0.964920
H 5.020560 -1.027612 -0.988501
H 0.747777 -2.851679 -0.115428
C -1.812208 -2.151013 0.043777
C -2.620098 -1.707085 -1.186985
C -1.532746 -3.653965 -0.032896
C -2.596839 -1.830474 1.326511
H -3.550325 -2.368117 1.330331
H -2.029932 -2.127380 2.214646
H -2.812075 -0.759602 1.404331
H -2.483119 -4.195415 -0.032094
H -1.002930 -3.929974 -0.951300
H -0.954300 -4.007864 0.827652
H -3.566262 -2.255217 -1.234460
H -2.859237 -0.638340 -1.151613
H -2.064244 -1.898837 -2.110249

33
Ga2*
Ga 0.187974 2.240610 0.232357
H 1.705710 2.587756 0.083470
H 0.176607 0.891457 -1.120806
H -0.957991 3.039749 -0.491400
H -0.307926 1.662770 1.654906
Li 0.569339 0.078127 1.957574
C 0.163275 -0.215323 -0.712471
N 1.088925 -0.502077 0.311032
C -1.292816 -0.497395 -0.361321
C -1.614789 -1.535474 0.519725
C -2.946264 -1.849069 0.799113
C -3.977842 -1.119294 0.208802
C -2.336473 0.226312 -0.951734
C -3.666693 -0.083797 -0.674322
H -0.800474 -2.112339 0.951600
H -3.177149 -2.667294 1.476857
H -5.014828 -1.358074 0.428747
H -2.105506 1.041832 -1.633163
H -4.462327 0.491614 -1.140241
H 0.372991 -0.709558 -1.680793
C 2.398162 -1.002351 -0.104827
C 3.251818 -1.075000 1.168420
C 2.297135 -2.419819 -0.706946
C 3.112625 -0.077926 -1.112567
H 4.105298 -0.465244 -1.372251
H 2.546988 0.011933 -2.047596
H 3.232283 0.926767 -0.695301

H 3.279956 -2.808674 -1.001416
H 1.855402 -3.107828 0.022200
H 1.664047 -2.430537 -1.602104
H 4.250623 -1.480100 0.971544
H 3.378402 -0.072871 1.599191
H 2.771357 -1.725296 1.912302

33
Ga3
Ga 0.877121 -1.731516 -0.238418
H -0.163913 -1.769562 -1.528678
H 0.199277 -2.386934 1.036565
H 0.075967 1.601368 1.476715
C -0.083904 0.576382 1.122196
N 0.821403 0.258171 0.004372
C -1.519994 0.478695 0.634928
C -2.017812 1.453885 -0.249314
C -3.307186 1.358538 -0.778738
C -4.117139 0.270899 -0.444362
C -2.344663 -0.606156 0.954232
C -3.636758 -0.700509 0.433194
H -1.399291 2.319807 -0.486009
H -3.679941 2.133616 -1.443396
H -5.119339 0.189760 -0.855559
H -1.964841 -1.381882 1.612209
H -4.261335 -1.549521 0.696953
H 0.045464 -0.088196 1.990280
C 2.138817 0.937796 0.020974
C 2.790560 0.689107 -1.346418
C 2.005066 2.462270 0.191208
C 3.043401 0.385232 1.135990
H 4.013681 0.894967 1.156678
H 2.576482 0.518769 2.118471
H 3.225016 -0.684185 0.987561
H 2.973723 2.940351 0.010923
H 1.289074 2.874002 -0.530532
H 1.690358 2.758071 1.197123
H 3.795678 1.122309 -1.384524
H 2.876442 -0.380884 -1.554541
H 2.196738 1.154545 -2.144411
Li -0.431979 0.018625 -1.456402
H 2.283806 -2.289306 -0.669609

35
Ga4
Ga -1.904666 -0.943474 -0.788121
H -3.402159 -0.395576 -1.207066
H -2.348161 -1.977441 0.411100
H 0.652400 1.653490 -1.276466
H -1.230413 -1.734865 -1.988037
Li -4.313361 -1.686493 0.107179
C 0.496755 0.596587 -1.011440
N -0.686160 0.408024 -0.198616
C 1.810787 0.064488 -0.445765
C 1.835702 -0.960402 0.499615
C 3.043620 -1.481175 0.959233
C 4.252512 -0.980357 0.481572
C 3.030024 0.559848 -0.918390
C 4.240768 0.046212 -0.461188
H 0.889259 -1.335445 0.875736
H 3.041631 -2.280514 1.694762
H 5.194261 -1.382659 0.841717
H 3.031218 1.362662 -1.652629
H 5.176299 0.450054 -0.837135
H 0.357988 0.088034 -1.976663
C -1.080708 1.552896 0.636743
C -2.171660 1.100185 1.612493
C 0.099482 2.080354 1.472650
C -1.643325 2.710020 -0.216630
H -1.940795 3.561224 0.406478
H -0.900803 3.072585 -0.934440
H -2.519189 2.371566 -0.778838
H -0.225806 2.912759 2.105947
H 0.498562 1.291647 2.116006
H 0.915328 2.445616 0.842896
H -2.476798 1.927907 2.260270
H -3.061167 0.758450 1.073052
H -1.810721 0.281782 2.242087
H -2.966768 -6.463593 2.760381
H -3.570071 -6.888621 2.867997

35
Ga5*
Ga 2.425142 -0.974616 -0.076076
H 3.126186 -0.427452 1.380401
H 3.825534 -0.862604 -0.893475
H -0.108754 -0.476273 1.722610
H 2.489614 -2.539077 0.394240
Li 4.267782 -1.753847 0.838751

C -0.256864 -0.678643 0.654187
N 0.709888 0.070478 -0.160708
C -1.724859 -0.547615 0.311613
C -2.175723 -0.769890 -0.995384
C -3.533320 -0.697239 -1.298888
C -4.463609 -0.400844 -0.299529
C -2.664657 -0.269964 1.308282
C -4.025361 -0.187525 1.006540
H -1.453078 -1.000389 -1.775257
H -3.868289 -0.873125 -2.317869
H -5.521822 -0.339256 -0.538578
H -2.328111 -0.104356 2.329903
H -4.741340 0.039590 1.792152
H 0.005659 -1.738926 0.510196
C 0.760404 1.555095 -0.083785
C 1.978046 1.981211 -0.917254
C -0.494001 2.202240 -0.689841
C 0.939803 2.031096 1.364462
H 1.078937 3.117890 1.387078
H 0.059868 1.803582 1.975254
H 1.813982 1.560891 1.824743
H -0.367123 3.290605 -0.714161
H -0.656763 1.853576 -1.714328
H -1.393240 1.982737 -0.110431
H 2.052408 3.072764 -0.950519
H 2.910978 1.600390 -0.486131
H 1.898693 1.612135 -1.945246
H 0.842094 -0.561393 -1.267619
H 1.343441 -1.337537 -1.798314

35
Ga6
Ga 3.012688 -1.185430 -0.114423
H 3.780398 0.239028 -0.361128
H 4.440775 -2.045692 0.011966
H -0.133258 -0.339802 1.513200
H 2.780843 -1.060307 1.496082
Li 4.723867 -0.624364 1.144515
C -0.323430 -0.536497 0.453249
N 0.455993 0.409217 -0.333423
C -1.823955 -0.631630 0.225846
C -2.344731 -0.896317 -1.047204
C -3.718622 -1.008928 -1.251713
C -4.601970 -0.867295 -0.179408
C -2.719428 -0.505862 1.293137
C -4.096847 -0.618076 1.095953
H -1.668476 -1.018824 -1.891200
H -4.101560 -1.214227 -2.248129
H -5.673425 -0.954306 -0.337561
H -2.333473 -0.310996 2.292003
H -4.774489 -0.510848 1.939151
H 0.114762 -1.524583 0.256484
C 0.358589 1.862568 -0.101244
C 1.347922 2.506665 -1.082265
C -1.042213 2.454354 -0.346033
C 0.808350 2.168871 1.332731
H 0.888762 3.251094 1.479559
H 0.096017 1.794177 2.075438
H 1.783235 1.712124 1.528931
H -1.022667 3.543885 -0.222693
H -1.385476 2.236437 -1.363554
H -1.783333 2.050289 0.348521
H 1.358649 3.595034 -0.961901
H 2.357060 2.118099 -0.919746
H 1.063595 2.292446 -2.120378
H 0.367517 0.210196 -1.325235
H 2.025680 -1.873446 -1.100577

Mechanism Bo

6
Bo0
B -0.506897 0.000003 -0.000017
H -0.030240 -0.843462 -0.786302
H -0.028959 -0.258962 1.123108
H -1.705517 -0.000106 0.000910
H -0.029984 1.102596 -0.337724
Li 1.443061 -0.000027 0.000030

33
Bo1
B 0.958611 3.834816 0.287235
H 0.604773 3.339647 -0.798451
H 1.001019 5.036191 0.245964
H 2.065858 3.335733 0.559254
H 0.159368 3.421304 1.146117
Li 0.888031 1.846918 0.367567
C 0.055655 -0.946612 -0.075549
N 1.035331 -0.128014 0.005870
C -1.358270 -0.545428 -0.075116
C -1.786412 0.750039 -0.407091
C -3.138190 1.074775 -0.360345
C -4.081098 0.112066 0.007728
C -2.316753 -1.512152 0.266742
C -3.669047 -1.184407 0.317336
H -1.078511 1.505297 -0.739732
H -3.455453 2.080232 -0.619951
H -5.135638 0.370527 0.042322
H -1.995391 -2.523229 0.506949
H -4.399616 -1.938871 0.594312
H 0.223999 -2.027096 -0.135884
C 2.440262 -0.596677 -0.033699
C 3.095849 -0.120048 1.273079
C 2.613765 -2.111597 -0.173783
C 3.090994 0.114407 -1.232022
H 4.158111 -0.125650 -1.277418
H 2.625979 -0.201482 -2.171307
H 2.993690 1.202223 -1.151505
H 3.681729 -2.345765 -0.204310
H 2.185294 -2.657978 0.673915
H 2.170299 -2.493853 -1.099808
H 4.156921 -0.388319 1.281368
H 3.034639 0.968968 1.381801
H 2.617927 -0.582819 2.142721

33
Bo2*
C -0.342213 0.995843 0.656854
N -1.547023 0.595775 0.108492
C 0.997682 0.392372 0.290507
C 1.468178 0.419048 -1.027704
C 2.714002 -0.114393 -1.345307
C 3.505099 -0.689514 -0.346996
C 1.808476 -0.157555 1.288143
C 3.049780 -0.711566 0.970776
H 0.846403 0.864007 -1.800301
H 3.072479 -0.080444 -2.370701
H 4.476260 -1.108908 -0.595456
H 1.462562 -0.156321 2.319900
H 3.663271 -1.150302 1.753155
H -0.336689 1.252008 1.733509
C -1.912784 -0.811877 -0.067888
C -3.392450 -0.791323 -0.486154
C -1.133650 -1.572671 -1.161878
C -1.797344 -1.589182 1.259230
H -2.174367 -2.614171 1.157242
H -0.753656 -1.653095 1.585553
H -2.372274 -1.085926 2.044584
H -1.578716 -2.561391 -1.329915
H -1.165376 -1.018431 -2.106301
H -0.086828 -1.724721 -0.891156
H -3.788017 -1.803921 -0.622177
H -3.998378 -0.288708 0.277610
H -3.512318 -0.259632 -1.440032
B -0.633630 3.471455 0.031543
H -1.568249 3.560042 0.805544
H -0.134686 2.197636 0.261455
H 0.315162 4.160010 0.266638
H -0.928500 3.281127 -1.135552
Li -2.409810 2.149451 -0.336082

33
Bo3
B -1.040349 -1.487892 1.351321
H 0.149708 -1.819232 1.446749
H -1.720631 -2.454949 1.082584
H -0.425961 0.001188 -1.701110
C -0.146662 -0.585875 -0.818291
N -1.057834 -0.302159 0.300249
C 1.286670 -0.224636 -0.464695
C 1.595991 1.073453 -0.021813
C 2.902219 1.424670 0.331313
C 3.925678 0.479995 0.244976
C 2.326989 -1.157106 -0.547898
C 3.633105 -0.809415 -0.201552
H 0.805790 1.823797 0.005219
H 3.121442 2.438742 0.656129
H 4.942133 0.747407 0.519625
H 2.103320 -2.170118 -0.872450
H 4.423138 -1.552715 -0.267403
H -0.183055 -1.644107 -1.120403
C -2.384201 0.231083 -0.113950
C -3.271184 0.397469 1.126469
C -2.226126 1.622314 -0.754225
C -3.096168 -0.714472 -1.099980
H -4.100072 -0.344137 -1.336480
H -2.549806 -0.803427 -2.045496
H -3.191176 -1.714000 -0.665476
H -3.204157 2.022010 -1.044082
H -1.774323 2.322838 -0.041702
H -1.606668 1.609082 -1.657154
H -4.219784 0.864065 0.839846
H -3.487176 -0.562522 1.600330
H -2.790982 1.041626 1.872828
Li 0.125379 0.113357 1.774762
H -1.339166 -1.015932 2.463696

35
Bo4
B -1.121600 1.676480 -0.080074
H -1.791111 2.264641 0.846640
H -1.575438 2.180702 -1.133850
H -0.252384 -1.352735 1.142290
H 0.018100 2.171923 0.034191
Li -1.089547 3.622048 -0.103701
C -0.063457 -0.297442 0.909509
N -1.070484 0.188287 -0.020654
C 1.362439 -0.212437 0.390879
C 1.647528 -0.280788 -0.976238
C 2.965878 -0.263801 -1.431962
C 4.024741 -0.177323 -0.526405
C 2.430795 -0.118708 1.290307
C 3.751281 -0.103797 0.840172
H 0.814609 -0.331119 -1.671792
H 3.167926 -0.317236 -2.499229
H 5.051924 -0.162968 -0.881608
H 2.225228 -0.052683 2.357653
H 4.566253 -0.029595 1.556168
H -0.103048 0.224694 1.884593
C -2.322575 -0.597705 -0.068983
C -3.296565 0.058590 -1.056477
C -2.039330 -2.019361 -0.589728
C -3.007982 -0.678856 1.311764
H -3.962988 -1.213896 1.247875
H -2.383805 -1.206627 2.041242
H -3.204924 0.326468 1.699636
H -2.970567 -2.593331 -0.657344
H -1.587602 -1.973107 -1.585971
H -1.362365 -2.582057 0.060754
H -4.184465 -0.571923 -1.174011
H -3.626077 1.041664 -0.707855
H -2.827946 0.182866 -2.038083
H -1.000147 5.798835 -0.439088
H -1.357671 5.778695 0.216817

35
Bo5*
B -2.574633 1.650758 -0.097562
H -3.054302 1.051327 1.103817
H -3.611983 1.588989 -0.750920
H -0.471808 1.057466 1.608771
H -2.415682 2.782419 0.369620
Li -4.128447 2.327937 0.944814
C -0.211392 1.137262 0.550250
N -1.337273 0.556833 -0.242652
C 1.182686 0.625705 0.284005
C 1.784329 0.854104 -0.961575
C 3.089038 0.433640 -1.207607
C 3.820612 -0.211045 -0.207037
C 1.930550 -0.002891 1.284977
C 3.240218 -0.421769 1.043078
H 1.218661 1.368833 -1.736476
H 3.540111 0.616046 -2.179408
H 4.838827 -0.538233 -0.399031
H 1.487462 -0.163855 2.265598
H 3.805949 -0.910204 1.832104
H -0.242782 2.197519 0.269063
C -1.618038 -0.936561 -0.149226
C -2.988128 -1.191540 -0.791385
C -0.564517 -1.709839 -0.956809
C -1.622543 -1.424126 1.301078
H -1.902675 -2.483876 1.318109
H -0.633594 -1.341738 1.761192
H -2.342850 -0.863668 1.901501
H -0.841907 -2.769494 -0.977554
H -0.522629 -1.354439 -1.992026
H 0.435193 -1.633999 -0.527337
H -3.154006 -2.271715 -0.859920
H -3.793805 -0.753477 -0.199601
H -3.035544 -0.773460 -1.802091
H -1.165793 0.855804 -1.230458
H -1.774322 2.286681 -1.561513

35
Bo6
B 0.696717 3.701893 0.291709
H -0.289965 3.378621 0.975332

H 1.695358 3.213002 0.848249
H 0.248038 -1.835012 -1.016202
H 0.580317 3.119601 -0.802665
Li 0.530469 1.723558 0.513829
C 0.091834 -0.765098 -0.823883
N 0.979945 -0.260025 0.251389
C -1.349876 -0.524289 -0.445498
C -1.957763 0.713435 -0.694116
C -3.270882 0.953236 -0.288889
C -3.995699 -0.045355 0.361668
C -2.088025 -1.520277 0.205474
C -3.403180 -1.284821 0.605708
H -1.408734 1.497219 -1.214567
H -3.724825 1.920166 -0.485046
H -5.019608 0.140005 0.673619
H -1.633281 -2.491727 0.390372
H -3.965463 -2.069401 1.104526
H 0.331349 -0.225979 -1.745326
C 2.451410 -0.491303 0.080170
C 3.111355 0.037642 1.359211
C 2.784188 -1.981389 -0.088146
C 2.954168 0.307599 -1.126522
H 4.044232 0.233116 -1.188558
H 2.549860 -0.073127 -2.069821
H 2.695022 1.368335 -1.039640
H 3.869201 -2.123383 -0.117558
H 2.395432 -2.571740 0.750478
H 2.378961 -2.394130 -1.017170
H 4.194984 -0.107311 1.316350
H 2.923914 1.108935 1.491872
H 2.742367 -0.493192 2.245632
H 0.703774 -0.744591 1.107035
H 0.789282 4.894339 0.162261

Mechanism AN

58
Li(THF)4 AlH4
Li -0.022951 -0.035203 0.086842
O 1.948022 -0.633607 0.384476
O -0.963294 0.329538 1.879714
O -1.286203 0.176647 -1.584493
O 0.635807 2.004061 -0.204670
C 2.227269 -1.413785 1.566775
C -1.340390 -0.707336 2.808001
C -2.656999 -0.245068 -1.400357
C 1.795999 2.580214 0.401154
C 2.725719 -1.141427 -0.724764
C -1.670881 1.543843 2.190501
C -0.795223 -0.325376 -2.846702
C 0.027536 3.047070 -0.964901
C 3.477245 -2.361674 -0.198167
C -2.534463 1.239856 3.417714
C -2.037522 -0.622687 -3.672984
C 1.197554 3.768618 -1.635566
C 3.571412 -2.075577 1.302852
C -2.699379 -0.281116 3.340437
C -2.999907 -1.131059 -2.595968
C 2.351633 3.590915 -0.621167
H 2.225239 -0.728607 2.420437
H -1.334560 -1.650355 2.257451
H -2.727034 -0.781816 -0.449676
H 1.502401 3.086508 1.333568
H 2.045816 -1.401309 -1.541682
H -0.943323 2.343472 2.365877
H -0.144491 0.442947 -3.277126
H -0.682577 2.579466 -1.648344
H 1.436752 -2.164405 1.691282
H -0.594980 -0.754005 3.615032
H -3.280807 0.658296 -1.361087
H 2.470367 1.757384 0.642108
H 3.404810 -0.341967 -1.050334
H -2.280173 1.814734 1.319723
H -0.217978 -1.241607 -2.666643
H -0.517881 3.725340 -0.288290
H 4.395621 -1.385719 1.522253
H -3.488133 -0.553753 2.630395
H -2.784682 -2.177311 -2.355281
H 2.618386 4.533839 -0.134789
H 2.882835 -3.263833 -0.374326
H -2.006928 1.516510 4.337605
H -2.419972 0.290928 -4.144479
H 1.441721 3.286486 -2.586857
H 3.709092 -2.980254 1.901428
H -2.935475 -0.739163 4.304960
H -4.052616 -1.050229 -2.882224
H 3.251808 3.209090 -1.110423
H 4.451842 -2.486741 -0.678876
H -3.483717 1.782903 3.399994
H -1.844254 -1.360751 -4.456338
H 0.971082 4.818591 -1.842365
Al -0.443082 -3.458589 -0.424307
H -1.933320 -4.013979 -0.763648
H 0.332123 -4.281041 0.749340
H -0.629842 -1.910252 0.146978
H 0.479492 -3.317721 -1.771542

53
Li(THF)4(+)
Li 0.000000 -0.000000 -0.000000
O -1.205900 -1.153057 1.079690
O 1.205976 -1.152975 -1.079693
O 1.205905 1.153053 1.079688
O -1.205981 1.152978 -1.079684
C -2.163915 -1.987147 0.389384
C 2.164036 -1.987014 -0.389390
C 2.163915 1.987147 0.389380
C -2.164037 1.987016 -0.389373
C -1.231059 -1.444566 2.495188
C 1.231158 -1.444472 -2.495194
C 1.231071 1.444557 2.495187
C -1.231170 1.444480 -2.495183
C -2.081697 -2.700229 2.646272
C 2.081870 -2.700083 -2.646283
C 2.081709 2.700219 2.646271
C -2.081882 2.700092 -2.646264
C -3.059054 -2.565956 1.476031
C 3.059214 -2.565763 -1.476037
C 3.059060 2.565952 1.476024
C -3.059220 2.565768 -1.476014
H -2.691081 -1.363131 -0.337963
H 2.691163 -1.362973 0.337964
H 2.691078 1.363134 -0.337972
H -2.691161 1.362973 0.337981
H -0.200581 -1.570696 2.840601
H 0.200689 -1.570659 -2.840613
H 0.200595 1.570685 2.840606
H -0.200703 1.570668 -2.840607
H -1.620150 -2.774762 -0.148175
H 1.620315 -2.774665 0.148161
H 1.620148 2.774764 -0.148173
H -1.620313 2.774664 0.148178
H -1.677249 -0.589050 3.017680
H 1.677301 -0.588926 -3.017677
H 1.677265 0.589039 3.017673
H -1.677315 0.588935 -3.017667
H -3.865329 -1.866449 1.724768
H 3.865449 -1.866207 -1.724765
H 3.865336 1.866444 1.724755
H -3.865456 1.866213 -1.724741
H -1.465895 -3.599396 2.530784
H 1.466120 -3.599287 -2.530806
H 1.465907 3.599386 2.530791
H -1.466131 3.599295 -2.530787
H -3.512496 -3.515824 1.180254
H 3.512709 -3.515607 -1.180265
H 3.512500 3.515821 1.180249
H -3.512713 3.515611 -1.180237
H -2.576192 -2.751557 3.619977
H 2.576373 -2.751375 -3.619987
H 2.576210 2.751544 3.619974
H -2.576389 2.751387 -3.619965

6
AN0 Li(+) ----- AlH4(-)
Al -3.643006 0.042370 -0.491863
H -2.361406 0.091328 0.547571
H -3.940228 1.559590 -1.070678
H -4.977376 -0.513846 0.305333
H -3.293194 -0.967920 -1.749805
Li 20.643762 -0.239989 2.787267

5
AN0
Al 0.000023 0.000000 -0.000003
H 0.953268 1.347798 -0.009257
H 0.953037 -1.347976 -0.006739
H -0.964629 -0.001169 -1.339736
H -0.941978 0.001347 1.355765

32
AN1
C -0.903783 0.488388 0.004510
N -0.062691 -0.464632 0.004539
C -2.361734 0.245886 0.000077
C -2.891022 -1.053923 -0.004091
C -4.267280 -1.253663 -0.008243
C -5.141034 -0.160818 -0.008312
C -3.244061 1.333984 -0.000020
C -4.624956 1.134736 -0.004184
H -2.195830 -1.888249 -0.003998
H -4.665732 -2.265390 -0.011473
H -6.216363 -0.320789 -0.011584
H -2.840868 2.344894 0.003166
H -5.296165 1.989991 -0.004231
H -0.610156 1.547981 0.007644
C 1.387776 -0.216967 0.008721
C 1.942094 -0.910389 -1.246284
C 1.811339 1.255079 0.014350
C 1.936144 -0.918219 1.261967
H 3.029498 -0.854834 1.283413
H 1.541110 -0.447141 2.169782
H 1.638825 -1.972106 1.266515
H 2.904361 1.323775 0.017327
H 1.449969 1.785823 -0.875509
H 1.445140 1.780346 0.905516
H 3.035415 -0.846210 -1.262959
H 1.645256 -1.964347 -1.258605
H 1.551176 -0.433908 -2.153010
Al 6.013794 0.223954 -0.012670
H 7.649896 0.389391 -0.001445
H 5.515370 -0.634492 1.304395
H 5.526360 -0.565673 -1.375945
H 5.291349 1.707388 0.022983

32
AN2*
Al -0.510375 2.601542 -0.541193
H -2.104134 2.796735 -0.513453
H 0.277346 2.199716 -1.873994
H -0.386408 -0.097932 1.611098
H 0.249331 3.818477 0.260734
C -0.191844 0.221598 0.561397
N -1.088035 -0.154286 -0.402333
C 1.276614 -0.069084 0.268738
C 1.629530 -0.799143 -0.866876
C 2.966481 -1.113089 -1.119373
C 3.966987 -0.705930 -0.234849
C 2.284761 0.350549 1.143809
C 3.620230 0.029929 0.902146
H 0.815704 -1.093125 -1.524798
H 3.230072 -1.675977 -2.013066
H 5.008590 -0.953602 -0.429762
H 2.016697 0.941735 2.019035
H 4.392064 0.358090 1.595744
H -0.140728 1.440025 0.848839
C -2.369800 -0.662058 0.060251
C -3.258999 -0.788726 -1.185621
C -2.214203 -2.069855 0.691309
C -3.101950 0.243484 1.080869
H -4.079751 -0.176484 1.357482
H -2.523090 0.355161 2.006968
H -3.254043 1.241502 0.659268
H -3.177657 -2.492179 1.015954
H -1.758691 -2.749865 -0.037528
H -1.556246 -2.032562 1.569294
H -4.240339 -1.223569 -0.947983
H -3.404563 0.199477 -1.634561
H -2.759877 -1.421720 -1.928236

32
AN3
Al 2.051204 2.046440 -0.341369
H 3.627032 1.887767 -0.803967
H 2.008261 2.810752 1.113974
H 0.175453 -0.800196 -1.762416
H 1.190452 2.883509 -1.459659
C 0.065830 0.115645 -1.149437
N 1.178217 0.341902 -0.262021
C -1.325479 0.072986 -0.514527
C -1.596646 0.785185 0.659123
C -2.880435 0.803865 1.204514
C -3.922289 0.104869 0.589186
C -2.377657 -0.621564 -1.123969
C -3.664064 -0.611139 -0.581337
H -0.771869 1.312981 1.132296
H -3.070130 1.367639 2.115981
H -4.922177 0.115550 1.017867
H -2.182543 -1.184876 -2.036596
H -4.463717 -1.166444 -1.068531
H 0.032502 0.944248 -1.874692
C 1.821476 -0.859754 0.277830
C 2.752072 -0.438279 1.424830
C 0.810820 -1.876325 0.849863
C 2.676855 -1.567189 -0.802530
H 3.174492 -2.465334 -0.409068
H 2.057337 -1.878310 -1.652727

H 3.438763 -0.874032 -1.173921
H 1.338557 -2.738966 1.278582
H 0.203588 -1.414969 1.635665
H 0.128438 -2.255719 0.081346
H 3.266219 -1.311465 1.847999
H 3.513097 0.266287 1.070791
H 2.180171 0.054757 2.217735

34
AN4
Al 2.127355 1.662208 -0.800802
H 3.673203 1.290292 -1.237682
H 2.187345 2.703844 0.472137
H -0.007156 -1.243579 -1.591211
H 1.301857 2.331699 -2.049483
C -0.026269 -0.218275 -1.173208
N 1.127779 0.087514 -0.366258
C -1.396094 -0.019196 -0.521666
C -1.573888 0.928103 0.492695
C -2.834845 1.159951 1.042440
C -3.947044 0.444099 0.591854
C -2.518077 -0.728910 -0.967100
C -3.782331 -0.505322 -0.418589
H -0.695870 1.467336 0.840522
H -2.951266 1.904321 1.827877
H -4.929326 0.621691 1.024512
H -2.396238 -1.473561 -1.753802
H -4.638000 -1.075957 -0.775412
H -0.017764 0.448992 -2.049750
C 1.692380 -1.031215 0.395096
C 2.686550 -0.466752 1.420948
C 0.624191 -1.827781 1.173684
C 2.457901 -2.006720 -0.532893
H 2.895932 -2.846149 0.026142
H 1.791247 -2.429162 -1.294792
H 3.260017 -1.466323 -1.046169
H 1.097014 -2.627806 1.759163
H 0.078231 -1.171824 1.859726
H -0.108180 -2.295812 0.506819
H 3.145284 -1.276922 2.003164
H 3.488300 0.089466 0.922175
H 2.178042 0.218316 2.107223
H 3.249746 4.799840 1.084341
H 3.552674 5.442104 1.319178

34
AN5*
Al 2.775557 -1.787698 0.188696
H 3.421107 -0.966314 1.495854
H 4.003995 -1.875611 -0.869516
H 0.266440 -0.373466 1.868575
H 2.130607 -3.147426 0.805117
C 0.099060 -0.757090 0.855635
N 1.191434 -0.341561 -0.030466
C -1.319393 -0.457833 0.410464
C -1.758606 -0.874676 -0.854888
C -3.075911 -0.659207 -1.253722
C -3.981682 -0.024969 -0.397676
C -2.235312 0.167458 1.262952
C -3.557054 0.386047 0.865440
H -1.045195 -1.372404 -1.513073
H -3.400162 -0.989908 -2.238058
H -5.008749 0.144605 -0.712963
H -1.909030 0.490399 2.250239
H -4.251991 0.879154 1.541953
H 0.192390 -1.850264 0.924659
C 1.499449 1.101530 -0.192636
C 2.801331 1.180244 -1.004562
C 0.398901 1.852854 -0.964536
C 1.720756 1.766666 1.172718
H 2.095415 2.788694 1.032613
H 0.789802 1.835585 1.747708
H 2.453236 1.192762 1.749096
H 0.711026 2.891164 -1.137706
H 0.219814 1.381679 -1.936821
H -0.548266 1.873492 -0.419087
H 3.047535 2.227510 -1.219652
H 3.631911 0.731688 -0.450434
H 2.700474 0.642059 -1.952852
H 1.114332 -0.923285 -0.970740
H 1.413787 -2.035265 -1.629829

34
AN6
Al 2.195712 3.257627 -0.096130
H 2.959629 2.577059 1.190182
H 0.752567 3.895135 0.371382
H -0.433787 -1.945807 -1.664908
H 3.138671 4.395847 -0.806717
C -0.196151 -0.890644 -1.475178
N 1.157510 -0.790697 -0.963176
C -1.352531 -0.273030 -0.693825
C -1.303379 1.063661 -0.274414
C -2.377011 1.632993 0.407313
C -3.524439 0.881485 0.676307
C -2.506865 -1.015659 -0.421234
C -3.587884 -0.447621 0.257976
H -0.414768 1.663622 -0.465822
H -2.306064 2.668397 0.730323
H -4.359787 1.327931 1.211090
H -2.559433 -2.055896 -0.741245
H -4.473769 -1.044958 0.464393
H -0.176205 -0.408879 -2.462655
C 1.559949 -1.472153 0.274671
C 3.083655 -1.297514 0.363915
C 0.927326 -0.894593 1.558472
C 1.229847 -2.967081 0.168645
H 1.657789 -3.507553 1.020797
H 0.148995 -3.149129 0.176838
H 1.646092 -3.386931 -0.754398
H 1.331897 -1.399975 2.445630
H 1.150810 0.173526 1.645905
H -0.159343 -1.019335 1.565226
H 3.479092 -1.787233 1.262019
H 3.567604 -1.728116 -0.519329
H 3.342885 -0.234430 0.415756
H 1.476666 0.181312 -0.963136
H 1.868537 2.052069 -1.185293

59
AN7
Al -1.739196 -0.841005 -0.154223
H -1.027967 -2.218141 -0.718621
H -1.239692 -0.621101 1.397122
H -4.956966 -0.370691 -1.893918
H -1.286753 0.445043 -1.067745
C -4.281507 0.079343 -1.141291
N -3.639383 -0.900957 -0.301260
C -5.062245 1.189010 -0.434660
C -4.709292 1.603995 0.854005
C -5.376843 2.660638 1.473681
C -6.417282 3.323283 0.817675
C -6.104879 1.861909 -1.083554
C -6.780061 2.917442 -0.468049
H -3.906223 1.070160 1.356585
H -5.083655 2.969402 2.475204
H -6.940900 4.143956 1.303195
H -6.395343 1.548558 -2.086314
H -7.593148 3.419531 -0.989272
H -3.502657 0.593154 -1.726688
C -4.427547 -2.099544 0.005879
C -3.730144 -2.859459 1.143844
C -5.859965 -1.770706 0.475651
C -4.512359 -3.038565 -1.222268
H -5.091966 -3.946891 -1.004332
H -4.993474 -2.535133 -2.069757
H -3.502515 -3.329312 -1.529799
H -6.398116 -2.693331 0.730755
H -5.837428 -1.126024 1.360337
H -6.438834 -1.254437 -0.297864
H -4.282246 -3.774529 1.394898
H -2.712270 -3.146690 0.856240
H -3.663218 -2.230660 2.037706
C 5.090383 0.459594 -0.291902
N 4.386767 -0.430386 0.281512
C 6.561260 0.503048 -0.156395
C 7.256680 -0.439391 0.616727
C 8.641461 -0.374995 0.726903
C 9.357627 0.630366 0.068161
C 7.286398 1.506171 -0.812340
C 8.675675 1.571588 -0.702402
H 6.682506 -1.211572 1.120098
H 9.169742 -1.110507 1.328708
H 10.440110 0.677598 0.156642
H 6.753745 2.240564 -1.413506
H 9.223989 2.356261 -1.217627
H 4.659967 1.251007 -0.922242
C 2.922999 -0.468562 0.143096
C 2.357619 -0.365872 1.568968
C 2.309519 0.626475 -0.735586
C 2.586983 -1.850408 -0.440629
H 1.501771 -1.990075 -0.487556
H 2.994377 -1.949522 -1.453456
H 3.021835 -2.639658 0.181264
H 1.222349 0.505669 -0.771253
H 2.515822 1.628881 -0.340499
H 2.679786 0.575828 -1.767042
H 1.268499 -0.480599 1.554730
H 2.790684 -1.144940 2.204809
H 2.599167 0.608508 2.009247

59
AN8*
Al -0.084672 -0.777994 -1.572513
H -0.767451 -2.065989 -2.210893
H -3.285168 0.880759 -1.557833
H 0.628268 0.286372 -2.555862
C -2.442531 0.563152 -0.900304
N -2.710391 -0.451341 0.006077
C -1.858146 1.850442 -0.319271
C -1.941612 2.101877 1.051701
C -1.470106 3.302718 1.586300
C -0.906940 4.272471 0.754944
C -1.278548 2.822156 -1.145288
C -0.811838 4.025791 -0.617860
H -2.383661 1.318448 1.662341
H -1.539077 3.481330 2.657994
H -0.537738 5.207891 1.170529
H -1.183595 2.623058 -2.211889
H -0.365564 4.769204 -1.275499
H -1.682157 0.307332 -1.788117
C -3.939647 -1.191018 -0.226894
C -4.012752 -2.284851 0.849078
C -5.200140 -0.298958 -0.064436
C -4.011493 -1.878081 -1.613861
H -4.935016 -2.463685 -1.734922
H -3.983075 -1.139141 -2.424907
H -3.152568 -2.543880 -1.746898
H -6.131506 -0.865073 -0.215676
H -5.213858 0.136002 0.941594
H -5.200633 0.527968 -0.785756
H -4.946597 -2.860401 0.783057
H -3.169639 -2.975803 0.745397
H -3.948318 -1.827461 1.843081
H 1.692120 0.691138 1.198200
C 1.811775 0.299174 0.176439
N 0.959808 -0.855256 -0.059929
C 3.304668 0.132268 -0.094163
C 3.780848 -0.748364 -1.070396
C 5.143606 -0.836069 -1.355270
C 6.062297 -0.041178 -0.666833
C 4.235585 0.926061 0.587933
C 5.600342 0.843608 0.309173
H 3.062193 -1.371101 -1.595839
H 5.490529 -1.528789 -2.119252
H 7.125191 -0.112051 -0.886083
H 3.882772 1.617020 1.352578
H 6.303857 1.466557 0.857785
H 1.480835 1.117798 -0.478451
C 0.788212 -1.770685 1.089625
C 0.067201 -3.031761 0.602232
C 2.141976 -2.197927 1.687679
C -0.079141 -1.120084 2.185086
H -0.223053 -1.810890 3.027240
H 0.391195 -0.213102 2.583988
H -1.054335 -0.855952 1.757199
H 1.974697 -2.905729 2.509173
H 2.770772 -2.684318 0.934201
H 2.702099 -1.349308 2.094071
H -0.065801 -3.735619 1.432742
H -0.922580 -2.776459 0.211170
H 0.639984 -3.530457 -0.188508

59
AN9
Al 0.003180 -1.388897 -1.054118
H 0.094387 -2.989001 -1.339013
H -2.990601 0.326418 -2.134580
H 0.485716 -0.473234 -2.319195
C -2.180150 0.404300 -1.384529
N -1.830949 -0.870748 -0.798320
C -2.554324 1.538482 -0.430794
C -2.058506 1.575557 0.876451
C -2.351110 2.643797 1.724595
C -3.150902 3.700197 1.282247
C -3.353167 2.604227 -0.864198
C -3.652169 3.675361 -0.020729
H -1.446146 0.741059 1.208056
H -1.952549 2.652221 2.737174
H -3.382608 4.531205 1.944785
H -3.751251 2.590193 -1.878674
H -4.281190 4.488391 -0.378520
H -1.311234 0.763755 -1.959916
C -2.958301 -1.800816 -0.626749
C -2.544365 -2.922851 0.335725
C -4.207617 -1.125502 -0.022221
C -3.353481 -2.445389 -1.977908

H -4.206166 -3.131471 -1.872534
H -3.633395 -1.678201 -2.709877
H -2.501607 -3.002772 -2.380856
H -5.001150 -1.869511 0.126711
H -3.973317 -0.671779 0.946245
H -4.611571 -0.339779 -0.669220
H -3.366998 -3.636249 0.476316
H -1.680549 -3.467718 -0.056210
H -2.279242 -2.504797 1.313278
H 1.564702 1.055573 1.175576
C 1.669387 0.512511 0.218473
N 1.242873 -0.869125 0.303502
C 3.081114 0.779327 -0.305294
C 3.677231 -0.075838 -1.238172
C 4.939091 0.204929 -1.761848
C 5.634411 1.348430 -1.360541
C 3.784361 1.925236 0.085262
C 5.049428 2.210797 -0.431690
H 3.127185 -0.963681 -1.537595
H 5.382794 -0.471848 -2.489473
H 6.620738 1.564133 -1.765615
H 3.334029 2.601754 0.811413
H 5.580274 3.103291 -0.105605
H 1.000987 1.043964 -0.476285
C 1.551482 -1.535759 1.574639
C 1.323789 -3.045496 1.426646
C 3.020969 -1.339995 2.007564
C 0.640696 -1.026503 2.719029
H 0.873987 -1.518769 3.673524
H 0.757119 0.053300 2.869251
H -0.408400 -1.222158 2.475119
H 3.218263 -1.889818 2.937345
H 3.702745 -1.708839 1.234646
H 3.262644 -0.287647 2.189163
H 1.562738 -3.559732 2.366656
H 0.285161 -3.270799 1.170635
H 1.952478 -3.452696 0.628787

61
AN10
Al -0.001437 -1.406075 0.966060
H -0.095123 -3.011024 1.216721
H 2.983678 0.287190 2.102688
H -0.490873 -0.521357 2.252197
C 2.177832 0.381437 1.349678
N 1.832548 -0.880791 0.734010
C 2.557467 1.536340 0.423301
C 2.074434 1.599647 -0.887634
C 2.372035 2.686518 -1.710049
C 3.163991 3.735633 -1.237468
C 3.348657 2.594981 0.887148
C 3.652523 3.684610 0.069603
H 1.467954 0.770682 -1.242982
H 1.983457 2.715223 -2.726123
H 3.399504 4.581271 -1.879826
H 3.736637 2.560607 1.905031
H 4.275271 4.491794 0.450788
H 1.305620 0.728579 1.927550
C 2.961197 -1.806949 0.548570
C 2.552950 -2.907251 -0.441082
C 4.213893 -1.118403 -0.033408
C 3.348254 -2.481060 1.887472
H 4.201858 -3.164338 1.772182
H 3.623305 -1.730224 2.637979
H 2.494314 -3.047573 2.272893
H 5.008339 -1.859005 -0.193761
H 3.985570 -0.643528 -0.993093
H 4.613856 -0.347188 0.633206
H 3.376167 -3.617787 -0.592164
H 1.686558 -3.460444 -0.066864
H 2.294262 -2.467664 -1.410892
H -1.548473 1.091013 -1.212234
C -1.659063 0.527017 -0.268044
N -1.235432 -0.853434 -0.382078
C -3.072631 0.785363 0.254983
C -3.677930 -0.092298 1.160538
C -4.941561 0.179711 1.684588
C -5.629382 1.336951 1.310859
C -3.768535 1.944684 -0.108189
C -5.035256 2.221719 0.409257
H -3.133803 -0.990767 1.438299
H -5.392342 -0.514649 2.390940
H -6.616974 1.546088 1.716251
H -3.311001 2.638798 -0.812901
H -5.560214 3.125263 0.104845
H -0.992599 1.041401 0.441295
C -1.539071 -1.490915 -1.669380
C -1.315921 -3.004137 -1.553510
C -3.005675 -1.281571 -2.105496
C -0.620861 -0.958771 -2.797212
H -0.850463 -1.428896 -3.763658
H -0.733514 0.124446 -2.923807
H 0.426340 -1.162912 -2.552201
H -3.199188 -1.809758 -3.048492
H -3.692668 -1.665989 -1.344913
H -3.243711 -0.224771 -2.264667
H -1.550700 -3.496859 -2.505966
H -0.279343 -3.237772 -1.296535
H -1.950252 -3.427283 -0.768499
H -0.638329 -0.914404 4.762457
H -0.688320 -0.979137 5.504801

61
AN11*
Al 0.109791 -0.722734 -1.054525
H -0.177095 -2.200306 -1.719159
H -3.321123 -1.432549 -1.134617
H -0.199217 0.393034 -2.186527
C -2.799011 -0.489064 -0.937886
N -1.713987 -0.707658 0.030912
C -3.812713 0.590598 -0.608133
C -3.413970 1.815732 -0.055641
C -4.350853 2.822015 0.178895
C -5.697991 2.629710 -0.136557
C -5.164726 0.408393 -0.924660
C -6.102759 1.416105 -0.692865
H -2.360114 1.968836 0.187462
H -4.022792 3.765739 0.608744
H -6.424483 3.417483 0.049250
H -5.489368 -0.537555 -1.355666
H -7.148649 1.250028 -0.942208
H -2.320659 -0.206282 -1.887408
C -1.991751 -1.645618 1.152064
C -0.773244 -1.610154 2.085175
C -3.234120 -1.223806 1.954646
C -2.180320 -3.078621 0.630718
H -2.260068 -3.774200 1.475305
H -3.094449 -3.183228 0.035186
H -1.333627 -3.369528 0.003082
H -3.374473 -1.902190 2.805667
H -3.122563 -0.205100 2.339361
H -4.144864 -1.256257 1.348492
H -0.929815 -2.294884 2.927134
H 0.136504 -1.911543 1.556148
H -0.611278 -0.600441 2.474029
H -1.259429 0.252452 0.397956
H -0.328696 1.139493 0.486153
H 2.633034 -0.575324 1.655701
C 2.236446 0.011858 0.805434
N 1.855574 -0.814806 -0.322204
C 3.194858 1.159986 0.531043
C 3.082962 1.908999 -0.647952
C 3.904742 3.012299 -0.874387
C 4.859815 3.392663 0.072722
C 4.148445 1.556343 1.476326
C 4.976391 2.658597 1.254253
H 2.337481 1.606833 -1.378789
H 3.796715 3.583283 -1.794567
H 5.503626 4.250897 -0.107605
H 4.247581 0.985753 2.399528
H 5.716868 2.940280 2.000859
H 1.310653 0.499907 1.158943
C 2.797456 -1.897679 -0.690903
C 2.761201 -2.122229 -2.214268
C 4.264592 -1.581678 -0.335738
C 2.410779 -3.212585 0.022896
H 3.101812 -4.032637 -0.220571
H 2.425973 -3.071306 1.110937
H 1.400051 -3.510768 -0.273183
H 4.900302 -2.409707 -0.674173
H 4.606227 -0.666139 -0.827141
H 4.422925 -1.466019 0.741169
H 3.438570 -2.938353 -2.501911
H 1.752216 -2.378321 -2.548175
H 3.076470 -1.209765 -2.732617

61
AN12
Al 1.288240 -2.043397 -1.179219
H 1.339858 -3.674459 -0.994900
H -4.736120 -0.795805 -1.493379
H 1.348304 -1.661961 -2.771525
C -3.656135 -0.642707 -1.372062
N -3.097711 -1.763414 -0.637064
C -3.424876 0.779112 -0.870370
C -2.126668 1.250623 -0.630071
C -1.911851 2.560624 -0.205915
C -2.992678 3.427035 -0.018245
C -4.497704 1.657455 -0.684090
C -4.288572 2.972369 -0.259993
H -1.279625 0.579472 -0.763159
H -0.896752 2.903836 -0.024119
H -2.822911 4.448349 0.314279
H -5.512238 1.307015 -0.870451
H -5.137539 3.637583 -0.116455
H -3.231381 -0.708187 -2.382935
C -3.568704 -2.104681 0.711643
C -2.927240 -3.461595 1.039629
C -3.162833 -1.090512 1.802417
C -5.094751 -2.261366 0.703365
H -5.435348 -2.665206 1.663566
H -5.604304 -1.302833 0.553410
H -5.404775 -2.948676 -0.091874
H -3.467811 -1.447781 2.794940
H -2.076544 -0.953676 1.807554
H -3.624689 -0.112987 1.637851
H -3.209453 -3.794564 2.045693
H -3.243195 -4.216577 0.312525
H -1.835068 -3.391168 0.995657
H -2.076726 -1.747035 -0.662288
H -0.110865 -1.449445 -0.533198
H 2.758369 -0.474462 1.837752
C 2.261320 -0.260451 0.872638
N 2.654522 -1.166381 -0.176643
C 2.433756 1.234446 0.594130
C 2.435301 1.724689 -0.716047
C 2.526926 3.094188 -0.967878
C 2.623169 4.003342 0.088422
C 2.527832 2.155125 1.645427
C 2.623887 3.525986 1.400889
H 2.371710 1.002986 -1.527197
H 2.521918 3.454239 -1.994751
H 2.699002 5.070535 -0.108160
H 2.529996 1.789835 2.672307
H 2.703885 4.221802 2.234027
H 1.186918 -0.401817 1.070391
C 4.031818 -1.668385 -0.107230
C 4.368824 -2.345434 -1.444066
C 5.066976 -0.546908 0.119491
C 4.184385 -2.719681 1.019077
H 5.209667 -3.111827 1.077051
H 3.940613 -2.287123 1.997129
H 3.497724 -3.552772 0.836586
H 6.083247 -0.962742 0.128868
H 5.005821 0.201295 -0.677533
H 4.913511 -0.030865 1.073522
H 5.390255 -2.747249 -1.428545
H 3.682694 -3.175937 -1.646361
H 4.283962 -1.628699 -2.267455

86
AN13
Al -1.268692 -0.379316 0.565705
H -0.149146 -0.762017 1.689512
H -2.353063 -3.020261 -1.660482
H -0.584184 -0.000207 -0.870286
C -2.687896 -2.052949 -1.239934
N -2.355873 -1.905867 0.160476
C -4.151032 -1.868144 -1.641635
C -5.014551 -1.070263 -0.884461
C -6.332152 -0.854856 -1.289318
C -6.815775 -1.436940 -2.463223
C -4.646428 -2.445337 -2.817784
C -5.963785 -2.235917 -3.228314
H -4.626522 -0.632180 0.031348
H -6.985561 -0.228811 -0.684968
H -7.844114 -1.272302 -2.777011
H -3.988660 -3.073477 -3.418219
H -6.327426 -2.700887 -4.142679
H -2.118474 -1.297807 -1.806085
C -2.411653 -3.151347 0.942598
C -2.408806 -2.808317 2.438534
C -3.687928 -3.974660 0.668691
C -1.180630 -4.043385 0.650983
H -1.215402 -4.987477 1.212814
H -1.121172 -4.294108 -0.414845
H -0.265779 -3.507660 0.923721
H -3.702403 -4.868047 1.306493
H -4.584461 -3.383572 0.881457
H -3.750757 -4.312258 -0.371068
H -2.439295 -3.722220 3.045760
H -1.506565 -2.250094 2.705626
H -3.283528 -2.198457 2.690235
H -3.770001 2.207290 -0.009461
C -2.699284 1.956511 -0.118922
N -2.169368 1.237498 1.022243
C -1.973377 3.233082 -0.544477

C -0.599362 3.387646 -0.334850
C 0.069400 4.527049 -0.782104
C -0.624902 5.539369 -1.448971
C -2.658209 4.251628 -1.218267
C -1.996385 5.396149 -1.666292
H -0.073927 2.595198 0.190594
H 1.139346 4.626098 -0.610259
H -0.103903 6.429983 -1.793339
H -3.729367 4.147062 -1.389512
H -2.552104 6.178152 -2.180215
H -2.658948 1.296808 -0.999384
C -2.705398 1.657351 2.323788
C -1.862965 1.035290 3.444532
C -2.659935 3.188324 2.518898
C -4.170073 1.191836 2.512605
H -4.573674 1.507820 3.484519
H -4.822736 1.607243 1.735802
H -4.226375 0.100282 2.452154
H -3.030496 3.453421 3.517801
H -1.635288 3.560148 2.419148
H -3.278355 3.717624 1.786674
H -2.244543 1.347383 4.425162
H -1.884998 -0.057033 3.404416
H -0.817121 1.345446 3.356705
C 5.846356 -0.423128 -0.553857
N 5.225807 -0.581478 0.544489
C 7.321421 -0.450050 -0.632261
C 8.115349 -0.653204 0.506888
C 9.501942 -0.673621 0.403086
C 10.120898 -0.491841 -0.838394
C 7.949524 -0.268891 -1.871172
C 9.340474 -0.289244 -1.976153
H 7.616025 -0.792143 1.461151
H 10.107568 -0.831635 1.292101
H 11.205094 -0.508287 -0.915669
H 7.339941 -0.110474 -2.758662
H 9.813199 -0.146907 -2.944598
H 5.334769 -0.256628 -1.512495
C 3.757293 -0.553426 0.611867
C 3.396251 0.567547 1.599840
C 3.031420 -0.323119 -0.717304
C 3.331049 -1.904892 1.207238
H 2.251308 -1.906454 1.386641
H 3.575257 -2.724150 0.521667
H 3.850967 -2.083859 2.153950
H 1.949413 -0.316044 -0.553523
H 3.299247 0.640557 -1.167078
H 3.248809 -1.116695 -1.442601
H 2.316758 0.569425 1.780120
H 3.916633 0.417631 2.551306
H 3.687937 1.545106 1.199824

86
AN14*
Al 0.182494 -0.783708 0.369626
H 0.539175 -1.984310 1.344099
H -2.275485 -2.215952 -1.487208
C -2.303715 -1.563101 -0.588167
N -2.337107 -2.168752 0.640791
C -3.340910 -0.474391 -0.853572
C -4.258916 -0.126274 0.138423
C -5.231663 0.845935 -0.103256
C -5.309350 1.472701 -1.348511
C -3.424129 0.158330 -2.100021
C -4.401675 1.120885 -2.351591
H -4.175831 -0.655941 1.083967
H -5.936173 1.112855 0.682336
H -6.071867 2.224820 -1.539647
H -2.717415 -0.111939 -2.883993
H -4.458196 1.595310 -3.329230
H -1.267451 -0.919993 -0.848877
C -2.290142 -3.624788 0.660974
C -2.144278 -4.031986 2.134315
C -3.610234 -4.228213 0.117472
C -1.117272 -4.257901 -0.127604
H -1.130908 -5.354148 -0.050513
H -1.165353 -4.009394 -1.195076
H -0.161222 -3.896003 0.262302
H -3.621141 -5.327339 0.173747
H -4.457242 -3.838053 0.693002
H -3.762969 -3.947176 -0.932472
H -2.152504 -5.123546 2.260063
H -1.205808 -3.635390 2.535510
H -2.967323 -3.602898 2.717209
H -1.915582 2.109166 0.864185
C -0.936437 1.856833 0.426882
N -0.248554 0.835264 1.196374
C -0.193573 3.165386 0.166315
C 1.197046 3.261088 0.262592

C 1.858394 4.453798 -0.033526
C 1.136719 5.579164 -0.434519
C -0.907652 4.300994 -0.238819
C -0.253838 5.496557 -0.536226
H 1.745223 2.379296 0.582872
H 2.942171 4.503629 0.047837
H 1.650318 6.509954 -0.663648
H -1.992347 4.243766 -0.319567
H -0.830291 6.366258 -0.844747
H -1.196442 1.454076 -0.561048
C -0.472048 0.929867 2.667144
C 0.574259 0.084392 3.409543
C -0.314424 2.374275 3.183935
C -1.877202 0.419492 3.043140
H -2.016007 0.413653 4.133037
H -2.655783 1.063850 2.617524
H -2.031336 -0.590169 2.644748
H -0.485334 2.388480 4.267367
H 0.691396 2.760452 2.990655
H -1.032693 3.065482 2.732391
H 0.467845 0.227529 4.491942
H 0.462177 -0.979741 3.193995
H 1.588262 0.385260 3.121210
H 2.650949 -2.663926 -1.493462
C 2.492556 -1.967079 -0.653798
N 1.510568 -0.942402 -0.968422
C 3.863815 -1.501108 -0.172428
C 4.026413 -0.298099 0.521328
C 5.273275 0.081351 1.018929
C 6.388259 -0.737272 0.829842
C 4.989492 -2.314796 -0.354219
C 6.240167 -1.941191 0.138610
H 3.156579 0.339255 0.654658
H 5.374559 1.021114 1.557493
H 7.361102 -0.440110 1.214269
H 4.882856 -3.254874 -0.894569
H 7.100524 -2.587910 -0.020864
H 2.089278 -2.586505 0.162149
C 1.527477 -0.503225 -2.376453
C 0.657532 0.745084 -2.536383
C 2.945729 -0.134000 -2.857953
C 0.961525 -1.601883 -3.305661
H 0.970343 -1.278553 -4.354986
H 1.545670 -2.526674 -3.242531
H -0.069616 -1.836346 -3.022528
H 2.906866 0.226970 -3.893424
H 3.373702 0.655867 -2.233015
H 3.629061 -0.988607 -2.831652
H 0.672656 1.092887 -3.576284
H -0.384103 0.532333 -2.276449
H 1.017736 1.557716 -1.899522

86
AN15
Al -0.602175 -0.343340 0.761998
H -1.653569 -0.736635 1.953637
H -0.344352 3.221637 0.725653
C 0.245049 2.373542 0.329035
N 0.323621 1.266652 1.262433
C 1.540170 2.960775 -0.226916
C 2.651922 2.161340 -0.514561
C 3.799061 2.702675 -1.094775
C 3.861263 4.063832 -1.400965
C 1.615422 4.323731 -0.543599
C 2.759645 4.874566 -1.122503
H 2.610839 1.104295 -0.272969
H 4.646022 2.055076 -1.309424
H 4.757143 4.487196 -1.849384
H 0.761801 4.964676 -0.324606
H 2.793025 5.938191 -1.350577
H -0.326203 2.020446 -0.542757
C 0.448528 1.677318 2.678742
C 0.806386 0.455771 3.538332
C 1.559309 2.725000 2.892157
C -0.881961 2.256679 3.216669
H -0.778763 2.601814 4.254642
H -1.215859 3.112783 2.618746
H -1.660934 1.488255 3.181981
H 1.672678 2.938613 3.963070
H 2.517137 2.358176 2.508897
H 1.339734 3.672004 2.388642
H 0.809884 0.730067 4.600914
H 0.077963 -0.349164 3.400979
H 1.800087 0.075306 3.283570
H 2.294476 -1.950356 1.922318
C 2.023344 -1.500331 0.947909
N 0.654509 -1.786183 0.564639
C 3.160148 -1.843201 -0.013659
C 2.999191 -1.834327 -1.402983

C 4.077002 -2.075884 -2.255526
C 5.347396 -2.331568 -1.736009
C 4.441573 -2.100165 0.493987
C 5.524862 -2.341889 -0.351186
H 2.010414 -1.642738 -1.804778
H 3.923197 -2.062330 -3.332574
H 6.187026 -2.522239 -2.400449
H 4.588297 -2.115375 1.573533
H 6.507101 -2.543862 0.071489
H 2.088565 -0.415067 1.116381
C 0.222902 -3.174032 0.852123
C -1.122159 -3.425068 0.159347
C 1.211497 -4.229092 0.317335
C 0.036777 -3.415395 2.369478
H -0.260175 -4.453140 2.573933
H 0.966213 -3.226983 2.919442
H -0.734613 -2.745592 2.761176
H 0.810253 -5.235850 0.492198
H 1.374446 -4.104801 -0.757903
H 2.186757 -4.172930 0.811471
H -1.506677 -4.420723 0.414074
H -1.869820 -2.686004 0.463352
H -1.010134 -3.368455 -0.928025
H -2.845462 1.962441 -0.675998
C -2.830492 0.974763 -0.181453
N -1.767825 0.116889 -0.680662
C -4.260104 0.441511 -0.192481
C -4.570332 -0.782578 0.416282
C -5.881098 -1.252241 0.452237
C -6.915609 -0.508461 -0.124580
C -5.305813 1.181561 -0.754563
C -6.622802 0.713976 -0.727739
H -3.761727 -1.352102 0.866060
H -6.099925 -2.203037 0.933770
H -7.937908 -0.879171 -0.101762
H -5.083401 2.138289 -1.225428
H -7.417227 1.303676 -1.180806
H -2.601687 1.193196 0.872383
C -1.728320 -0.086529 -2.135790
C -0.327747 -0.575456 -2.516198
C -2.750174 -1.147056 -2.612614
C -1.993601 1.209248 -2.935109
H -1.825269 1.027080 -4.003573
H -3.024950 1.561844 -2.825975
H -1.318150 2.012945 -2.620095
H -2.656605 -1.323877 -3.693453
H -2.583337 -2.094165 -2.091703
H -3.777405 -0.831032 -2.411741
H -0.273500 -0.812793 -3.586032
H 0.423296 0.192071 -2.295789
H -0.069967 -1.467829 -1.939899

88
AN16
Al -0.567250 -0.351675 0.727765
H -1.641256 -0.737720 1.901550
H -0.379813 3.216170 0.608389
C 0.236226 2.370685 0.248389
N 0.312246 1.288984 1.211072
C 1.533542 2.970255 -0.289020
C 2.667216 2.186314 -0.529924
C 3.818112 2.736397 -1.094236
C 3.862064 4.091028 -1.431040
C 1.590755 4.326675 -0.636032
C 2.738542 4.886248 -1.199303
H 2.640113 1.134566 -0.264457
H 4.682391 2.100726 -1.272402
H 4.760751 4.521358 -1.867034
H 0.719833 4.955657 -0.453526
H 2.757418 5.944727 -1.451672
H -0.305125 1.984596 -0.628491
C 0.392628 1.736700 2.619306
C 0.751000 0.543755 3.517949
C 1.477621 2.810686 2.834509
C -0.962044 2.303036 3.108476
H -0.892486 2.675426 4.139785
H -1.296342 3.137555 2.481018
H -1.725228 1.518969 3.073169
H 1.559312 3.052659 3.902265
H 2.451747 2.453233 2.484922
H 1.253307 3.740586 2.302058
H 0.723179 0.844327 4.573037
H 0.040777 -0.277761 3.383212
H 1.757437 0.175765 3.297088
H 2.330172 -1.869233 2.000455
C 2.075328 -1.448992 1.008483
N 0.722888 -1.772512 0.597785
C 3.243219 -1.791793 0.084865
C 3.117814 -1.820788 -1.307824

C	4.221853	-2.060708	-2.126621
C	5.483422	-2.276673	-1.569004
C	4.516159	-2.009076	0.630946
C	5.625468	-2.248888	-0.180325
H	2.135960	-1.660093	-1.739056
H	4.095484	-2.077111	-3.207191
H	6.343560	-2.466049	-2.207057
H	4.635379	-1.994308	1.713878
H	6.600455	-2.419581	0.271888
H	2.114376	-0.358786	1.151168
C	0.311917	-3.161545	0.908911
C	-1.008043	-3.457815	0.186422
C	1.336186	-4.208648	0.428491
C	0.088904	-3.368639	2.426360
H	-0.192110	-4.407019	2.649307
H	0.998768	-3.146692	2.996198
H	-0.706629	-2.705795	2.779761
H	0.951041	-5.218872	0.618828
H	1.525981	-4.108628	-0.644878
H	2.296138	-4.119064	0.947056
H	-1.379979	-4.454177	0.456594
H	-1.778154	-2.726439	0.450298
H	-0.866787	-3.426813	-0.898576
H	-2.820230	1.870626	-0.823518
C	-2.796999	0.895897	-0.304245
N	-1.704238	0.049319	-0.755083
C	-4.214312	0.331531	-0.337553
C	-4.513509	-0.884694	0.292204
C	-5.814516	-1.381900	0.307345
C	-6.849941	-0.674384	-0.311944
C	-5.261233	1.035407	-0.942296
C	-6.568250	0.539853	-0.936591
H	-3.704314	-1.425920	0.774680
H	-6.024991	-2.325872	0.805723
H	-7.864398	-1.066612	-0.305178
H	-5.047596	1.985614	-1.430115
H	-7.363532	1.101459	-1.422618
H	-2.599740	1.144979	0.749186
C	-1.622610	-0.187854	-2.203339
C	-0.202302	-0.654348	-2.535803
C	-2.608575	-1.281876	-2.679983
C	-1.895234	1.082234	-3.040527
H	-1.694962	0.878453	-4.099524
H	-2.936609	1.413862	-2.966525
H	-1.246120	1.908293	-2.728049
H	-2.484254	-1.481152	-3.753789
H	-2.433966	-2.212893	-2.133280
H	-3.647196	-0.984589	-2.511650
H	-0.115058	-0.916201	-3.597707
H	0.525998	0.134786	-2.315292
H	0.059584	-1.526575	-1.931185
H	-6.500081	1.957089	3.541377
H	-6.876675	2.305243	4.080255

88

AN17*

Al	-0.040592	-0.379448	0.567974
H	-0.842369	-0.931137	1.883281
H	-2.324436	2.331050	0.967694
C	-1.409105	2.320050	0.357265
N	-0.283652	1.734850	1.079865
C	-1.229395	3.700220	-0.261783
C	-0.047256	4.053416	-0.925216
C	0.079158	5.291960	-1.552944
C	-0.976292	6.206852	-1.533270
C	-2.282151	4.623368	-0.255991
C	-2.161732	5.865443	-0.881999
H	0.774111	3.340983	-0.948521
H	1.006182	5.542916	-2.063693
H	-0.875455	7.173599	-2.020834
H	-3.209688	4.365815	0.253406
H	-2.992637	6.567317	-0.856661
H	-1.622719	1.640239	-0.477503
C	-0.063367	2.234353	2.457874
C	1.252826	1.637568	2.965896
C	0.057904	3.767787	2.538378
C	-1.203142	1.779283	3.391685
H	-1.001724	2.088487	4.425640
H	-2.162426	2.223470	3.101132
H	-1.303346	0.690942	3.354856
H	0.305574	4.063184	3.566286
H	0.846823	4.136619	1.874886
H	-0.872848	4.273043	2.264138
H	1.423880	1.924278	4.010366
H	1.236327	0.546184	2.909112
H	2.097252	2.000834	2.370804
H	0.604773	1.487030	0.137933
H	0.961189	0.948219	-0.631446
H	3.374753	-0.298458	1.727253
C	2.896551	-0.361960	0.730517
N	1.710183	-1.191075	0.736772
C	4.012796	-0.656305	-0.272422
C	3.777189	-1.312552	-1.483356
C	4.800530	-1.495346	-2.414045
C	6.088193	-1.023419	-2.152194
C	5.309609	-0.188385	-0.020756
C	6.338747	-0.366635	-0.945676
H	2.776597	-1.686378	-1.673002
H	4.590713	-2.009695	-3.349725
H	6.886611	-1.167698	-2.876532
H	5.514731	0.321343	0.920456
H	7.337959	0.002514	-0.722703
H	2.590006	0.665751	0.494818
C	1.860490	-2.445117	1.520411
C	0.728670	-3.409426	1.142519
C	3.185318	-3.181665	1.233933
C	1.787831	-2.186878	3.043728
H	1.919708	-3.117667	3.612389
H	2.574655	-1.493596	3.365434
H	0.815522	-1.756846	3.301670
H	3.211513	-4.115014	1.810838
H	3.280075	-3.432861	0.173448
H	4.064454	-2.595067	1.519286
H	0.830940	-4.355584	1.689673
H	-0.242461	-2.974917	1.391745
H	0.753858	-3.622458	0.068280
H	-3.281839	-0.002538	-0.875604
C	-2.690106	-0.733262	-0.291613
N	-1.325725	-0.854727	-0.770349
C	-3.546527	-1.992364	-0.162259
C	-2.983342	-3.247506	0.083853
C	-3.787876	-4.370697	0.275988
C	-5.179218	-4.261788	0.225468
C	-4.943156	-1.895467	-0.211040
C	-5.755355	-3.014115	-0.020254
H	-1.901320	-3.324448	0.108537
H	-3.325511	-5.337046	0.466619
H	-5.806486	-5.138099	0.372411
H	-5.400262	-0.925766	-0.406904
H	-6.837818	-2.912962	-0.069056
H	-2.649241	-0.320960	0.728506
C	-1.182230	-0.885508	-2.239382
C	0.244845	-1.319827	-2.582089
C	-2.142718	-1.889342	-2.910048
C	-1.423167	0.500459	-2.882883
H	-1.347136	0.446590	-3.977239
H	-2.422340	0.886375	-2.646641
H	-0.680309	1.219192	-2.524324
H	-1.954607	-1.913095	-3.991174
H	-1.996586	-2.898400	-2.512928
H	-3.194602	-1.622260	-2.764634
H	0.386907	-1.357250	-3.669734
H	0.966732	-0.613986	-2.163695
H	0.451246	-2.311487	-2.165788

88

AN18

Al	0.338510	-0.502767	0.334968
H	0.021640	-1.523128	1.570139
H	-5.281340	-1.792066	-1.820998
C	-4.445229	-1.191177	-1.441561
N	-3.540827	-2.050394	-0.700939
C	-5.014931	0.057172	-0.774849
C	-4.164544	1.013695	-0.203226
C	-4.683163	2.170126	0.375620
C	-6.062327	2.396316	0.391517
C	-6.392933	0.297102	-0.758462
C	-6.917943	1.455465	-0.179989
H	-3.089298	0.841263	-0.206954
H	-4.007086	2.898322	0.816515
H	-6.464252	3.298453	0.846317
H	-7.066272	-0.435401	-1.201695
H	-7.993368	1.619216	-0.173392
H	-3.887220	-0.863985	-2.329303
C	-3.993162	-2.816754	0.468554
C	-2.839379	-3.770240	0.812595
C	-4.307241	-1.955862	1.710414
C	-5.231150	-3.641719	0.093285
H	-5.483392	-4.329386	0.908205
H	-6.108767	-3.009252	-0.082594
H	-5.039331	-4.230746	-0.810640
H	-4.566066	-2.593909	2.565439
H	-3.434458	-1.356453	1.988896
H	-5.144408	-1.275316	1.530804
H	-3.091587	-4.386162	1.684034
H	-2.624022	-4.427367	-0.036294
H	-1.928859	-3.207945	1.048624
H	-2.657987	-1.576266	-0.511358
H	-1.020136	-0.275334	-0.571304
H	-0.546530	2.927884	0.876584
C	0.172952	2.340375	0.273419
N	0.751720	1.237705	1.006225
C	1.130178	3.339216	-0.379050
C	2.454980	2.999794	-0.666502
C	3.303269	3.898738	-1.313694
C	2.840887	5.162472	-1.686300
C	0.677234	4.609306	-0.758017
C	1.519358	5.514882	-1.404193
H	2.798803	2.015346	-0.359963
H	4.330387	3.611196	-1.528923
H	3.502014	5.865320	-2.188096
H	-0.351795	4.893197	-0.538534
H	1.145690	6.497836	-1.684265
H	-0.436225	1.927187	-0.546665
C	1.007723	1.501061	2.436561
C	1.983268	0.443946	2.973474
C	1.650996	2.880131	2.689543
C	-0.303270	1.420629	3.254273
H	-0.134394	1.633240	4.319223
H	-1.039746	2.144771	2.885141
H	-0.735106	0.418995	3.162619
H	1.845327	3.006016	3.762273
H	2.601516	2.974947	2.155691
H	1.006202	3.707249	2.373987
H	2.175011	0.603607	4.042435
H	1.576091	-0.563707	2.848812
H	2.937507	0.497011	2.439766
H	2.170695	-3.463603	-0.589709
C	2.318322	-2.508005	-0.051862
N	1.665021	-1.393909	-0.702224
C	3.814678	-2.381730	0.236867
C	4.437014	-1.134080	0.334171
C	5.790265	-1.028745	0.656765
C	6.552661	-2.175568	0.887980
C	4.589486	-3.525109	0.471051
C	5.943729	-3.428731	0.793066
H	3.829881	-0.253404	0.141851
H	6.252491	-0.046268	0.728666
H	7.608490	-2.094704	1.136254
H	4.122542	-4.506887	0.394786
H	6.525898	-4.332021	0.965018
H	1.833708	-2.666200	0.924910
C	1.657804	-1.447274	-2.176176
C	1.255498	-0.071189	-2.723705
C	3.040983	-1.787311	-2.768774
C	0.638043	-2.491767	-2.688480
H	0.628695	-2.545087	-3.786045
H	0.876230	-3.494016	-2.312500
H	-0.367506	-2.232391	-2.343383
H	2.987875	-1.786241	-3.865081
H	3.790513	-1.051868	-2.460163
H	3.395178	-2.775864	-2.457767
H	1.237024	-0.082701	-3.820901
H	0.258068	0.211916	-2.373559
H	1.965311	0.695879	-2.398802

References

[S1] S.-N. Trzaska, Alkalimetallbortetrahydride und -Aluminiumtetrahydride, Universtät Hamburg, **2008**.

[S2] N. Momiyama, H. Nishimoto, M. Terada, *Org. Lett*. **2011**, 13, 2126–2129.

[S3] X. Cattoën, S. Solé, C. Pradel, H. Gornitzka, K. Miqueu, D. Bourissou, G. Bertrand, *J. Org. Chem*. **2003**, 68, 911–914.

[S4] Rigaku Oxford Diffraction, **2018**, CrysAlisPro Software system, version 1.171.39.46, Rigaku Corporation, Oxford, UK.

[S5] O. V. Dolomanov, L. J. Bourhis, R.J. Gildea, J. A. K. Howard and H. Puschmann, *J. Appl. Cryst*., **2009**, 42, 339–341.

[S6] G. M. Sheldrick, *Acta Cryst. A*, **2015**, 71, 3–8.

[S7] G. M. Sheldrick, *Acta Cryst*. **2008**, A64, 112-122.

[S8] A. Thorn, B. Dittrich and G. M. Sheldrick, *Acta Cryst. A*, **2012**, 68, 448–451.

[S9] Rigaku Oxford Diffraction, **2019**, CrysAlisPro Software system, version 1.171.40.67a, Rigaku Corporation, Oxford, UK.

[S10] M. J. Frisch, G. W. Trucks, H. B. Schlegel, G. E. Scuseria, M. A. Robb, J. R. Cheeseman, G. Scalmani, V. Barone, G. A. Petersson, H. Nakatsuji, X. Li, M. Caricato, A. V. Marenich, J. Bloino, B. G. Janesko, R. Gomperts, B. Mennucci, H. P. Hratchian, J. V. Ortiz, A. F. Izmaylov, J. L. Sonnenberg, D. Williams-Young, F. Ding, F. Lipparini, F. Egidi, J. Goings, B. Peng, A. Petrone, T. Henderson, D. Ranasinghe, V. G. Zakrzewski, J. Gao, N. Rega, G. Zheng, W. Liang, M. Hada, M. Ehara, K. Toyota, R. Fukuda, J. Hasegawa, M. Ishida, T. Nakajima, Y. Honda, O. Kitao, H. Nakai, T. Vreven, K. Throssell, J. A. Montgomery, J. E. Peralta, F. Ogliaro, M. J. Bearpark, J. J. Heyd, E. N. Brothers, K. N. Kudin, V. N. Staroverov, T. A. Keith, R. Kobayashi, J. Normand, K. Raghavachari, A. P. Rendell, J. C. Burant, S. S. Iyengar, J. Tomasi, M. Cossi, J. M. Millam, M. Klene, C. Adamo, R. Cammi, J. W. Ochterski, R. L. Martin, K. Morokuma, O. Farkas, J. B. Foresman, D. J. Fox, Gaussian 16 Rev. A.03, Wallingford CT, **2016**.

[S11] A. D. Becke, *J. Chem. Phys*. **1993**, 98, 1372–1377.

[S12] J. P. Perdew, Electronic Structure of Solids, Akademie Verlang, Berlin, **1991**.

[S13] W. J. Hehre, L. Radom, P. v. R. Schleyer, J. A. Pople, Ab Initio Molecular Orbital Theory, John Wiley, New York, **1986**.

[S14] R. C. Binning, L. A. Curtiss, *J. Comput. Chem*. **1990**, 11, 1206–1216.

[S15] T. Clark, J. Chandrasekhar, G. W. Spitznagel, P. v. R. Schleyer, *J. Comp. Chem*. **1983**, 4, 294–301.

[S16] G. Scalmani, M. J. Frisch, *J. Chem. Phys*. **2010**, 132, 114110-1–16.

[S17] H. Elsen, F. Christian, G. Ballmann, S. Harder, *Angew. Chemie - Int. Ed*. **2018**, 57, 7156–7160.
